# How Alkali Metal Alkoxides Initiate Organic Radical Reactions

**DOI:** 10.1021/jacs.5c22122

**Published:** 2026-02-20

**Authors:** Seb Tyerman, Kenneth F. Clark, Alexander J. Stewart, Krystian Kolodziejczak, Craig M. Robertson, Laura Evans, Alan R. Kennedy, Tell Tuttle, David J. Nelson, John A. Murphy

**Affiliations:** † Department of Pure and Applied Chemistry, 3527 University of Strathclyde, 295 Cathedral Street, Glasgow G1 1XL, U.K.; ‡ GSK Medicines Research Centre, Gunnels Wood Road, Stevenage, Hertfordshire SG1 2NY, U.K.; § AstraZeneca, Oncology Targeted Discovery, Oncology R&D, The Discovery Centre, Cambridge Biomedical Campus, 1 Francis Crick Avenue, Cambridge CB2 0AA, U.K.

## Abstract

Alkali metal alkoxides have long been known to cause hydrodehalogenation of aryl halides; this conversion of aryl halides to arenes happens when the reactions are conducted in appropriate solvents (with weak C–H bonds). More recently, when aryl halides are heated with alkoxides in arene solvents, coupling to arenes occurs. Both of these reaction types are known to involve aryl radical intermediates. The consensus has been that alkali metal alkoxides undergo electron transfer to aryl halides to form radicals, but crucial evidence has been missing. We now refute this proposal and show through deuterium isotope studies that the deprotonation of the substrates leads to benzynes that initiate radical chemistry. Surprisingly, *o*-, *m*-, *p*- and, in appropriate cases, *r*- (remote) benzynes are simultaneously formed. During reactions with potassium *tert*-butoxide, we observed for the first time low-level methylation of arenes, resulting from methyl radicals derived from *tert*-butoxide. Although methyl radicals could, in principle, arise by electron transfer from *tert*-butoxide ions, followed by known radical fragmentation, we show that a different, previously unreported mechanism applies.

## Graphical abstract

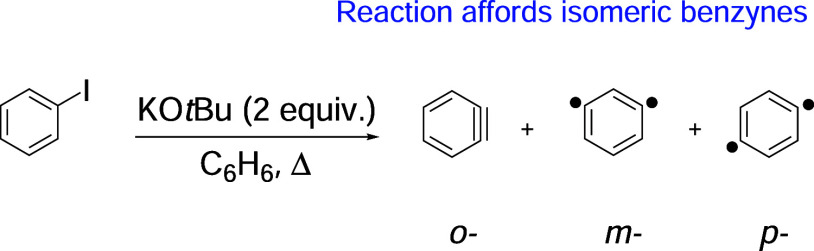

## Introduction

Activation of aryl halides to convert the C–Hal bond to a C–H or C–C or C–heteroatom is central to organic chemistry. Conversion of aryl halides **1** to arenes **4** on heating with alkoxides **3** (Scheme 1A) has been reported since 1899.^1^ In 1992, Bunnett reviewed known cases and proposed a radical mechanism for the propagation steps of the reactions but avoided any comment on the source of the radicals (i.e., the initiation of the reactions).^2^ More recently, when aryl halides were heated with an alkali metal *tert*-alkoxide above 100 °C in an arene solvent, coupling to the arene was observed to give a biaryl. This type of coupling reaction is termed a base-promoted homolytic aromatic substitution (BHAS) reaction (Scheme 1B) and again proceeds through aryl radical intermediates. Thus, the two types of reactions, hydrodehalogenation and BHAS coupling, are linked by the reaction of alkali metal alkoxides with aryl halides and the formation of aryl radical intermediates.

**Scheme 1:**
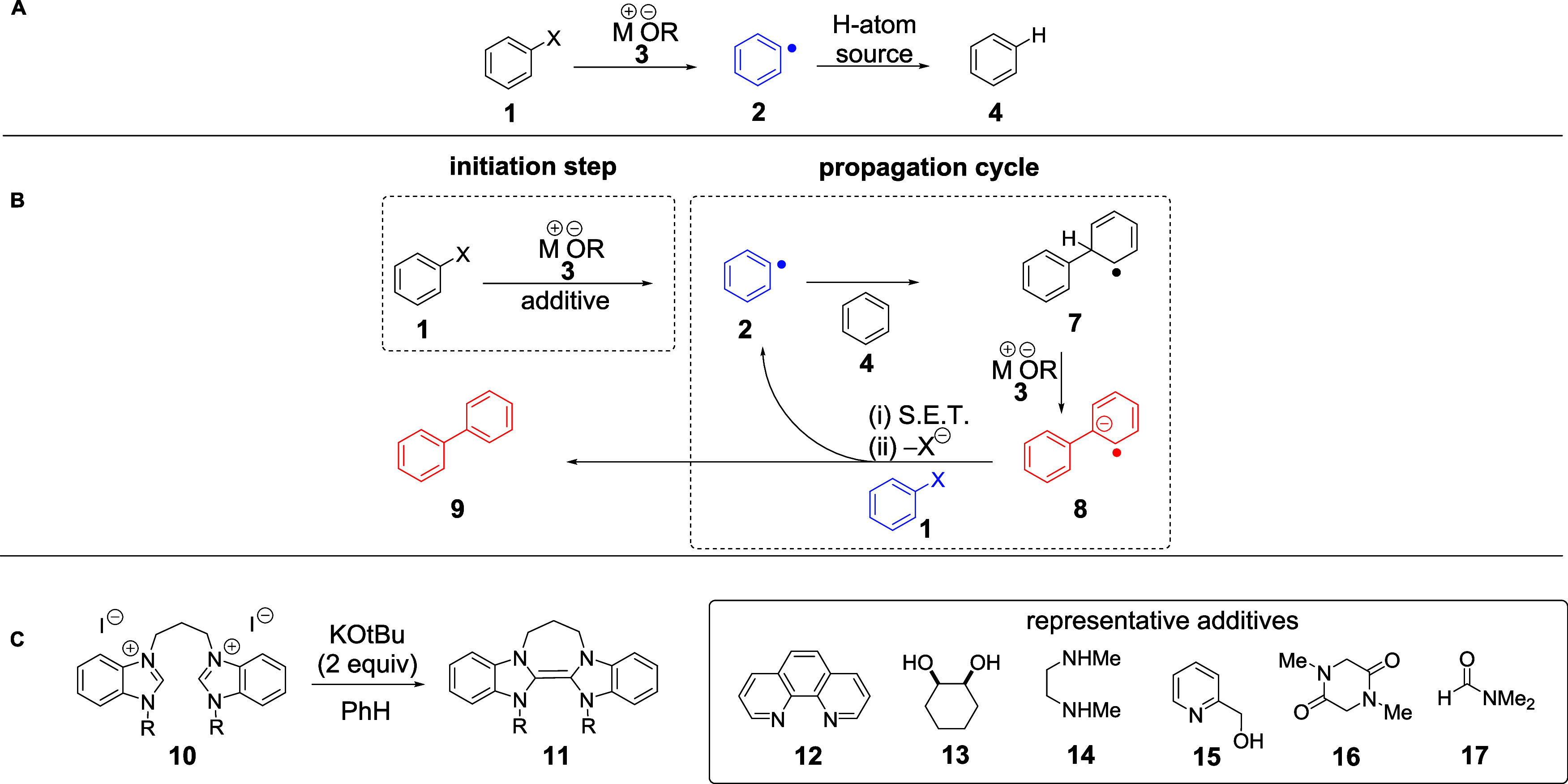
(A) Protodehalogenation of Haloarenes **1** to Arene **4**; (B) BHAS Coupling of Aryl Halide **1** to Form Biaryl **9**; and (C) Additives **10**, **12**–**17** React with KO*t*Bu to Form Strong Organic Electron Donors

Further studies on BHAS reactions showed that they are assisted by any of a wide range of additives. The reactions generally proceed in high yield.^3–11^ Propagation occurs when aryl radicals **2** add to arene **4** to give **7**. Deprotonation affords radical anion **8**, which donates an electron to another molecule of **1**, thereby propagating the chain and forming the product, biphenyl **9**.^11^ We previously explored the role of the additives and showed that super electron donors **11**,^12–14^ formed *in situ* from additive **10**, can initiate these BHAS reactions.^15^ Additives other than **10** facilitate these reactions, e.g., **12**–**17**, and are converted *in situ* by KO*t*Bu into strong organic electron donors that initiate the chemistry^16–19^ (Scheme 1C). Thus, it was clear for BHAS reactions that the formation of strong electron donor molecules facilitated the reaction by converting the aryl halide substrates into aryl radicals through electron transfer.

But we also showed that a slower coupling of aryl halides to arenes can occur in the absence of additives, i.e., simply by the reaction of KO*t*Bu **18** with the aryl halide in the presence of excess arene.^15^ Although not discussed in detail, the literature represents the reaction between KO*t*Bu and an aryl halide as involving direct electron transfer from the alkoxide to the aryl halide, leading to a *tert*-butoxyl radical, an aryl radical, and a halide anion.^7–9,20–26^ However, alkali metal alkoxides are very poor electron donors. For example, for potassium *tert*-butoxide, *E*_ox_ = +0.10 V (vs. saturated calomel electrode (SCE) in dimethylformamide (DMF)),^27^ while for Ar–I, *E*_red_ ≈ −2.3 V (vs. SCE in DMF),^28^ making outer sphere electron transfer from KO*t*Bu to ArI impossible.^27^

Besides the problem of incompatible redox potentials, another problem with proposing electron transfer from KO*t*Bu as the route to the initiation of radical chemistry is the absence of evidence of methyl radicals in these reactions. If direct electron transfer from KO*t*Bu to aryl halides occurred, the resulting *tert-*butoxyl radicals **19** would partition between hydrogen atom abstraction to form *t*BuOH **23** and fragmentation to acetone **20** and methyl radicals **21** (Scheme 2A).^29–31^ No evidence has been published of methyl radicals arising in these reactions (methyl radicals, generated by metal-free methods from peroxides, are known to add to heteroarenes).^32–37^

**Scheme 2:**
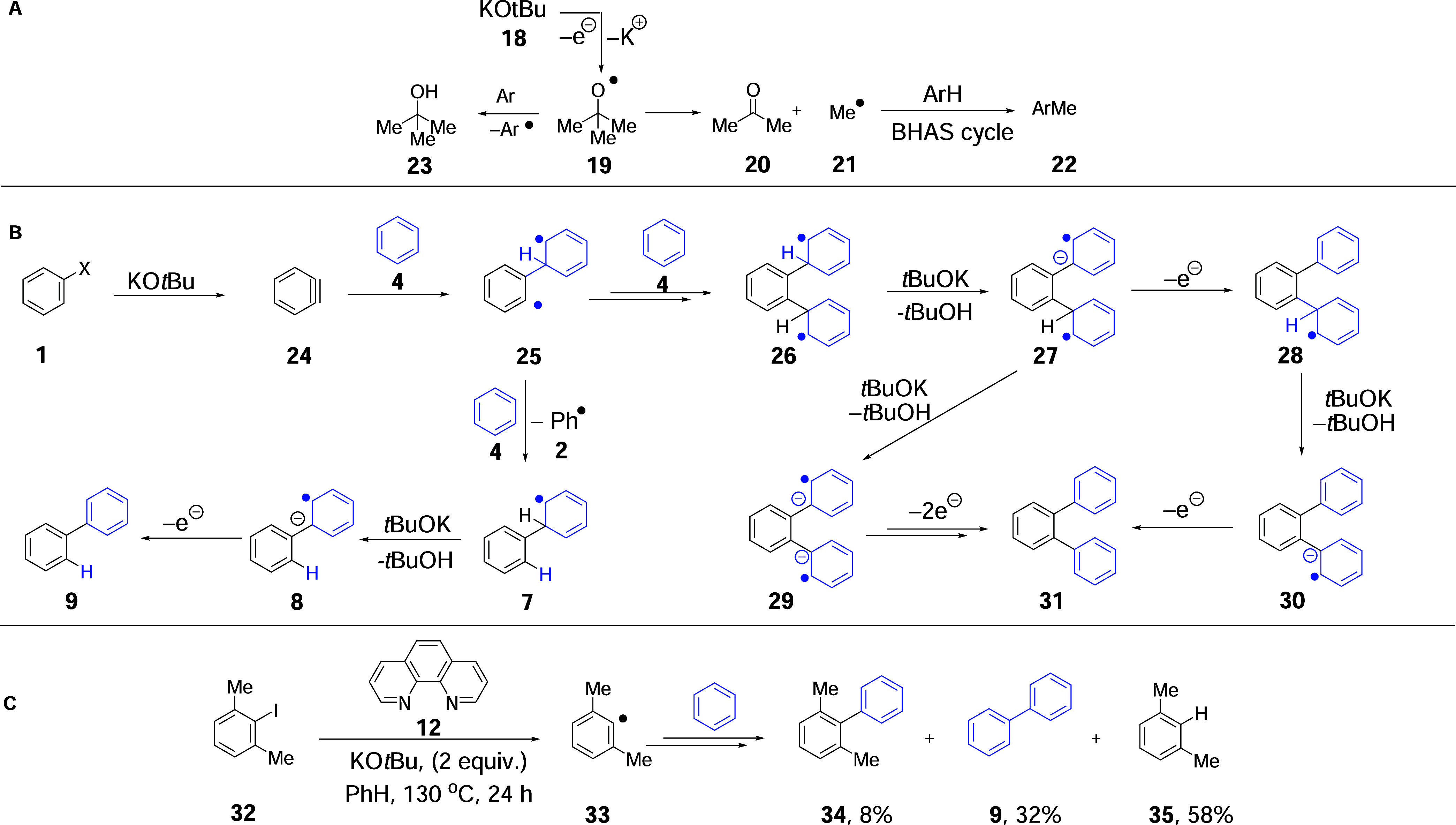
(A) The Known Fragmentation of *tert*-Butoxyl Radicals **19**; (B) Potential Initiation of BHAS Coupling through Formation of *o*-Benzyne **24**; and (C) 2,6-Dimethyliodobenzene **32** as a BHAS Substrate

In 2014, we tentatively proposed an alternative initiation of BHAS reactions in the absence of recognized electron donors, through benzyne intermediates (Scheme 2B).^15^ Benzynes are known to form when aryl halides are treated with a strong base, and addition of benzynes to alkenes has been proposed to go through diradical intermediates.^38–40^ In principle, a similar addition to arenes might provide a source of radicals to initiate BHAS chemistry.^15^ But disruption of the aromaticity of two benzene rings by a ground-state *o-*benzyne to form products like **31** is unknown and would be very surprising.^41^ In our proposal, the addition of *o*-benzyne **24** to benzene **4** forms diradical **25**. This is both a reactive aryl radical and a less reactive cyclohexadienyl radical. The aryl radical could either add to benzene to form **26** or abstract an H atom from benzene to form **7**. Deprotonations and electron transfers would convert **7** and **26** to **9** and **31**, respectively. The electron transfer steps would activate substrate **1** as in Scheme 1B to allow the propagation of the reaction. Byproduct **31** emerging from the proposed initiation pathway has never been reported. That tallies with propagation being much faster than initiation, leading to very low concentrations of such byproducts.

To test the proposal, we reported the outcome of experiments with 2,6-dimethyliodobenzene **32**, which cannot form an *o*-benzyne.^15,42^ This hindered substrate affords lower yields of products, but hindrance is key to its role as an indicator of mechanism.^43^ Using a suitable electron donor, formed from KO*t*Bu and phenanthroline^4,7,15^ (Scheme 2C), coupling to benzene occurred, affording **34** via radical **33**. The alternative outcome for **33** was that it abstracted an H atom from benzene yielding **35**, and the resulting phenyl radical was added to benzene to form biphenyl **9** by a BHAS mechanism. Seeing these products reinforced the importance of electron transfer initiation of the reaction.

When the experiment was repeated without an electron donor,^15^ i.e., in the presence of KO*t*Bu alone, we expected that the combined yield of coupled products, **34** + **9**, would be 0%. Although the combined yield dropped significantly from 42 to <1%, nevertheless, the fact that the coupled products **34** and **9** were still formed to any extent indicated that an additional mechanism for their formation was in play.

Returning to Scheme 2B, our motivation was to search for terphenyl byproduct **31** and to see if that might give evidence of the involvement of benzyne in the initiation process. If **31** could be identified, (i) it might simply arise through further BHAS reaction of phenyl radicals **2** with biphenyl **9**, or alternatively, (ii) it could arise from the reaction of benzyne **24** with two molecules of benzene solvent. The two routes might be distinguished by reacting deuterated iodobenzene, **1-*d*_5_**, with a base, in benzene as a solvent, and observing the products that are formed (Scheme 3). In this case, if labeled phenyl radicals **2-*d*_5_** are produced, then they should routinely lead to labeled biphenyl, **9-*d*_5_**, through reaction with benzene solvent; reaction of another radical **2-*d*_5_** with **9-*d*_5_** would yield equal amounts of terphenyls **31-*d*_9_** and **31-*d*_10_**. On the other hand, if the terphenyl arises from labeled benzyne **24-*d*_4_** reacting with two molecules of benzene solvent, this would afford **31-*d*_4_**. As will be seen below, deuterium isotope studies provide strong evidence in support of *o*-terphenyl formation from *o-*benzyne **24**, but they will also provide evidence of simultaneous formation of *m*- and *p*-benzynes from iodobenzene as a substrate. Further important information about the regiochemistry of benzyne formation will then be explored using isotopologues of 9-bromoanthracene and 9,10-dibromoanthracene. Our strategy also involves studying the outcomes of such reactions with the deuterated solvent C_6_D_6_ and deuterated base KO*t*Bu-*d*_9_ to provide further important mechanistic information.

**Scheme 3:**
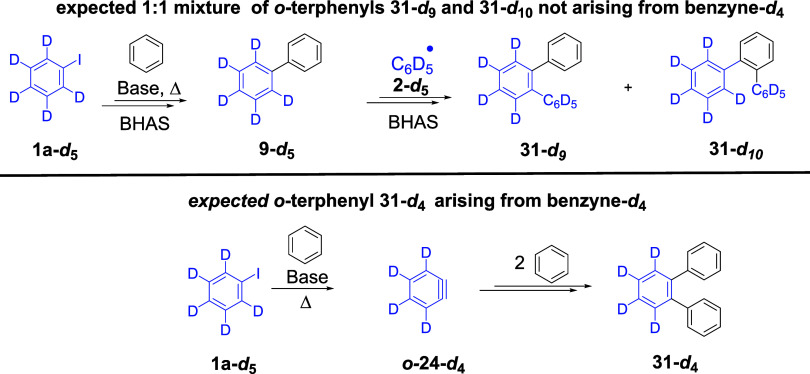
Using **1a-** ***d***_**5**_ to Distinguish between Routes to *o*-Terphenyl **31**

## Results and Discussion

### Evidence of Benzynes

As mentioned in the Introduction, our study began with a search for terphenyl product **31** in the reaction of iodobenzene **1a** (X = I), with KO*t*Bu in benzene as a solvent. This reaction afforded *tert*-butoxybenzene, **36** (16.1%), from attack by butoxide on benzyne and traces of triphenylene **37** from the trimerization of benzyne^44^ (Scheme 4a). In addition, biphenyl **9** (27.2%) from the propagation steps in Scheme 1B starting material, and iodobenzene **1a**, X = I (36.7%), were quantified by gas chromatography-flame ionization detection (GCFID) or NMR. Three terphenyls, **31** (0.18%), **38** (0.08%), and **39** (0.05%), were also identified by GC mass spectrometry (GCMS) (see SI, pp S6–S10), quantified by GCFID, and correlated with authentic samples of *o-*, *m*-, and *p*-terphenyl (Scheme 4A).

**Scheme 4:**
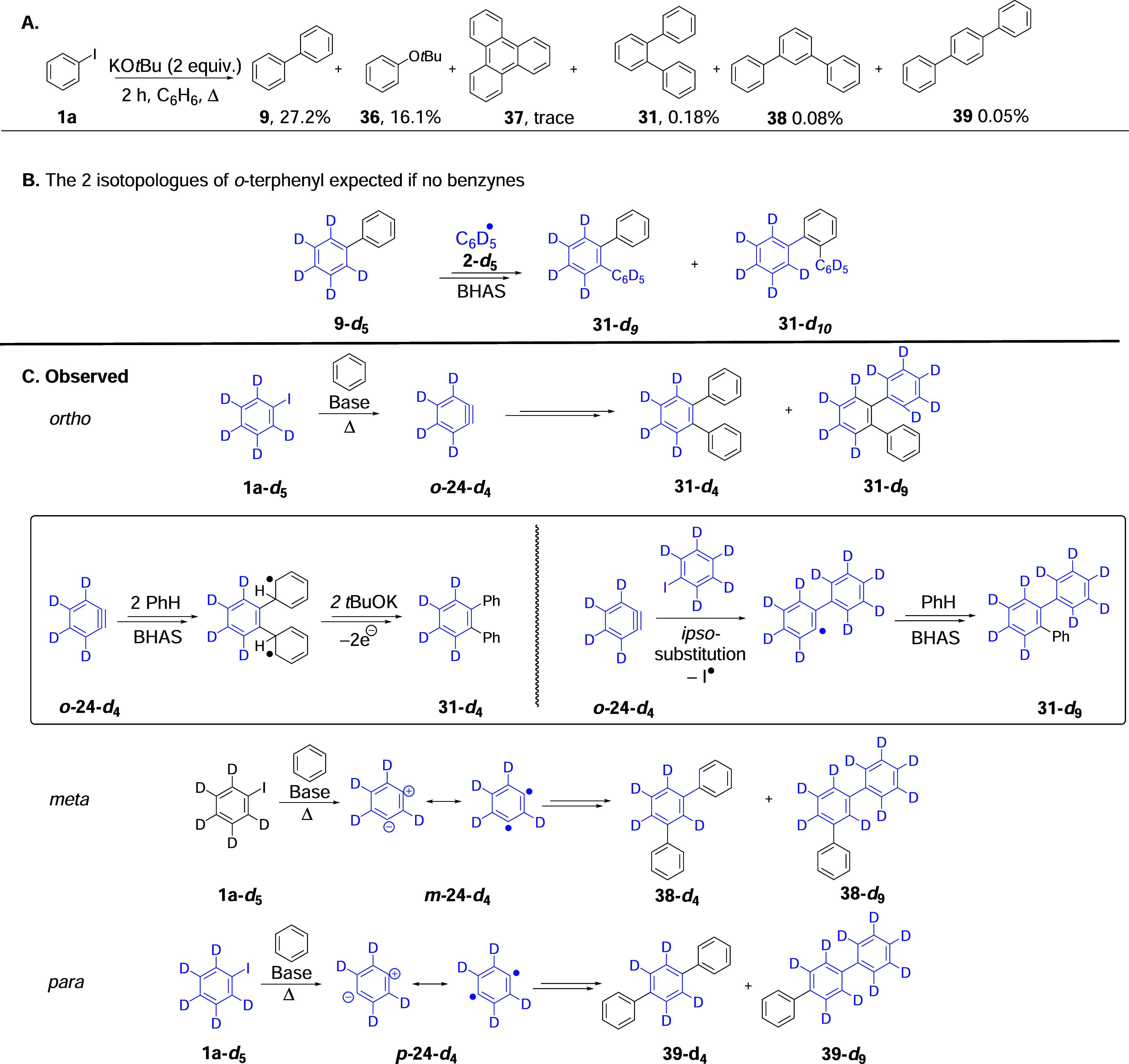
(A) Products from the Reaction of PhI with KO*t*Bu in PhH; (B) From Reaction of C_6_D_5_I with KO*t*Bu in PhH, Terphenyls Might Arise from Attack of Phenyl Radicals **2-*d*_5_** on Biphenyl Product **9-** ***d***_**5**_;^a^ and (C) The Observed *o*-, *m*-, and *p*-Terphenyl Products and How They Arise from *o*-, *m*-, and *p*-Benzynes

a Only the *o*-terphenyl products expected **31-*d*_9_** and **31-*d*_10_** are shown here.

These terphenyls might simply have arisen through the BHAS reaction of the standard propagation product of the reaction, biphenyl **9**, with further phenyl radicals in solution. However, this was disproved when the experiment was repeated with iodobenzene-*d*_5_, where the deuteration patterns of the products revealed important information.

In this reaction, phenyl radicals **2-*d*_5_** might react with the biphenyl formed in this reaction, i.e., **9-*d*_5_**; *o*-, *m*-, and *p*-terphenyls could form in this way. Scheme 4B focuses only on the *o-*terphenyl products and shows the two isotopologues that would arise. The radical attack of **2-*d*_5_** can occur on either ring of the biphenyl, ultimately giving a ∼1:1 mixture of *o*-terphenyls, **31-*d*_9_** and **31-*d*_10_**. Likewise, ***d*_9_** and ***d*_10_** isotopologues would be seen for the *m*- and *p*-terphenyls. However, the observed labeling patterns were quite different; ***d*_9_** isotopologues were seen, but ***d*_10_** isotopologues were not seen. The other isotopologues seen were ***d*_4_** isotopologues. While both these ***d*_4_** and ***d*_9_** isotopologues were easily seen for the *ortho*- and *para*-isomers, **31** and **39**, the *meta*-case formed almost exclusively **38-*d*_9_** (see SI, pp S14 and S15). Scheme 4C shows how they arise from benzyne intermediates.

First, we focus on *o*-benzyne, *o-* **24-*d*_4_**. This led to the terphenyls **31-** ***d***_**4**_ and **31-** ***d***_**9**_. As shown in the inset, **31-** ***d***_**4**_ would arise from sequential BHAS reactions with two benzene molecules. The route to **31-** ***d***_**9**_, on the other hand, would involve an *ipso*-substitution^45,46^ reaction on C_6_D_5_I, together with a BHAS reaction with PhH. Similar outcomes for the *m*- and *p*-terphenyls would fit with the proposal. Our experiment therefore surprisingly establishes that the terphenyl products formed in this reaction arise because *o*-, *m*-, and *p*-benzynes are being formed in solution. (*m*- and *p*-Benzynes may alternatively be referred to as “arenediyls” or as “didehydroarenes”, but we will use the benzyne terminology in this paper.)

These reactions also show the following:

(i) *m-* and *p*-benzynes are formed through base-treatment of a simple halobenzene (until now, only *o*-benzynes were known to form in this way).^47^ Perrin et al. have recently pioneered the nonradical chemistry of *p*-benzynes and widely investigated the reverse reaction, i.e., addition of halide anion to *p*-benzynes.^48,49^

(ii) Addition of a ground state *o*-benzyne to an arene, benzene, does indeed lead to products derived from diradical intermediates.^50,51^ Ground state *o-*benzynes do not routinely show diradical behavior with closed shell molecules, but recent results showed that they can be attacked by persistent radicals like TEMPO.^52,53^

### Haloanthracene Substrates

Having observed that the deprotonation of iodobenzene could occur at *ortho*, *meta*, and *para*-sites to lead to *o*-, *m*-, and *p*-benzynes, we were keen to explore the regiochemistry of deprotonation of other substrates, namely, 9-haloanthracenes **40**, **40** ^**Cl**^, and **40** ^**I**^ that cannot form *o*-benzynes (Scheme 5A). 9-Iodoanthracene **40** ^**I**^ and 9-bromoanthracene **40** reacted most efficiently, while 9-chloroanthracene **40** ^**Cl**^ was less reactive (see SI, pp S59–S68).^54^ For these substrates, while *o*- and *m*-benzynes cannot form, a *p-*benzyne could be possible. Focusing on the reaction with 9-bromoanthracene, this afforded anthracenes **41**–**44**, as well as biphenyl **9**, as detailed below. Notably, methylated compounds **45**–**48** were also identified (Scheme 5A and see SI, p. S25).

**Scheme 5:**
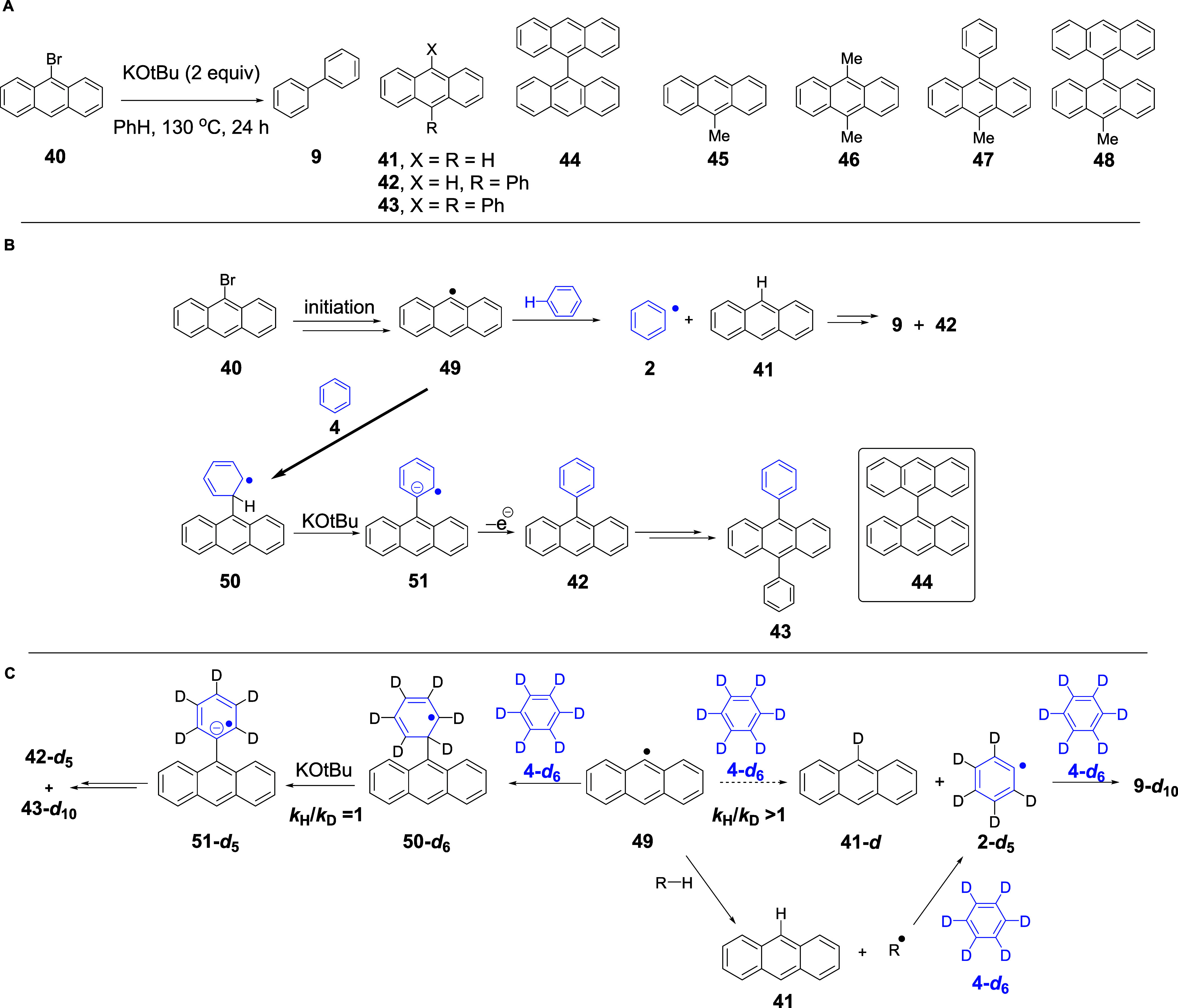
(A) Reaction of 9-Bromoanthracene, **40**; (B) Formation of the Non-methylated Products from 9-Bromoanthracene **40**; and (C) Expected Reactions of Radical **49** with C_6_D_6_ ^a^

a In the actual experiment, the product anthracene **41** was unlabeled.

The methylated products will be discussed below, while Scheme 5B illustrates the formation of the other products. 9-Anthracenyl radical **49** could (a) abstract an H atom from benzene to afford anthracene **41** and a phenyl radical **2** or (b) add to benzene **4** (shown) or to anthracene **41** (not shown) by the BHAS mechanism or to bromoanthracene **40** by *ipso*-substitution of the bromine atom (not shown) to afford 9-phenylanthracene **42** and 9,9′-bianthracene **44** (inset in Scheme 5B), respectively. In turn, further BHAS reaction of phenylanthracene **42** with phenyl radical **2** would form diphenylanthracene **43**, while **2** and **41** undergo further reactions to form products **9** and **42**.

### Isotope Effects with 9-Bromoanthracene **40**

The above reaction of **40** in C_6_H_6_ was repeated under identical conditions in a side-by-side reaction in C_6_D_6_ as a solvent. First, we describe what was expected when the anthracenyl radical **49** reacts with C_6_D_6_ in relation to isotope effects. Two possibilities exist:

(i) The radical adds to C_6_D_6_ to form arylcyclohexadienyl radical **50-** ***d***_**6**_ and this undergoes loss of D^+^ to afford **51-** ***d***_**5**_ en route to products (Scheme 5C). Deprotonations of cyclohexadienyl radicals (like **50**) to form cyclohexadienyl radical anions in BHAS chemistry are known not to be rate-determining steps.^7,23,55^

(ii) The radical abstracts a D atom from C_6_D_6_. This would form **41-** ***d*** together with a C_6_D_5_ radical, **2-** ***d***_**5**_. This breaking of an Ar–D bond is a kinetically challenging step in BHAS chemistry and would give rise to a primary deuterium isotope effect.^43^

The results are shown in Table 1, entries 1 and 2. Similar products were formed in both reactions, but the labeled products that formed in C_6_D_6_ were **9-** ***d***_**10**_, **42-** ***d***_**5**_, and **43-** ***d***_**10**_. Unlabeled anthracene **41** was formed in that reaction rather than **41-** ***d***. This implies that **49** abstracts an H atom from a source, R–H, other than deuterobenzene **4-** ***d***_**6**_, and that the resulting radical is responsible for the subsequent formation of the phenyl radical, **2-** ***d***_**5**_. The nature of R–H is discussed later in this paper.

**Table 1:**
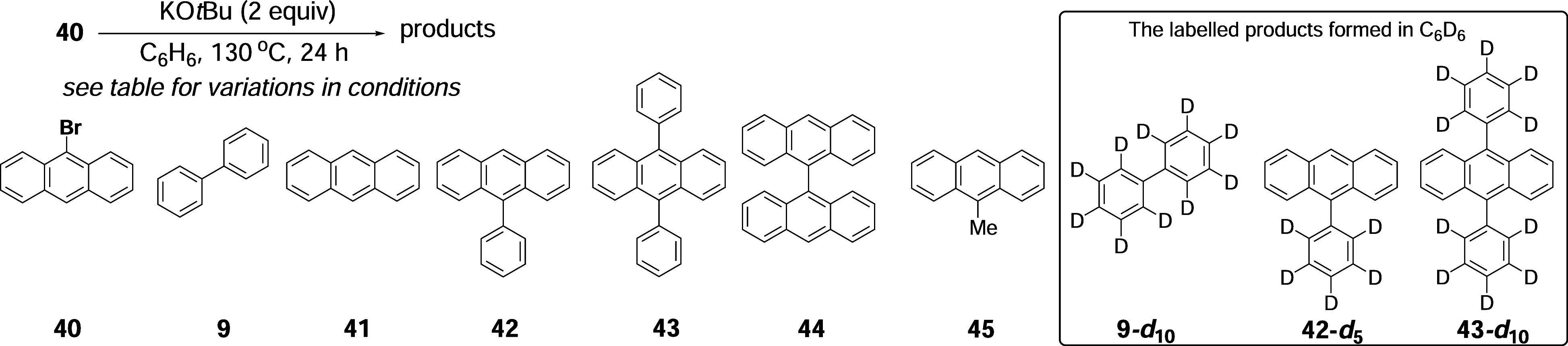
Reactions of **40** Using Isotopologues of the Solvent and KO*t*Bu

entry	conditions	**40**	**9** or **9-*d*_10_** ^b^ (%)	**41** (%)	**42** or **42-*d*_5_** ^b^ (%)	**43** or **43-*d*_10_** ^b^ (%)	**44** (%)	**45** or **45-*d*_*n*_** (%)
1^a^	KO*t*Bu, C_6_H_6_	0	28.2	39.2	20.4	1.4	5.8	3.2
2^a^	KO*t*Bu, C_6_D_6_	0	5.2^b^	20.4	20.1^b^	0.4^b^	6.8	6.2
3	KO*t*Bu-*d*_9_, C_6_H_6_	0	26.8	29.0	18.9	1.5	4.7	0.7^b^
4	KO*t*Bu-*d*_9_, C_6_D_6_	0	5.0^b^	8.3	18.1^b^	0.4^b^	3.8	2.5^b^

a Reactions were conducted in triplicate (entry 1) and in duplicate (entry 2).

b Indicates deuterated product.

The amount of biphenyl **9-** ***d***_**10**_ produced from the C_6_D_6_ reaction was greatly decreased compared to that of the corresponding compound in C_6_H_6_ (28.2 → 5.2%), reflecting that the formation of biphenyl **9** was occurring through the expected BHAS process. The amount of phenylanthracene **42-** ***d***_**5**_ produced remained constant relative to its isotopologue in the C_6_H_6_ reaction (20.1 → 20.4%), which would be expected, as no kinetic isotope effect (KIE) should be associated with the deprotonation of radical **50**. The same applies to the formation of bianthracene **44** (entries 3 and 4 of Table 1 will be discussed later).

Further information was gathered by preparing a range of selectively deuterated isotopologues of **40**, namely, **40-** ***d***_**1**_, **40-** ***d***_**8**_, and **40-** ***d***_**9**_, and comparing their reactivity with that of undeuterated **40**. In these experiments, the duration of the reaction is shorter (30 min) to ensure that none of the substrates is fully converted into product and hence that the differences in their reactivity are evident. The substrates were tested under identical conditions in side-by-side reactions (***d***_**0**_, ***d***_**1**_, ***d***_**8**_, ***d***_**9**_) (Table 2).

**Table 2:**
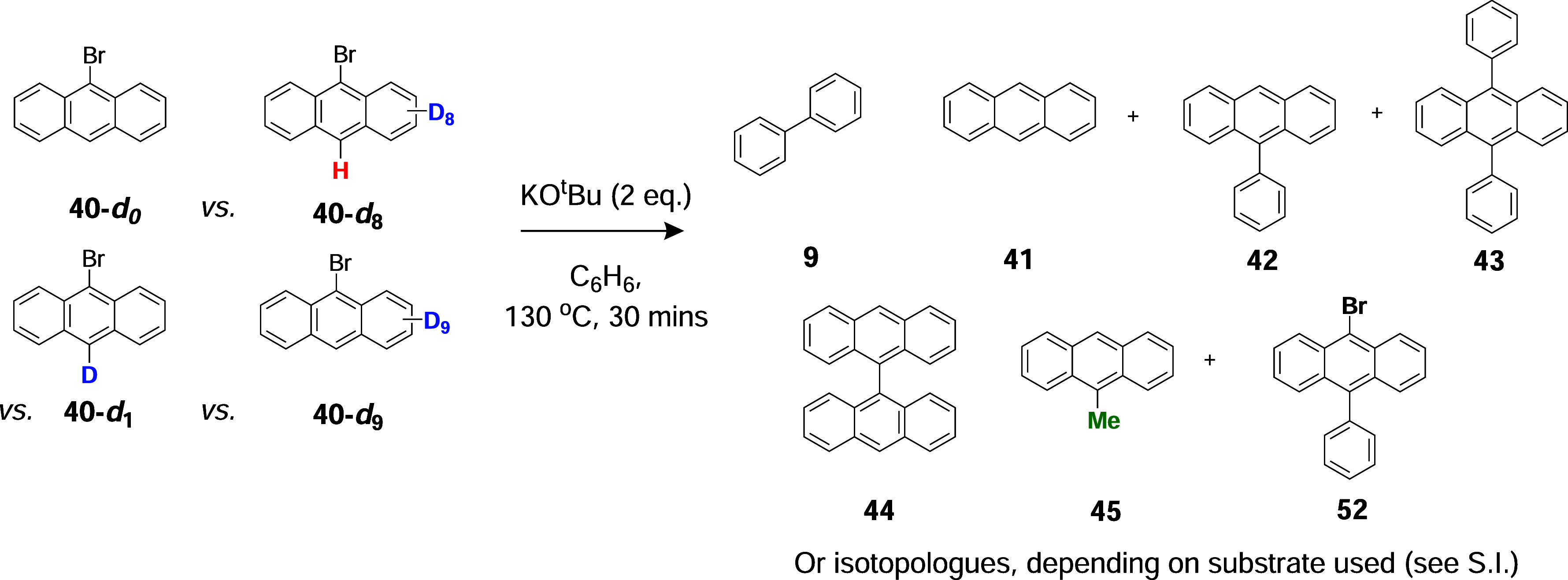
Reactions of Isotopologues of **40** with C_6_H_6_ and KO*t*Bu

		% yield^a^ ^,^ ^b^							
entry	substrate	**40**	**9**	**41**	**42**	**43**	**44**	**45**	**52**
1	**40-** ***d***_**0**_	45.1	12.4	17.6	7.1	0.5	2.0	1.7	0.8
2	**40-** ***d***_**1**_	65.2	5.7	8.7	3.3	0.2	1.1	0.9	0.5
3	**40-** ***d***_**8**_	75.7	2.2	3.5	1.4	0.1	0.5	0.4	0.2
4	**40-** ***d***_**9**_	81.0	1.8	2.8	1.1	0.1	0.3	0.3	0.2

a Isotopologue yields determined using GCFID calibrations for nondeuterated compounds.

b Reactions were performed in triplicate; averaged values are presented in this table.

The results show that an increasing level of deuteration in **40** slows the reaction. The amount of the particular isotopologue of **9** (or of anthracene **41**) formed in each experiment represents the radical flux. Comparing experiments in Table 2 (entries 1 and 2) shows that the yield of **9** from **40-** ***d***_**1**_ (5.7%) is significantly lower than that from **40-** ***d***_**0**_ (12.4%). This shows that the amount of phenyl radical being produced in these reactions is dependent on the C–H/C–D bond in the 10-position of the anthracene. This is not at all consistent with the radical chemistry being driven by single electron transfer (SET) from KO*t*Bu **18** (Scheme 2A). Instead, it suggests that removal by KO*t*Bu of H^+^/D^+^ from this C–H/C–D bond controls the level of initiation of radical chemistry. This makes sense if this step is followed by the loss of a bromide anion affording a *p*-benzyne. More surprising information emerges from the reactions of the ***d***_**8**_ and ***d***_**9**_ isotopologues (entries 3 and 4), which have progressively stronger inhibitory effects. This shows that breaking the C–H at sites other than at position 10 also contributes to the flux of radical chemistry. This suggests that the site of deprotonation and loss of halide do not need to be on the same ring, i.e., that “remote benzynes” (“distal diradicals” resulting from loss of HBr from substrates; we suggest that distal diradicals is the better term) can also trigger these reactions.

### Explaining the Methylated Products

Now returning to consider the methylated products from Scheme 5A, the structure of methylanthracene **45** was confirmed by ^1^H NMR and by high-resolution MS (HRMS), and its retention time on GCMS was confirmed by comparison with that of authentic 9-methylanthracene. The positions of the methyl groups in the other methylated products **46**–**48** have not been determined but are proposed by analogy to **45**. These compounds arise through the BHAS reaction of methyl radicals **21** with the anthracenes present in the reaction mixture. Methyl radicals might arise through fragmentation of *tert-*butoxyl radicals **19**, which, in turn, might form following electron transfer from KO*t*Bu **18** (Scheme 2A), and so SET from KO*t*Bu seemed at least a possible explanation.

Comparing the reactions conducted in C_6_D_6_ versus C_6_H_6_ (Table 1), the amount of 9-methylanthracene **45** formed in C_6_H_6_ was much lower compared to the reaction in C_6_D_6_ (3.2 vs 6.3%). If *tert-*butoxyl radicals **19** were present and were fragmenting as in Scheme 2A, this could explain the data. In C_6_H_6_, the methyl radicals would (i) add to anthracenes or (ii) abstract an H from the solvent in a defined ratio. In C_6_D_6_, it would be more difficult to abstract a D atom, resulting in a higher percentage of addition to anthracenes. However, please note that an alternative rationale for this chemistry is provided below.

As mentioned above, the anthracene **41** generated from the reaction of 9-bromoanthracene **40** with KO*t*Bu in C_6_D_6_ was unlabeled, suggesting KO*t*Bu as the most likely source of the abstracted H atom. To investigate this, KO*t*Bu-***d***_9_ was used in a repeat experiment (Table 1, entry 3; spectra pp S38–S40). Treatment of 9-bromoanthracene **40** with KO*t*Bu-*d*_9_ in C_6_H_6_ still led to the formation of unlabeled anthracene **41**, suggesting that, in this case, the hydrogen atom transfer (HAT) is solely or predominantly occurring from the solvent. That would mean that the selectivity for abstraction of an H versus a D atom was good, with KO*t*Bu being used as a source of HAT when the solvent is C_6_D_6_, and C_6_H_6_ being used when KO*t*Bu-*d*_9_ is the base. In view of this, we reacted **40** with a combination of C_6_D_6_ and KO*t*Bu-*d*_9_, which yielded predominantly anthracene **41-** ***d***_**1**_ along with undeuterated anthracene (Table 1, entry 4; spectra pp S41–S43). The undeuterated anthracene component shows that intermolecular hydrogen abstraction from anthracene C–H bonds competed with the abstraction of C–D from KO*t*Bu-*d*_9_ and C_6_D_6_. In this case, when faced with both deuterated reagents, then labeling did indeed occur; the yield of the anthracene was significantly decreased compared to the parent reaction (entry 1) with KO*t*Bu in C_6_H_6_ (39.2 → 8.3%).

The information that KO*t*Bu is used for hydrogen atom transfer (HAT) by the aryl radicals is very important and it raises the question of what happens to the product radicals **54** (Scheme 6A). The answer to that comes from comparing entries 1 and 3 or 2 and 4 in Table 1. Comparing entries 1 and 3, we see that using C_6_H_6_ as the solvent and changing from KO*t*Bu to KO*t*Bu-*d*_9_ decrease the amount of methylanthracene isotopologue **45** 4-fold (3.2 → 0.7%). This results from a 4-fold decrease in the flux of methyl radicals. This number represents a primary kinetic isotope effect associated with C–H/C–D bond cleavage in KO*t*Bu in the rate-determining step (Scheme 6A). That is completely inconsistent with the picture shown in Scheme 2A. There, the reactions would simply involve electron transfer from KO*t*Bu or KO*t*Bu-*d*_9_ to substrate **40**. This could involve a minor quantitative change but far smaller than what is seen here. Similarly comparing entries 2 and 4 in Table 1 shows that a primary deuterium isotope effect is evident. A primary deuterium isotope effect would align with HAT from KO*t*Bu by diradicals **53** (or aryl radicals) and fragmentation of the resulting alkyl radical **54** to afford methyl radicals **21** and the potassium enolate of acetone, **55**. Computational studies show that both the hydrogen atom abstraction from potassium *tert-*butoxide and the fragmentation of the resulting radical are easily achieved under the reaction conditions (see pp. S144–S146). That therefore means that electron transfer from KO*t*Bu was NOT driving the reaction, but rather that formation of anthracenyl radicals **49** controls the formation of methyl radicals **21**, in line with our other evidence for the role of benzynes.

**Scheme 6:**
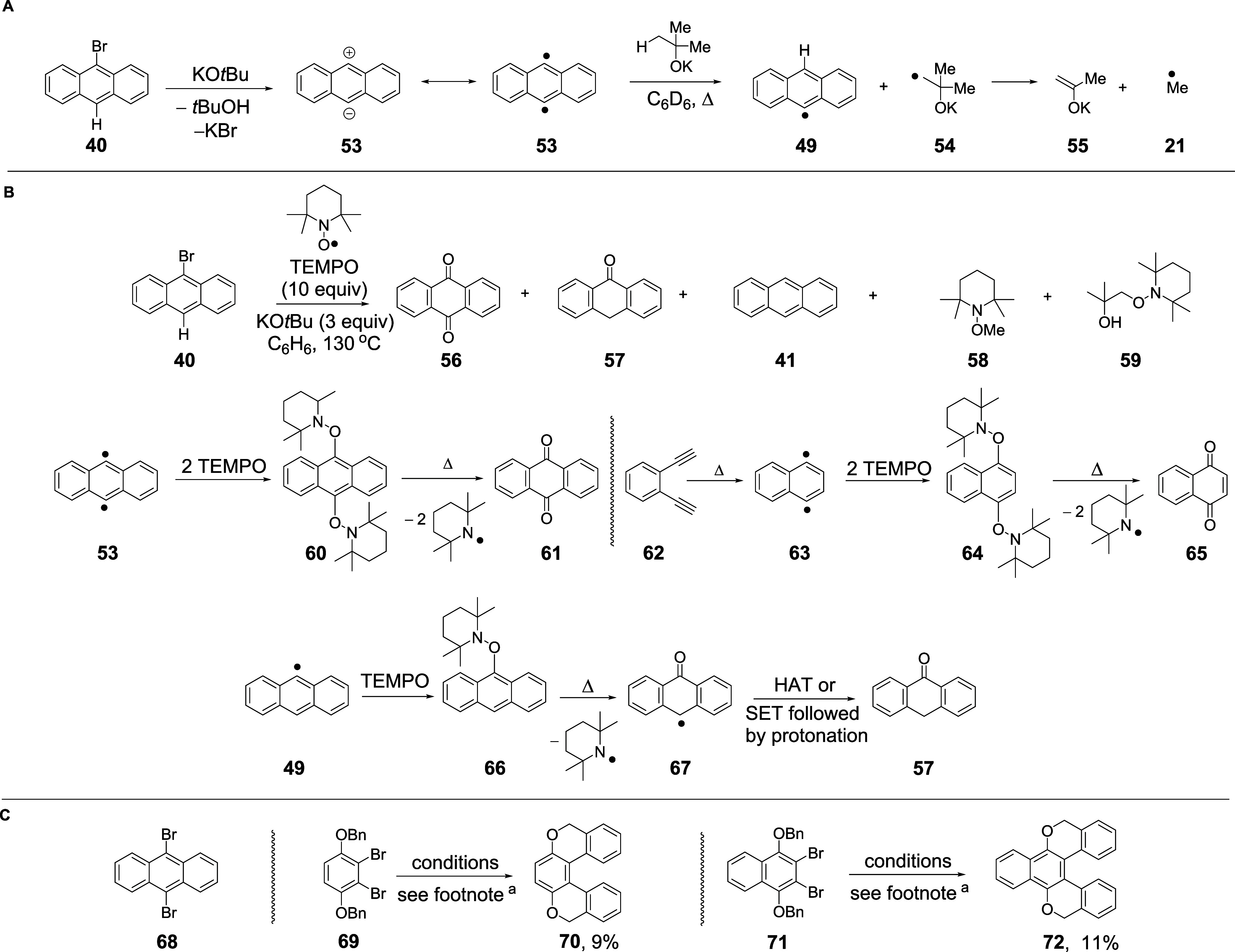
(A) A Role for KO*t*Bu in Hydrogen Atom Transfer (HAT) Reactions; (B) TEMPO Studies; and (C) Double BHAS Reactions on Substrates **68**, **69**, and **71** ^a^

a Conditions: KO*t*Bu (2 equiv), PhH, 130 °C, 24 h.

Turning now to results in Table 2, the yield of 9-methylanthracene **45** also decreases with an increasing level of deuteration of the bromoanthracene substrate. Again, this is completely inconsistent with the electron transfer from KO*t*Bu. If that were controlling the initiation, then no notable change in the level of initiation would be expected with the different isotopologues. Instead, the level of radical flux is controlled by the speed of the removal of HBr/DBr from the substrate by KO*t*Bu.

### TEMPO Studies

Given the role of radicals in these reactions, we next used TEMPO to probe the intermediates (Scheme 6B). When bromoanthracene **40** was heated with KO*t*Bu in benzene in the presence of TEMPO, this led to products including anthraquinone **56** and anthrone **57** in addition to TEMPO-trapped products **58** and **59**. Anthraquinone arises by thermolysis of bis-TEMPO intermediate **60**, which, in turn, comes from trapping of the *p*-benzyne **53** from the initiation of the reaction. This has clear direct analogy in the literature when Bergman cyclization of *o*-benzenediyne **62** led to formation of naphthalen-1,4-diyl **63**; the initial bis-TEMPO-trapped intermediate **64** thermolyzed *in situ* to afford *p*-naphthoquinone **65**.^56^ The anthrone **57** formed in our experiment would similarly form from thermolysis of **66**, which in turn arises from trapping anthracenyl radical **49**.

### Studies with Different Bases

Given the outcome of the experiments of 9-bromoanthracene with KO*t*Bu in C_6_H_6_, we also explored a number of variants that are detailed in the SI (pp S45–S59). The reaction worked well when KOEt was used, establishing that the reaction was not confined to KO*t*Bu as the base (see SI). This was important because it showed that nontertiary alkoxides promote this reaction. Unlike *tert*-butoxide, ethoxide contains weak H–CO bonds that can quench aryl radicals, and so, in this case, quenching of anthracenyl radicals to form anthracene, a hydrodehalogenation reaction, was more prevalent than in reactions with KO*t*Bu. The hydrodehalogenation reactions reviewed by Bunnett^2^ invariably used primary or secondary alkoxides and this therefore links the mechanism for radical formation in those reactions to our observations on BHAS reactions here.

When KOCEt_3_ was used instead of KO*t*Bu, the reaction was again successful. As expected, KOCEt_3_ afforded traces of ethylated products rather than the methylated products seen with KO*t*Bu. No reaction was seen with NaO*t*Bu or LiO*t*Bu, but this may have been due to the low solubility in benzene. Adding 15-crown-5 to the NaO*t*Bu experiment allowed conversion to the expected products, including the BHAS products. 15-Crown-5 is an excellent H atom donor, and so anthracene **41** was the major product in this case (see pp S50–S52). A control reaction using KO*t*Bu (but carried out while in darkness (under foil)) also afforded the products seen under normal daylight conditions, so the reaction was not light-dependent.

### Dihalo-Substrates

Having studied the reactivities of monohalo substrates, we then explored the reactivity of three dibromo substrates **68**, **69**, and **71** (Scheme 6C). 9,10-Dibromoanthracene **68** was of interest because benzyne-type activation of this substrate specifically could only be achieved by deprotonation at a site remote from the C–Br bonds (i.e., not *o*-, *m*-, or *p*- to these bonds). Here, we compared the reaction of the parent to the reaction of its ***d***_**8**_-isotopologue (Table 3). Side-by-side reactions (15 min) of the two isotopologues were conducted under identical conditions. The yield of **9** dropped from 19.1% in the parent substrate to 2.8% in the deuterated substrate. This provides strong backing to the generality of the benzyne activation in this case, where no *o*-, *m*-, or *p*-benzyne can form. In this case, deprotonation at the periphery must lead to an “*r*-benzyne” (or “*r*-diyl”).

**Table 3:**
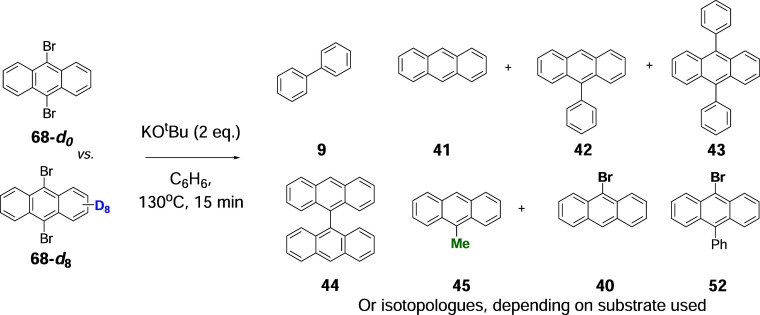
Reaction of 9,10-Dibromoanthracene

		% yield^a^								
entry	substrate	**68**	**9**	**40**	**41**	**42**	**43**	**44**	**45**	**52**
1	**68-** ***d***_**0**_	41.0	19.1	22.1	0.7	2.4	0.4	3.7	0.4	3.7
2	**68-** ***d***_**8**_	85.1	2.8	4.1	0.0	0.3	0.0	0.0	0.0	4.1

a Isotopologue yields determined using GCFID calibrations for nondeuterated compounds.

The remaining substrates that we discuss here are vicinal dibromides **69** and **71**. These substrates were interesting as *o-*dibromides that offered opportunities for intramolecular BHAS reactions. In **69**, deprotonation can occur at *m*- and *p*- to the bromides, while in **71**, deprotonation at a remote ring site is needed. Comparing the outcomes allows for an assessment of any penalty in **71** for requiring deprotonation in a remote ring. The complex multistep reactions lead to similar yields, suggesting that the need for remote deprotonation does not necessarily impede the reaction.

## Conclusions

The route by which base-treatment of haloarenes affords aryl radicals, which has puzzled chemists since 1899, is now revealed.

(i) Deuterium labeling establishes that treatment of iodobenzene with KO*t*Bu in benzene affords simultaneously *o*-, *m*-, *p*-benzynes through deprotonation and loss of halide anion but, starting from C_6_D_5_I, the formation of specifically deuterated *o*-, *m*-, and *p*-terphenyl products shows that each type of benzyne is able to trap two benzenes regiospecifically through radical reactions and thereby they act as initiators of radical chain chemistry. As initiators, they only need to be present in minute quantities.
(ii) A study of specifically deuterated haloanthracenes shows that base-induced removal of H^+^/D^+^ does not need to occur in the ring that bears the halogen substituent but can happen in a neighboring ring, thereby giving rise to distal diradicals that are effectively “*r*-benzynes” (remote benzynes).
(iii) When KO*t*Bu is used as a base, low levels of methylated arenes are also formed that arise from methyl radicals. These are generated when aryl radicals or diradicals abstract an H atom from a C–H bond in KO*t*Bu and the resulting carbon radical undergoes a previously unreported fragmentation.

The results are completely at odds with suggestions that KO*t*Bu acts as an electron donor to haloarenes. They rationalize the initiation pathway for the protodehalogenations observed since 1899 as reviewed by Bunnett.^2^

### Wider Applications?

Questions arise about how widespread our observations may prove to be. Clearly, any case in which alkoxides encounter aryl halides could lead to benzynes that trigger further reactions. Some colleagues report on preferences for sodium alkoxides over potassium alkoxides as bases in synthetic transformations such as Buchwald–Hartwig aminations. It may be that potassium alkoxides are more effective bases on aryl halides and promote byproducts to a greater extent, but this would require detailed analysis. The role of alkoxides in deprotonating arenes is not necessarily confined to aryl halides but can also be manifested in other arenes that may be subject to deprotonation.

One case in which KO*t*Bu was suggested to act as an electron donor to a surprising substrate was reported by Lei and Jutand. They showed that the reaction of KO*t*Bu *E*_ox_ = +0.10 V (vs. SCE in DMF)^27^ with phenanthroline *E*_red_ ≈ −2.06 V (vs. SCE in DMF) afforded radical anions derived from phenanthroline. They proposed that electron transfer from KO*t*Bu was taking place despite the large gap in potentials. We proposed an alternative explanation, where deprotonation of phenanthroline by KO*t*Bu led to an electron-rich dimeric product and, in turn, to formation of radical anion organic electron donors.^15,57^ The take-home message is a reassuring one, that KO*t*Bu is a notably poor electron donor in its ground state^58–60^ and that its radical-forming properties derive from its ability to deprotonate aryl halides and to convert them to benzynes/arenediyls, or to deprotonate other arenes.

## Supplementary Material



## Notes

Experimental procedures and spectroscopic data in support of the results are reported; X-ray data for compound **69** are available (Database ref number is CCDC 2211274) Computational data underpinning this study can be downloaded from the ioChem-BD database^61,62^ at the following URL: https://doi.org/10.19061/iochem-bd-6-628 (PDF)
