# Supplementary material for: How Alkali Metal Alkoxides Initiate Organic Radical Reactions
Source: J Am Chem Soc. 2026 Feb 20;148(8):8970–81. doi: 10.1021/jacs.5c22122 (PMC12964405; doi:10.1021/jacs.5c22122)
Supplement: Supplementary file 1 [file ja5c22122_si_001.pdf]

Supplementary information

How Alkali Metal Alkoxides Initiate Organic Radical Reactions

Seb Tyerman,[1‡] Kenneth F. Clark,[1‡] Alexander J. Stewart,[1] Krystian Kolodziejczak,[1] Craig M. Robertson,[2] Laura Evans[3], Alan R. Kennedy,[1] Tell Tuttle,[1] David J. Nelson,[1*] John A. Murphy.[1*]

[1]Department of Pure and Applied Chemistry, University of Strathclyde, 295 Cathedral Street, Glasgow G1 1XL, U.K.;
[2]GSK Medicines Research Centre, Gunnels Wood Road, Stevenage, Herts SG1 2NY, United Kingdom;
[3]AstraZeneca, Oncology Targeted Discovery, Oncology R&D, The Discovery Centre, Cambridge Biomedical Campus, 1 Francis Crick Avenue, Cambridge, CB2 0AA, United Kingdom.

Contents

General procedures **S2**

Blank GC-MS spectrum **S4**

Reactions of iodobenzene **S5**

- Reactions of iodobenzene isotopologues with KO*t*Bu in C_6H_6 .... S5
- GC-MS spectra of authentic samples of trace products S18
- Reactions of iodobenzene with KO*t*Bu in C_6D_6 .... S19

Reactions of 9-haloanthracene substrates **S25**

- Reactions of 9-bromoanthracene with KO*t*Bu **or** KO*t*Bu-d_9 in C_6H_6 **or** C_6D_6 .... S25
- Reaction of 9-bromoanthracene in the dark S43
- Reactions of 9-bromoanthracene with alternative bases or no base S45
- Reactions of other 9-haloanthracenes S59
- Side-by-side reactions of 9-bromoanthracene isotopologues S68
- Reactions of 9-bromoanthracene KO*t*Bu in C_6H_6 with TEMPO S87
- Methylanthracene isomer determination S94

Reactions of dibromoarene substrates **S96**

- Side-by-side reactions of 9,10-dibromoanthracene isotopologues S96
- Reactions of compound **69** with KO*t*Bu in C_6H_6 .... S98
- Reactions of compound **71** with KO*t*Bu in C_6H_6 .... S100

Substrate synthesis **S101**

NMR spectra **S108**

nOe study **S127**

GC-FID calibrations **S129**

X-ray crystal structure of 69 .... **S137**

DFT Calculations **S142**

References **S*1**

General Procedures

All reagents and solvents were obtained from commercial suppliers and were used without further purification unless mentioned otherwise. For short-timescale reactions, commercial haloarenes (9-bromoanthracene, 9-chloroanthracene, 9-iodoanthracene and 9,10-dibromoanthracene) were purified by column chromatography and/or recrystallization prior to use. Anthracene-d_{10} was purchased from Sigma Aldrich (98%D). Where used, diethyl ether, THF and dichloromethane (DCM) were dried using a Pure-Solv 400 solvent purification system (Innovative Technology Inc., USA). Anhydrous benzene (C_6H_6) was obtained from Sigma Aldrich, while benzene-d_6 99.5%D (C_6D_6) was obtained from Eurisotop. Where stated, reactions were prepared in a glovebox supplied by Innovative Technology Inc., USA, operated with a nitrogen atmosphere. For mechanistic reactions, new microwave vials and Teflon stirbars were used in the first instance, after which between each use they were soaked overnight in a NaOH base bath, and then rinsed thoroughly with aqueous HCl, water and acetone before drying overnight in an oven set to 150 °C.

Thin Layer Chromatography was performed on silica gel pre-coated aluminium plates (60 Å, F254 UV indicator) purchased from Merck. The thin layer chromatograms were analysed by UV (254 nm, UVP mineralight UVG-11 lamp) and staining either with basic $KMnO_4$ [$KMnO_4$ (6 g), K_2CO_3 (40 g), NaOH (5 mL, 10% w/w) in water (600 mL)] or an ethanolic solution of phosphomolybdic acid [phosphomolybdic acid hydrate (10 g) in ethanol (100 mL)]. Column Chromatography purification was performed with 35-70 µm particle size silica gel 60 Å (200-400 mesh) purchased from Prolabo.

NMR spectroscopy was performed using Bruker spectrometers, either an AV3-400 or AV3-400Nano. ^{1}H NMR, ^{2}H NMR and ^{13}C NMR spectra were recorded on these spectrometers operating at 400 MHz, 61 MHz and 101 MHz, respectively. All spectral data were acquired at 295 K. Chemical shifts (δ) are quoted in parts per million (ppm) relative to the following residual solvent peaks, δ_H 7.26 and δ_C 77.16 for $CDCl_3$, δ_H 2.05 and δ_C 29.84 for $(CD_3)_2CO$, δ_H 5.32 and δ_C 53.5 for DCM-d_2, and δ_H 7.16 and δ_C 128.0 for C_6D_6 was used as a reference. In ^{2}H NMR spectra, samples made up in $CHCl_3$ were doped with a small quantity of $CDCl_3$ to provide a reference peak. Coupling constants (*J*) are reported in Hertz (Hz) to the nearest 0.1 Hz. The multiplicity abbreviations used are: s (singlet), d (doublet), t (triplet), q (quartet), m (multiplet), dd (doublet of doublets), br (broad). Signal assignment was achieved by analysis of nOe and HSQC experiments where required. For quantitative ^{1}H NMR, the pulse delay was extended from 2 seconds to 10 seconds, and the number of scans was increased from 4 scans to 16 scans, using a known mass of 1,1,2,2-tetrachloroethane (TCE) as an internal standard. Infrared (IR) spectra were obtained on a Shimadzu IRAffinity-1 FTIR-ATR spectrometer instrument. Melting points were determined using a Gallenkamp Griffin Melting Point Apparatus.

Due to a change in instrument columns and detectors mid-study, GC-MS and GC-FID spectra will be referred to as either Method 1 or Method 2. GCMS spectra were obtained on an Agilent 7890A GC system coupled to a 5975C inert XL EI/CI MSD triple axis-mass detector. Electron impact (EI) ionisation was utilised, specifically the method EI320.

(Method 1). Fitted with an Agilent HP5 column (30 m x 0.25 mm x 0.25 µm). The column maximum temperature was 320 °C, and the carrier gas was helium with a flow rate of 1 mL/min and was operated in splitless mode.

(Method 2) Fitted with an Agilent DB-5MS column (30 m x 0.32 mm x 0.25 µm). The column maximum temperature was 320 °C, and the carrier gas was helium with a flow rate of 2 mL/min and was operated in 1:10 split mode.

GC-FID analyses were carried out using an Agilent 7890A gas chromatograph with FID detector. (Method 1) Fitted with an Agilent HP5 column (30 m x 0.25 mm x 0.25 µm). Helium was used as the carrier gas (2.0 mL/min flow rate). The maximum column temperature was 320 °C and was operated in splitless mode. (Method 2) Fitted with an Agilent DB-5MS column (30 m x 0.32 mm x 0.25 µm). Helium was used as the carrier gas (2.0 mL/min flow rate). The maximum column temperature was 320 °C and was operated in splitless mode. For calibrations of compounds by GC-FID, see page S127.

High resolution mass spectrometry (HRMS) was carried out using a Thermo-Fisher Scientific Exactive Plus Orbi-Trap Mass Spectrometer with accurate mass measurements obtained using an Orbi-Trap Mass Analyser using Atmospheric Pressure Chemical Ionisation (APCI) or using Electrospray Ionisation (ESI). The mass range was scanned upon direct injection using positive/negative switching for 1.8 minutes (mass range 150-2000 Da) managed by a Thermo-Fisher Scientific Vanquish UHPLC (10 µL injection, with 1 mL/min flow rate using MeOH with 0.1% formic acid as carrier solvent. Additional HRMS was performed at the University of Swansea, at the National Mass Spectrometry Centre. In these cases, GC/MS using the Waters GCT Premier TOF-MS (accurate to 5 ppm at 7,000 resolution using perfluorotributylamine as the internal calibration standard) was performed on all samples

Blank GC-MS spectrum

A number of key products in this study are identified by GC-MS, with their retention times and fragmentation patterns compared with those of authentic samples. As they are often present in trace quantities, noise from the baseline due to polysiloxanes bleeding from the column can be visible in the mass-spec traces for the peaks of products present in trace amounts. A solvent (EtOAc) blank GC-MS spectrum is provided below, in which noise from the baseline across the whole spectrum is shown. These common masses can be observed in the MS of products which are present in trace amounts.

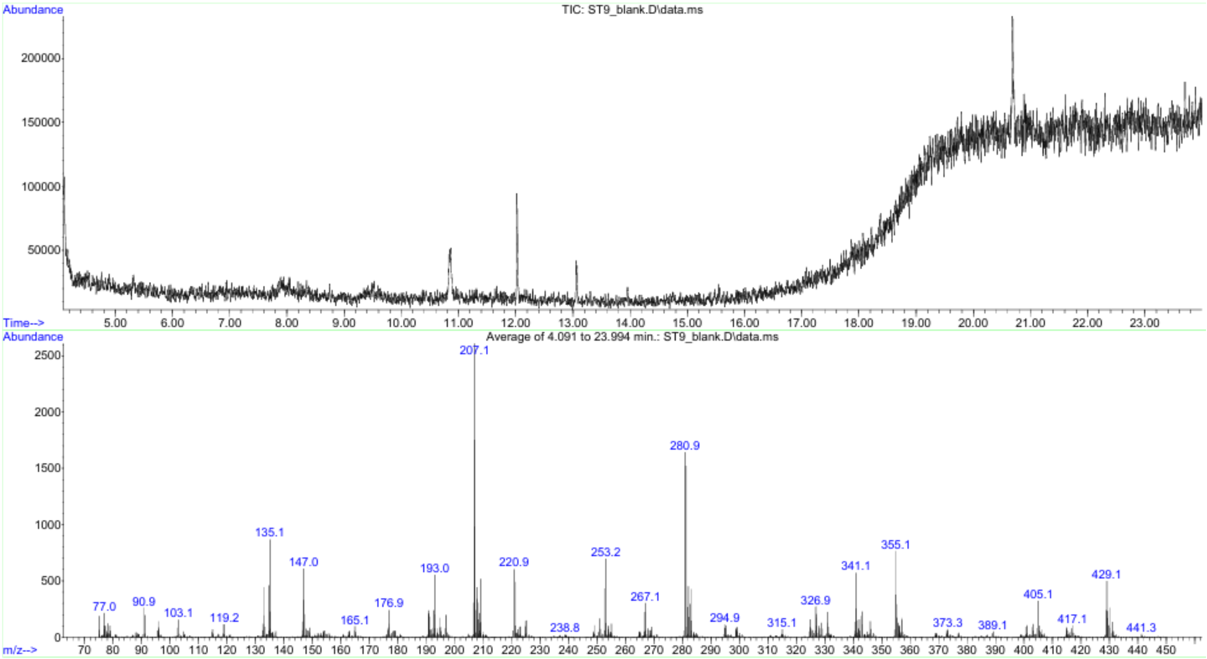

GC-MS spectrum of a blank reaction showing bleed peaks in GC, with a mass spec trace taken to record bleed from the GC column from the entire run-time.

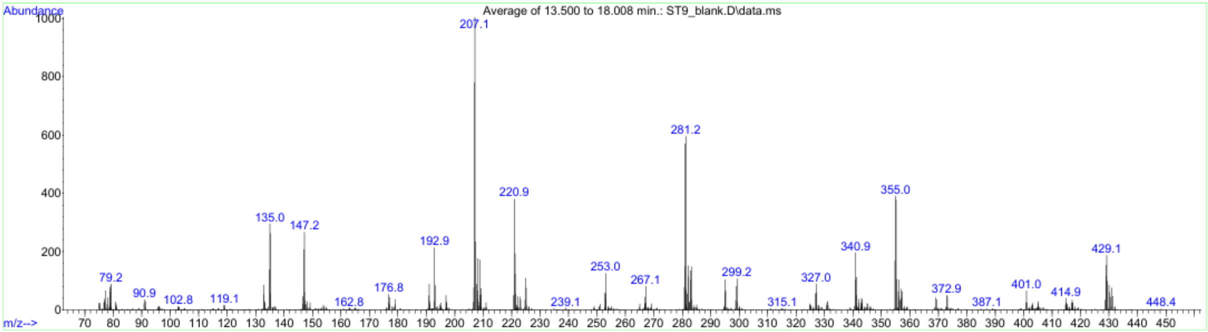

Mass trace of a blank reaction, taken from times 13 min – 18 min, which spans the retention times of ortho-, meta- and para-terphenyls, along with triphenylene. These background polysiloxane masses can be seen as background in the mass spectra of the trace terphenyls and triphenylene.

Reactions of iodobenzene 1

Reactions of iodobenzene isotopologues with KO*t*Bu in C_6H_6

Two reactions were carried out in a side-by-side manner. To an oven-dried microwave vial, primed with a stirrer bar, in a glovebox was added iodobenzene **1** (39 µL, 0.35 mmol, 1 equiv.) or iodobenzene-*d*$_5$ **1-*d*$_5$** (40 µL, 0.35 mmol, 1 equiv.) with KO*t*Bu (79 mg, 0.7 mmol, 2 equiv.) and benzene (3.5 mL), with the vials subsequently sealed and stirred at 130°C in an oil bath for exactly 24 h. Once complete, the crude mixtures were cooled to room temperature, then H_2O (0.2 mL) and an accurately weighed amount of *n*-dodecane in EtOAc (5 mL) was added. An aliquot of the crude mixtures was then analysed by both GCMS and GC-FID (Method 2). The crude mixtures were then washed with water (10 mL), extracted into EtOAc (2 x 10 mL), washed with brine, dried over $MgSO_4$ and concentrated. The residue was dissolved in $CDCl_3$, a known weight of 1,1,2,2-tetrachloroethane (TCE) added to the mixture and a portion analysed by quantitative ^{1}H NMR to determine the yield of *tert*-butoxybenzene **36** (Ar-OC(CH_3)$_3$, δ = 1.35 ppm, 9 H).

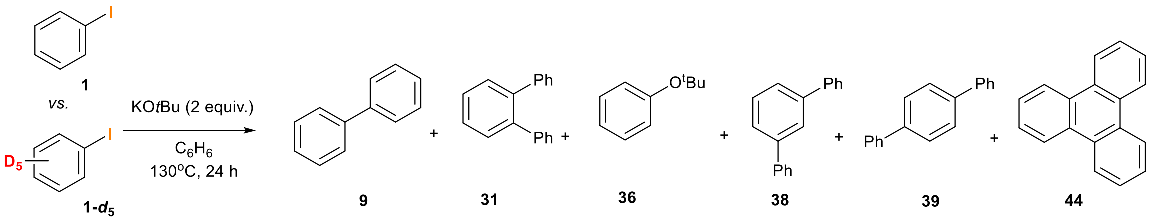

Or deuterated isotopologues, depending on substrate

The reaction was carried out twice, with the average yields listed below (the individual trials are noted on pages S10-S11):

**Substrate**		**% Yield**				
	**1**[a]	**9**[a]	**31**[a]	**36**[b]	**38**[a]	**39**[a]
**1**	36.7	27.2	0.18	16.1	0.08	0.05
**1-*d*$_5$**	52.6	11.9	0.41	18.3	0.03	0.10

[a]Yields determined by GC-FID calibrated with authentic samples. Isotopologue yields calculated using calibrations of non-labelled compounds. [b]Yields determined by quantitative ^{1}H NMR using TCE as an internal standard.

GCMS data for reaction of 1-d_0 , including mass spectra of individual products

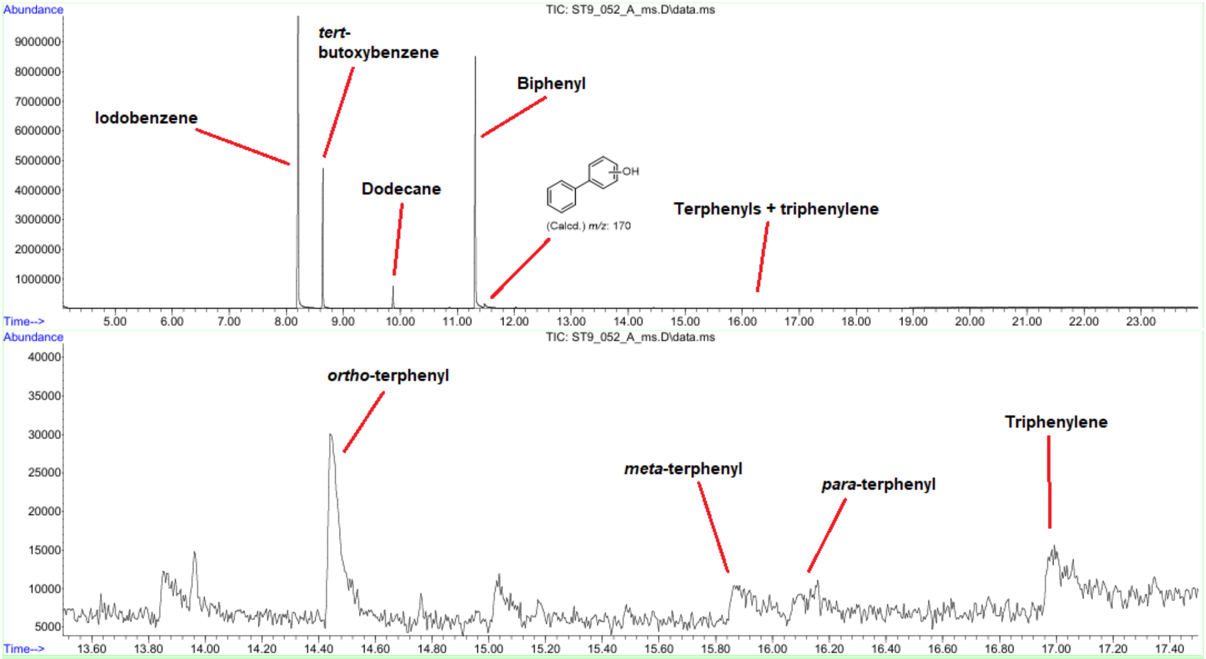

GC-MS of the crude reaction mixture. Unlabelled peaks in the baseline are polysiloxanes due to column bleeding.

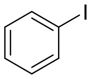

(Calcd.) *m/z*: 203.9436 (100.0%), 204.9469 (6.5%)

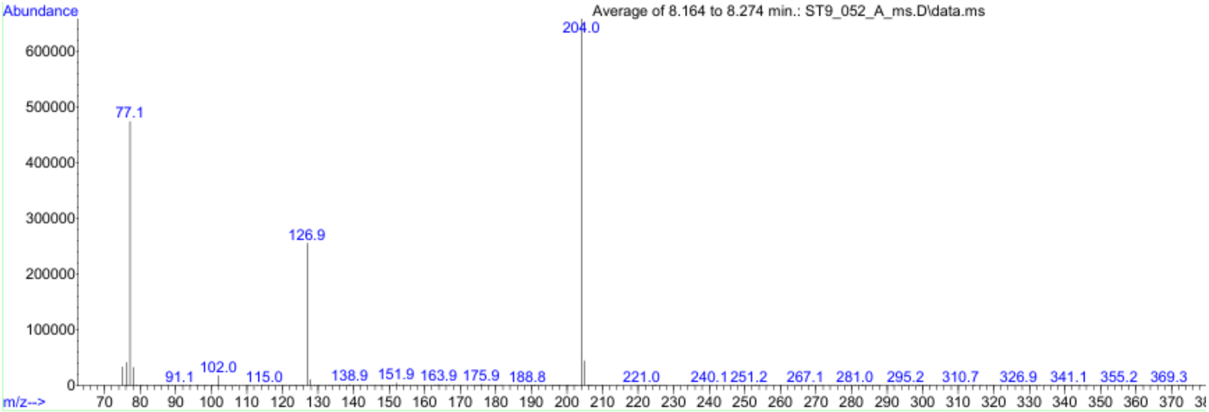

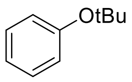

(Calcd.) *m/z* : 150.1045 (100.0%), 151.1078 (10.8%)

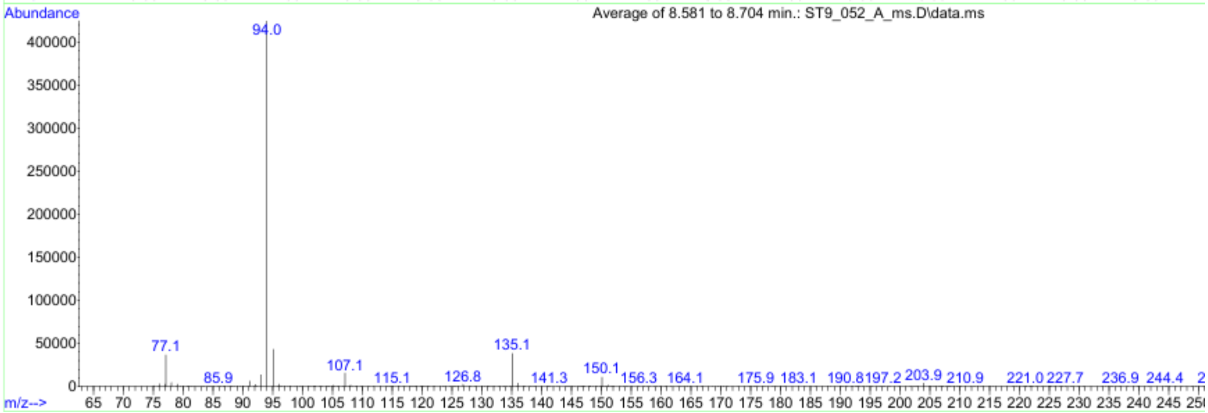

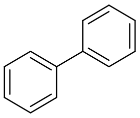

(Calcd.) *m/z*: 154.0783 (100.0%), 155.0816 (13.0%)

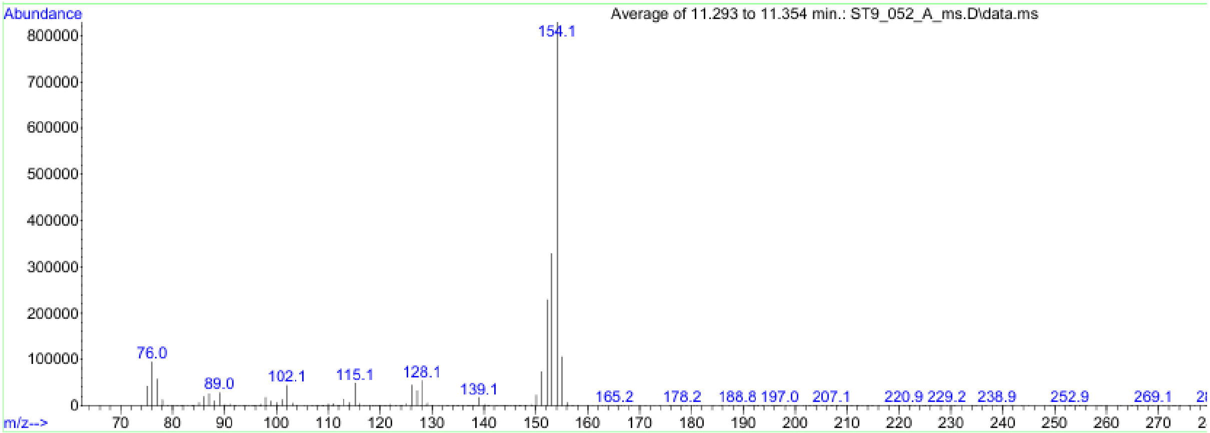

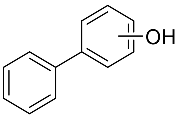

(Calcd.) *m/z*: 170.0732 (100.0%), 171.0765 (13.0%)

This product likely arose from base-induced E2 elimination from *t*BuOC$_6$H$_4$Ph)

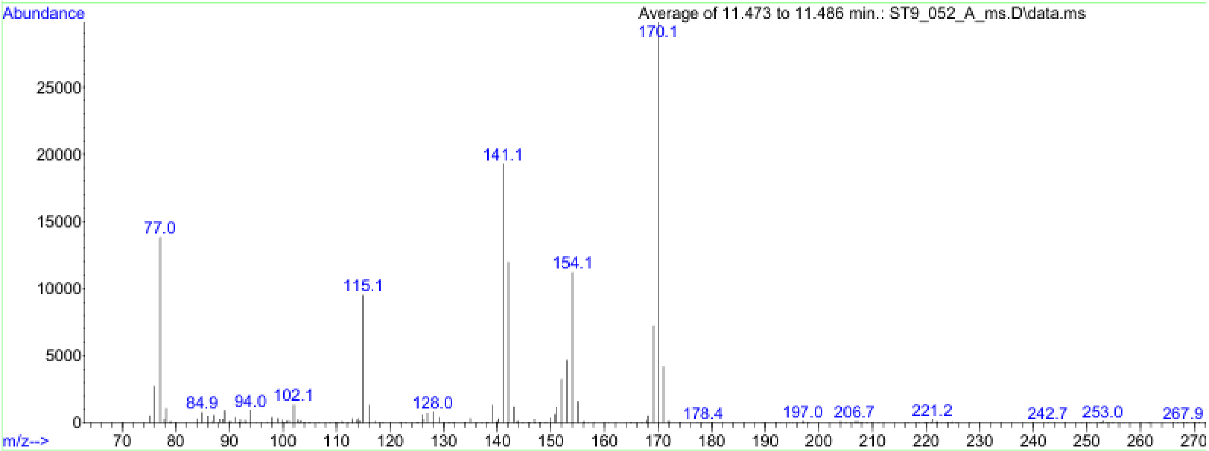

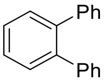

(Calcd.) *m/z*: 230.1096 (100.0%), 231.1129 (19.5%), 232.1163 (1.8%)

For GCMS of a prepared authentic sample of *o*-terphenyl, see page S18

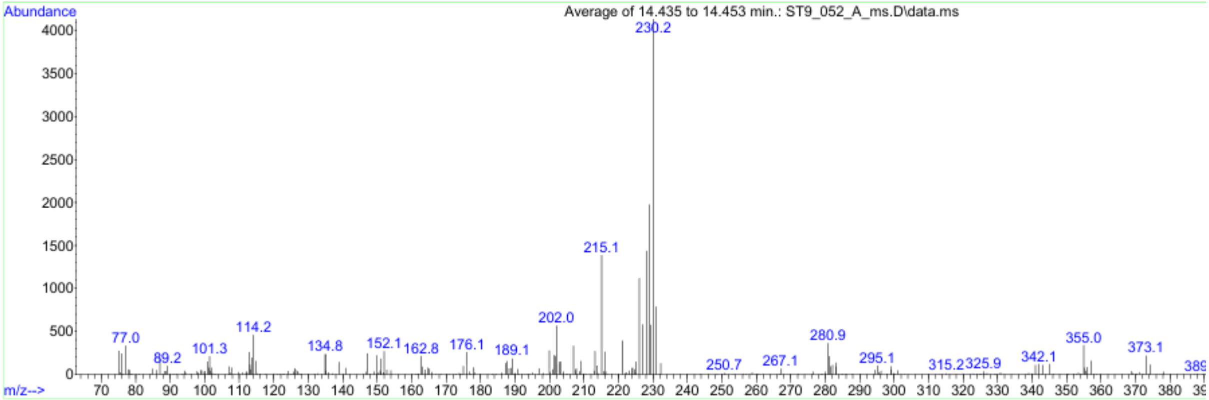

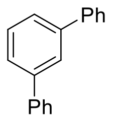

(Calcd.) *m/z*: 230.1096 (100.0%), 231.1129 (19.5%), 232.1163 (1.8%)

For GCMS of a prepared authentic sample of *m*-terphenyl, see page S18

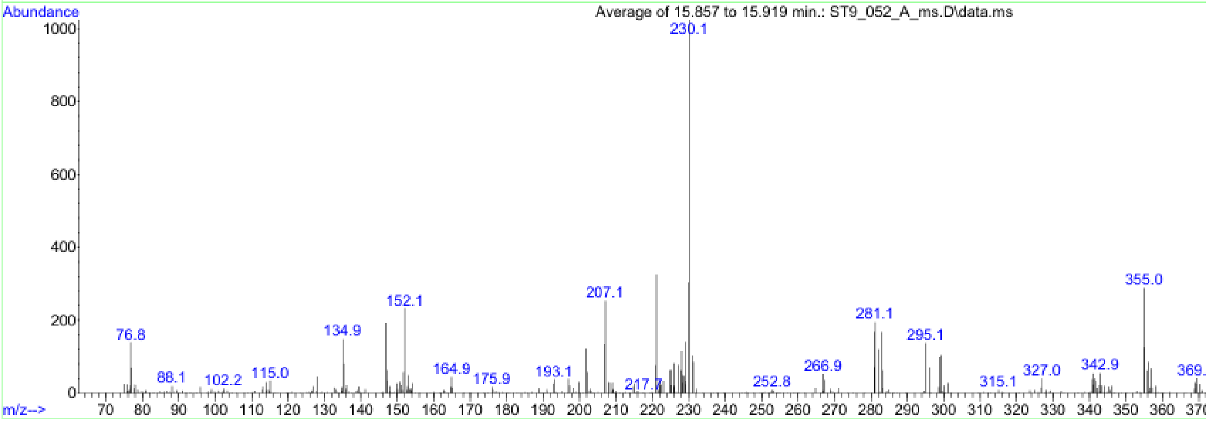

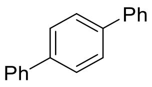

(Calcd.) *m/z*: 230.1096 (100.0%), 231.1129 (19.5%), 232.1163 (1.8%)

For GCMS of a commercial authentic sample of *p*-terphenyl, see page S18

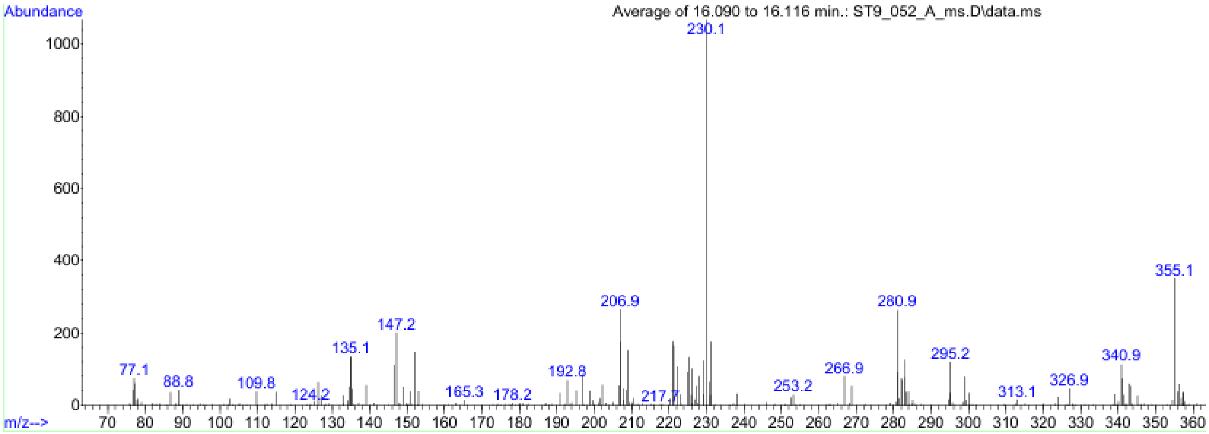

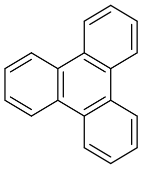

(Calcd.) *m/z*: 228.0939 (100.0%), 229.0973 (19.5%), 230.1006 (1.8%)

For GCMS of a purchased authentic sample of triphenylene, see page S19

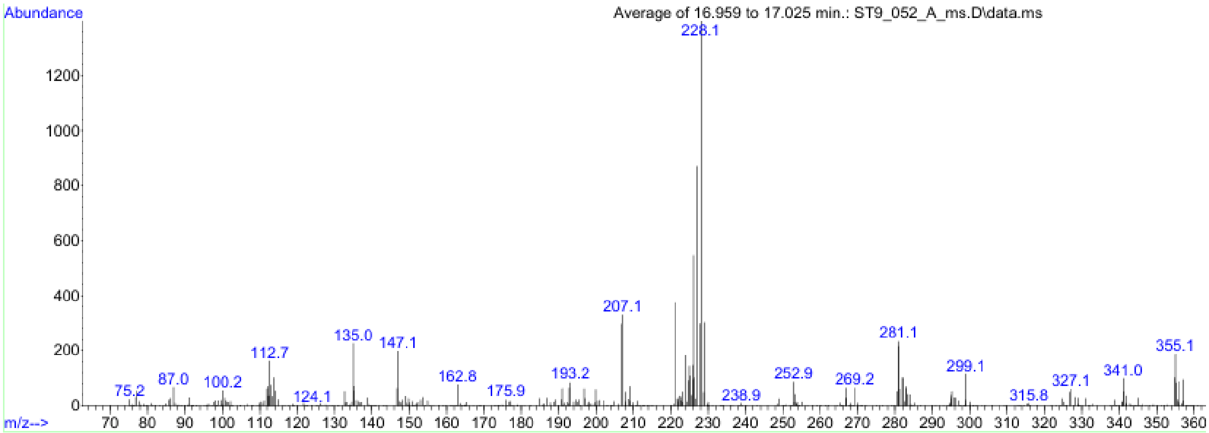

GC-FID and ^{1}H NMR data for reactions of 1-d_0 including table quantitating components that had been separately calibrated. Here are the data for the two separate runs:

Run 1

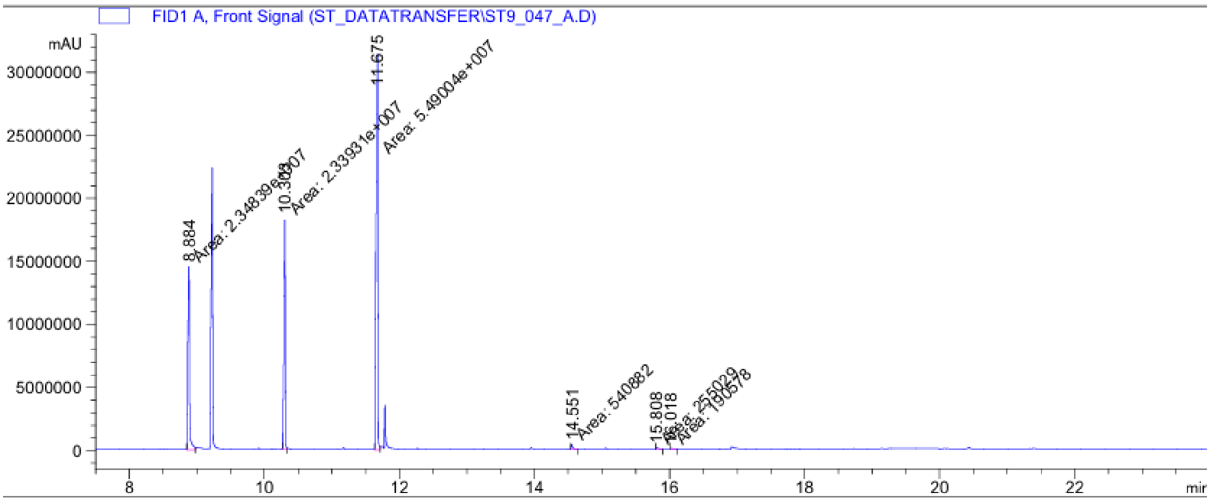

Retention Time (min)	Sample	Peak Area	%Yield
8.884	Iodobenzene	23483900	31.1
10.303	Dodecane	23393100	N/A
11.676	Biphenyl	54900400	29.8
14.551	*o*-terphenyl	540882	0.19
15.808	*m*-terphenyl	255029	0.08
16.018	*p*-terphenyl	190578	0.05

Dodecane added = 8.0 mg

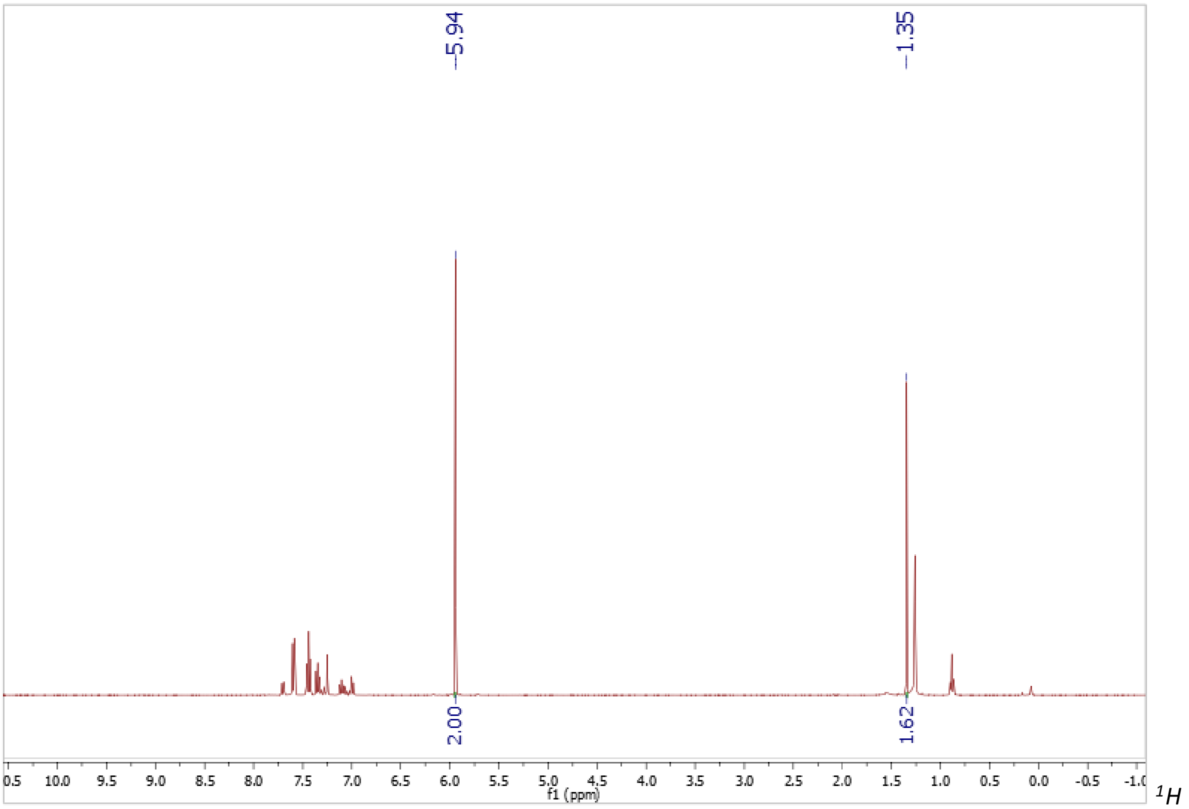

NMR of crude reaction mixture. (Quantitation of tert-butoxybenzene ***36****). TCE added = 51.2 mg. Signal at δ 5.94 ppm = TCE, δ 1.35 ppm = tert-butoxybenzene. The singlet at 1.26 ppm and the triplet at 0.88 ppm are due to residual n-dodecane in sample.*

Run 2

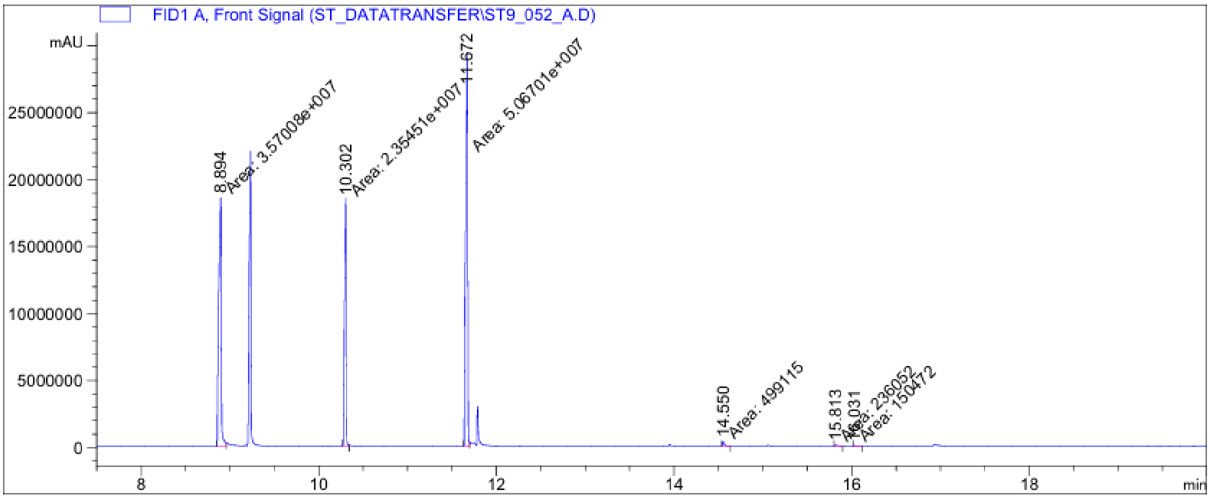

Retention Time (min)	Sample	Peak Area	%Yield
8.894	Iodobenzene	35700800	42.3
10.302	Dodecane	23545100	N/A
11.672	Biphenyl	50670100	24.6
14.550	*o*-terphenyl	499115	0.16
15.813	*m*-terphenyl	236052	0.07
16.031	*p*-terphenyl	150472	0.04

Dodecane added = 7.2 mg

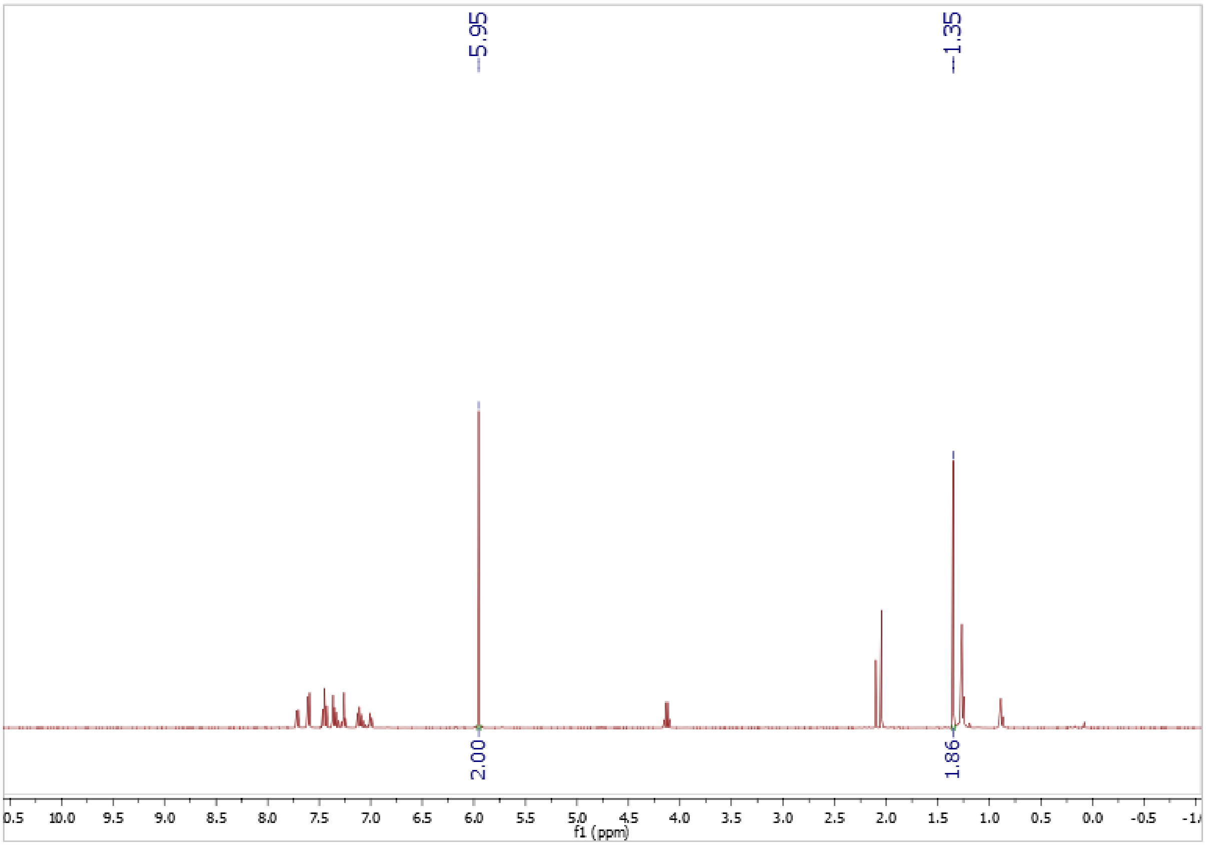

^{1}H NMR of crude reaction mixture. (Quantitation of **36**). TCE added = 46.6 mg. Signal at δ 5.94 ppm = TCE, δ 1.35 ppm = tert-butoxybenzene. The singlet at 1.26 ppm and the triplet at 0.88 ppm are due to residual n-dodecane in sample. Traces of EtOAc are also visible.

GCMS data for reaction of 1-d_5 *with KOtBu in* C_6H_6 including mass spectra of individual products

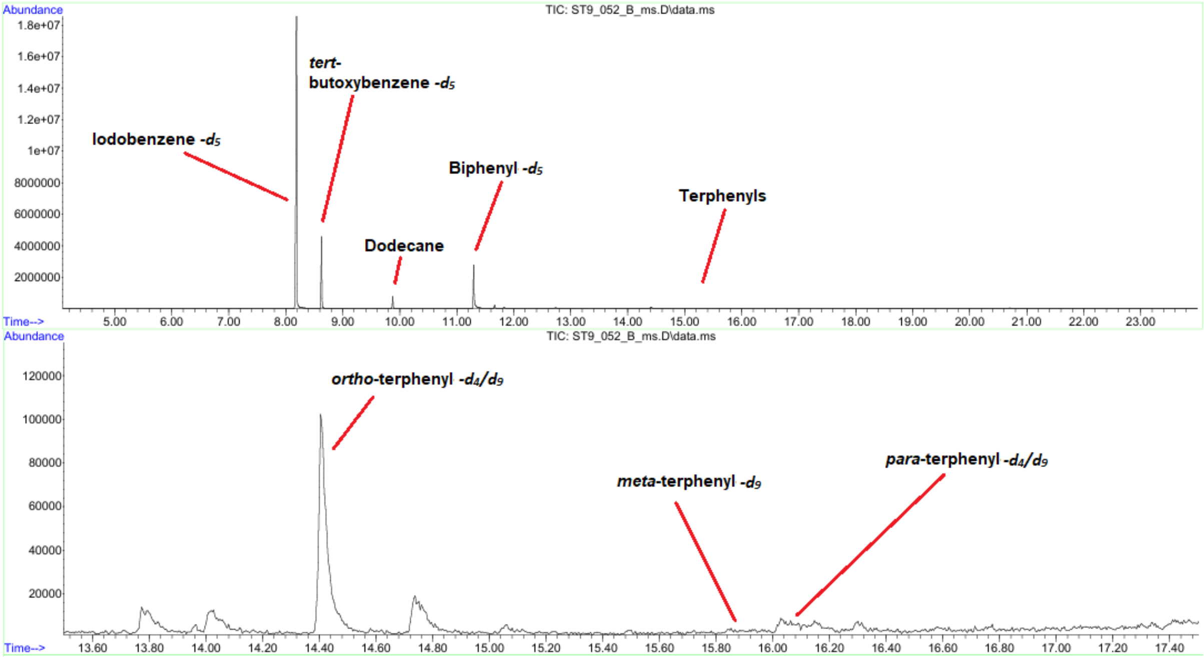

GC-MS of the crude reaction mixture. Unlabbelled peaks in the baseline are polysiloxanes due to column bleeding.

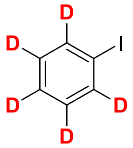

(Calcd.) m/z: 208.9750 (100.0%), 209.9783 (6.5%)

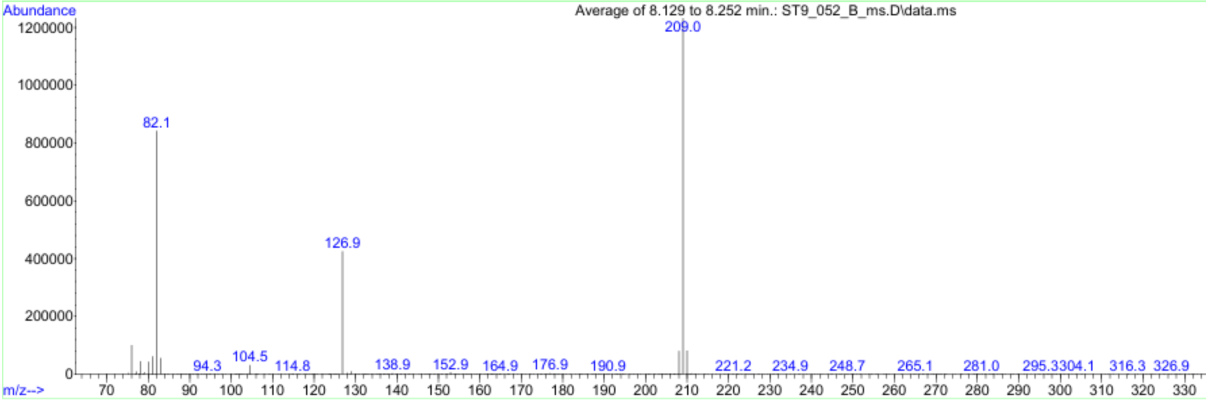

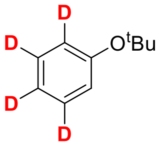

(Calcd.) m/z: 154.1296 (100.0%), 155.1329 (10.8%)
(Observed) m/z: 139.1 [(M-Me)$^+$]

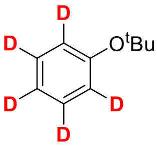

(Calcd.) m/z: 155.1358 (100.0%), 156.1392 (10.8%)
(Observed) m/z: 140.1 [(M-Me)$^+$]

Here we observe both **36-*d*$_4$** and **36-*d*$_5$**, as the aryl anion formed after attack of *tert*-butoxide onto the benzyne is highly reactive and thus not selective in being protonated.

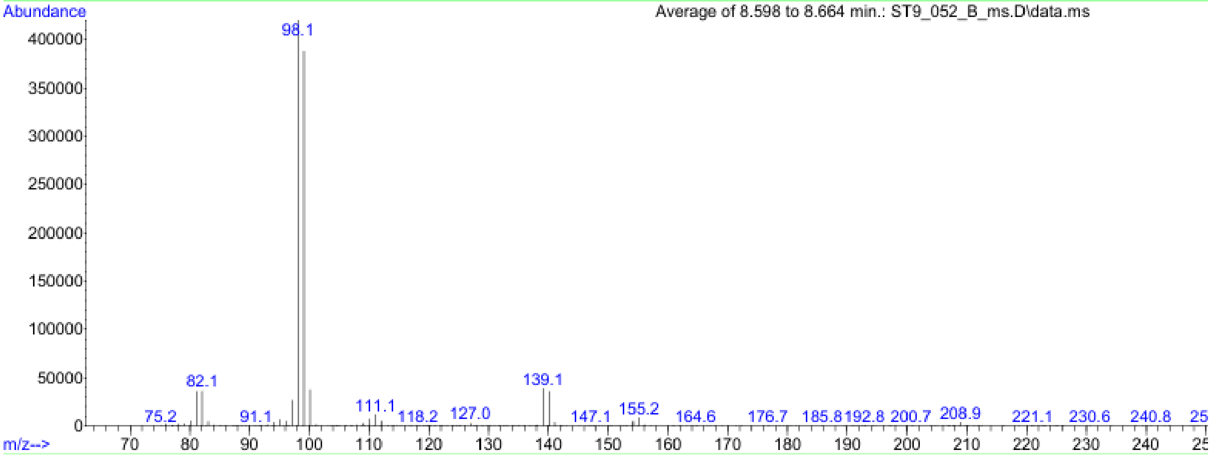

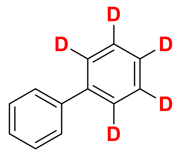

(Calcd.) *m/z*: 159.1096 (100.0%), 160.1130 (13.0%)

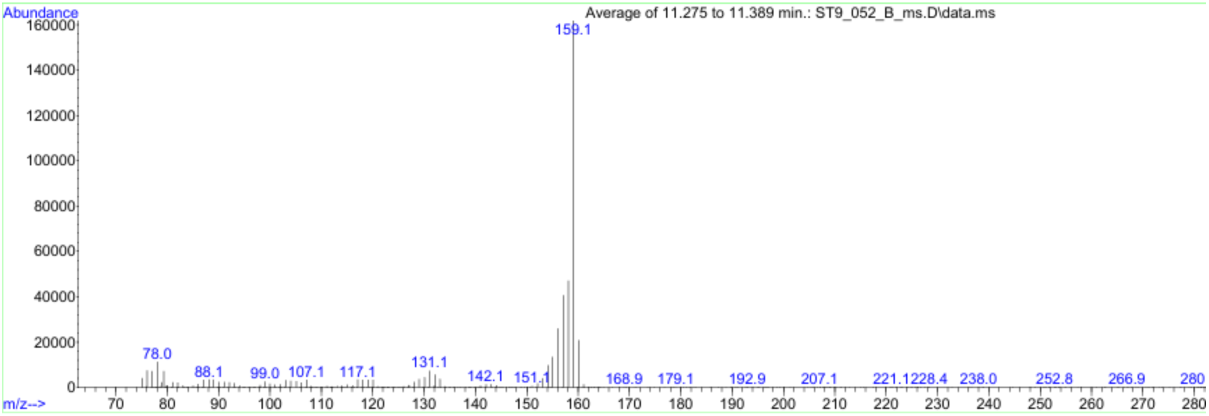

Below is the mass spectrum of the *o*-terphenyl, showing the -d_4 isotopologue (major) and -d_9 isotopologue (minor).

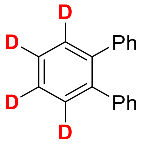

(Calcd.) *m/z*: 234.1347 (100.0%), 235.1380 (19.5%), 236.1414 (1.8%)

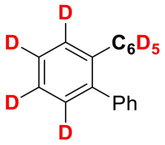

(Calcd.) *m/z*: 239.1660 (100.0%), 240.1694 (19.5%), 241.1728 (1.8%)

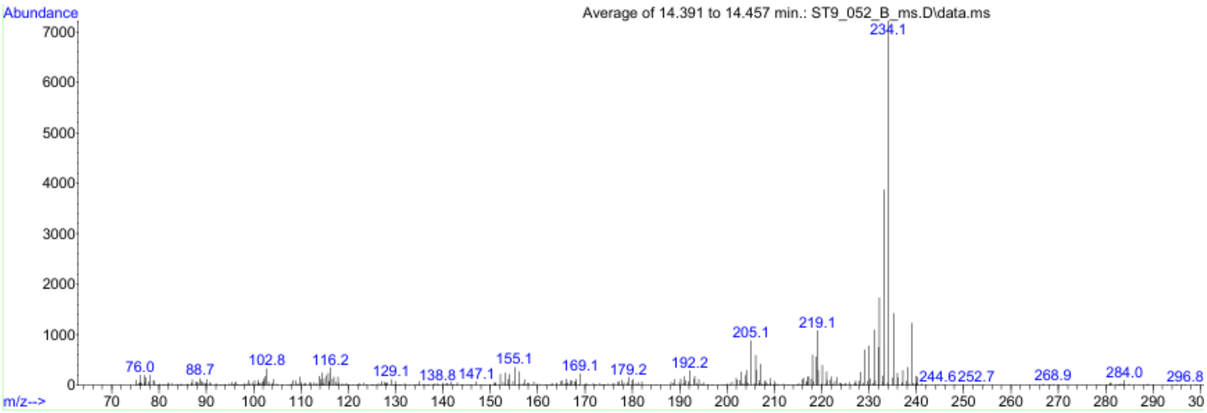

Below is the mass spectrum of the *m*-terphenyl, showing the d_9-isotopologue and almost no d_4-isotopologue

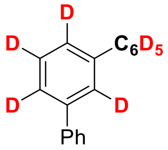

(Calcd.) *m/z*: 239.1660 (100.0%), 240.1694 (19.5%), 241.1728 (1.8%)

m/z = 239 is the dominant isotopologue of the *m*-product (*m/z* = 281 is from the bleeding of the silylated material of the column - see page S4)

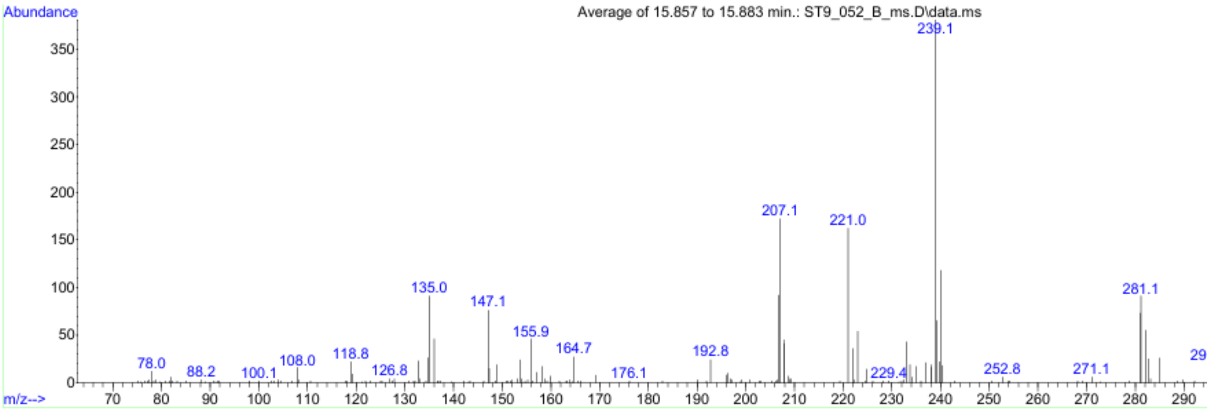

Below is the mass spectrum of the *p*-terphenyl, showing the d_4-isotopologue (major) and the d_9-isotopologue (minor).

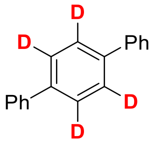

(Calcd.) *m/z*: 234.1347 (100.0%), 235.1380 (19.5%), 236.1414 (1.8%)

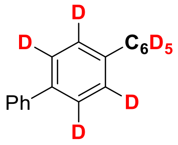

(Calcd.) *m/z*: 239.1660 (100.0%), 240.1694 (19.5%), 241.1728 (1.8%)

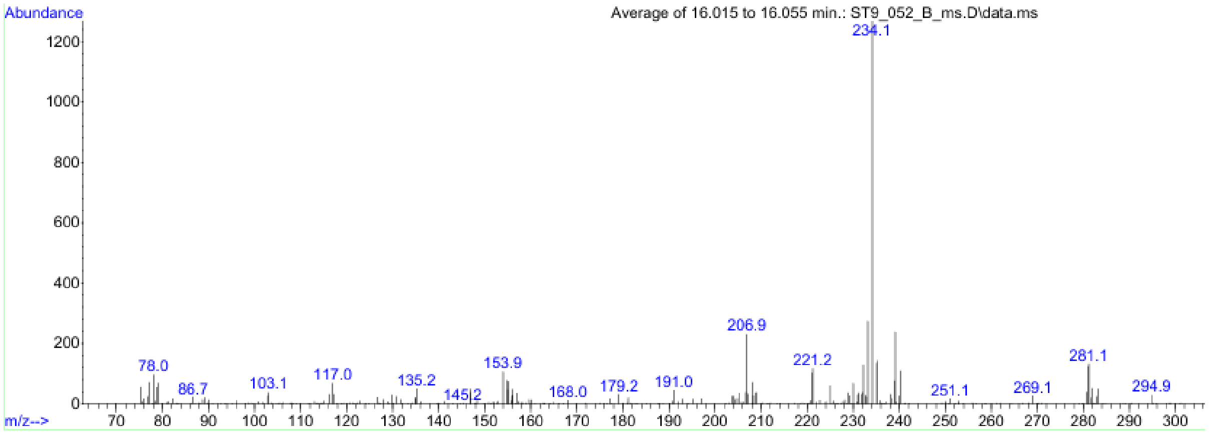

GC-FID and ^{1}H NMR data for reactions of 1-d_5 including table quantitating components that had been separately calibrated.

Run 1

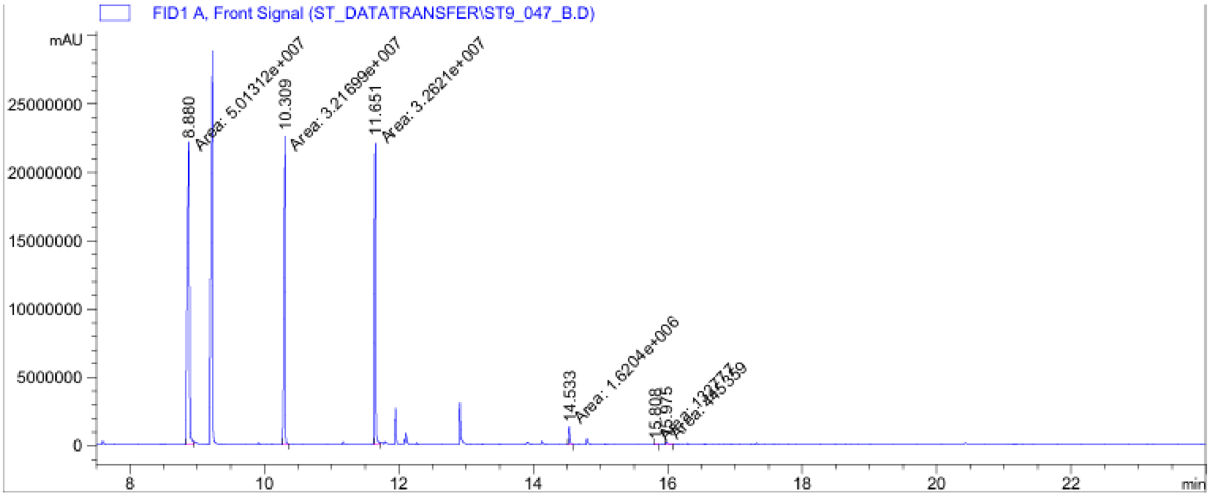

Retention Time (min)	Sample	Peak Area	%Yield
8.880	Iodobenzene	50131200	47.1
10.309	Dodecane	32169900	N/A
11.651	Biphenyl	32621000	12.6
14.553	*o*-terphenyl	1620400	0.41
15.808	*m*-terphenyl	132777	0.03
15.975	*p*-terphenyl	445359	0.10

Dodecane added = 7.8 mg

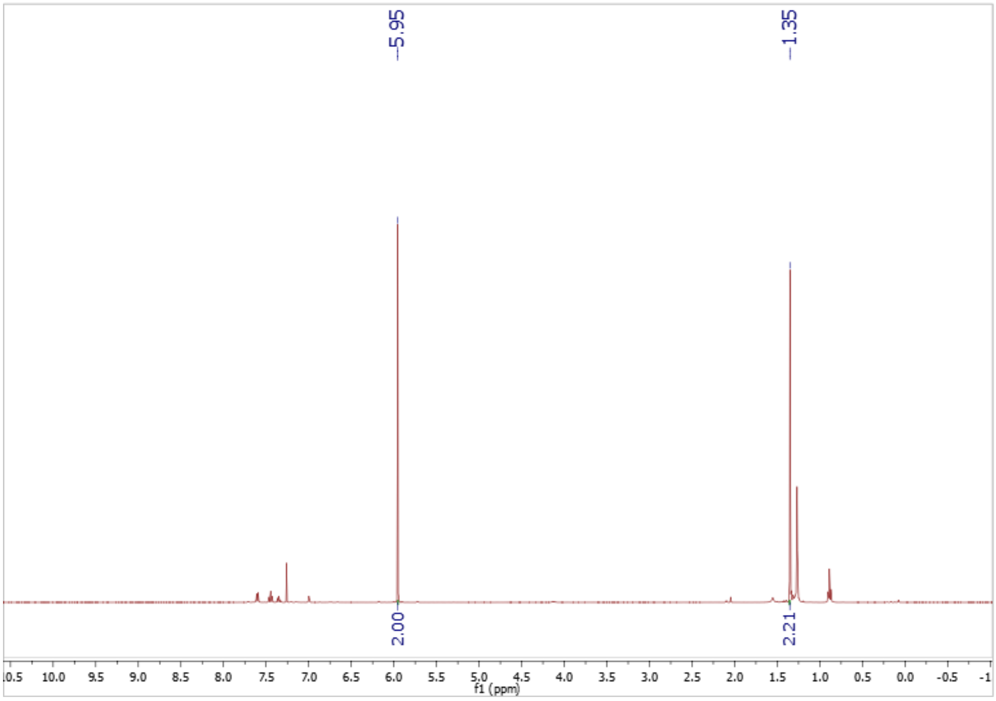

*^{1}H NMR of crude reaction mixture. (Quantitation of **36**). TCE added = 51.9 mg. Signal at δ 5.94 ppm = TCE, δ 1.35 ppm = tert-butoxybenzene. The singlet at 1.26 ppm and the triplet at 0.88 ppm are due to residual n-dodecane.*

Run 2

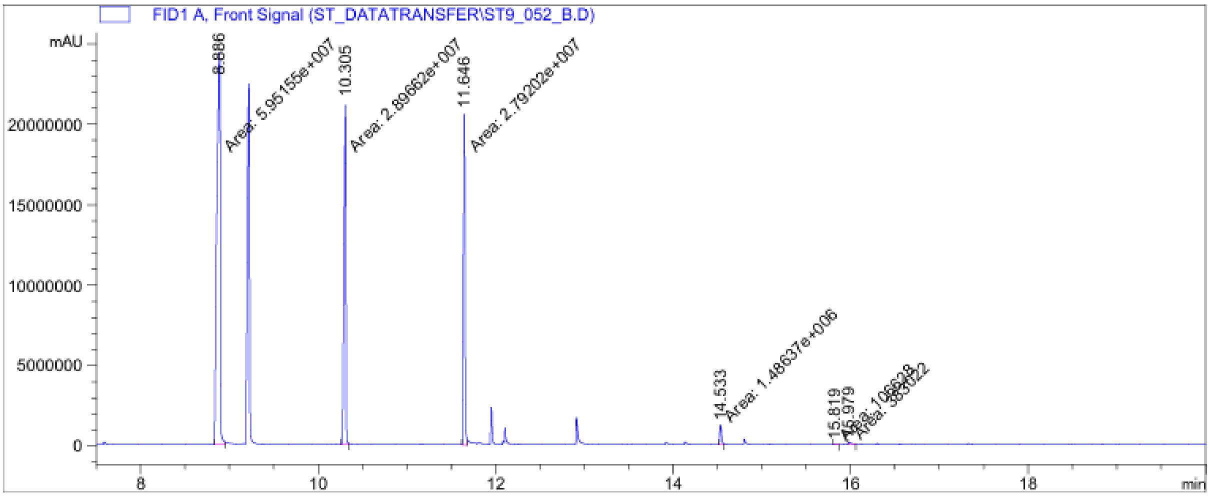

Retention Time (min)	Sample	Peak Area	%Yield
8.886	Iodobenzene	59515500	58.1
10.305	Dodecane	28966200	N/A
11.646	Biphenyl	27920200	11.1
14.533	*o*-terphenyl	1486370	0.40
15.819	*m*-terphenyl	106628	0.03
15.979	*p*-terphenyl	383022	0.09

Dodecane added = 7.3 mg

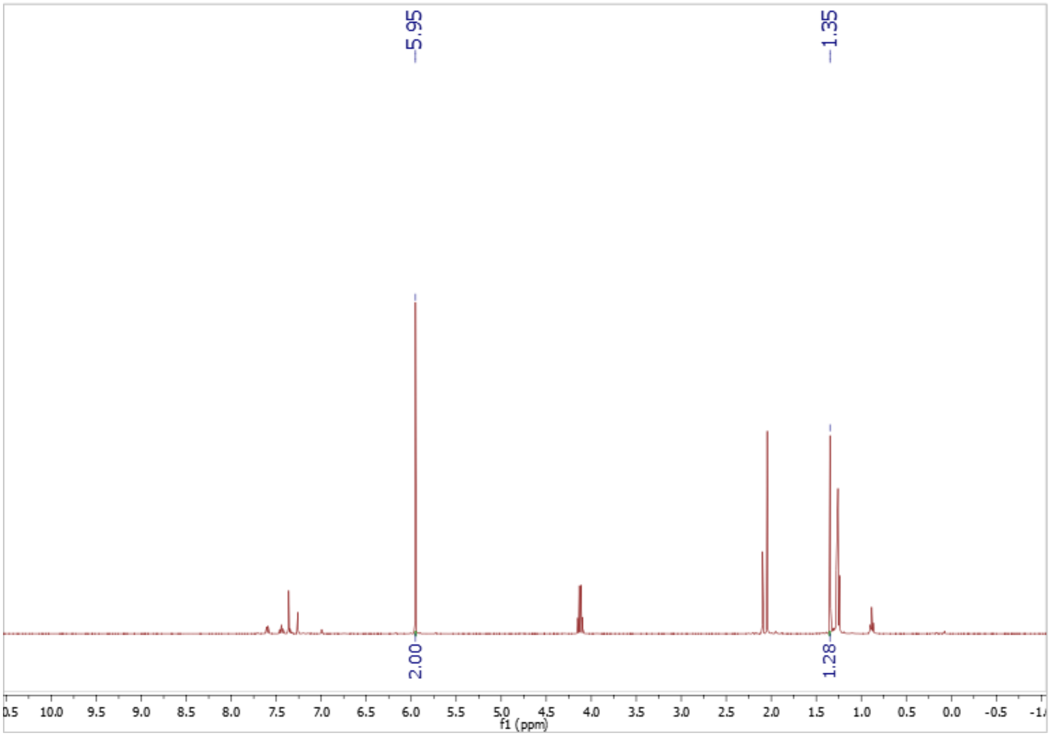

*^{1}H NMR of crude reaction mixture. (Quantitation of **36**). TCE added = 61.5 mg. Signal at δ 5.94 ppm = TCE, δ 1.35 ppm = tert-butoxybenzene. The singlet at 1.26 ppm and the triplet at 0.88 ppm are due to residual n-dodecane in sample. Traces of EtOAc are also visible.*

GC-MS of authentic samples of trace products - *o, m-* and *p*-terphenyl and triphenylene

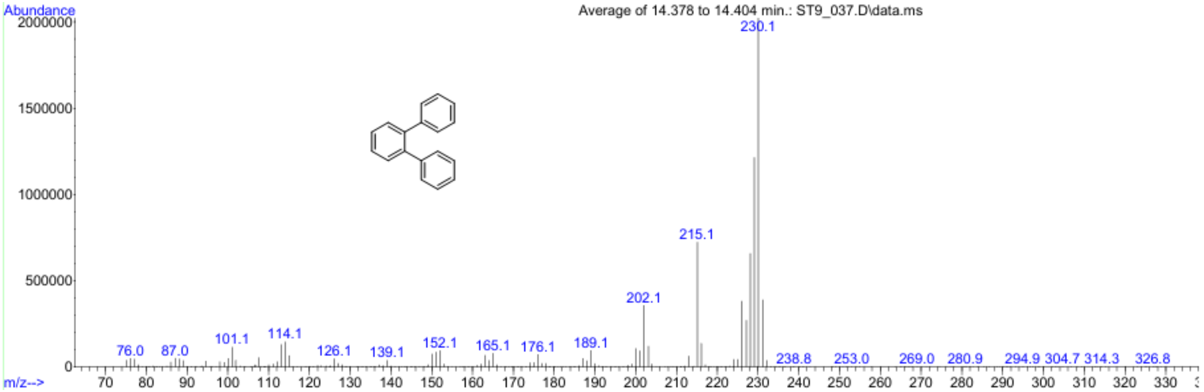

GC-MS spectrum of authentic sample of synthesised *ortho*-terphenyl (see synthesis section, page S105). Retention time ≈ 14.4 min.

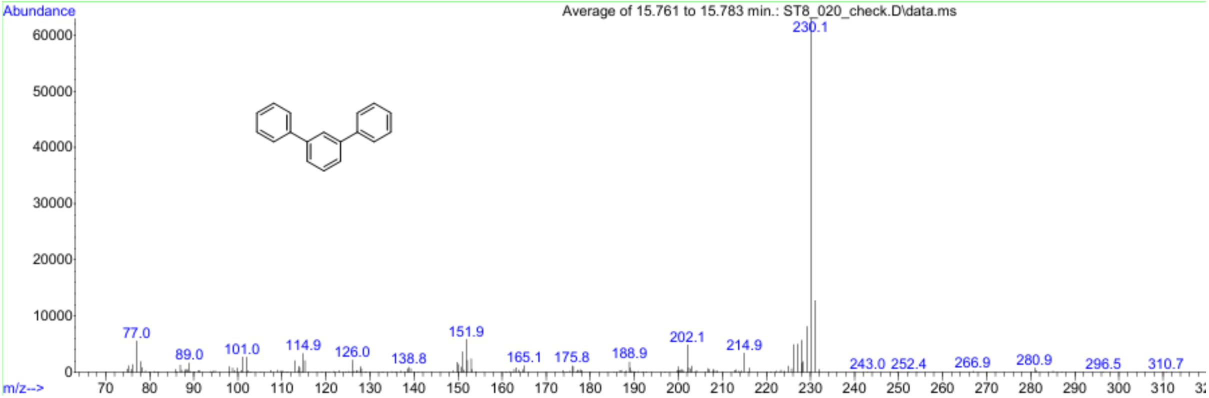

GC-MS spectrum of authentic sample of synthesised *meta*-terphenyl (see synthesis section, page S104. Retention time ≈ 15.8 min.

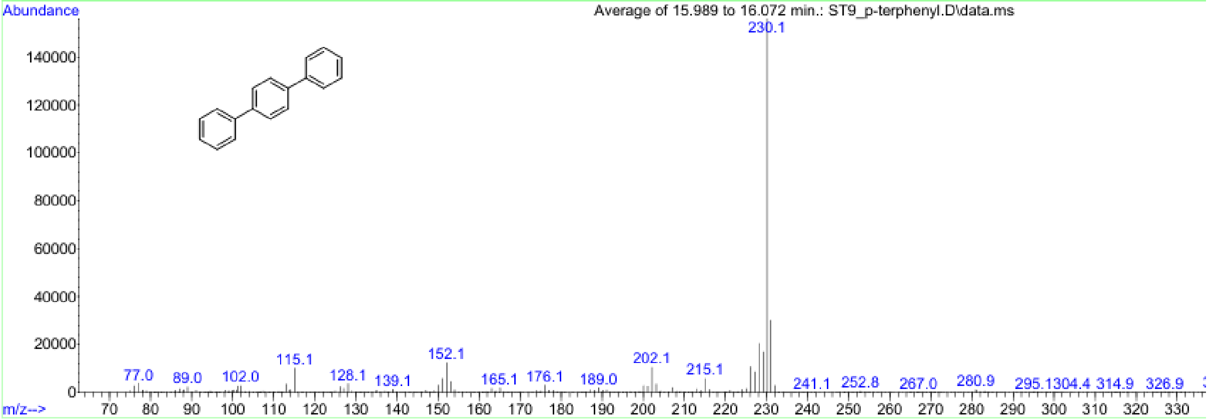

GC-MS spectrum of authentic sample of commercial *para*-terphenyl (Thermo Fisher). Retention time ≈ 16.0 min.

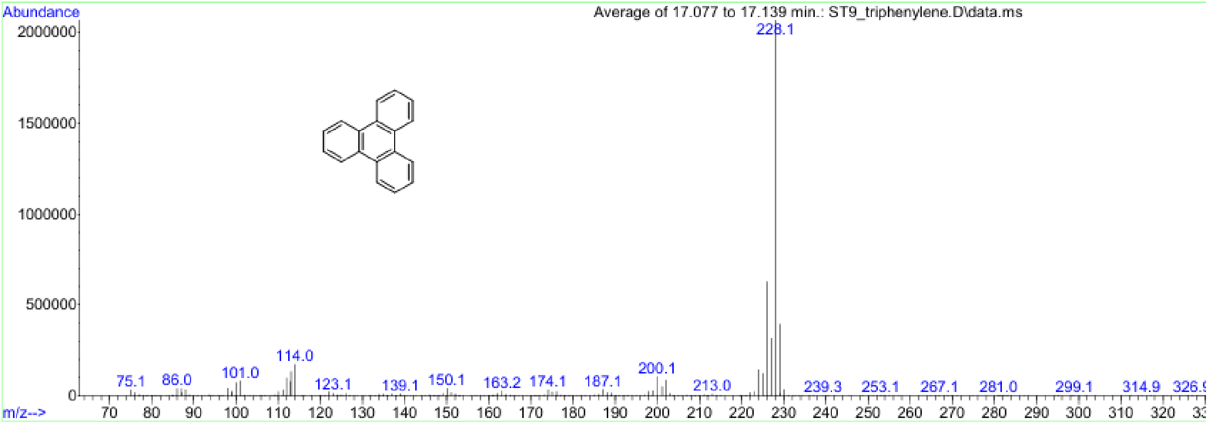

GC-MS spectrum of authentic sample of commercial *para*-terphenyl (Thermo Fisher). Retention time ≈ 17.1 min.

Reaction of iodobenzene with KO*t*Bu in C_6D_6

To an oven-dried microwave vial, primed with a stirrer bar, in a glovebox was added iodobenzene **1** (39 µL, 0.35 mmol, 1 equiv.) with KO*t*Bu (79 mg, 0.7 mmol, 2 equiv.) and benzene-D_6 (3.5 mL), with the vials subsequently sealed and stirred at 130°C in an oil bath for exactly 24 h. Once complete, the crude mixture was cooled to room temperature, then H_2O (0.2 mL) and an accurately weighed amount of *n*-dodecane in EtOAc (5 mL) was added. An aliquot of the crude mixture was then analysed by both GCMS and GC-FID (Method 2). The crude mixture was then washed with water (10 mL), extracted into EtOAc (2 x 10 mL), washed with brine, dried over $MgSO_4$ and concentrated. The residue was dissolved in $CDCl_3$, a known weight of 1,1,2,2-tetrachloroethane (TCE) added to the mixture and a portion analysed by quantitative ^{1}H NMR to determine the yield of *tert*-butoxybenzene **36** (Ar-OC$(CH_3)_3$, δ = 1.35 ppm, 9 H).

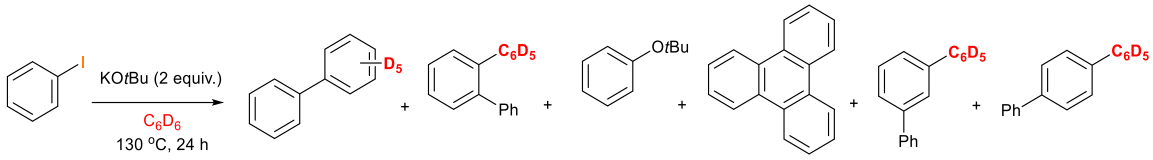

This reaction was carried out twice with the average yields listed below (The two separate runs are reported on pages S23 and S24:

	% Yield				
**1**[a]	**9**[a]	**31**[a]	**36**[b]	**38**[a]	**39**[a]
46.5	21.2	0.15	17.8	0.08	0.05

[a]Yield determined by GC-FID calibrated with authentic samples. Isotopologue yields calculated using calibrations of non-labelled compounds. [b]Yield determined by quantitative ^{1}H NMR using TCE as an internal standard.

GCMS data for the products of reaction of iodobenzene with KO*t*Bu in C_6D_6. including the mass spectra of products.

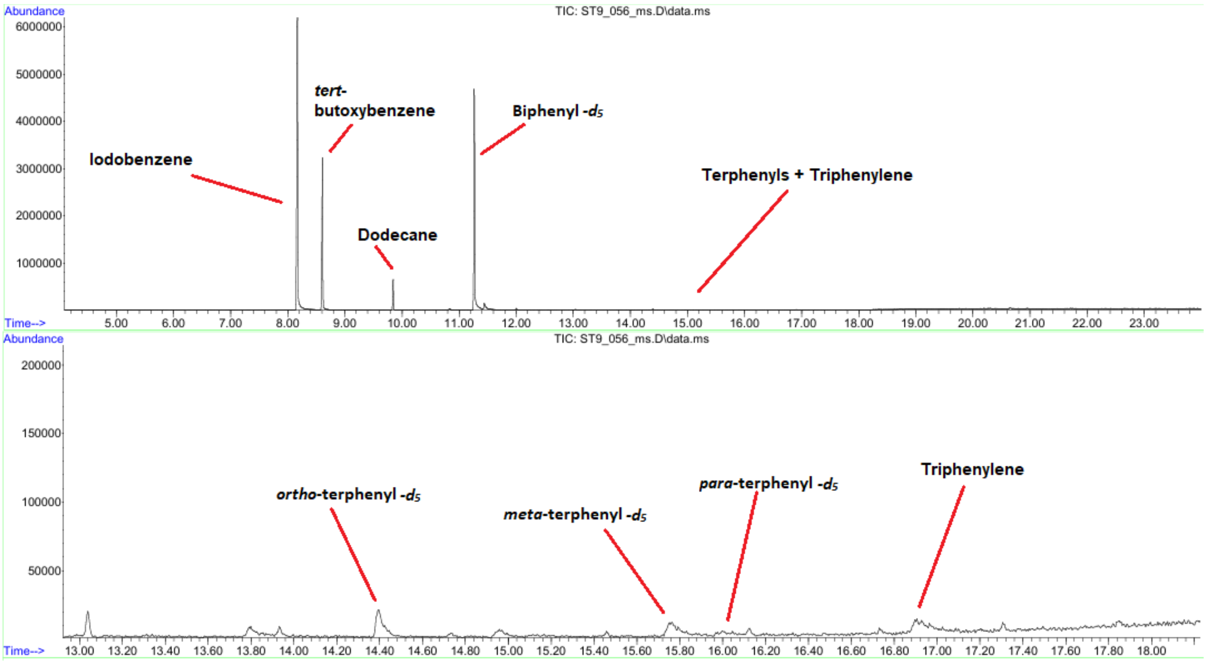

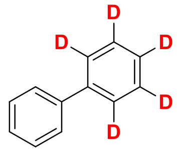

(Calcd.) *m/z*: 159.1096 (100.0%), 160.1130 (13.0%)

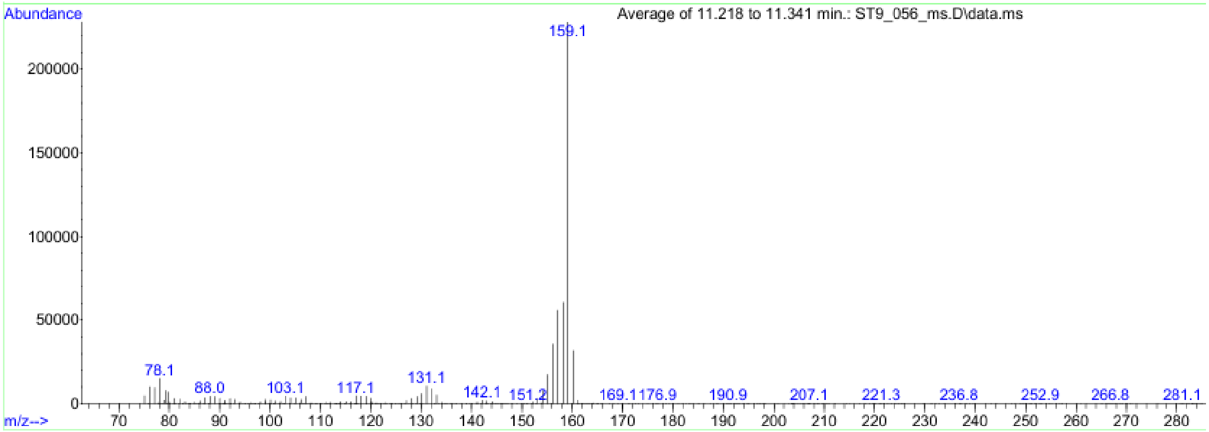

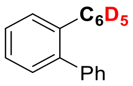

(Calcd.) *m/z*: 235.1409 (100.0%), 236.1443 (19.5%), 237.1476 (1.8%)

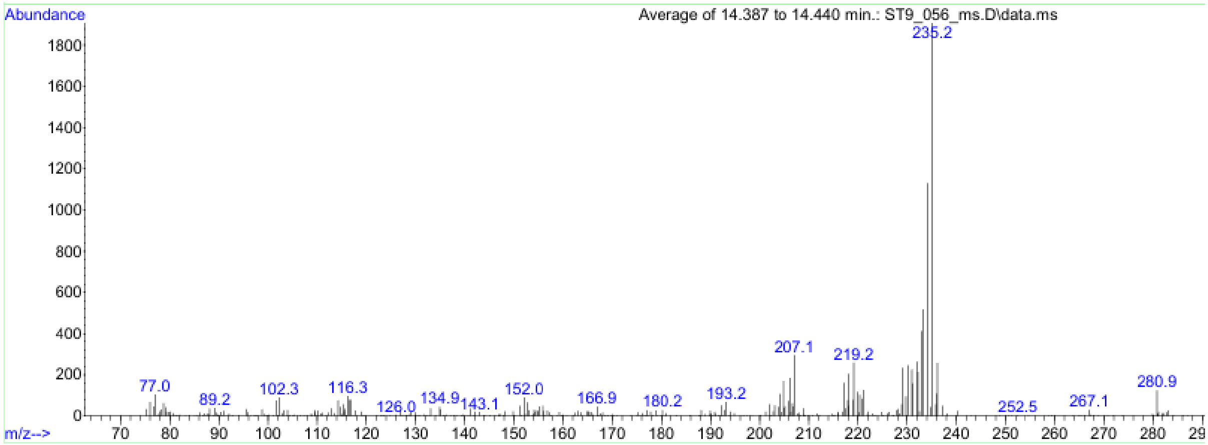

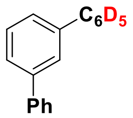

(Calcd.) *m/z*: 235.1409 (100.0%), 236.1443 (19.5%), 237.1476 (1.8%)

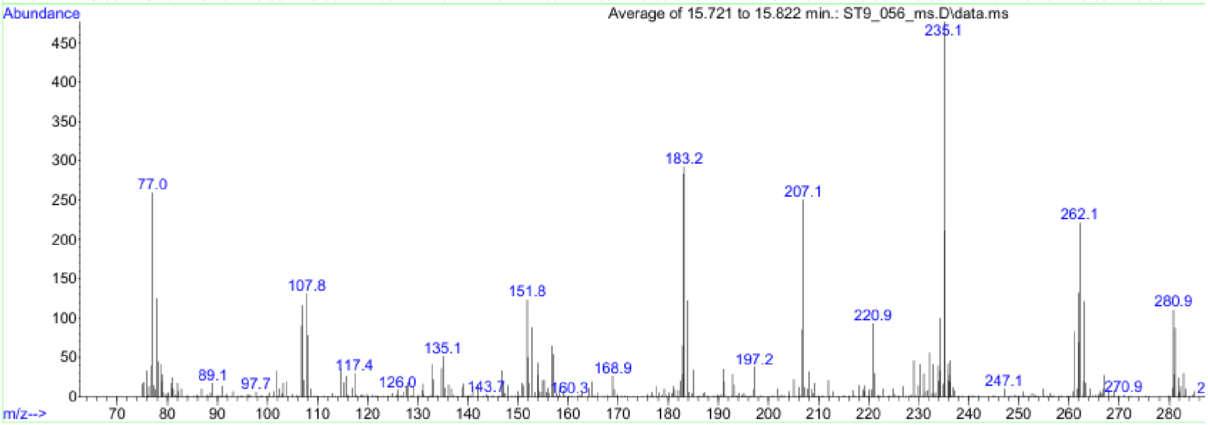

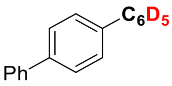

(Calcd.) *m/z*: 235.1409 (100.0%), 236.1443 (19.5%), 237.1476 (1.8%)

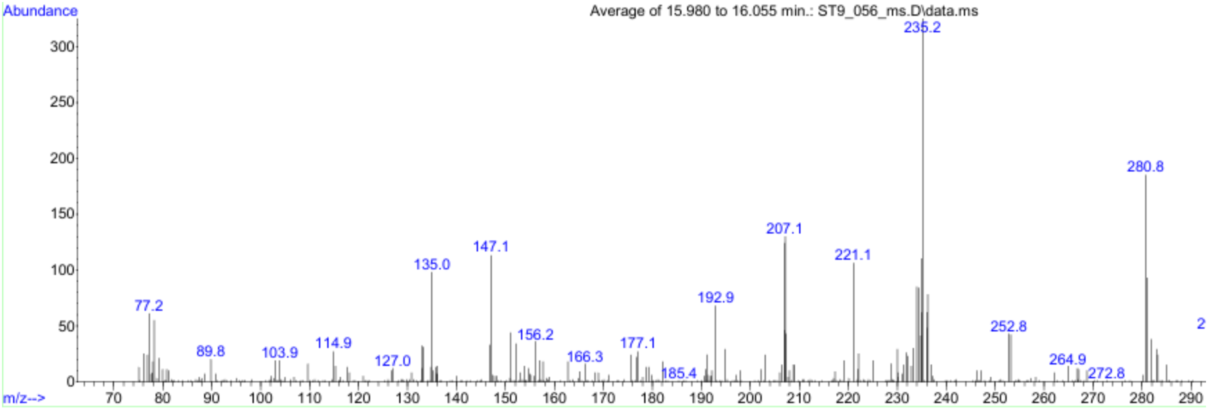

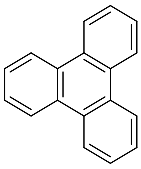

(Calcd.) *m/z*: 228.0939 (100.0%), 229.0973 (19.5%), 230.1006 (1.8%)

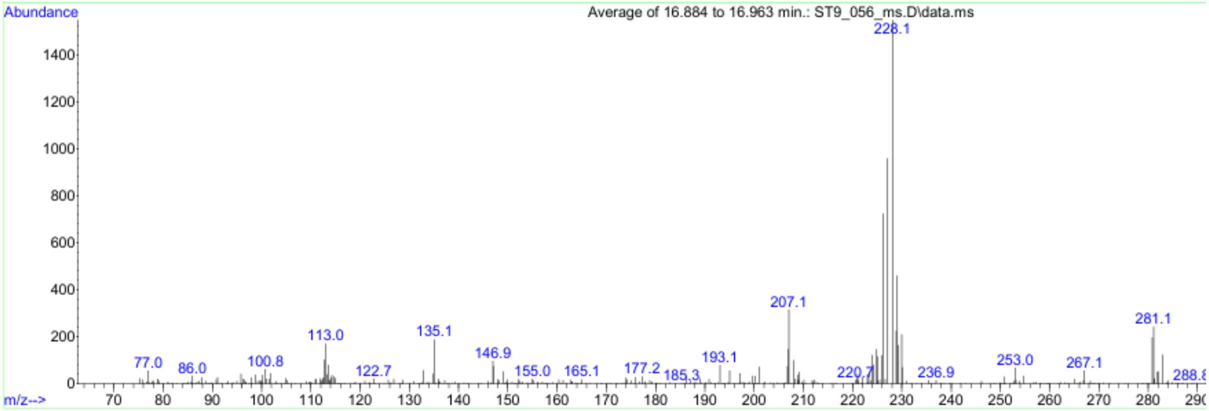

GC-FID and ^{1}H NMR data including table quantitating components that had been separately calibrated.

Run 1

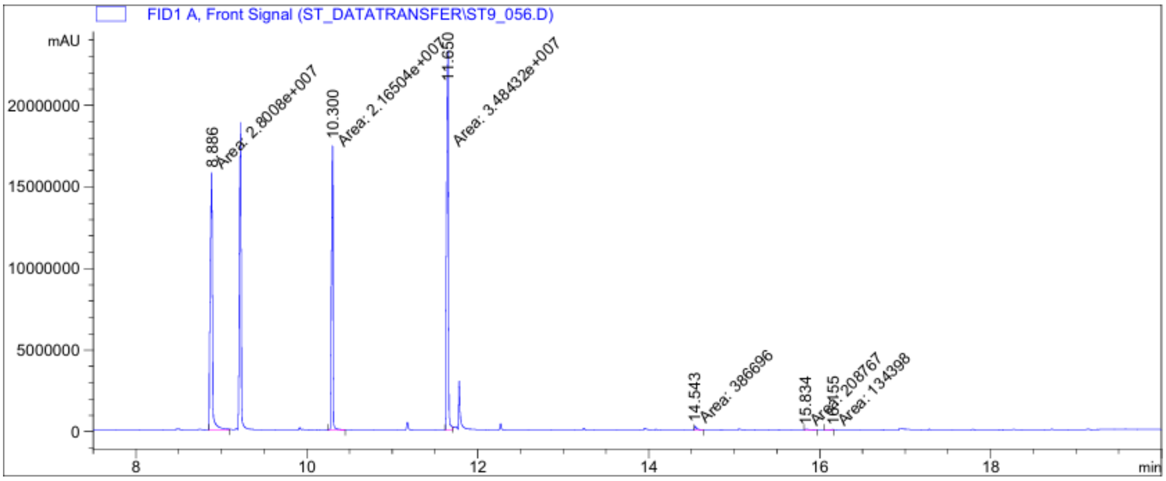

Retention Time (min)	Sample	Peak Area	%Yield
8.886	Iodobenzene	28008000	43.6
10.300	Dodecane	21650400	N/A
11.650	Biphenyl	34843200	22.2
14.543	*o*-terphenyl	386696	0.16
15.834	*m*-terphenyl	208767	0.08
16.155	*p*-terphenyl	134398	0.05

Dodecane added = 8.7 mg

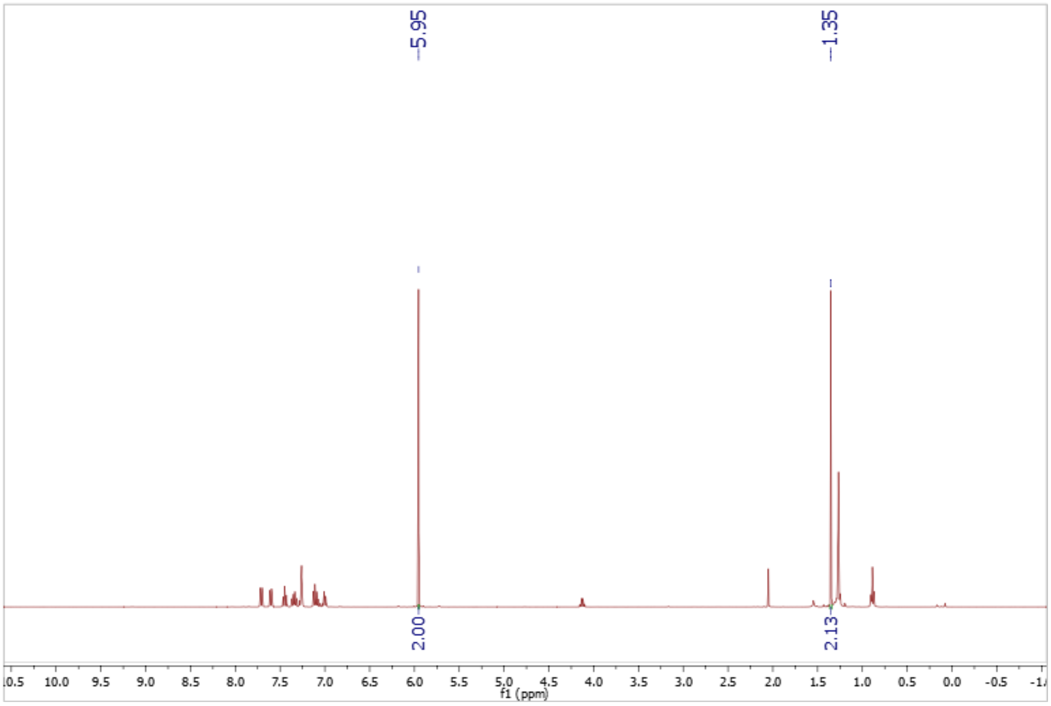

^{1}H NMR spectrum of crude reaction mixture (quantitation of ***36****). TCE added = 43.7 mg. Signal at δ 5.94 ppm = TCE, δ 1.35 ppm = tert-butoxybenzene. The singlet at 1.26 ppm and the triplet at 0.88 ppm are due to residual n-dodecane in sample. Traces of EtOAc are also visible.*

Run 2

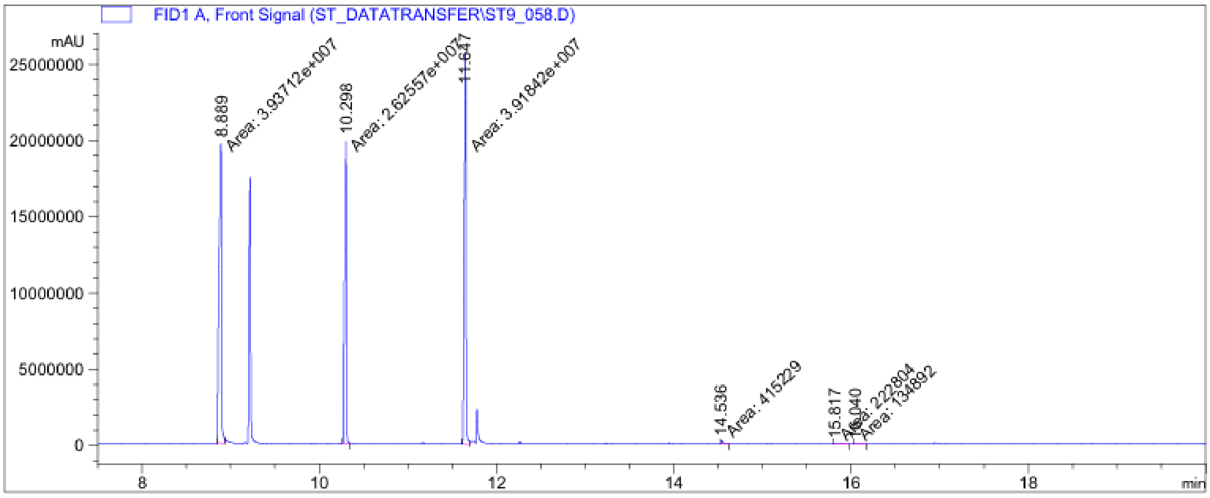

Retention Time (min)	Sample	Peak Area	%Yield
8.889	Iodobenzene	39371200	49.4
10.298	Dodecane	26255700	N/A
11.647	Biphenyl	39184200	20.2
14.536	*o*-terphenyl	415229	0.14
15.817	*m*-terphenyl	222804	0.07
16.040	*p*-terphenyl	134892	0.04

Dodecane added = 8.5 mg

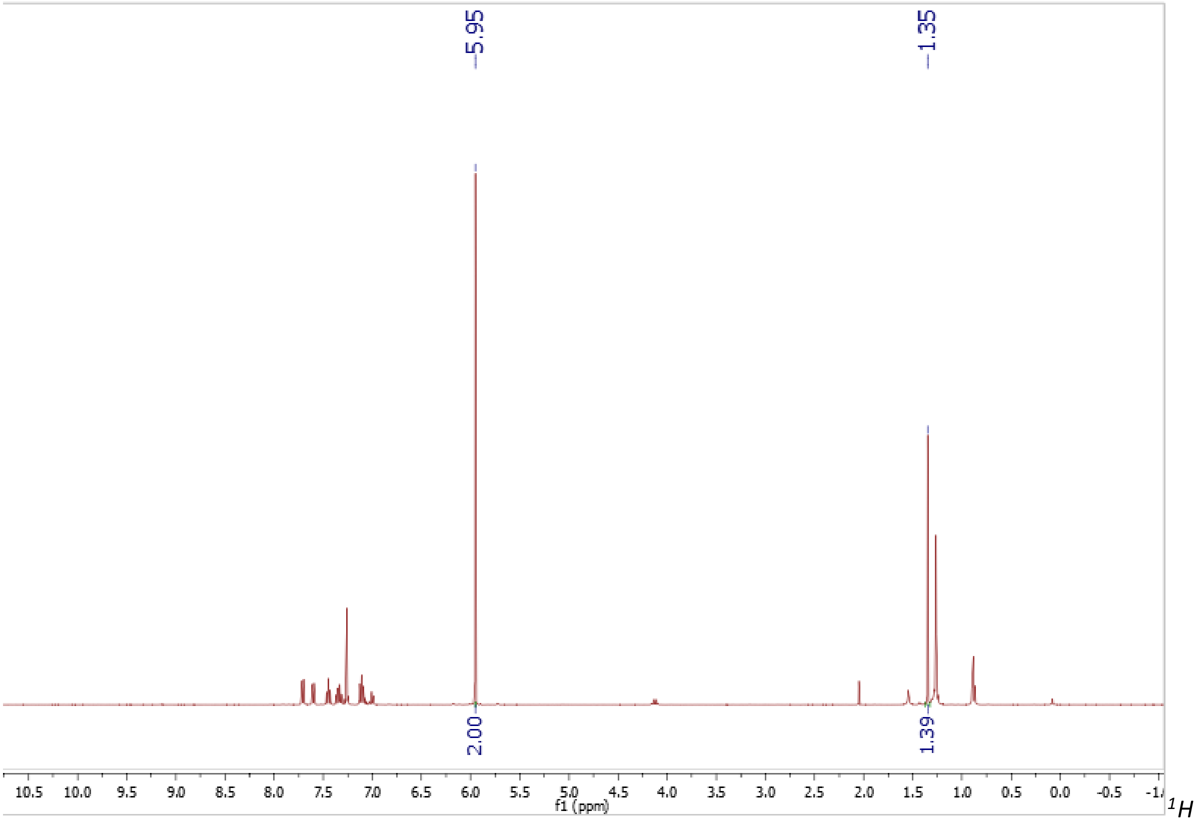

*NMR spectrum of crude reaction mixture (quantitation of **36**). TCE added = 67.4 mg. Signal at δ 5.94 ppm = TCE, δ 1.35 ppm = tert-butoxybenzene. The singlet at 1.26 ppm and the triplet at 0.88 ppm are due to residual n-dodecane in sample. Traces of EtOAc are also visible.*

Reactions of 9-haloanthracene substrates

Reaction of 9-bromoanthracene with KO*t*Bu in C_6H_6 (Table 1, Entry 1)

To an oven-dried microwave vial, primed with a stirrer bar, in a glovebox was added 9-bromoanthracene **40** (180 mg, 0.7 mmol, 1 equiv.), KO*t*Bu (157 mg, 1.4 mmol, 2 equiv.) and benzene (7 mL) with the vial subsequently sealed and stirred at 130°C in an oil bath for 24 h. Once complete, the crude mixture was allowed to cool to room temperature, H_2O (0.2 mL) and an accurately weighed amount of dodecane was added. An aliquot of the crude mixture was then analysed by both GCMS and GC-FID (Method 1).

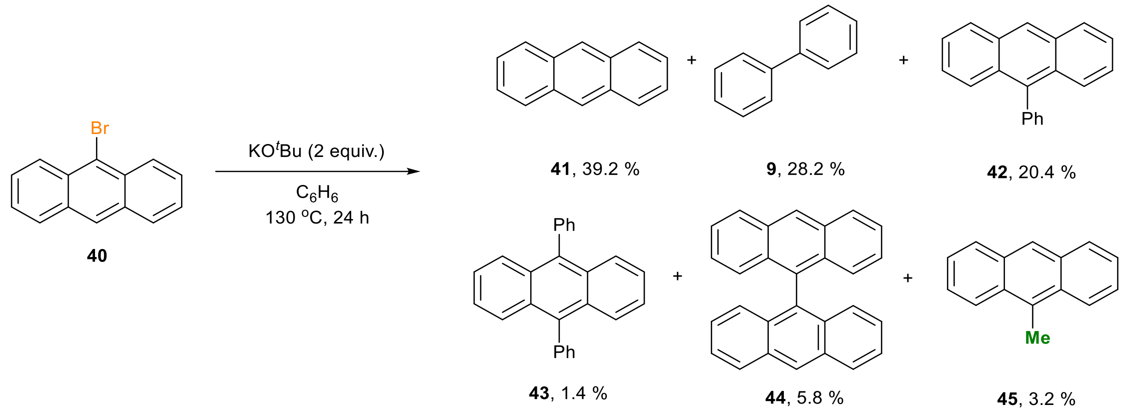

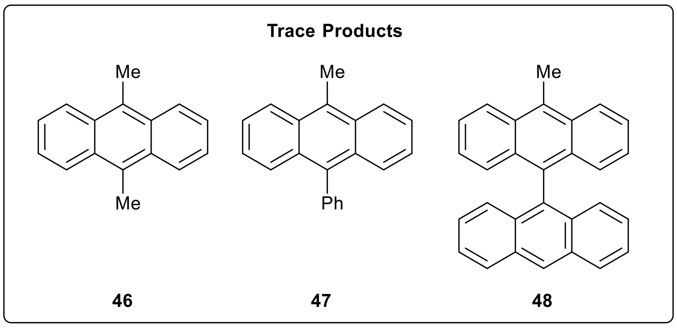

This reaction was carried out three times with the averaged values below (For the individual runs, see page S30-S31):

Sample	%Yield
Biphenyl	28.2
Anthracene	39.2
Methylanthracene	3.2
Phenylanthracene	20.4
Diphenylanthracene	1.4
Bianthracene	5.8

GCMS data

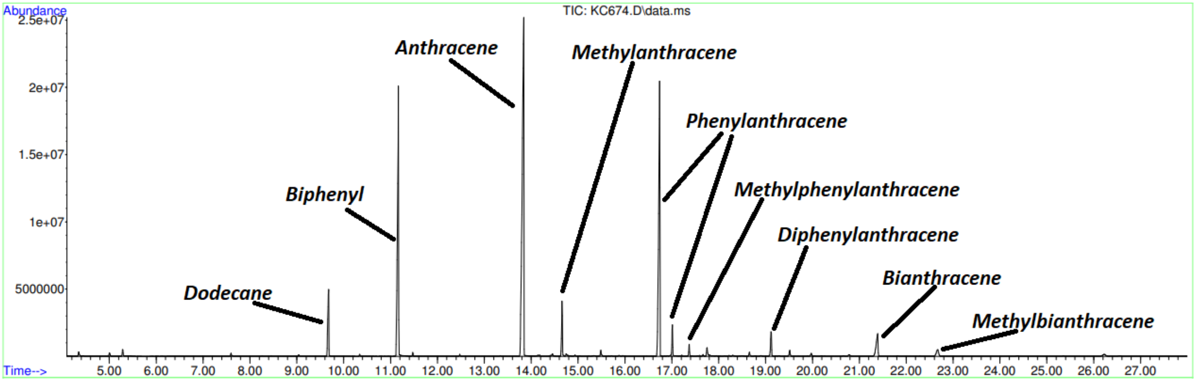

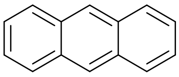

(Calcd.) *m/z*: 178.0783 (100.0%), 179.0816 (15.1%), 180.0850 (1.1%)

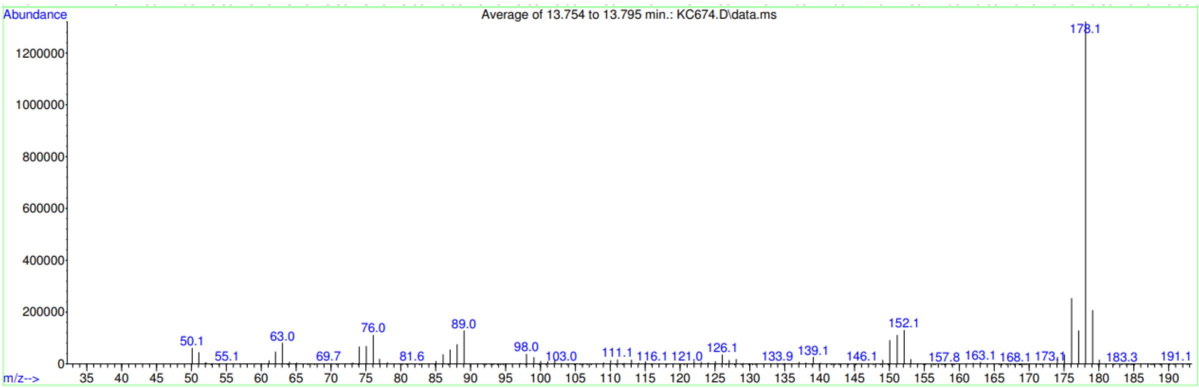

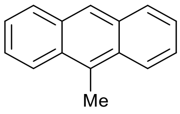

(Calcd.) *m/z:* 192.0939 (100.0%), 193.0973 (16.2%), 194.1006 (1.2%)

9-Methylanthracene - **HRMS** (EI+) [*m/z*] calcd. for $C_{15}H_{12}$ (M^{+}) 192.0939, found 192.0937.

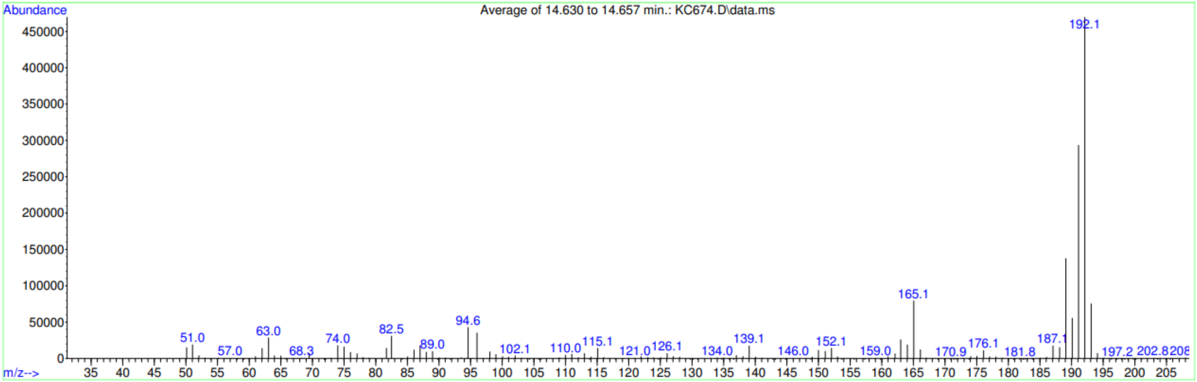

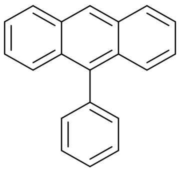

(Calcd.) *m/z:* 254.1096 (100.0%), 255.1129 (21.6%), 256.1163 (2.2%)

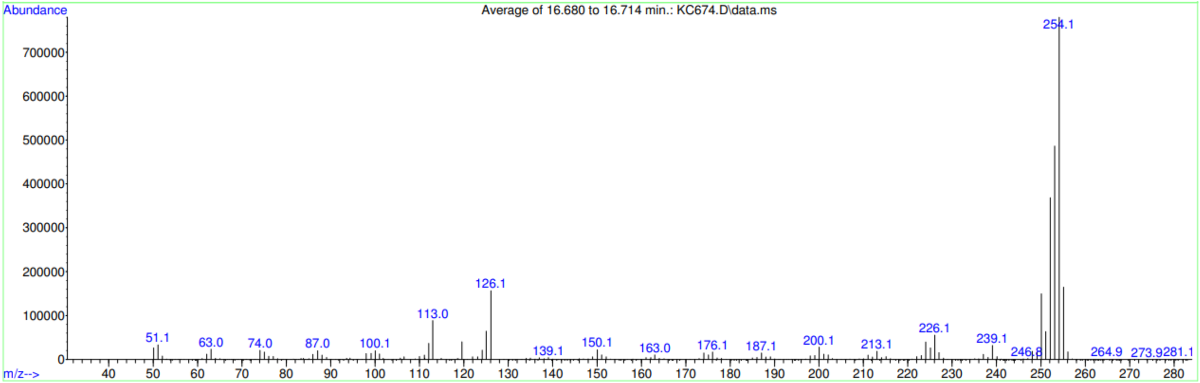

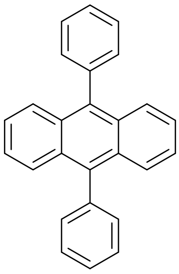

(Calcd.) *m/z:* 330.1409 (100.0%), 331.1442 (28.1%), 332.1476 (3.8%)

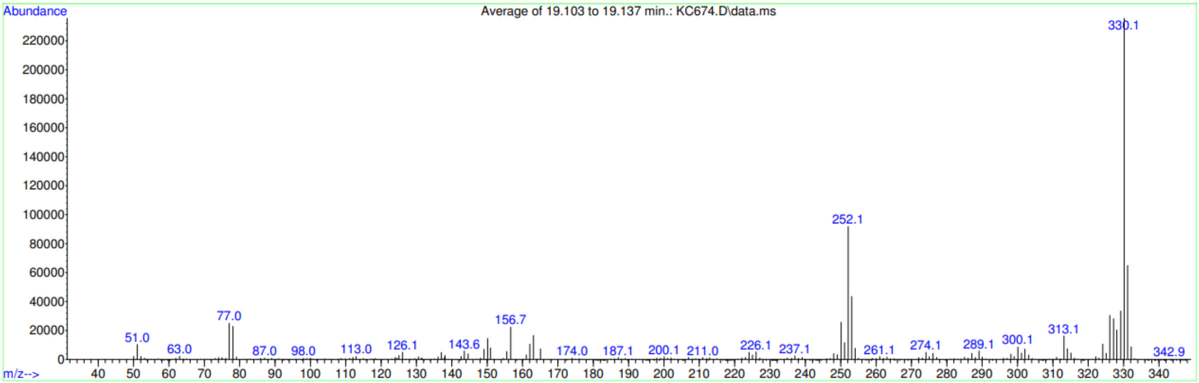

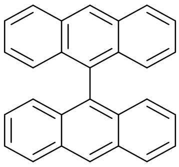

(Calcd.) *m/z*: 354.1409 (100.0%), 355.1442 (30.3%), 356.1476 (4.4%)

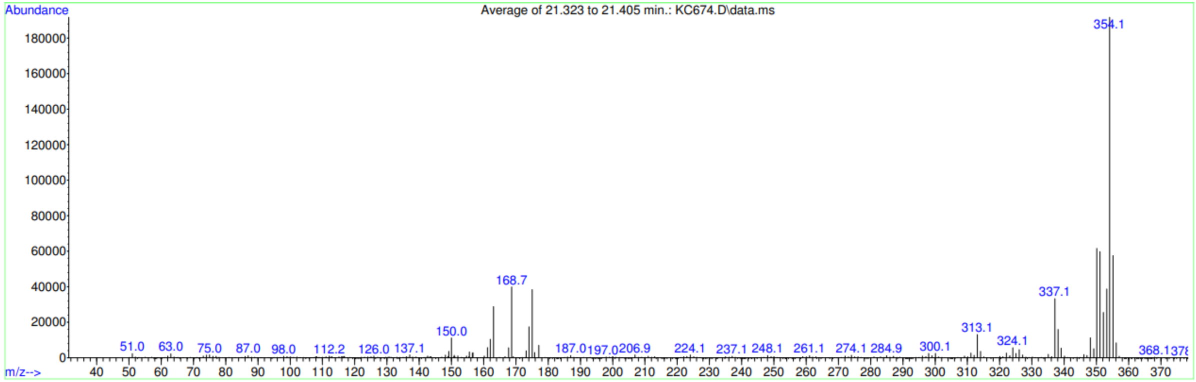

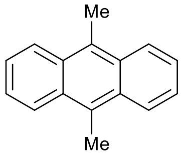

(Calcd.) *m/z*: 206.1096 (100.0%), 207.1129 (17.3%), 208.1163 (1.4%)

9,10-Dimethylanthracene - **HRMS** (EI+) [*m/z*] calcd. for $C_{16}H_{14}$ (M^+) 206.1096, found 206.1086.

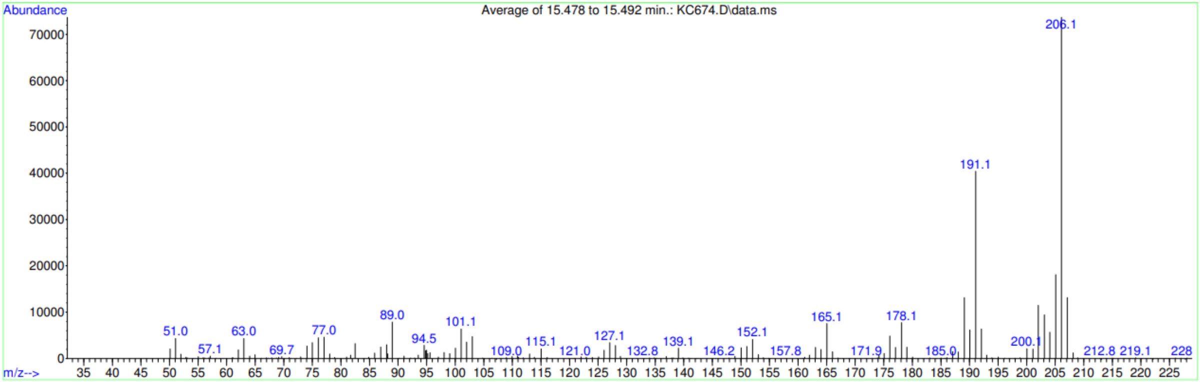

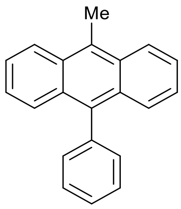

(Calcd.) *m/z*: 268.1252 (100.0%), 269.1286 (22.7%), 270.1319 (2.5%)

Methylphenylanthracene - two isomers found. Isomer 1: **HRMS** (EI+) [*m/z*] calcd. for $C_{21}H_{16}$ (M^+) 268.1252, found 268.1250. Isomer 2: **HRMS** (EI+) [*m/z*] calcd. for $C_{21}H_{16}$ (M^+) 268.1252, found 268.1248.

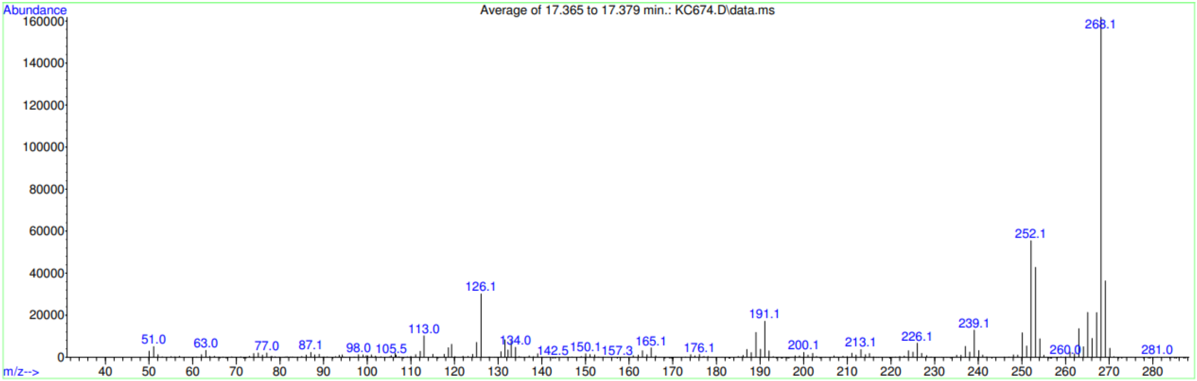

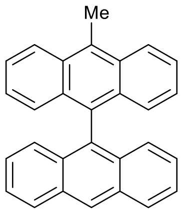

(Calcd.) *m/z*: 368.1565 (100.0%), 369.1599 (31.4%), 370.1632 (4.7%)

9-Methylbianthracene - **HRMS** (EI+) [*m/z*] calcd. for $C_{29}H_{20}$ (M^+) 368.1565, found 368.1570.

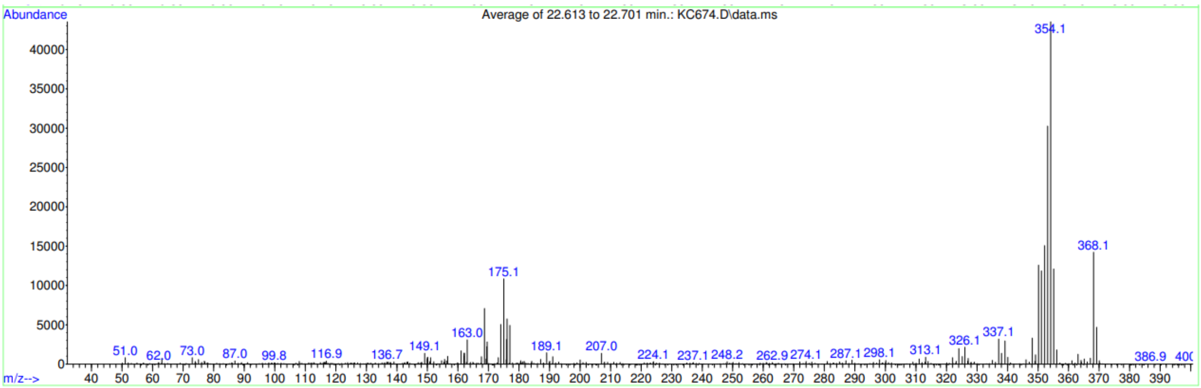

GCFID data including table quantitating components that had been separately calibrated.

Run 1

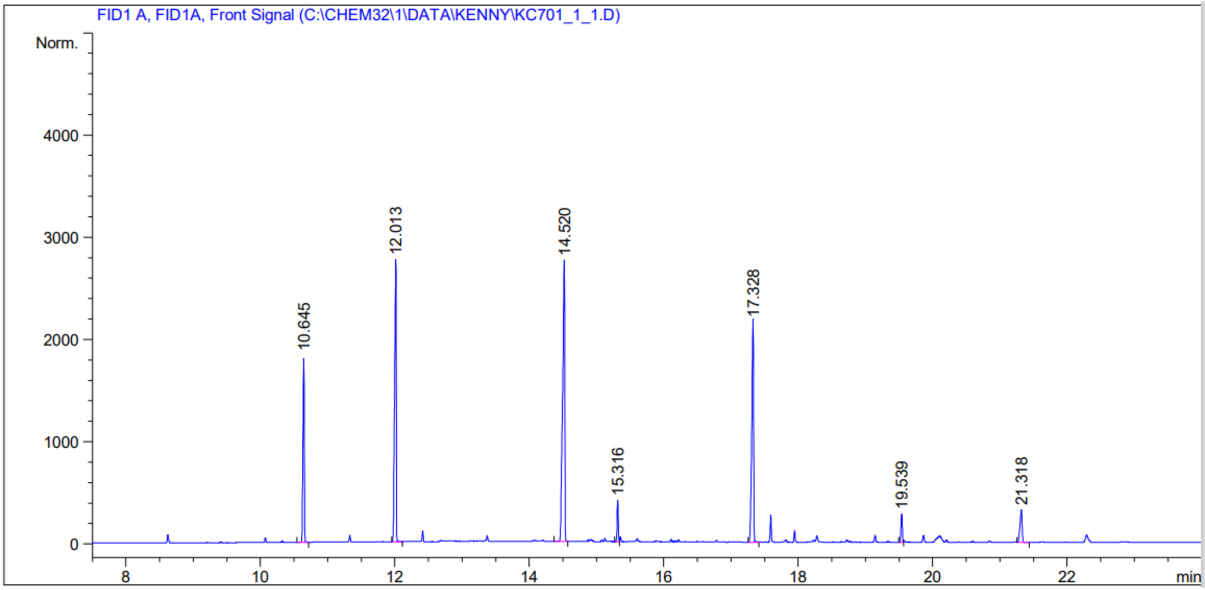

Retention Time (min)	Sample	Peak Area	%Yield
10.645	Dodecane	2341.343	N/A
12.013	Biphenyl	4530.036	28.3
14.520	Anthracene	6177.110	38.5
15.316	Methylanthracene	480.180	3.1
17.328	Phenylanthracene	4081.990	20.6
19.539	Diphenylanthracene	373.586	1.4
21.318	Bianthracene	705.674	5.7

Dodecane added = 15.3 mg

Run 2

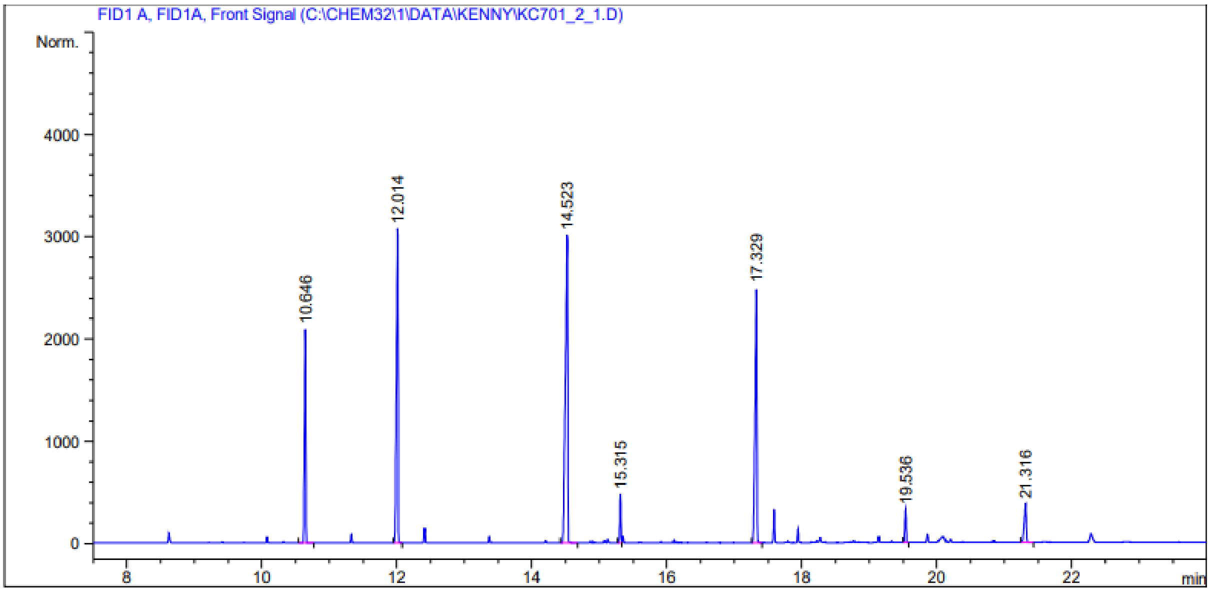

Retention Time (min)	Sample	Peak Area	%Yield
10.646	Dodecane	2763.562	N/A
12.014	Biphenyl	5262.872	27.9
14.523	Anthracene	7455.909	39.4
15.315	Methylanthracene	580.708	3.2
17.329	Phenylanthracene	4785.569	20.5
19.539	Diphenylanthracene	442.646	1.4
21.316	Bianthracene	821.727	5.6

Dodecane added = 15.3 mg

Run 3

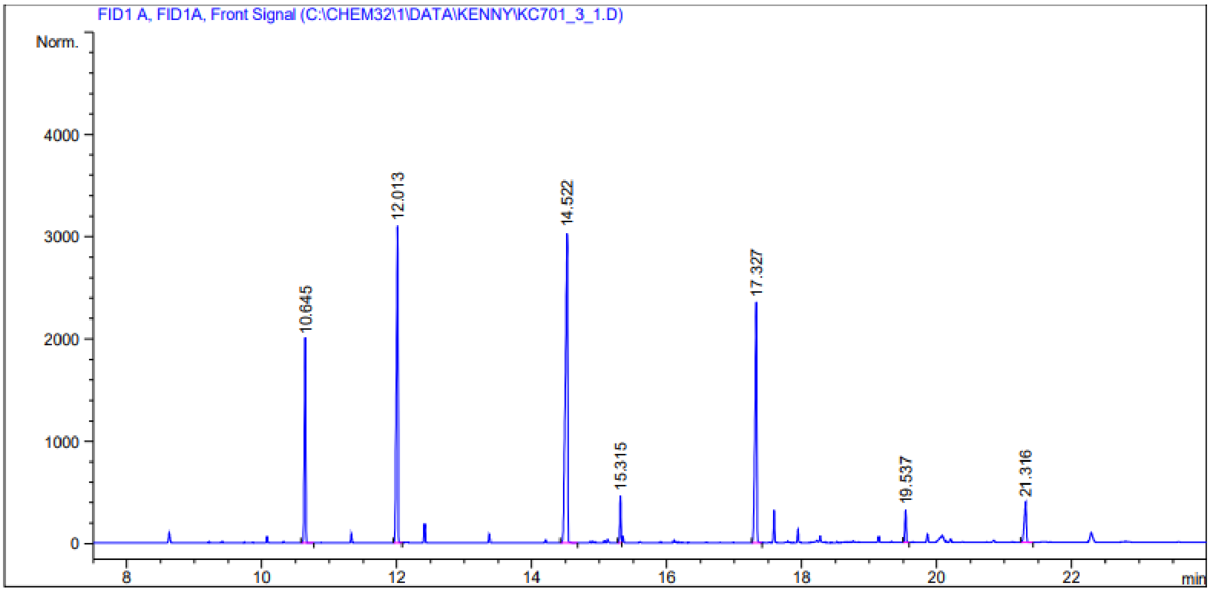

Retention Time (min)	Sample	Peak Area	%Yield
10.645	Dodecane	2707.513	N/A
12.013	Biphenyl	5230.520	28.5
14.522	Anthracene	7314.717	39.7
15.315	Methylanthracene	564.032	3.2
17.327	Phenylanthracene	4562.766	20.0
19.537	Diphenylanthracene	435.345	1.5
21.316	Bianthracene	860.907	6.1

Dodecane added = 15.4 mg

Checking for evidence of isomers of diphenylanthracene in GCMS (for Entry 1 Table 1)

Looking for evidence of *r*-benzynes: Isomers of diphenylanthracene

A reviewer pointed out that if *r*-benzynes were being formed from either 9-bromoanthracene or 9,10-dibromoanthracene, we might expect to see isomers of diphenylanthracene other than the major isomer 9,10-diphenylanthracene **43**.

Looking closely at the baseline of the reaction of 9-bromoanthracene **40** with KO^{t}Bu and C_6H_6 (Table 1, entry 1), we do indeed observe 4 additional peaks with matching masses (*m/z* = 330), indicating that additional isomers of diphenylanthracene are likely produced (see expanded section of GC-MS

trace below, with mass traces of unidentified isomers). These isomers could form through attack of the 4 possible *r*-benzynes onto two benzene rings. Alternatively, they could arise through addition of phenyl radicals onto phenylanthracene (or 9-bromoanthracene, followed by deprotonation, loss of bromide and further attack onto benzene). As the products would be identical, it is not possible to distinguish between the two routes.

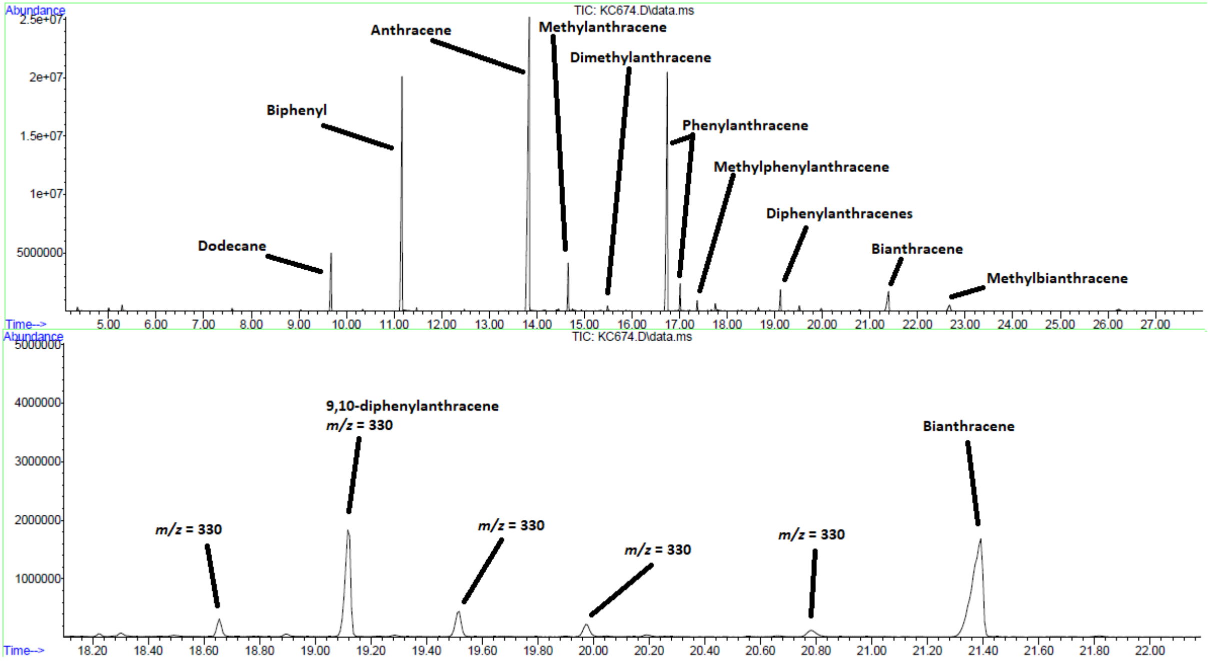

The mass spectrum of the major peak (that due to 9,10-diphenylanthracene) was shown on page S27; below are the spectra of the additional 4 isomers of diphenylanthracene.

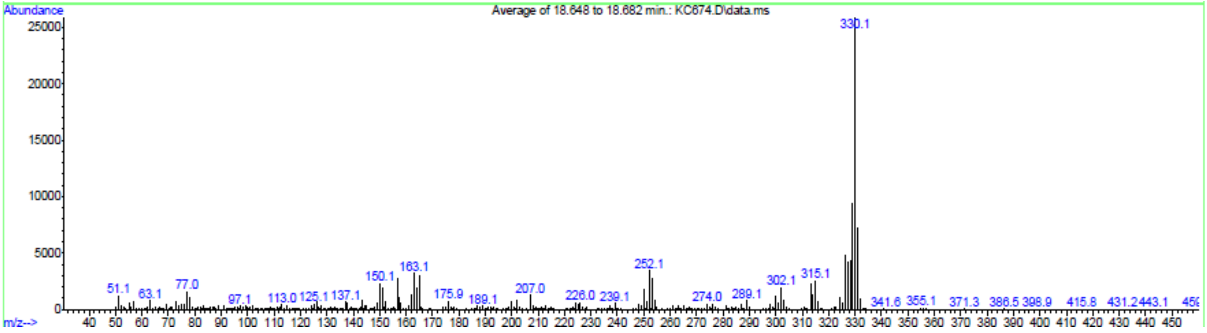

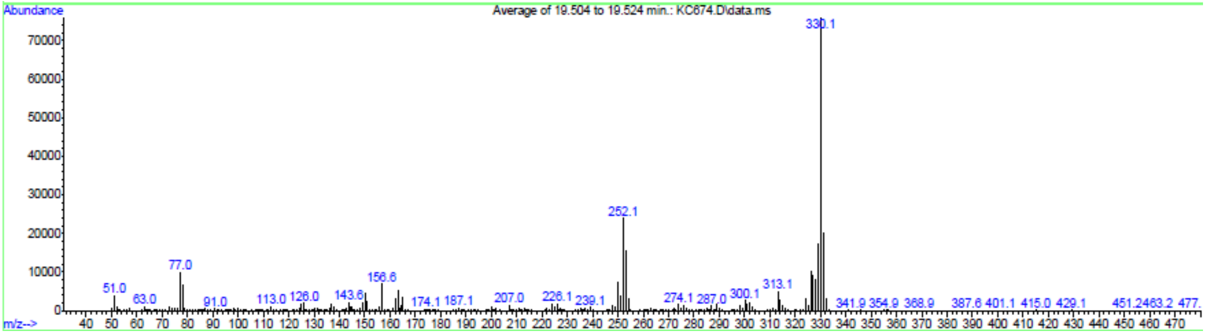

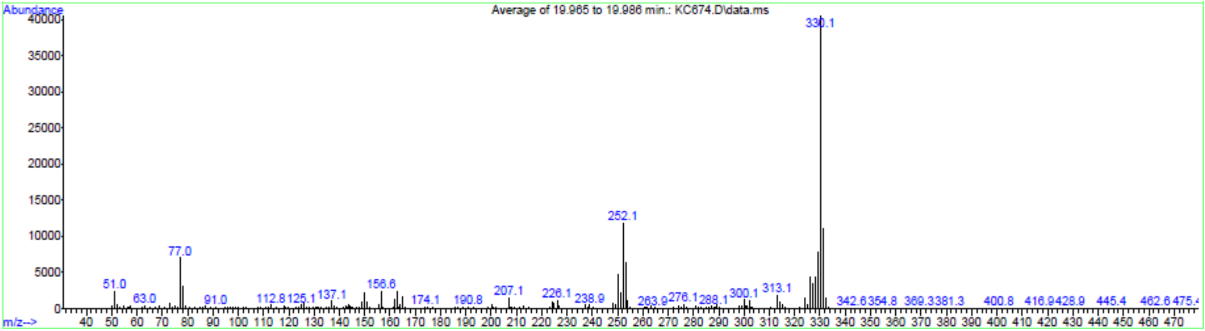

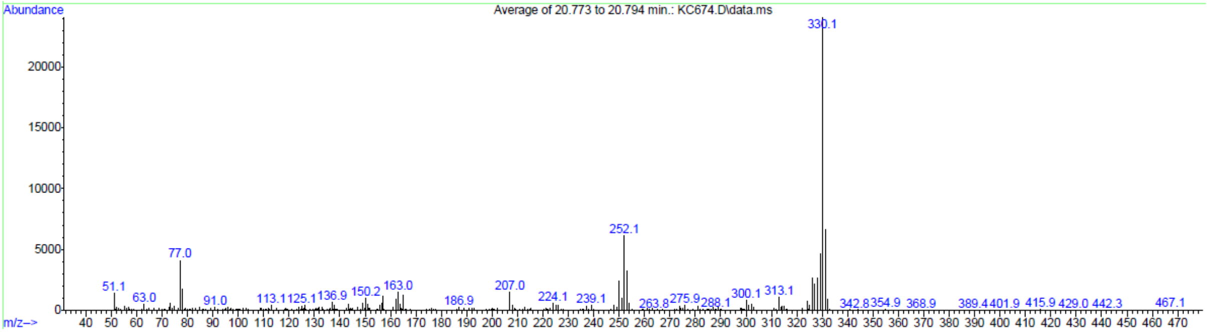

Reaction of 9-bromoanthracene 40 with KO*t*Bu in C_6D_6 (Table 1, Entry 2)

To an oven-dried microwave vial, primed with a stirrer bar, in a glovebox was added 9-bromoanthracene **40** (180 mg, 0.7 mmol, 1 equiv.), KO*t*Bu (157 mg, 1.4 mmol, 2 equiv.) and benzene-d_6 (7 mL) with the vial subsequently sealed and stirred at 130°C in an oil bath for 24 h. Once complete, the crude mixture was allowed to cool to room temperature, H_2O (0.2 mL) and an accurately weighed amount of dodecane was added. An aliquot of the crude mixture was then analysed by both GCMS and GC(FID).

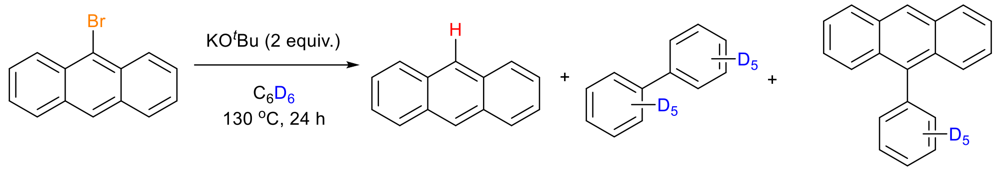

40 **41**, 20.4 % **9-*d*$_{10}$**, 5.2 % **42-*d*$_5$**, 20.1 %

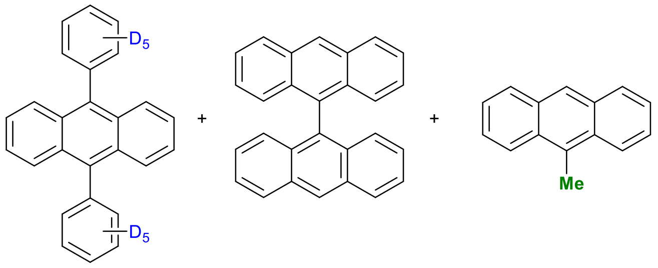

43-*d*$_{10}$, 0.4 % **44**, 6.8 % **45**, 6.2 %

This reaction was carried out in duplicate with the average below (for the individual runs, see pages S35-S36):

Sample	%Yield
Biphenyl	5.2
Anthracene	20.4
Methylanthracene	6.2
Phenylanthracene	20.1
Diphenylanthracene	0.4
Bianthracene	6.8

GCMS Data

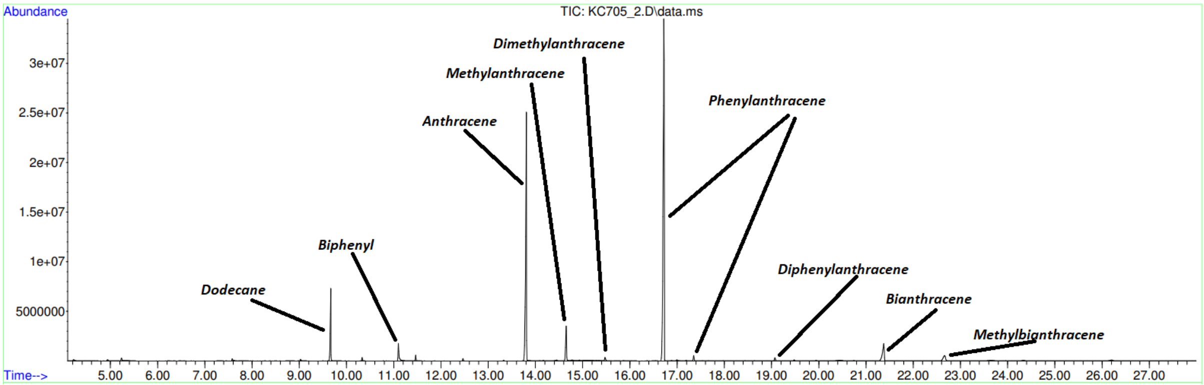

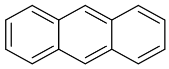

(Calcd.) *m/z*: 178.0783 (100.0%), 179.0816 (15.1%), 180.0850 (1.1%)

GC-MS shows that in this experiment anthracene in predominantly not deuterated.

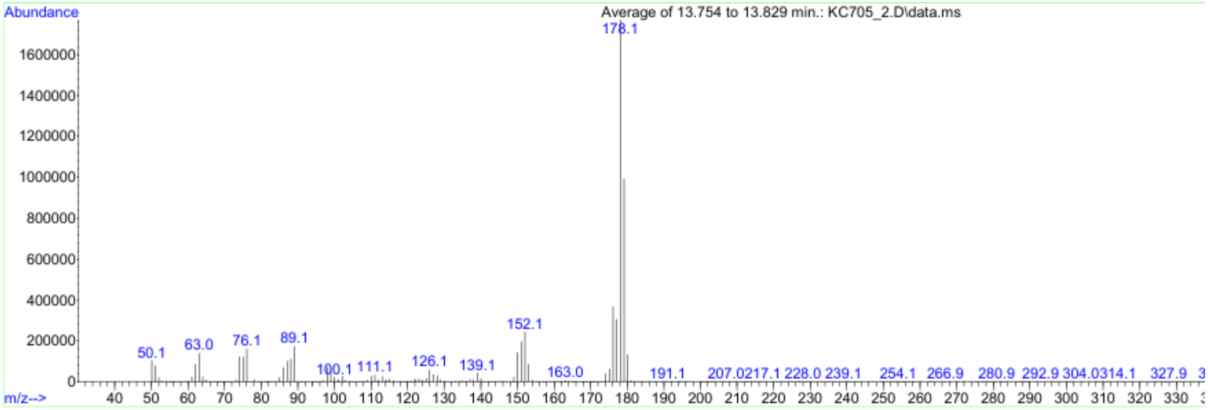

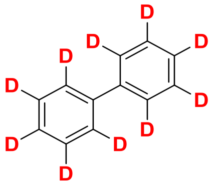

(Calcd.) *m/z*: 164.1410 (100.0%), 165.1444 (13.0%)

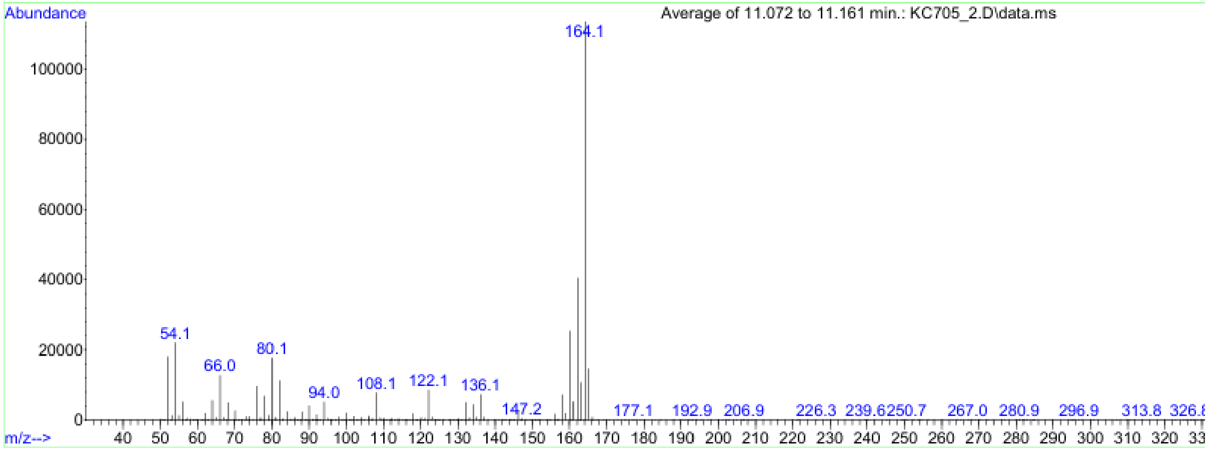

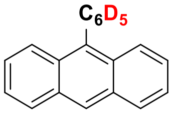

(Calcd.) *m/z*: 259.1409 (100.0%), 260.1443 (21.6%), 261.1476 (2.2%)

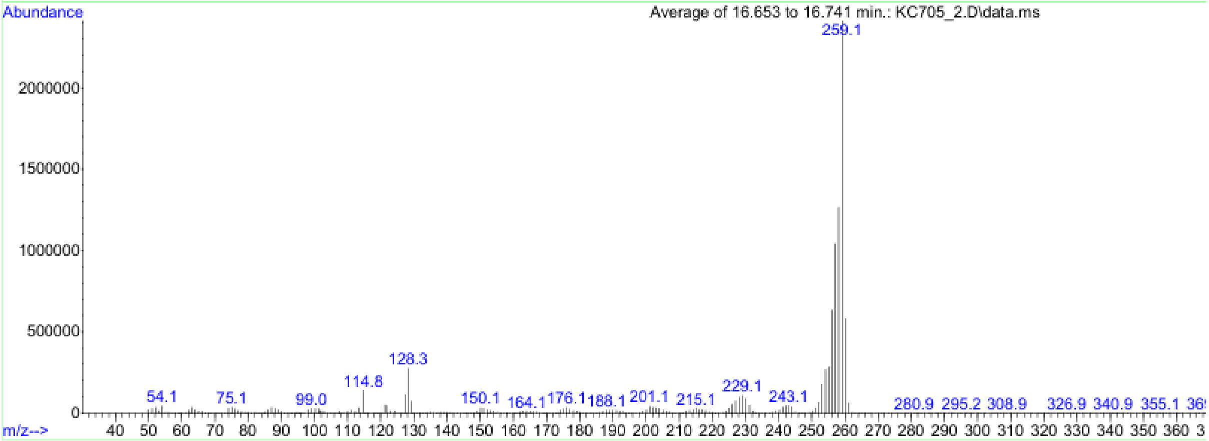

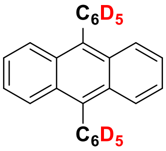

(Calcd.) *m/z*: 340.2036 (100.0%), 341.2070 (28.1%), 342.2103 (3.8%)

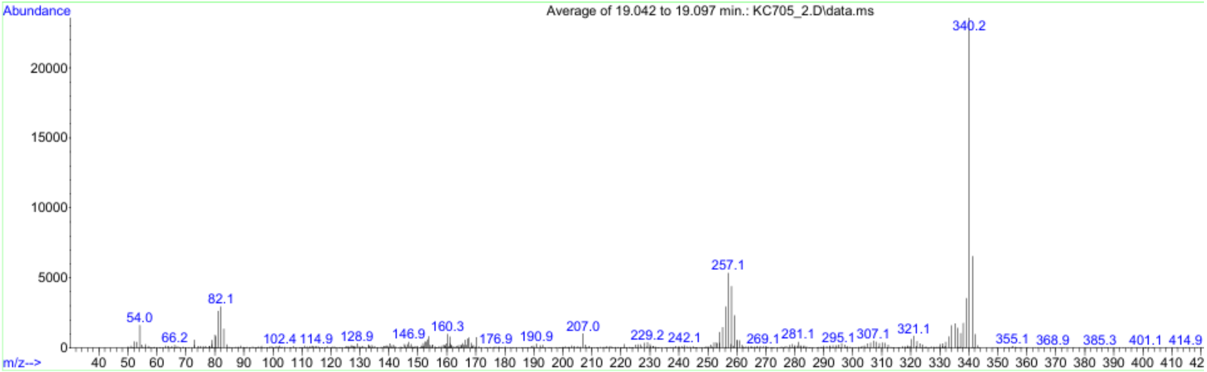

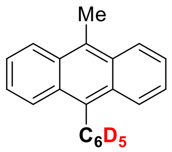

(Calcd.) *m/z*: 273.1566 (100.0%), 274.1599 (22.7%), 275.1633 (2.5%)

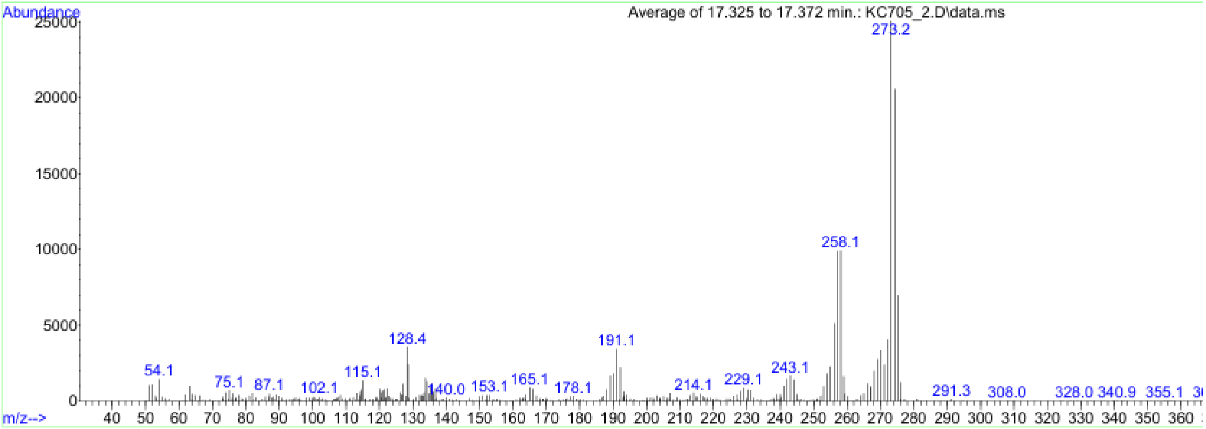

GCFID data including table quantitating components that had been separately calibrated.

Run 1

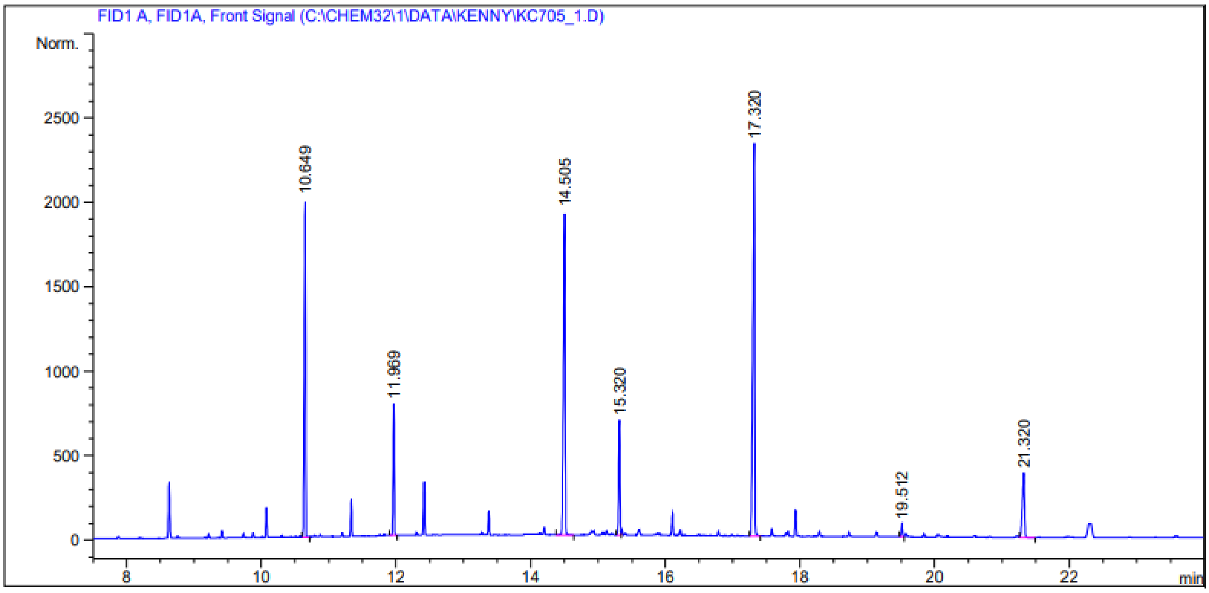

Retention Time	Sample	Peak Area	%Yield
10.649	Dodecane	2566.996	N/A
11.969	Biphenyl	892.161	5.2
14.505	Anthracene	3457.302	19.9
15.320	Methylanthracene	952.230	5.8
17.320	Phenylanthracene	4383.320	20.4
19.512	Diphenylanthracene	101.695	0.4
21.320	Bianthracene	875.744	6.5

Dodecane added = 15.5 mg

Run 2

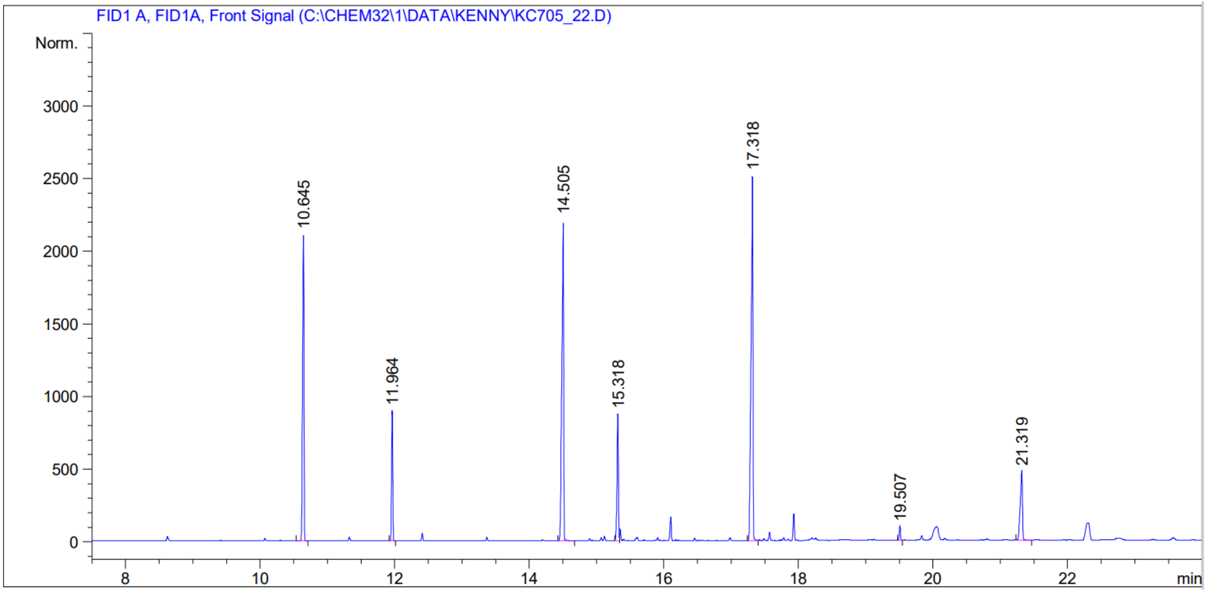

Retention Time	Sample	Peak Area	%Yield
10.645	Dodecane	2909.375	N/A
11.964	Biphenyl	1059.438	5.2
14.505	Anthracene	4238.967	20.8
15.318	Methylanthracene	1271.865	6.6
17.318	Phenylanthracene	4964.584	19.8
19.507	Diphenylanthracene	126.380	0.4
21.319	Bianthracene	1090.66	7.0

Dodecane added = 15.0 mg

Reaction of 9-bromoanthracene 40 with KO*t*Bu-d_9 in C_6H_6 (Table 1, Entry 3)

To an oven-dried microwave vial, primed with a stirrer bar, in a glovebox was added 9-bromoanthracene **40** (180 mg, 0.7 mmol, 1 equiv.), KO*t*Bu-d_9 (169 mg, 1.4 mmol, 2 equiv.) and benzene (7 mL) with the vial subsequently sealed and stirred at 130°C in an oil bath for 24 h. Once complete, the crude mixture was allowed to cool to room temperature, H_2O (0.2 mL) and an accurately weighed amount of dodecane was added. An aliquot of the crude mixture was then analysed by both GCMS and GC-FID (Method 1).

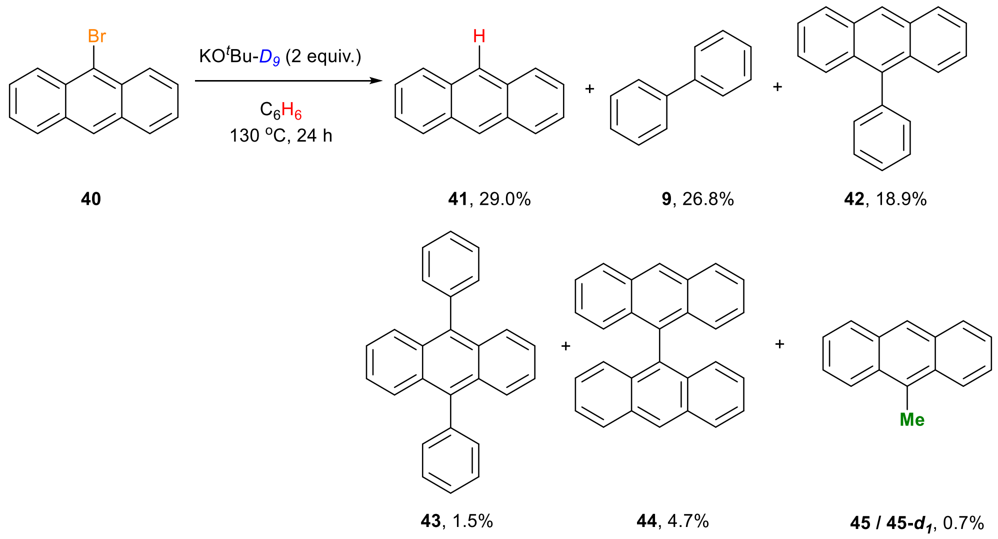

GCMS Data

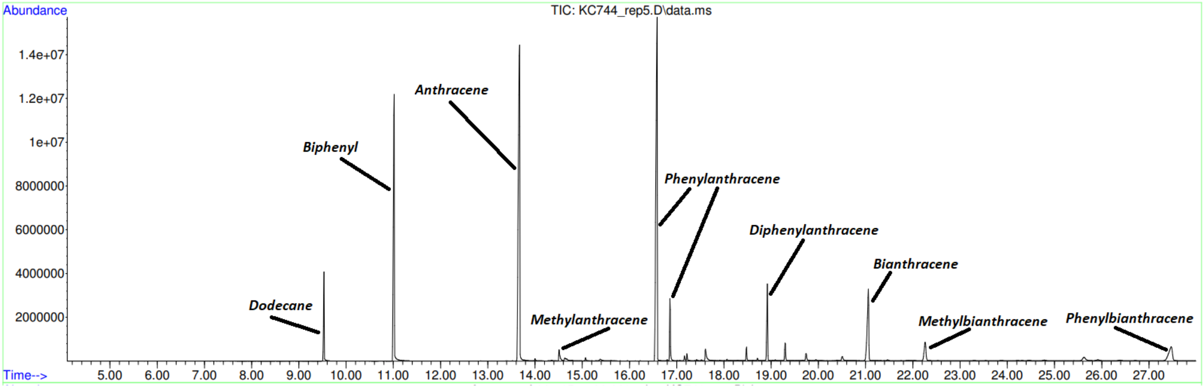

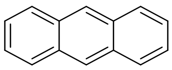

(Calcd.) *m/z*: 178.0783 (100.0%), 179.0816 (15.1%), 180.0850 (1.1%)

GC-MS shows that in this experiment anthracene is not deuterated.

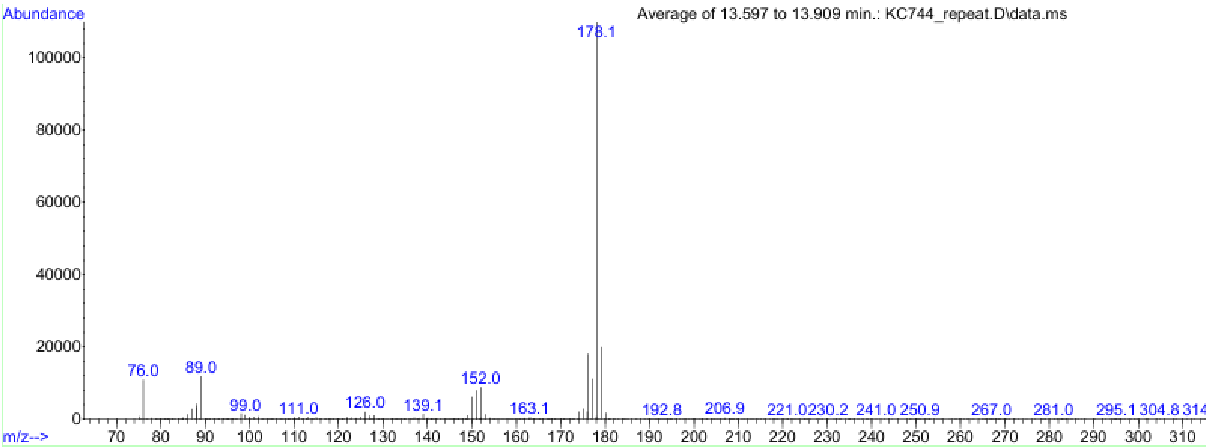

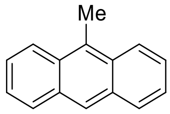

(Calcd.) *m/z:* 192.0939 (100.0%), 193.0973 (16.2%), 194.1006 (1.2%)

GC-MS shows only slight deuterium incorporation, due to exchange on the methyl group.

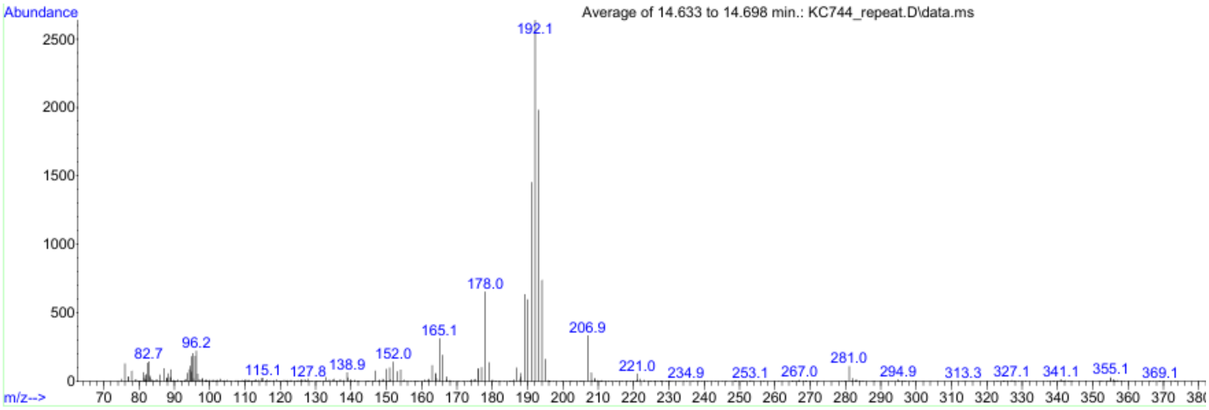

GCFID data including table quantitating components that had been separately calibrated.

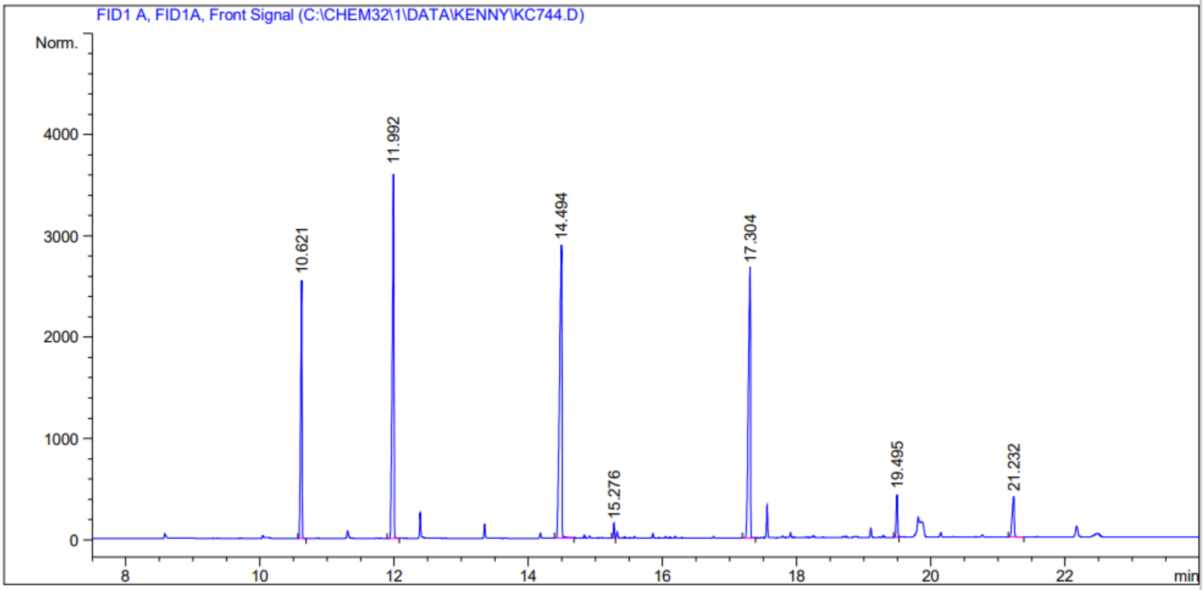

Retention Time	Sample	Peak Area	%Yield
10.621	Dodecane	3643.296	N/A
11.992	Biphenyl	6504.314	26.8%
14.494	Anthracene	7063.663	29.0%
15.276	Methylanthracene	170.462	0.7%
17.304	Phenylanthracene	5675.977	18.9%
19.495	Diphenylanthracene	582.722	1.5%
21.232	Bianthracene	877.569	4.7%

Dodecane added = 15.7 mg

Reaction of 9-bromoanthracene with KO*t*Bu-d_9 in C_6D_6 (Table 1, Entry 4)

To an oven-dried microwave vial, primed with a stirrer bar, in a glovebox was added 9-bromoanthracene **40** (180 mg, 0.7 mmol, 1 equiv.), KO*t*Bu-d_9 (169 mg, 1.4 mmol, 2 equiv.) and benzene-d_6 (7 mL) with the vial subsequently sealed and stirred at 130°C in an oil bath for 24 h. Once complete, the crude mixture was allowed to cool to room temperature, H_2O (0.2 mL) and an accurately weighed amount of dodecane in was added. An aliquot of the crude mixture was then analysed by both GCMS and GC-FID (Method 1).

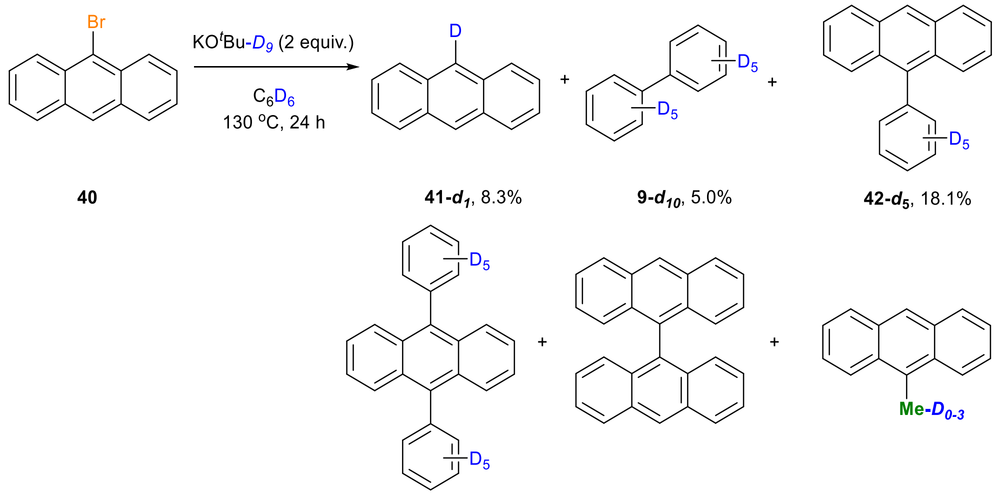

40 **41-d_1**, 8.3% **9-d_{10}**, 5.0% **42-d_5**, 18.1% **43-d_{10}**, 0.4% **44**, 3.8% **45-d_{0-3}**, 2.5%

GCMS Data

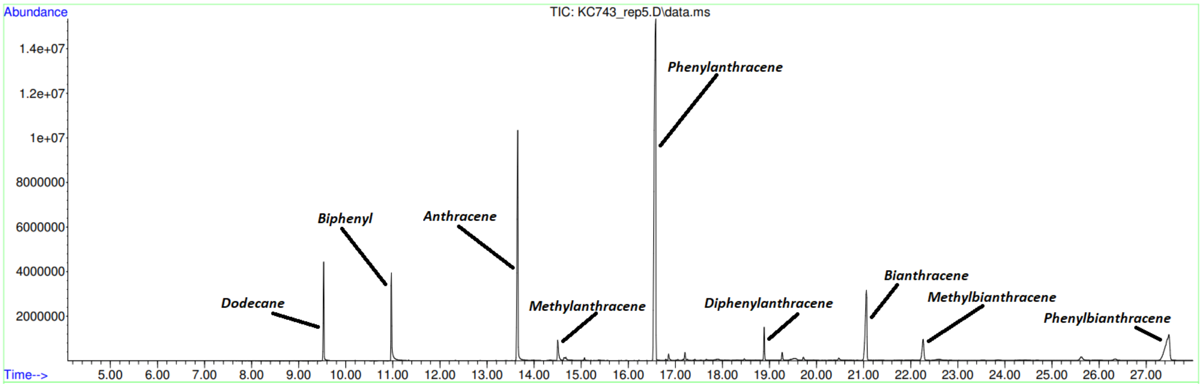

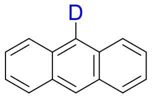

41-d_1

(Calcd.) *m/z*: 179.0845 (100.0%), 180.0879 (15.1%), 181.0912 (1.1%)

Retention Time = ~13.7 min

GC-MS shows that in this experiment the majority of anthracene **41** is mono-deuterated

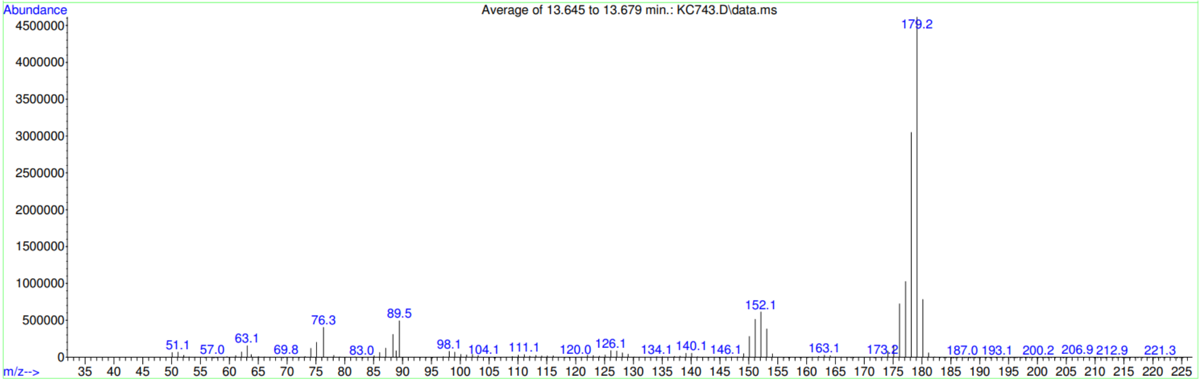

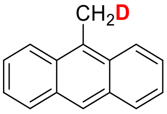

(Calcd.) *m/z*: 193.1002 (100.0%), 194.1035 (16.2%), 195.1069 (1.2%)

GC-MS shows only partial deuterium incorporation, due to exchange on the methyl group with KO*t*Bu.

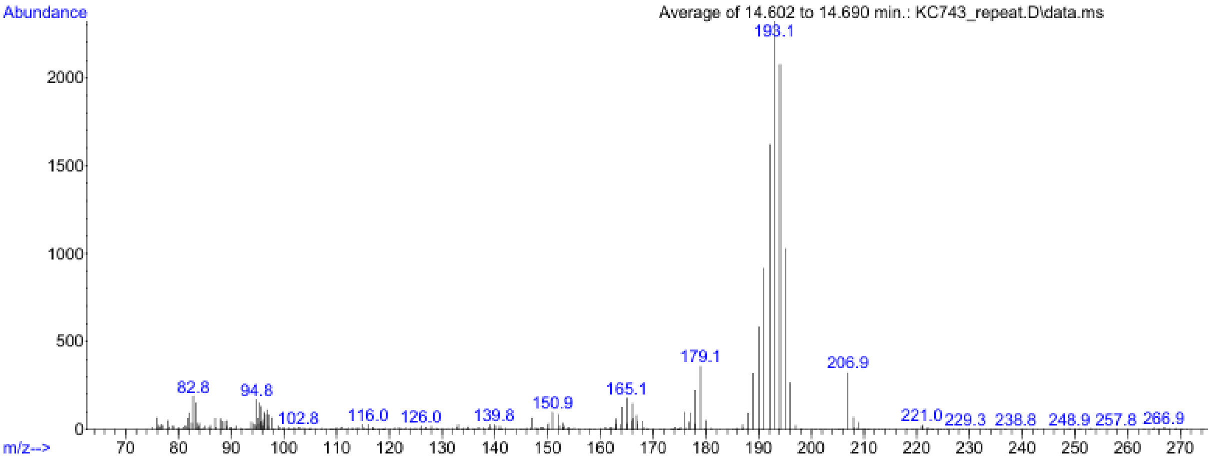

GCFID data including table quantitating components that had been separately calibrated.

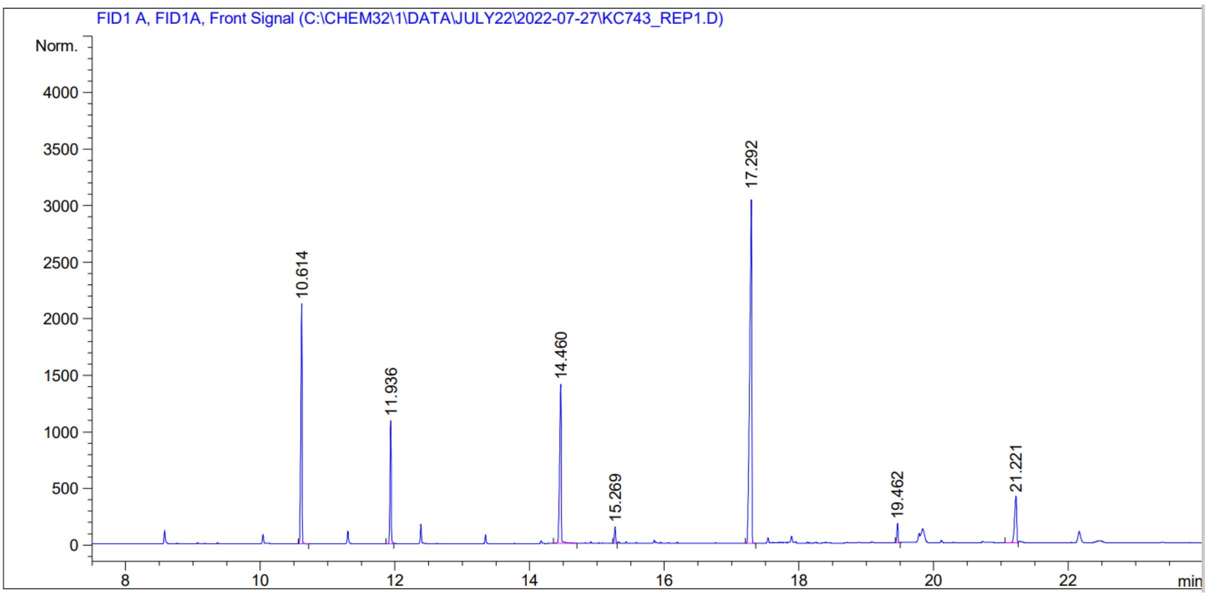

Retention Time	Sample	Peak Area	%Yield
10.614	Dodecane	3931.474	N/A
11.936	Biphenyl	1372.440	5.0%
14.460	Anthracene	2272.070	8.3%
15.269	Methylanthracene	652.851	2.5%
17.292	Phenylanthracene	6094.060	18.1%
19.462	Diphenylanthracene	196.470	0.4%
21.221	Bianthracene	804.801	3.8%

Dodecane added = 15.1 mg

Reaction of 9-bromoanthracene 40 with KO*t*Bu in C_6H_6 in the dark

To see whether ambient light may be causing the initiation, the reaction was repeated with a reaction vial covered in foil to block out light. The results are very similar to parent conditions, so it can be concluded that light is not required to initiate the reactions.

To an oven-dried microwave vial, primed with a stirrer bar, covered in aluminium foil, in a glovebox was added 9-bromoanthracene **40** (180 mg, 0.7 mmol, 1 equiv.), KO*t*Bu (157 mg, 1.4 mmol, 2 equiv.) and benzene (7 mL) with the vial subsequently sealed and stirrer at 130 °C for 24 h. Once complete, the crude mixture was allowed to cool to room temperature, H_2O (0.2 mL) and an accurately weighed amount of dodecane was added. An aliquot of the crude mixture was then analysed by both GCMS and GC-FID (Method 1).

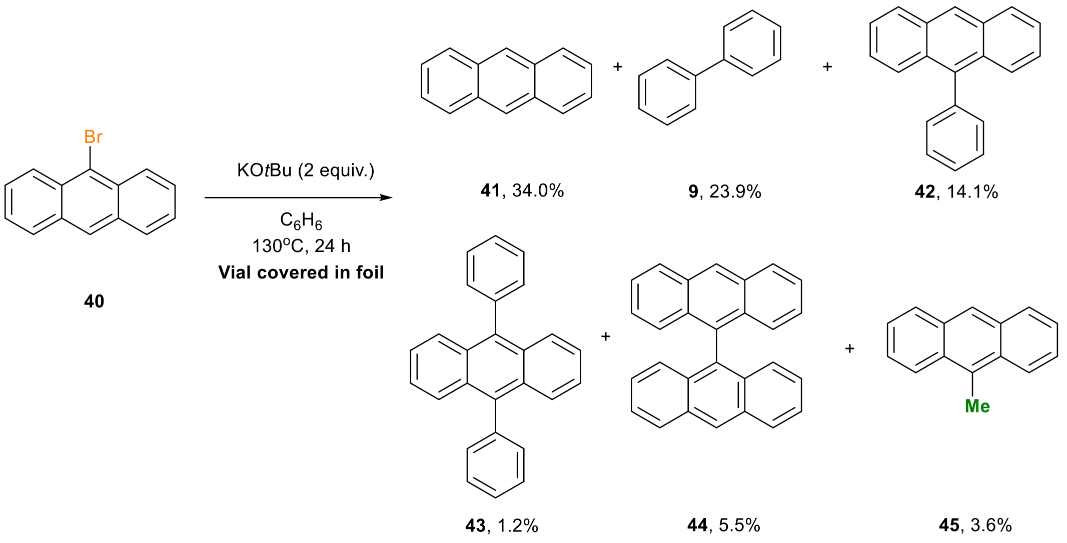

GCMS data

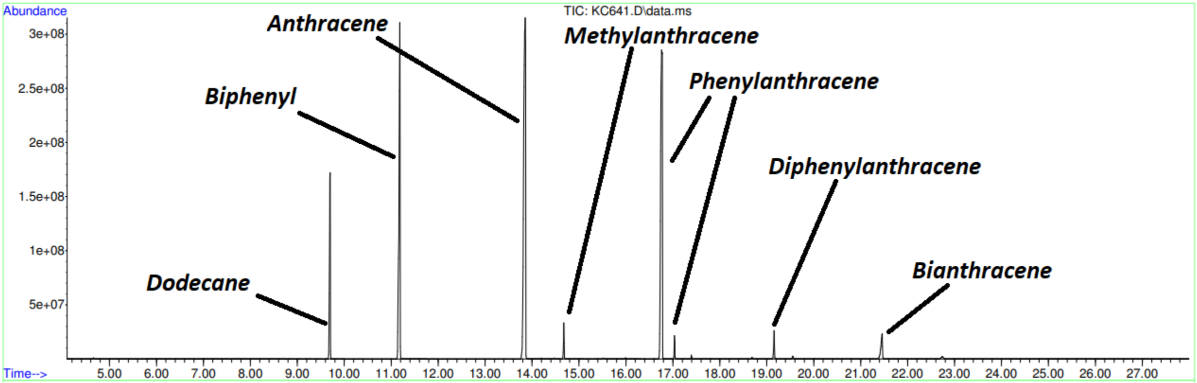

GC-FID data including table quantitating components that had been separately calibrated.

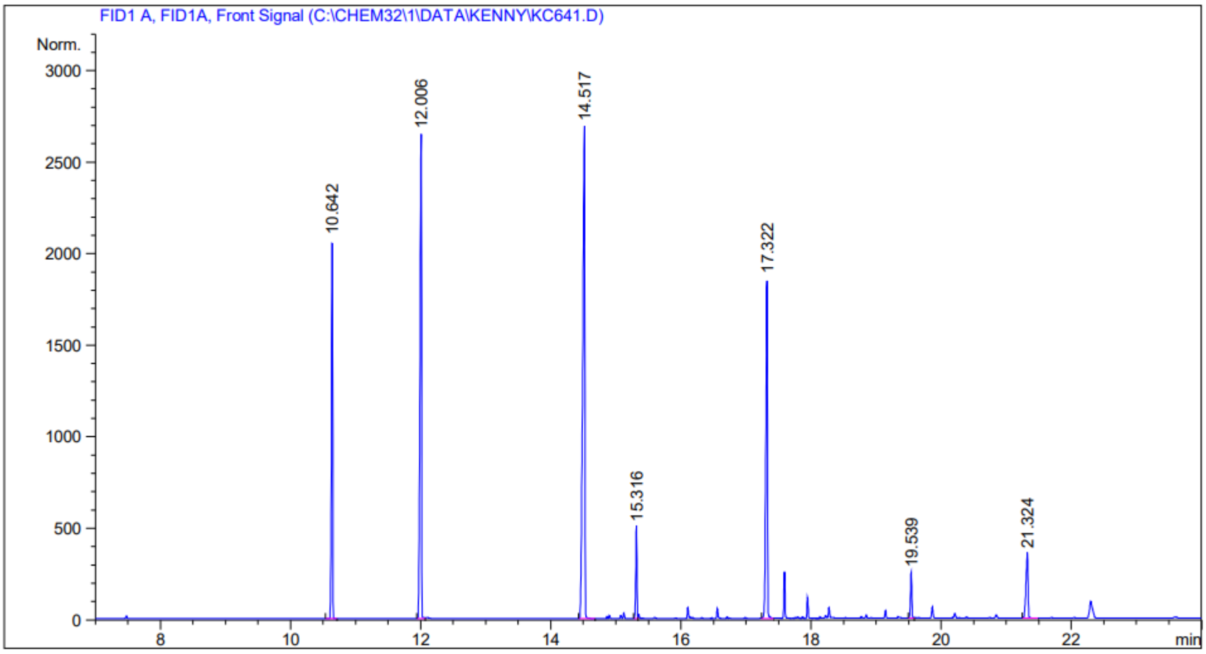

Retention Time (min)	Sample	Peak Area	%Yield
10.642	Dodecane	2760.46924	N/A
12.006	Biphenyl	4301.29785	23.9%
14.517	Anthracene	6151.16943	34.0%
15.316	Methylanthracene	622.14691	3.6%
17.322	Phenylanthracene	3150.11792	14.1%
19.539	Diphenylanthracene	342.32629	1.2%
21.324	Bianthracene	768.19708	5.5%

Dodecane added = 16.0 mg

Reaction of 9-bromoanthracene 40 with KO*t*Bu (99.99%) in C_6H_6

This reaction was carried out to check whether an ultra-pure KO*t*Bu (99.99%) would behave differently than our normal commercial '97%' KO*t*Bu (Alfa Aesar; actual purity of 99.1% was recorded in the assay of this batch by the commercial supplier). Thus, the highest grade KO*t*Bu available (Sigma Aldrich sublimed grade, 99.99% trace metal basis) was used. The results between the 97% KO*t*Bu and 99.99% KO*t*Bu were very similar.

To an oven-dried microwave vial, primed with a stirrer bar, in a glovebox was added 9-bromoanthracene **40** (180 mg, 0.7 mmol, 1 equiv.), 99.99% KO*t*Bu (157 mg, 1.4 mmol, 2 equiv.) and benzene (7 mL) with the vial subsequently sealed and stirred at 130°C in an oil bath for 24 h. Once complete, the crude mixture was allowed to cool to room temperature, H_2O (0.2 mL) and dodecane (14.4 mg) in EtOAc (5 mL) was added. An aliquot of the crude mixture was then analysed by both GCMS and GC-FID (Method 2). In this experiment, a trace peak corresponding to anthrylacetone **S1** was also observed.

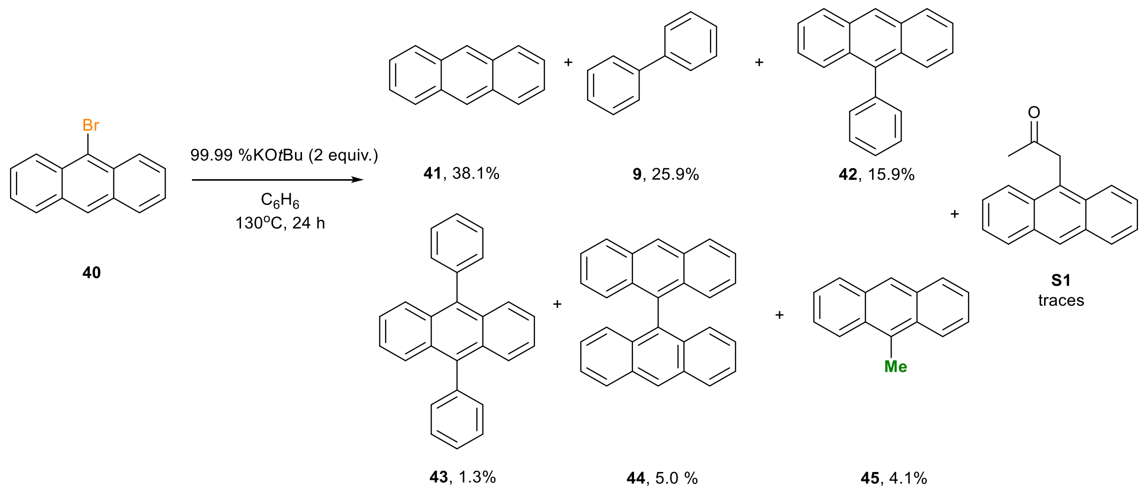

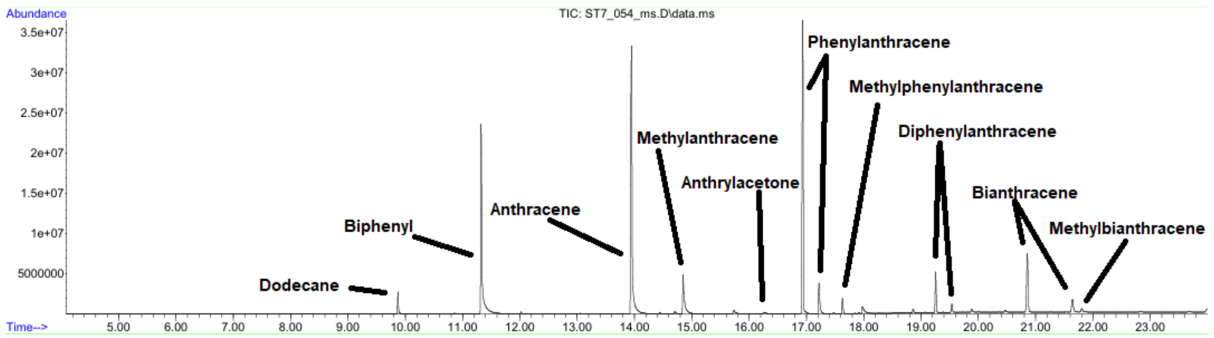

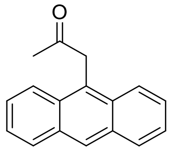

(Calcd.) *m/z*: 234.10 (100.0%), 235.11 (18.6%), 236.11 (1.8%)

Anthrylacetone **S1** - **HRMS** (ESI+) [*m/z*] calcd. for $C_{17}H_{15}O$ (M+H$^+$) 235.1117, found 235.1115.

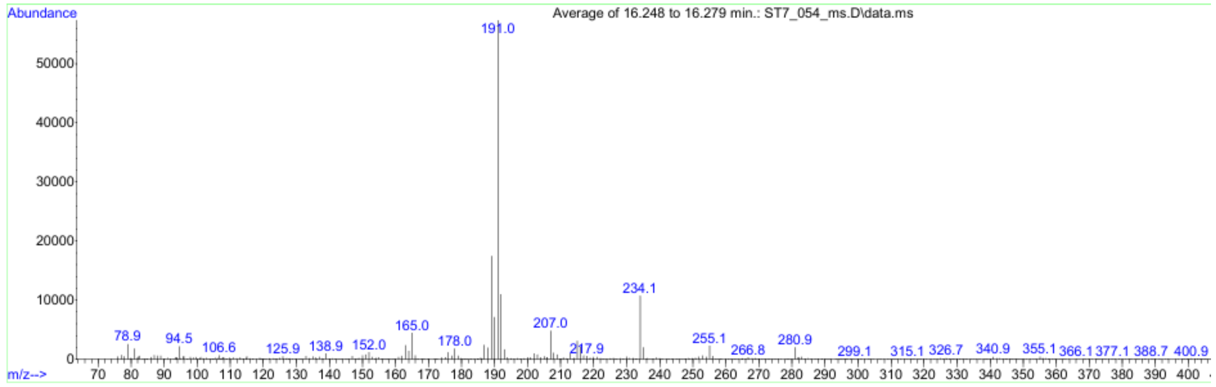

GC-FID data including table quantitating components that had been separately calibrated.

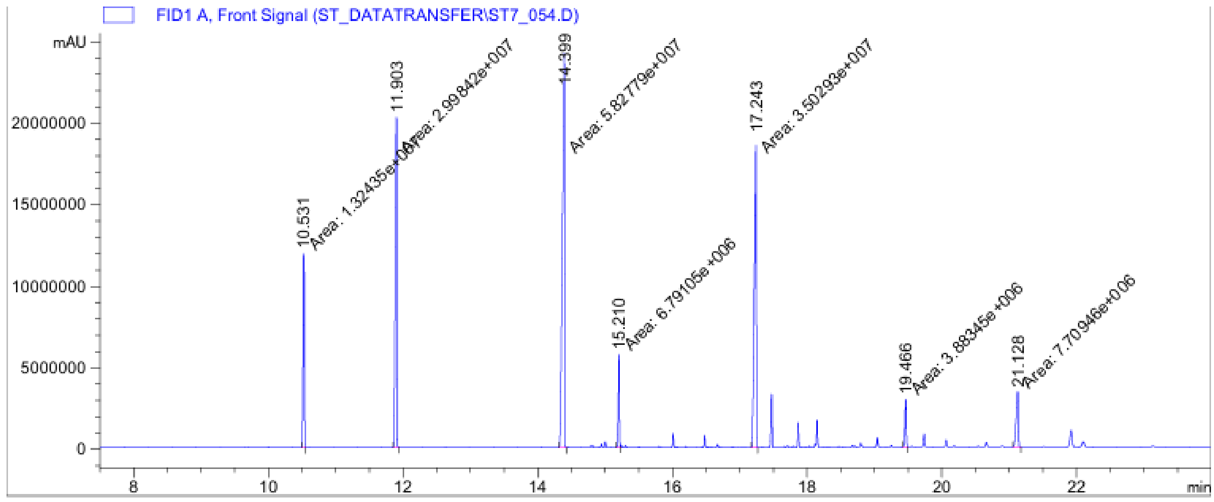

Retention Time (min)	Sample	Peak Area	%Yield
10.531	Dodecane	13243500	N/A
11.903	Biphenyl	29984200	25.9
14.399	Anthracene	58277900	38.1
15.210	Methylanthracene	6791050	4.1
17.243	Phenylanthracene	35029300	15.9
19.466	Diphenylanthracene	3883450	1.3
21.128	Bianthracene	7709460	5.0

Dodecane added = 14.4 mg

Control reaction of 9-bromoanthracene in C_6H_6 (no KO*t*Bu)

To an oven-dried microwave vial, primed with a stirrer bar, in a glovebox was added 9-bromoanthracene **40** (180 mg, 0.7 mmol, 1 equiv.), and benzene (7 mL) with the vial subsequently sealed and stirred at 130 °C in an oil bath for 24 h. Once complete, the crude mixture was allowed to cool to room temperature, H_2O (0.2 mL) and dodecane (14.9 mg) was added. An aliquot of the crude mixture was then analysed by both GCMS and GC-FID (Method 1). The data show that no reaction has occurred.

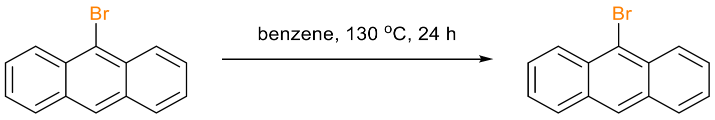

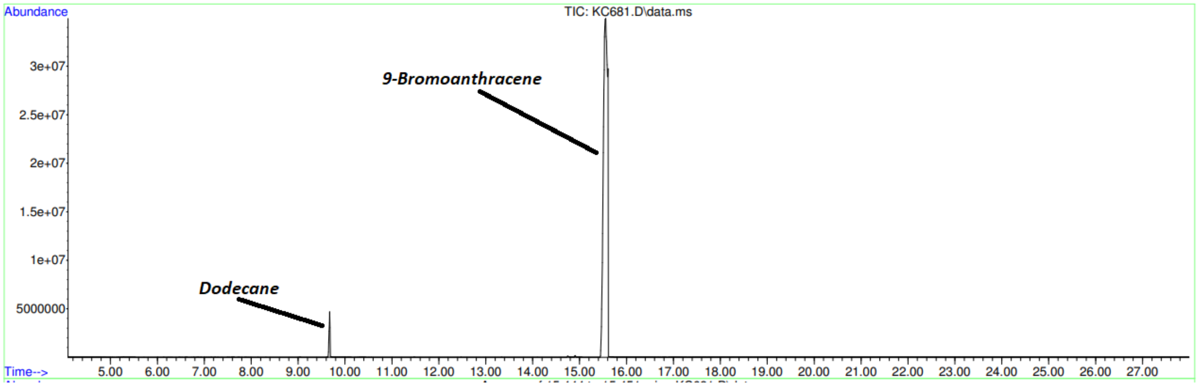

GCFID data including table quantitating components that had been separately calibrated.

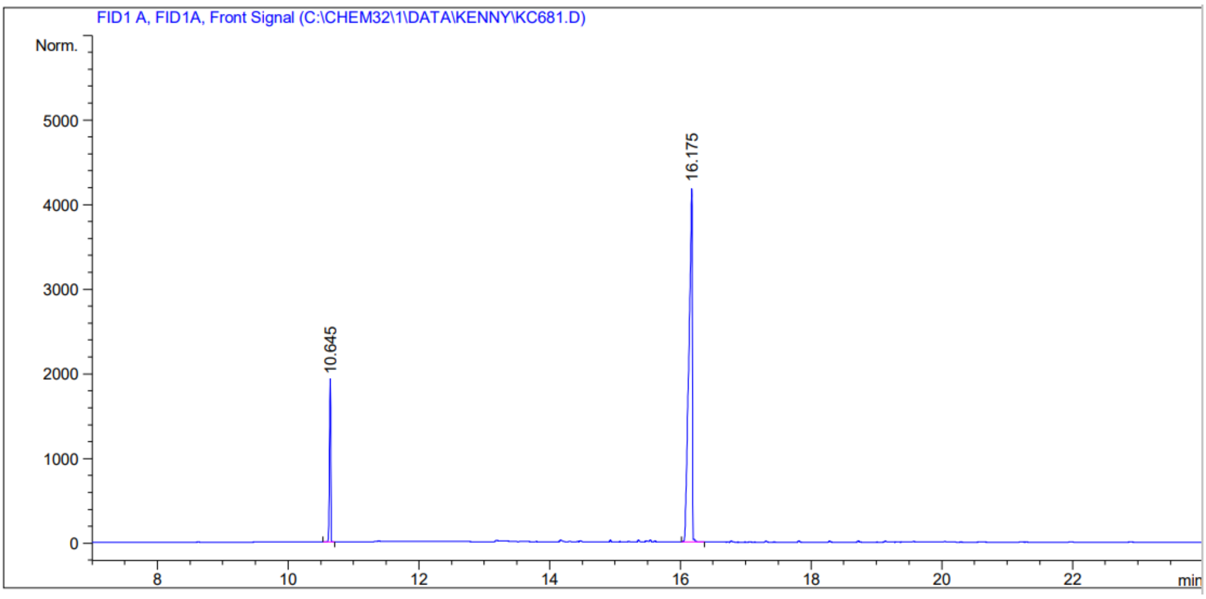

Retention Time (min)	Sample	Peak Area	%Yield
10.645	Dodecane	2619.62939	N/A
16.175	9-Bromoanthracene	15238.7	99.1%

Dodecane added = 14.9 mg

Reaction of 9-bromoanthracene 40 with NaO*t*Bu

In this experiment, KO*t*Bu was swapped for NaO*t*Bu, and no reaction was observed.

To an oven-dried microwave vial, primed with a stirrer bar, in a glovebox was added 9-bromoanthracene **40** (180 mg, 0.7 mmol, 1 equiv.), NaO*t*Bu (135 mg, 1.4 mmol, 2 equiv.) and benzene (7 mL) with the vial subsequently sealed and stirred at 130°C in an oil bath for 24 h. Once complete, the crude mixture was allowed to cool to room temperature, H_2O (0.2 mL) and dodecane (15.9 mg) in was added. An aliquot of the crude mixture was then analysed by both GCMS and GC-FID (Method 1).

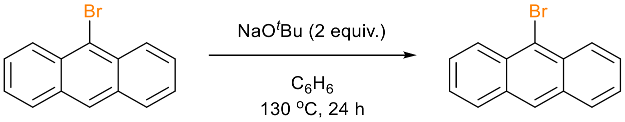

40 **40**

GCMS data

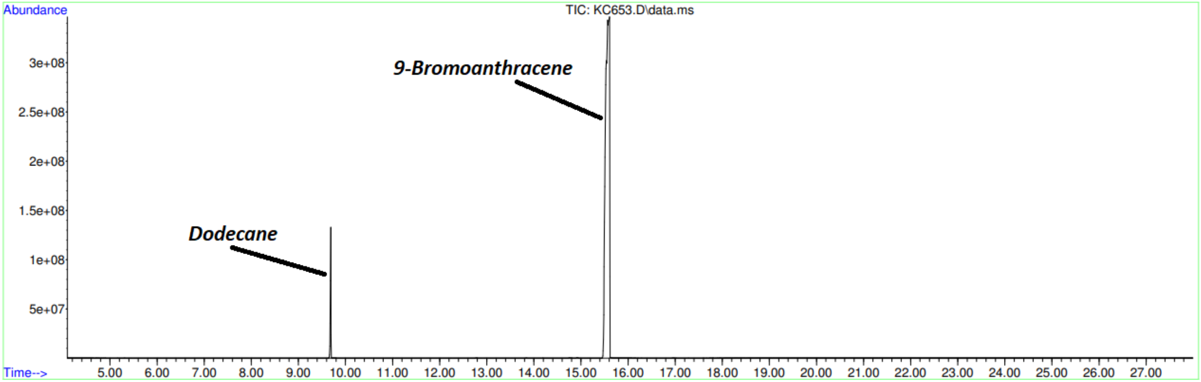

GCFID data including table quantitating components that had been separately calibrated.

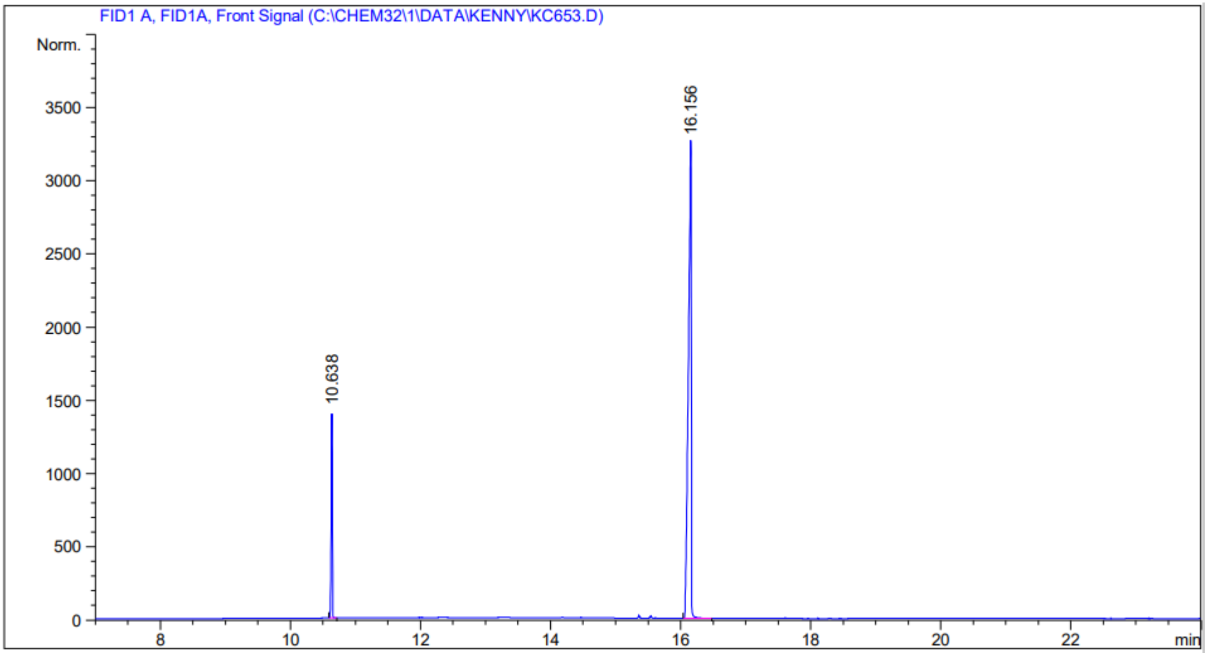

Retention Time	Sample	Peak Area	%Yield
10.640	Dodecane	1742.33716	N/A
16.156	9-Bromoanthracene	9966.78613	99.4%

Dodecane added = 15.9 mg

Reaction of 9-bromoanthracene 40 with NaO*t*Bu and 15-crown-5

To an oven-dried microwave vial, primed with a stirrer bar, in a glovebox was added 9-bromoanthracene **40** (180 mg, 0.7 mmol, 1 equiv.), NaO*t*Bu (135 mg, 1.4 mmol, 2 equiv.), 15-crown-5 (0.28 mL, 1.4 mmol, 2 equiv.) and benzene (7 mL) with the vial subsequently sealed and stirred at 130°C in an oil bath for 24 h. Once complete, the crude mixture was allowed to cool to room temperature, H_2O (0.2 mL) and an accurately weighed amount of dodecane was added. An aliquot of the crude mixture was then analysed by both GCMS and GC-FID (Method 1).

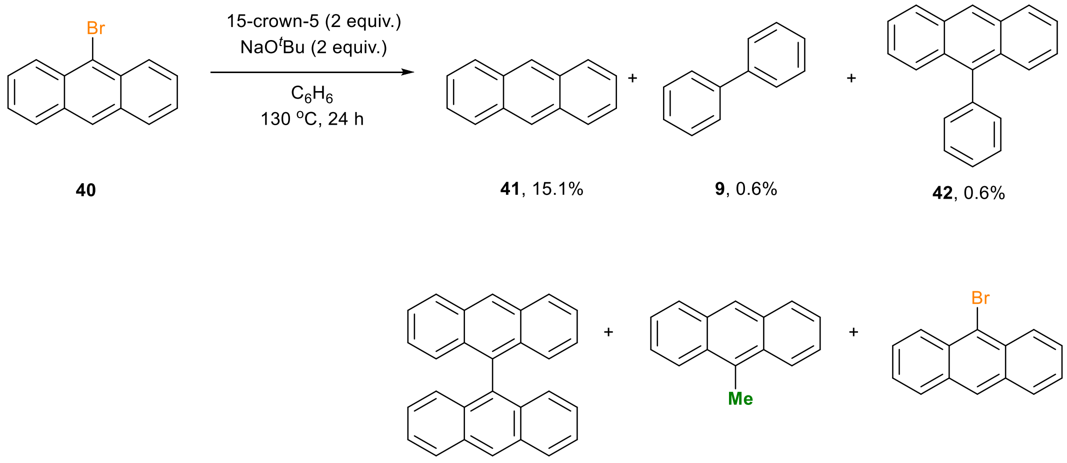

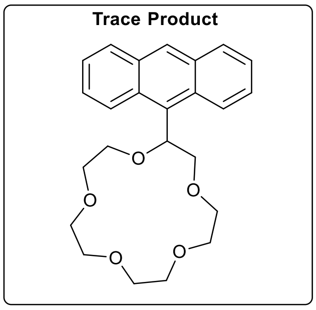

GCMS data

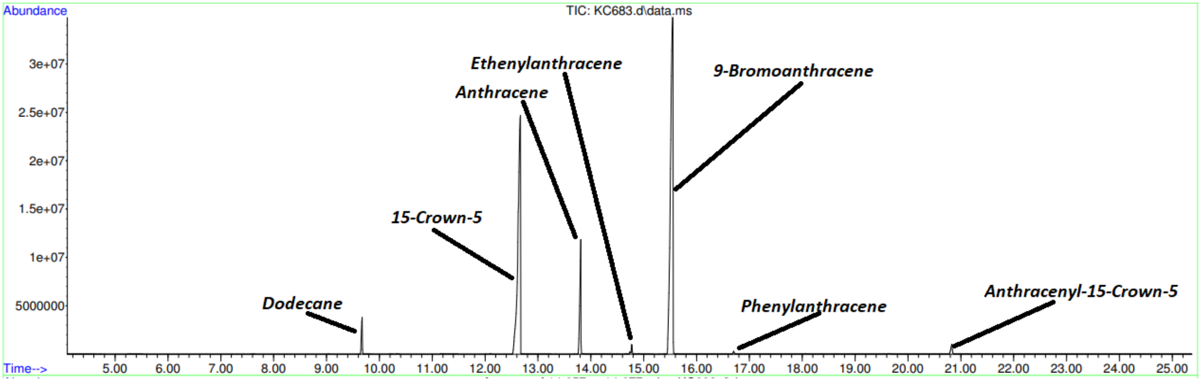

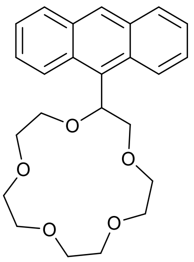

(Calcd.) *m/z*: 396.1937 (100.0%), 397.1970 (26.0%), 398.2004 (3.2%), 398.1979 (1.0%)

2-(Anthracene-9-yl)-1,4,7,10,13-pentaoxacyclopentadecane – **HRMS** (ESI+) [*m/z*] calcd. For $C_{24}H_{29}O_5$ $(M+H)^+$ 397.2010, found 397.2000

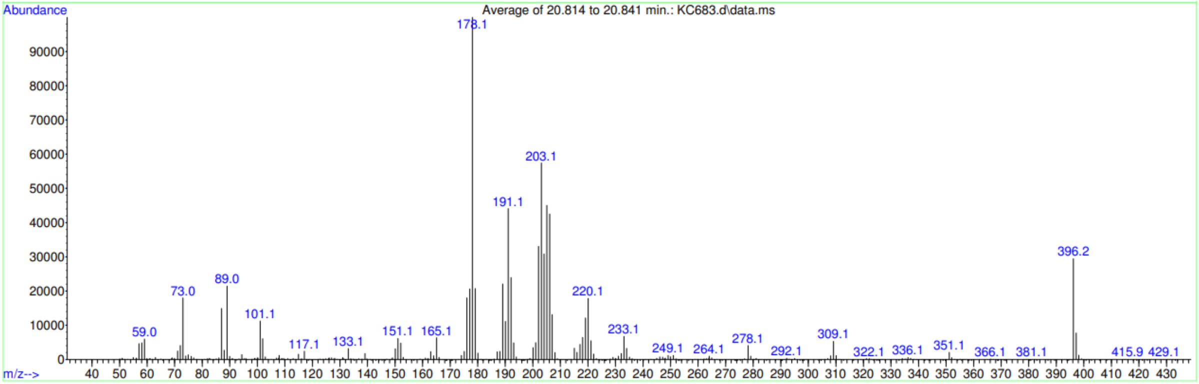

GCFID data including table quantitating components that had been separately calibrated

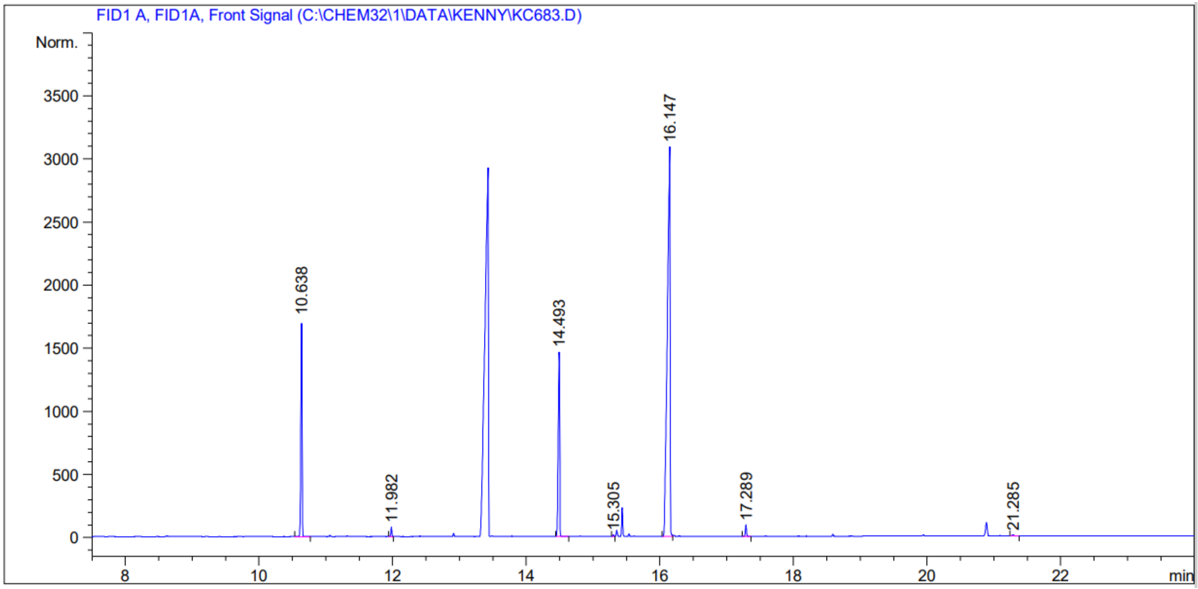

Retention Time (min)	Sample	Peak Area	%Yield
10.638	Dodecane	2178.06958	N/A
11.982	Biphenyl	83.29734	0.6
14.493	Anthracene	2227.43701	15.1
15.305	Methylanthracene	17.66481	0.1
16.147	9-Bromoanthracene	9106.69043	74.1
17.289	Phenylanthracene	103.97724	0.6
21.285	Bianthracene	19.94473	0.2

Dodecane added = 15.5 mg

The data above show that the expected products of radical chemistry are produced in this reaction, where no K^+ salts are present. 15-Crown-5 acts as a good hydrogen atom donor, and so anthracene is expected as the major product from quenching of anthracenyl radicals and diradicals

----

Reaction of 9-bromoanthracene 40 with KOEt

To an oven-dried microwave vial, primed with a stirrer bar, in a glovebox was added 9-bromoanthracene **40** (180 mg, 0.7 mmol, 1 equiv.), KOEt (118 mg, 1.4 mmol, 2 equiv.) and benzene (7 mL) with the vial subsequently sealed and stirred at 130°C in an oil bath for 24 h. Once complete, the crude mixture was allowed to cool to room temperature, H_2O (0.2 mL) and dodecane (15.9 mg) in was added. An aliquot of the crude mixture was then analysed by both GCMS and GC-FID (Method 1).

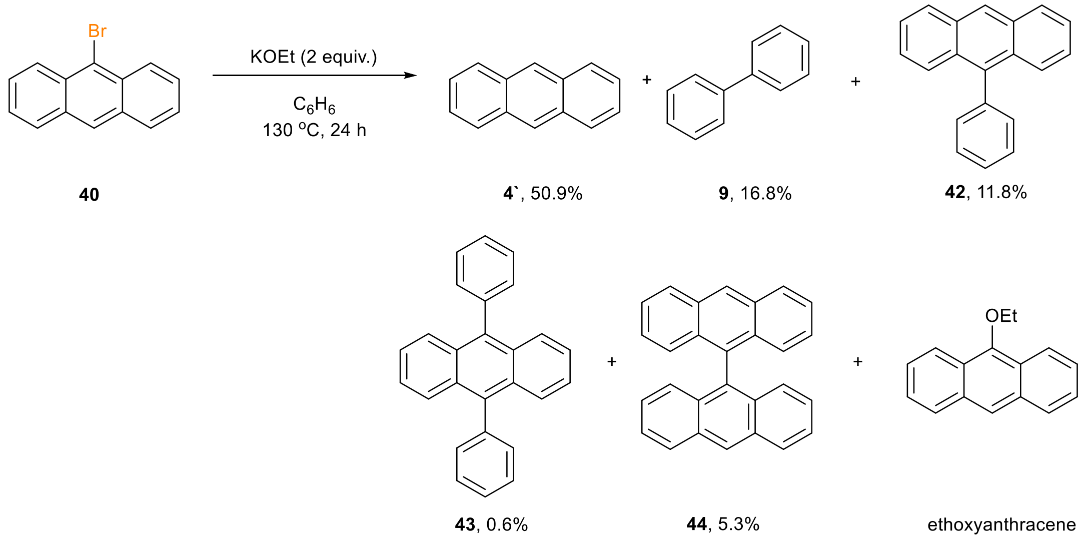

GCMS data

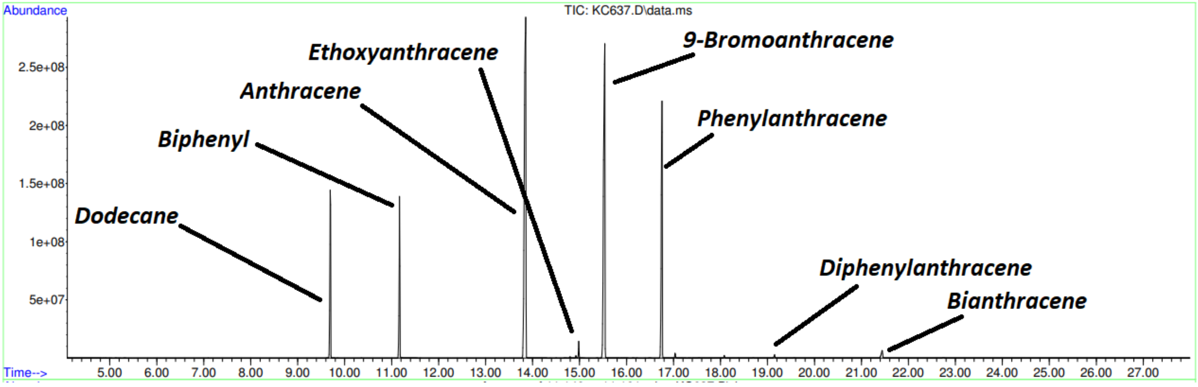

GCFID data including table quantitating components that had been seperately calibrated

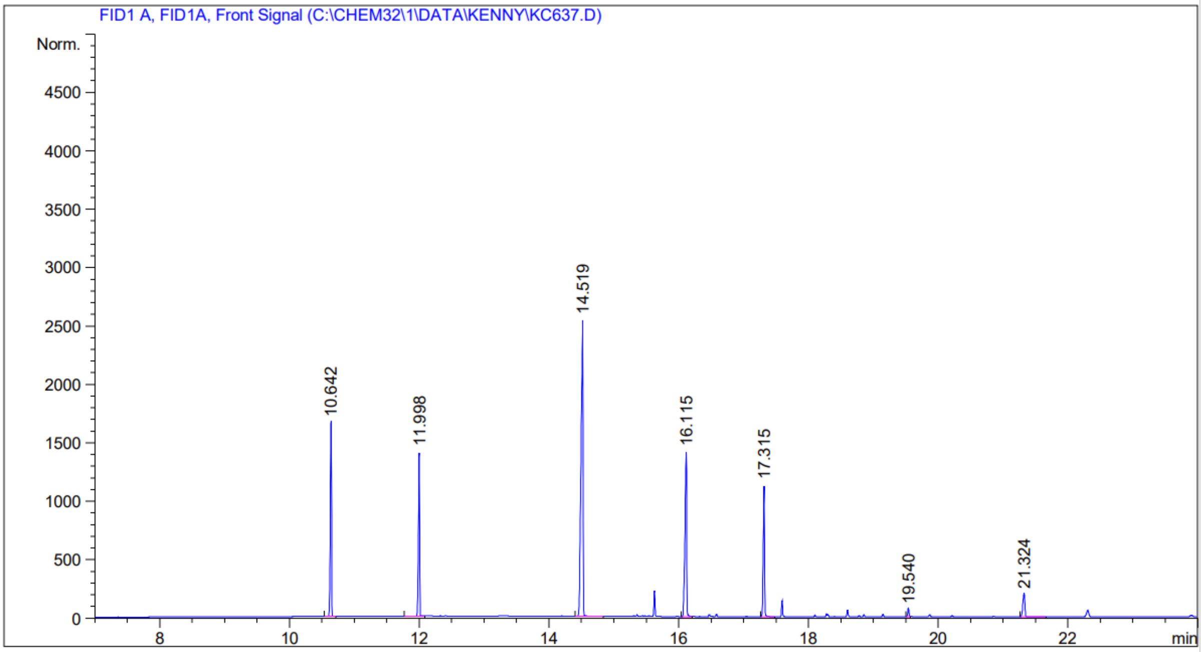

Retention Time (min)	Sample	Peak Area	%Yield
10.642	Dodecane	2174.79834	N/A
11.998	Biphenyl	1816.67480	16.8%
14.519	Anthracene	5531.40283	50.9%
16.115	9-Bromoanthracene	2588.99072	28.6%
17.315	Phenylanthracene	1581.83984	11.8%
19.540	Diphenylanthracene	96.58628	0.6%
21.324	Bianthracene	440.00836	5.3%

Dodecane added = 21.0 mg

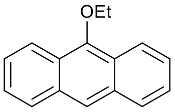

Ethoxyanthracene

m/z: 222.1045 (100.0%), 223.1078 (17.3%), 224.1112 (1.4%)

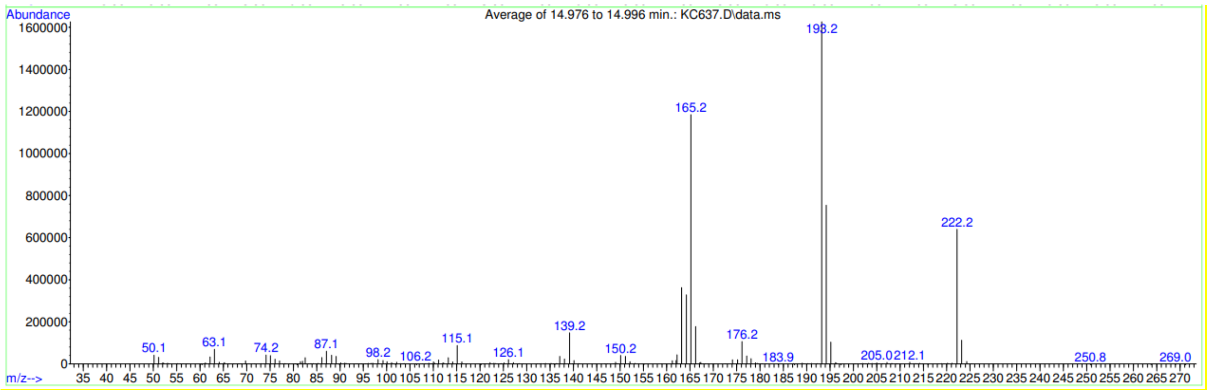

Reaction of 9-bromoanthracene 40 with LiO*t*Bu

To an oven-dried microwave vial, primed with a stirrer bar, in a glovebox was added 9-bromoanthracene **40** (180 mg, 0.7 mmol, 1 equiv.), LiO*t*Bu (112 mg, 1.4 mmol, 2 equiv.) and benzene (7 mL) with the vial subsequently sealed and stirred at 130°C in an oil bath for 24 h. Once complete, the crude mixture was allowed to cool to room temperature, H_2O (0.2 mL) and dodecane (15.9 mg) was added. An aliquot of the crude mixture was then analysed by both GCMS and GC-FID (Method 1).

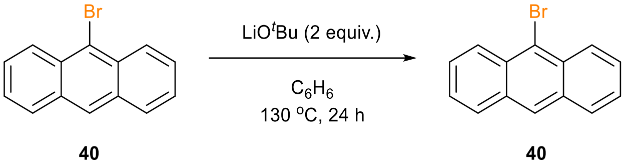

GCMS data

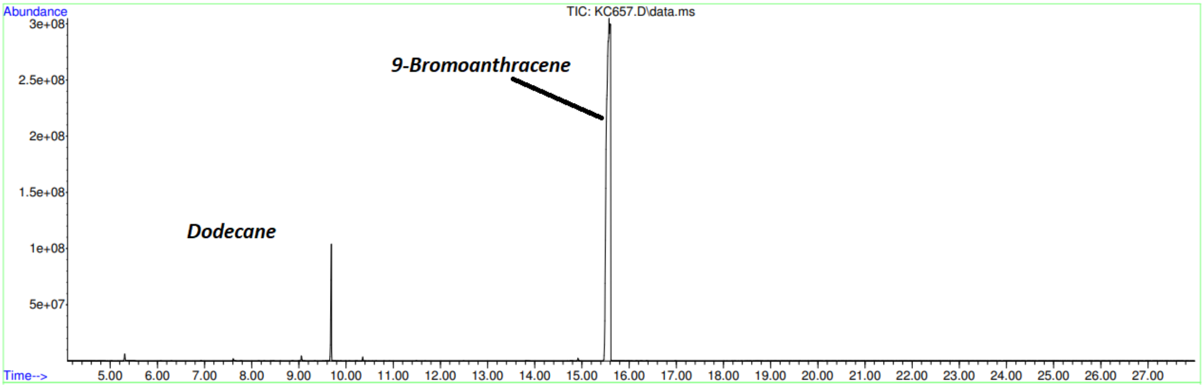

GCFID data including table quantitating components that had been seperately calibrated

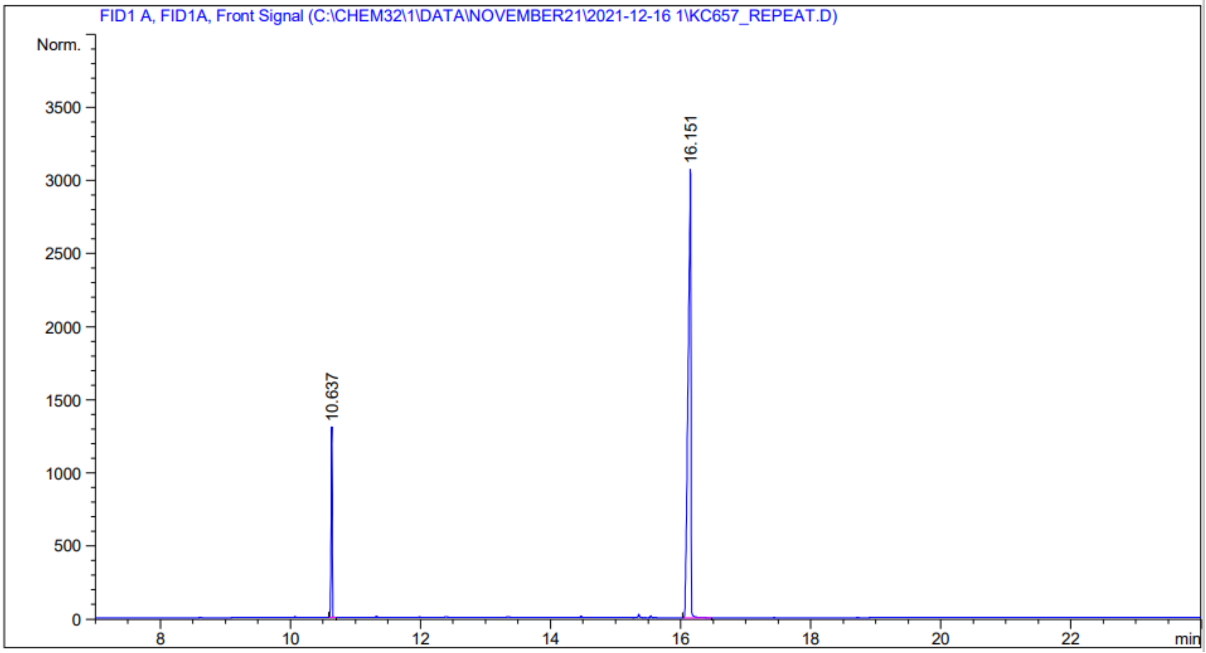

Retention Time (min)	Sample	Peak Area	%Yield
10.637	Dodecane	1564.16309	N/A
16.151	9-Bromoanthracene	9153.33984	99.7%

Dodecane added = 15.8 mg

<u>Reaction of 9-bromoanthracene 40 with $KOCEt_3$ in C_6H_6</u>

In this experiment KO*t*Bu was replaced with another tertiary potassium alkoxide, potassium *tert*-heptoxide ($KOCEt_3$). In this case, ethylated products (rather than methylated products) were observed. As with KO*t*Bu, HAT by radicals from the 2° carbon on $KOCEt_3$, and subsequent fragmentation would yield ethyl radicals.

To an oven-dried microwave vial, primed with a stirrer bar, in a glovebox was added 9-bromoanthracene **40** (180 mg, 0.7 mmol, 1 equiv.), $KOCEt_3$ (216 mg, 1.4 mmol, 2 equiv.) and benzene (7 mL) with the vial subsequently sealed and stirred at 130°C in an oil bath for 24 h. Once complete, the crude mixture was allowed to cool to room temperature, H_2O (0.2 mL) and dodecane (15.2 mg) was added. An aliquot of the crude mixture was then analysed by both GCMS and GC-FID (Method 1).

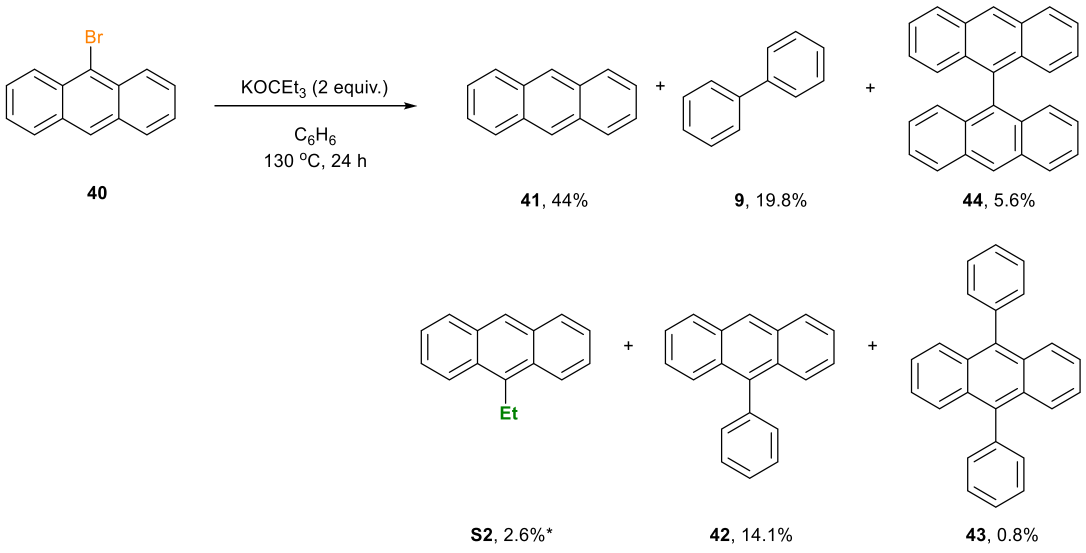

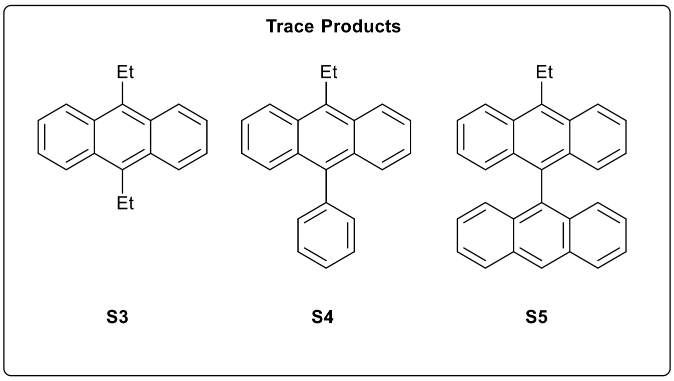

GCMS data

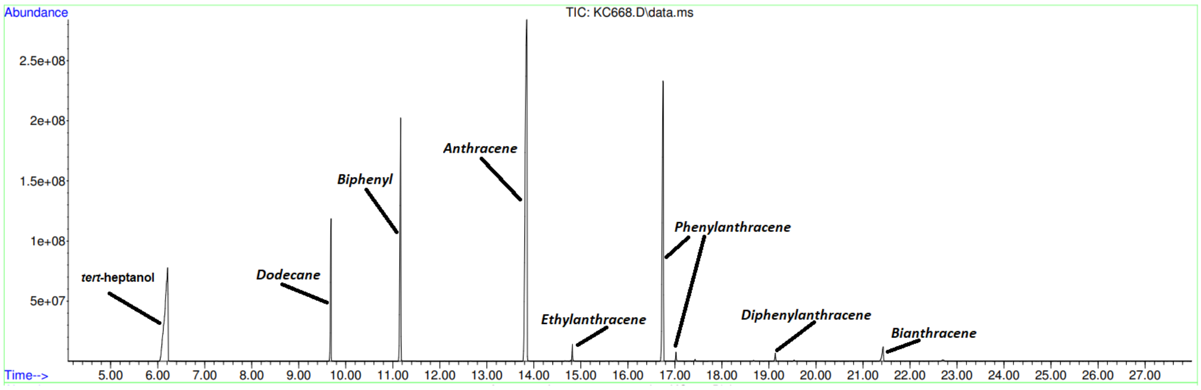

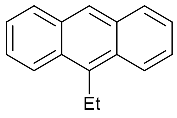

(Calcd.) *m/z*: 206.1096 (100.0%), 207.1129 (17.3%), 208.1163 (1.4%)

9-ethylanthracene **S2** - **HRMS** (EI+) [*m/z*] calcd. for $C_{16}H_{14}$ (M^+) 206.1096, found 206.1093.

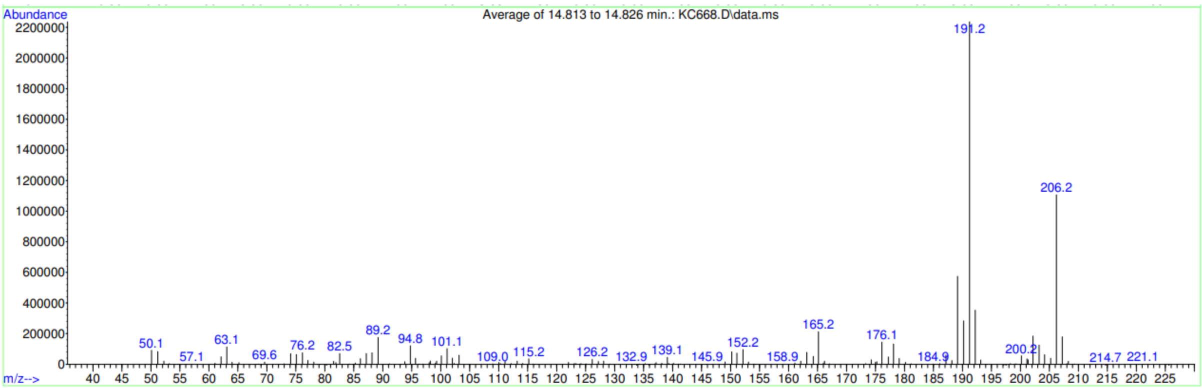

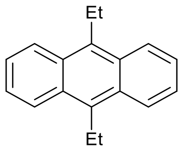

(Calcd.) *m/z*: 234.1409 (100.0%), 235.1442 (19.5%), 236.1476 (1.8%)

Diethylanthracene **S3** - **HRMS** (EI+) [*m/z*] calcd. for $C_{18}H_{18}$ (M^{+}) 234.1409, found 234.1410.

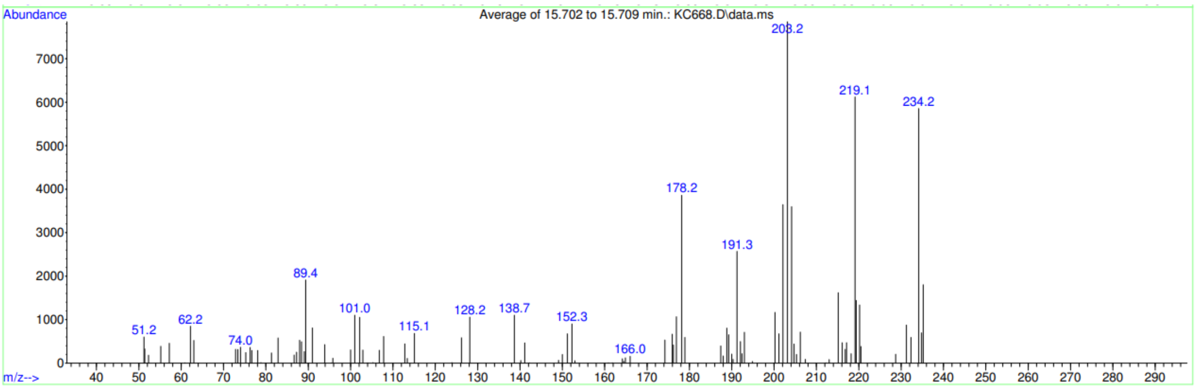

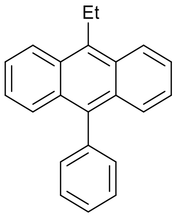

(Calcd.) *m/z*: 282.1409 (100.0%), 283.1442 (23.8%), 284.1476 (2.7%)

9-ethyl-10-phenylanthracene **S4** - **HRMS** (EI+) [*m/z*] calcd. for $C_{22}H_{18}$ (M^{+}) 282.1409, found 282.1413.

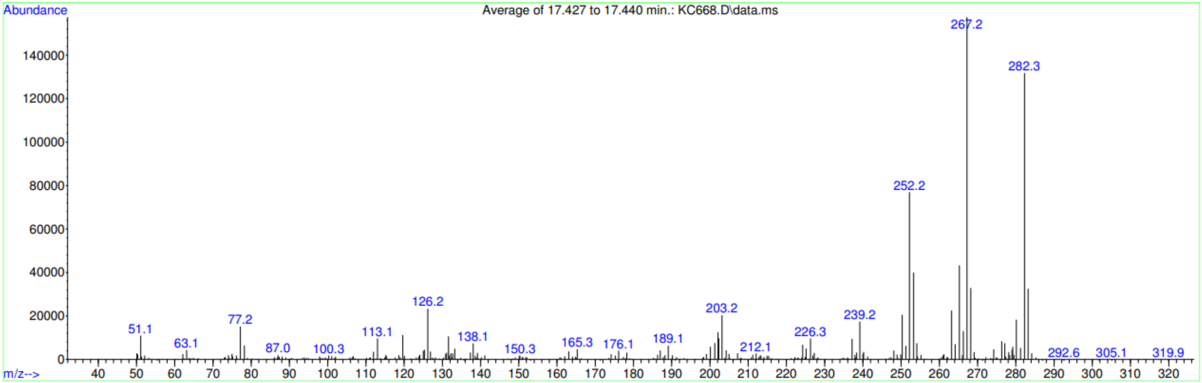

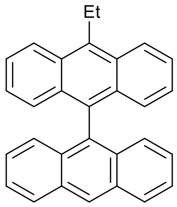

(Calcd.) *m/z*: 382.1722 (100.0%), 383.1755 (32.4%), 384.1789 (5.1%)

9-ethylbianthracene **S5** - **HRMS** (EI+) [*m/z*] calcd. for $C_{30}H_{22}$ (M^{+}) 382.1722, found 382.1726.

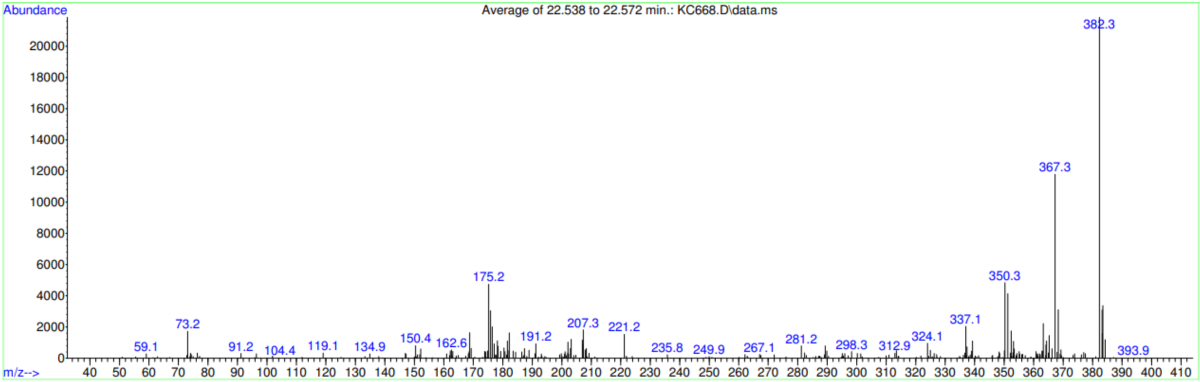

GCFID data including table quantitating components that had been separately calibrated.

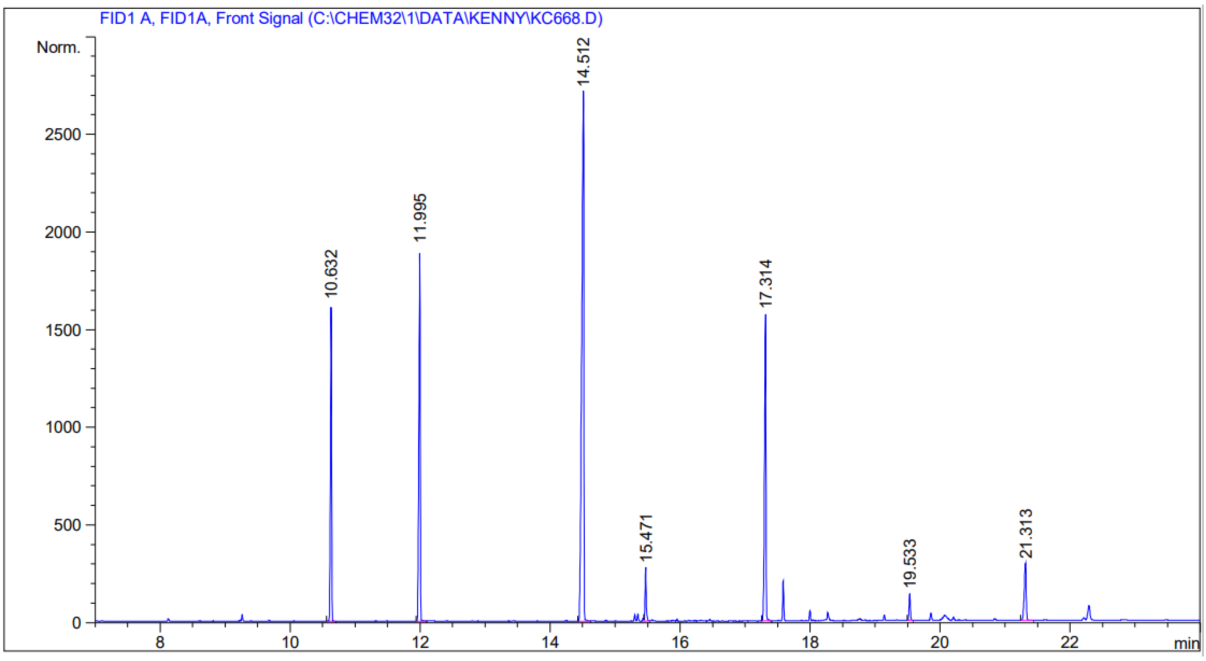

Retention Time (min)	Sample	Peak Area	%Yield
10.632	Dodecane	2019.85681	N/A
11.995	Biphenyl	2744.74390	19.8%
14.512	Anthracene	6139.38672	44.0%
15.471	Ethylanthracene	338.56952	2.6%*
17.314	Phenylanthracene	2434.26953	14.1%
19.533	Diphenylanthracene	189.87830	0.8%
21.313	Bianthracene	603.26007	5.6%

*%yield of ethylanthracene **S2** calculated using the calibration method for 9-methylanthracene

Dodecane added = 15.2 mg

Reaction of 9-chloroanthracene 40^{Cl} with KO*t*Bu in C_6H_6

To an oven-dried microwave vial, primed with a stirrer bar, in a glovebox was added 9-chloroanthracene **40**^{Cl} (149 mg, 0.7 mmol, 1 equiv.), KO*t*Bu (157 mg, 1.4 mmol, 2 equiv.) and benzene (7 mL) with the vial subsequently sealed and stirred at 130°C in an oil bath for 24 h. Once complete, the crude mixture was allowed to cool to room temperature, H_2O (0.2 mL) and dodecane (15.8 mg) in was added. An aliquot of the crude mixture was then analysed by both GCMS and GC-FID (Method 1).

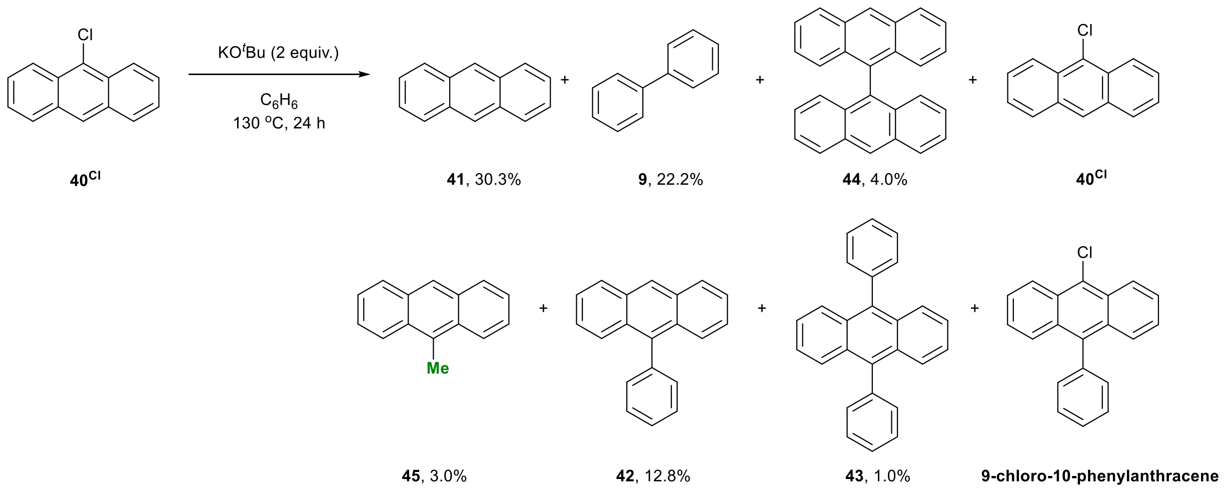

Products from reaction of chloroanthracene are show above

GCMS data

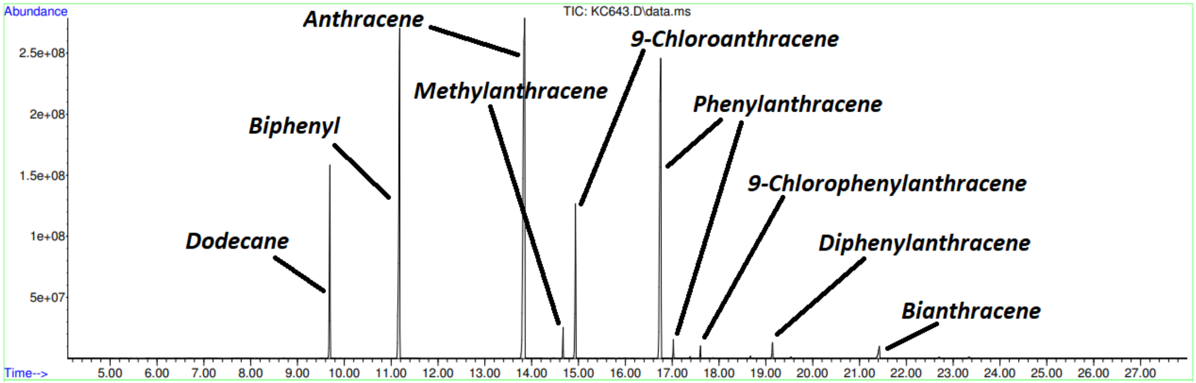

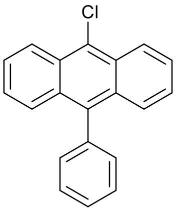

9-chloro-10-phenylanthracene

(Calcd.) *m/z*: 288.0706 (100.0%), 290.0676 (32.0%), 289.0739 (21.6%), 291.0710 (6.9%), 290.0773 (2.2%)

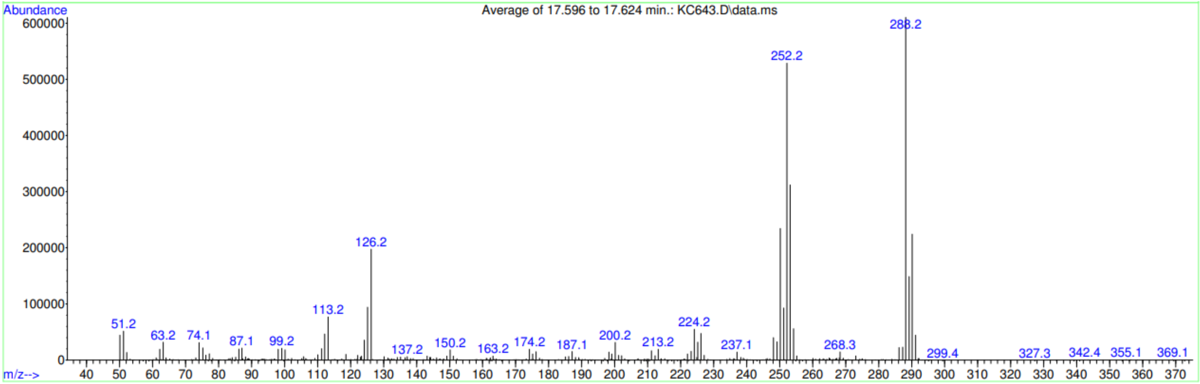

GCFID data including table quantitating components that had been separately calibrated.

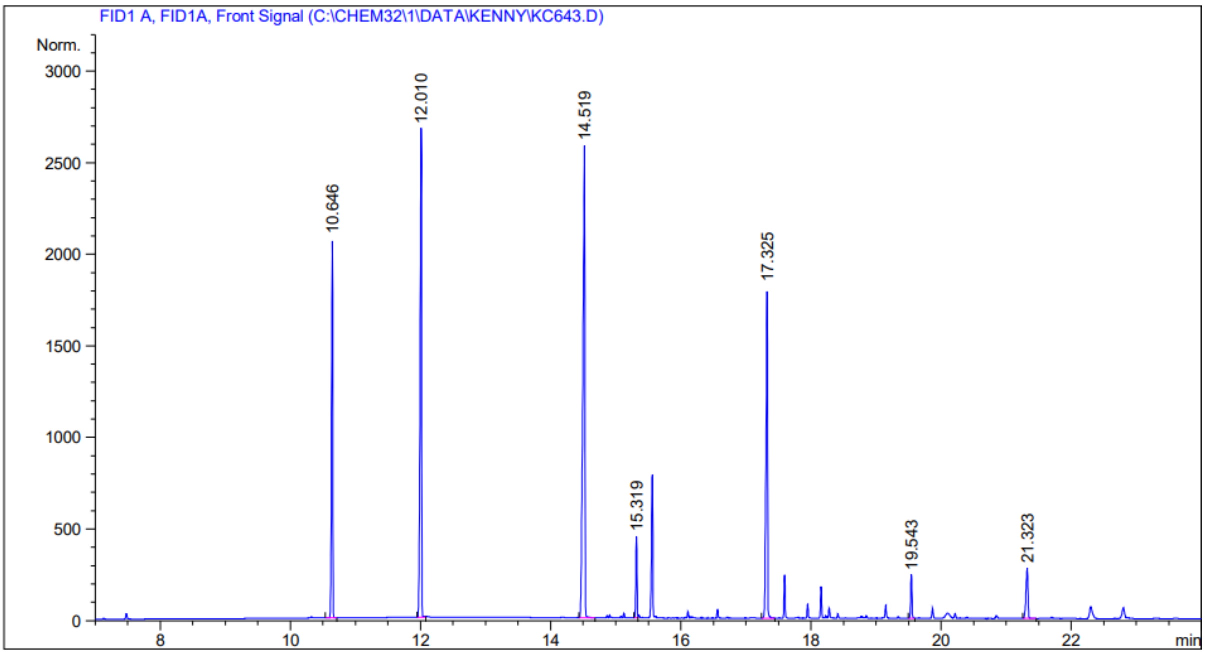

Retention Time (min)	Sample	Peak Area	%Yield
10.646	Dodecane	2800.229	N/A
12.010	Biphenyl	4252.313	22.2%
14.519	Anthracene	5626.188	30.3%
15.319	Methylanthracene	543.432	3.0%
17.325	Phenylanthracene	3032.333	12.8%
19.543	Diphenylanthracene	321.569	1.0%
21.323	Bianthracene	577.271	4.0%

Dodecane added = 15.8 mg

Reaction of 9-iodoanthracene 40[I] with KO*t*Bu in C_6H_6

To an oven-dried microwave vial, primed with a stirrer bar, in a glovebox was added 9-iodoanthracene **40c** (106 mg, 0.35 mmol, 1 equiv.), KO*t*Bu (79 mg, 0.7 mmol, 2 equiv.) and benzene (3.5 mL) with the vial subsequently sealed and stirred at 130°C in an oil bath for 24 h. Once complete, the crude mixture was allowed to cool to room temperature, H_2O (0.2 mL) and dodecane (9.1 mg) in EtOAc (5 mL) was added. An aliquot of the crude mixture was then analysed by both GCMS and GC-FID (Method 2).

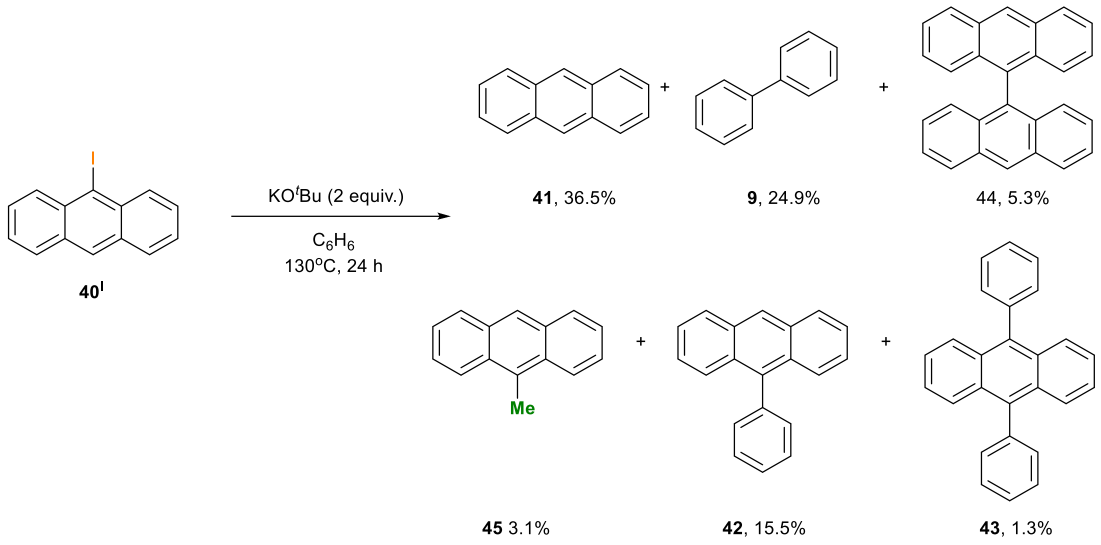

GC-MS data

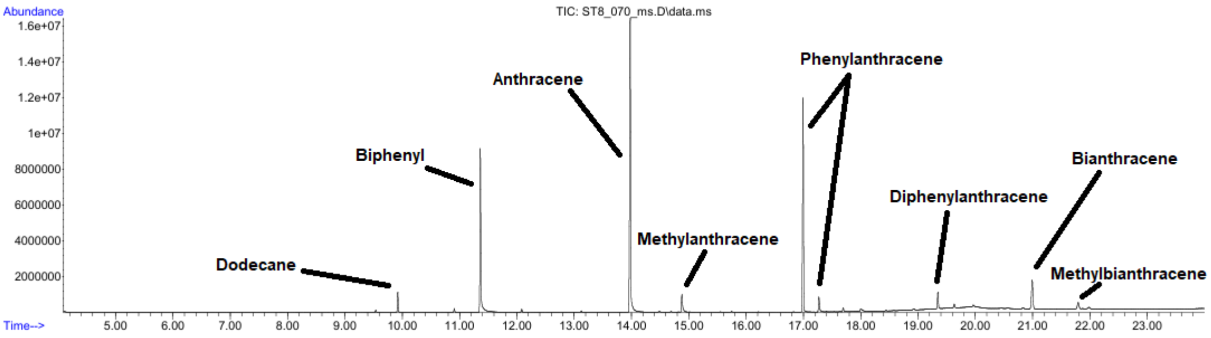

GCFID data including table quantitating components that had been separately calibrated.

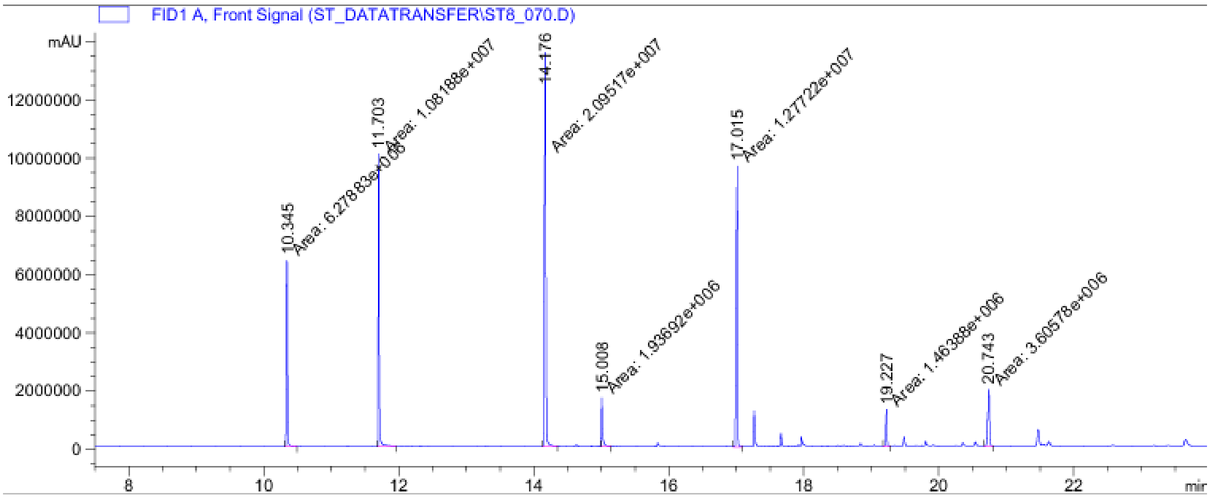

Retention Time (min)	Sample	Peak Area	%Yield
10.345	Dodecane	6278830	N/A
11.703	Biphenyl	10818800	24.9
14.176	Anthracene	20951700	36.5
15.008	Methylanthracene	1936920	3.1
17.015	Phenylanthracene	12772200	15.5
19.227	Diphenylanthracene	1463880	1.3
20.743	Bianthracene	3065780	5.3

Dodecane added = 9.1 mg

Side-by-side-by-side reactions of different haloanthracenes

In this experiment we see that bromoanthracene reacts faster than iodoanthracene, which is not consistent with a SET pathway. Potentially, the slower reaction of iodoanthracene compared to bromoanthracene is due to its lower acidity, slowing down formation of the initiating benzynes. Of course, chloroanthracene is likely even more acidic than bromoanthracene, but the higher strength of the Ar–Cl will also slow down the rate of reaction.

To an oven-dried microwave vial, primed with a stirrer bar, in a glovebox was added a 9-haloanthracene **40** (0.35 mmol, 1 equiv.), KO*t*Bu (79 mg, 0.7 mmol, 2 equiv.) and benzene (3.5 mL) with the vial subsequently sealed and stirred at 130 °C in a pre-heated oil bath for 30 min. Once complete, the crude mixture was quickly cooled to room temperature, H_2O (0.2 mL) and an accurately weighed mass of *n*-dodecane in EtOAc (5 mL) was added. An aliquot of the crude mixture was then analysed by both GCMS and GC-FID (Method 2).

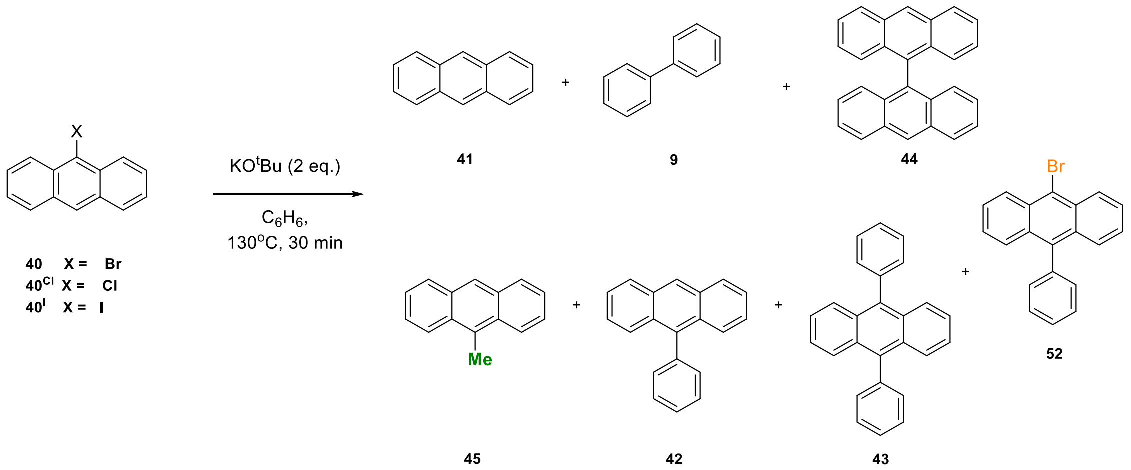

Substrate X =	Dodecane (mg)	% Yield[a]							
		40	9	41	42	43	44	45	52
Cl	6.6	N/A	3.7	4.4	1.3	0.04	0.1	0.2	N/A
Br	6.7	62.1	6.7	9.9	4.0	0.3	1.2	1.0	0.6
I	6.7	N/A	4.7	10.5	3.3	0.1	1.0	0.4	N/A

[a]*Yields determined by GC-FID calibrated with authentic samples.*

GC-MS data for reaction of 9-chloroanthracene

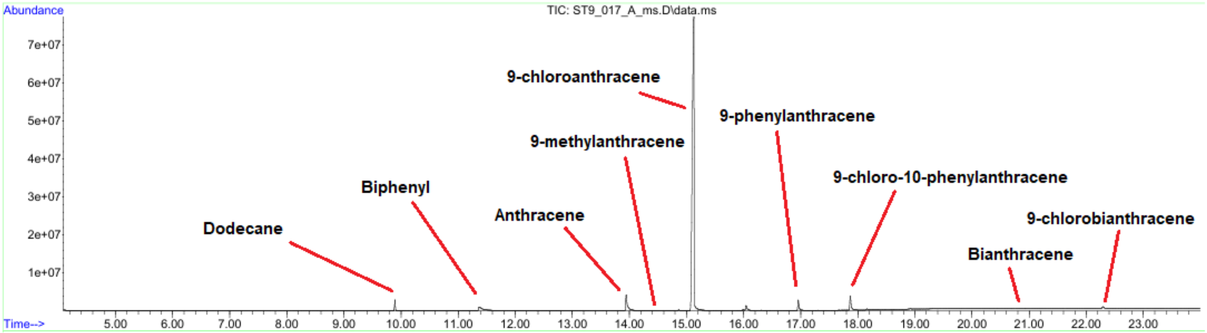

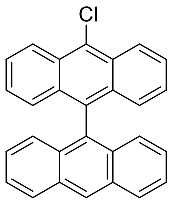

(Calc.) *m/z*: 388.1019 (100.0%), 390.0989 (32.0%), 389.1052 (30.3%), 391.1023 (9.7%)

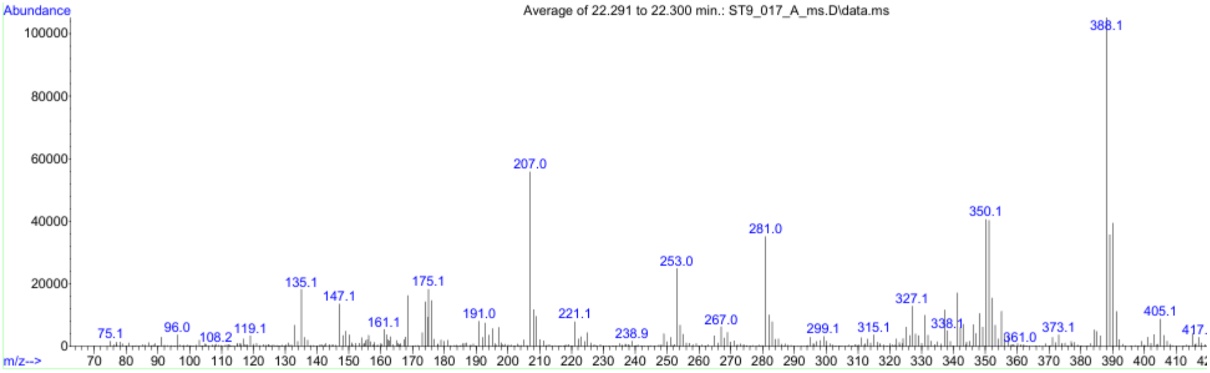

GCFID data for reaction of 9-chloroanthracene including table quantitating components that had been separately calibrated.

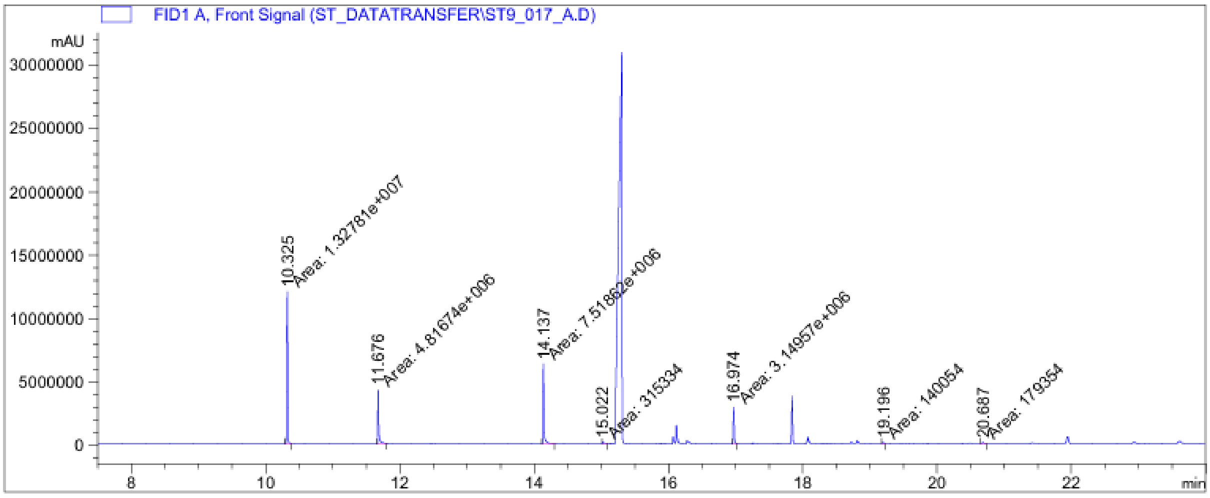

Retention Time (min)	Sample	Peak Area	%Yield
10.325	Dodecane	13278100	N/A
11.676	Biphenyl	4816740	3.7
14.137	Anthracene	7518620	4.4
15.022	Methylanthracene	315334	0.2
16.974	Phenylanthracene	3149570	1.3
19.196	Diphenylanthracene	140054	0.04
20.687	Bianthracene	179354	0.1

Dodecane added = 6.5 mg

GC-MS data for reaction of 9-bromoanthracene

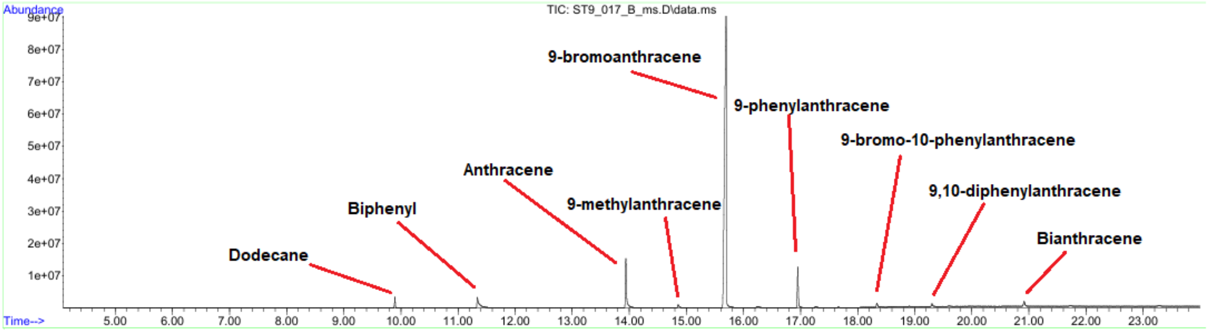

GCFID data for reaction of 9-bromoanthracene including table quantitating components that had been separately calibrated.

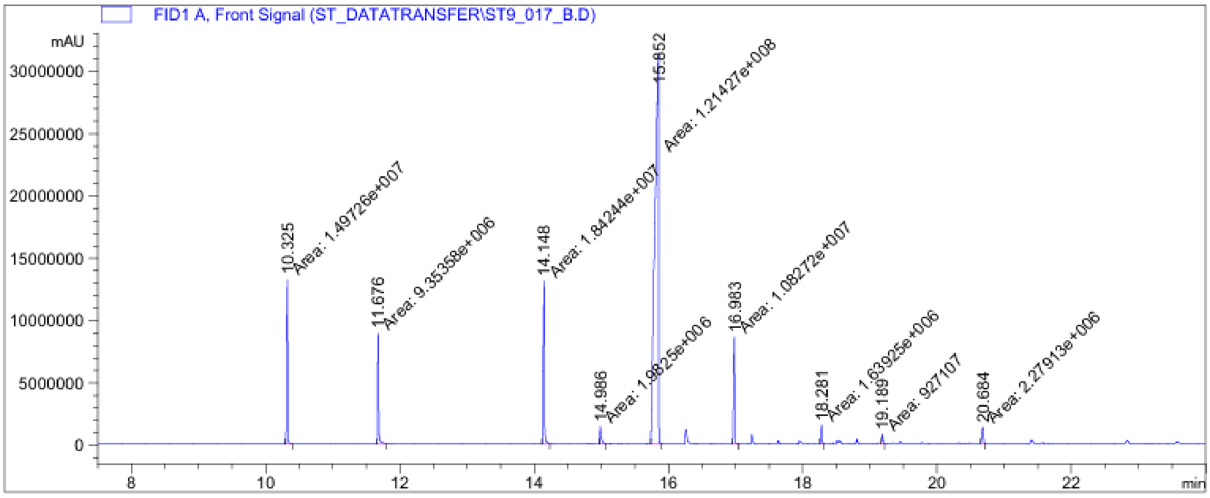

Retention Time (min)	Sample	Peak Area	%Yield
10.325	Dodecane	14972600	N/A
11.676	Biphenyl	9353580	6.7
14.148	Anthracene	18424400	9.9
14.986	Methylanthracene	1982500	1.0
15.852	Bromoanthracene	121427000	62.1
16.983	Phenylanthracene	10827200	4.0
18.281	Bromophenylanthracene	1639250	0.6
19.189	Diphenylanthracene	927107	0.3
20.684	Bianthracene	2279130	1.2

Dodecane added = 6.7 mg

GC-MS data for reaction of 9-iodoanthracene

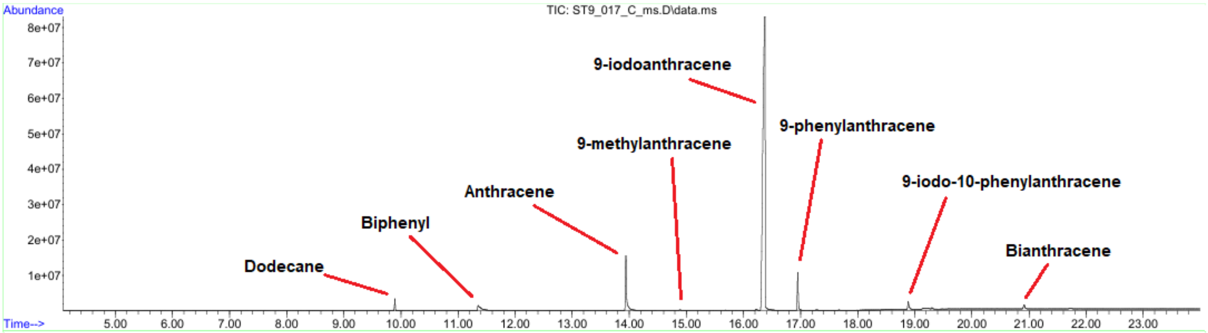

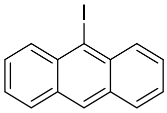

(calc.) *m/z*: 303.9749 (100.0%), 304.9783 (15.1%), 305.9816 (1.1%)

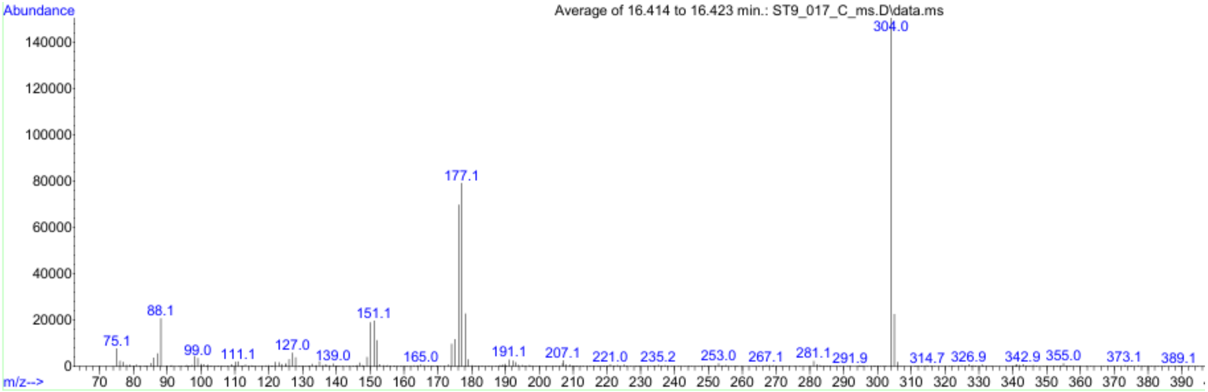

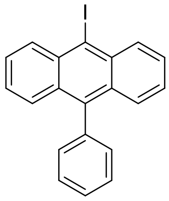

(calc.) *m/z*: 380.0062 (100.0%), 381.0096 (21.6%), 382.0129 (2.2%)

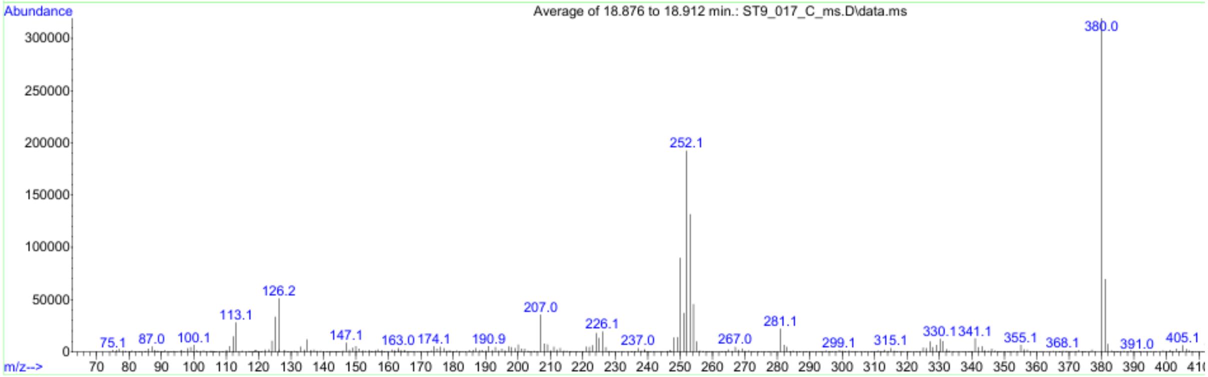

GCFID data for reaction of 9-iodoanthracene including table quantitating components that had been separately calibrated.

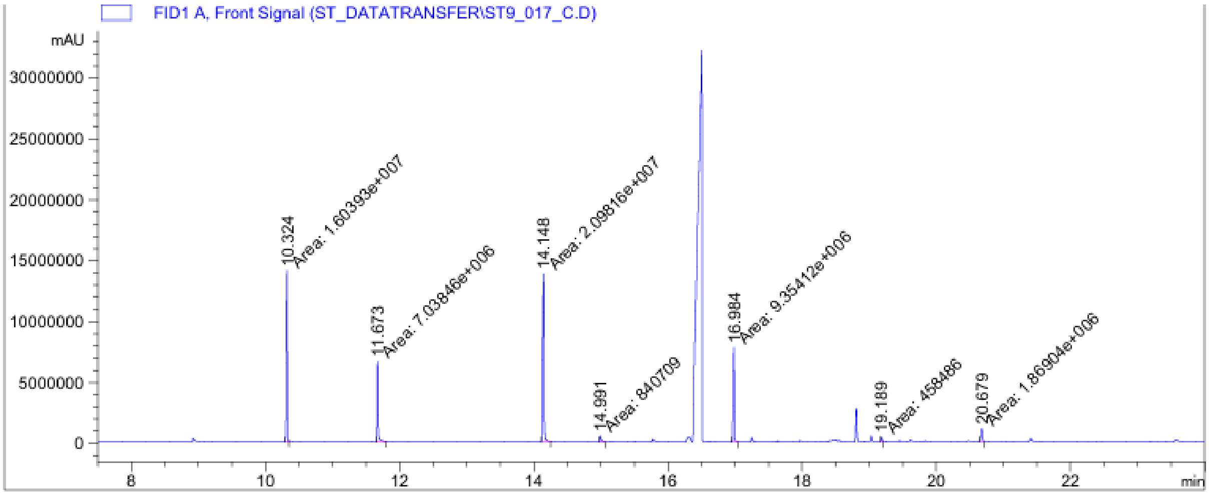

Retention Time (min)	Sample	Peak Area	%Yield
10.324	Dodecane	16039300	N/A
11.673	Biphenyl	7038460	4.7
14.148	Anthracene	20981600	10.5
14.991	Methylanthracene	840709	0.4
16.984	Phenylanthracene	9354120	3.3
19.189	Diphenylanthracene	458486	0.1
20.679	Bianthracene	1869040	1.0

Dodecane added = 6.7 mg

Side-by-side-by-side-by-side reactions of bromoanthracene isotopologues (Table 2 in paper)

To an oven-dried microwave vial, primed with a stirrer bar, in a glovebox was added a 9-bromoanthracene isotopologue (0.35 mmol, 1 equiv.) or mixture of two isotopologues (0.175 mmol, 0.5 eq. of each), KO*t*Bu (79 mg, 0.7 mmol, 2 equiv.) and benzene (3.5 mL) with the vial subsequently sealed and stirred at 130°C in a pre-heated oil bath for 30 min. Once complete, the crude mixture was quickly cooled to room temperature, D_2O (0.2 mL) and an accurately weighed mass of *n*-dodecane in EtOAc (5 mL) was added. An aliquot of the crude mixture was then analysed by both GCMS and GC-FID (Method 2).

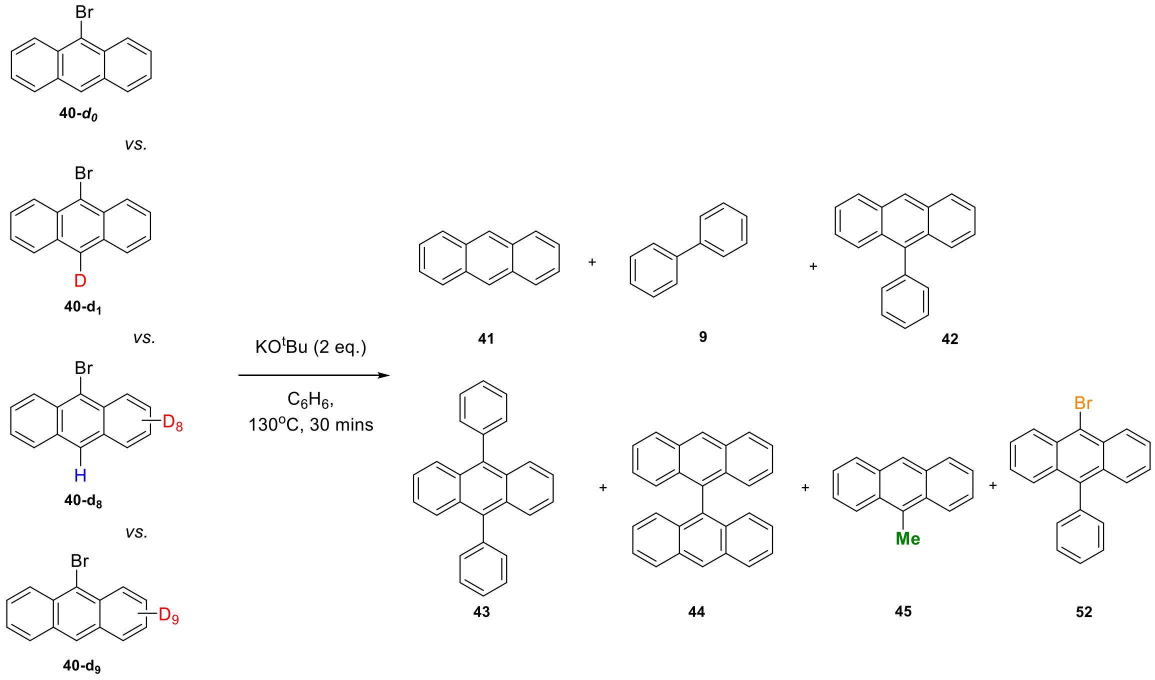

Or isotopologues, depending on substrate used

The side-by-side experiments were repeated 3 separate times, with the average yields of each compound given below. The individual runs:

Substrate		% Yield[a]						
	40	9	41	42	43	44	45	52
**40-d_0**	45.1	12.4	17.6	7.1	0.5	2.0	1.7	0.8
**40-d_1**	65.2	5.7	8.7	3.3	0.2	1.1	0.9	0.5
**40-d_8**	75.7	2.2	3.5	1.4	0.1	0.5	0.4	0.2
**40-d_9**	81.0	1.8	2.8	1.1	0.1	0.3	0.3	0.2

[a] Yields determined by GC-FID calibrated with authentic samples. Isotopologue yields calculated using calibrations of non-labelled compounds.

GCFID data for reaction of 40-d_0 including table quantitating components that had been separately calibrated. (Table 2, entry 1)

Run 1

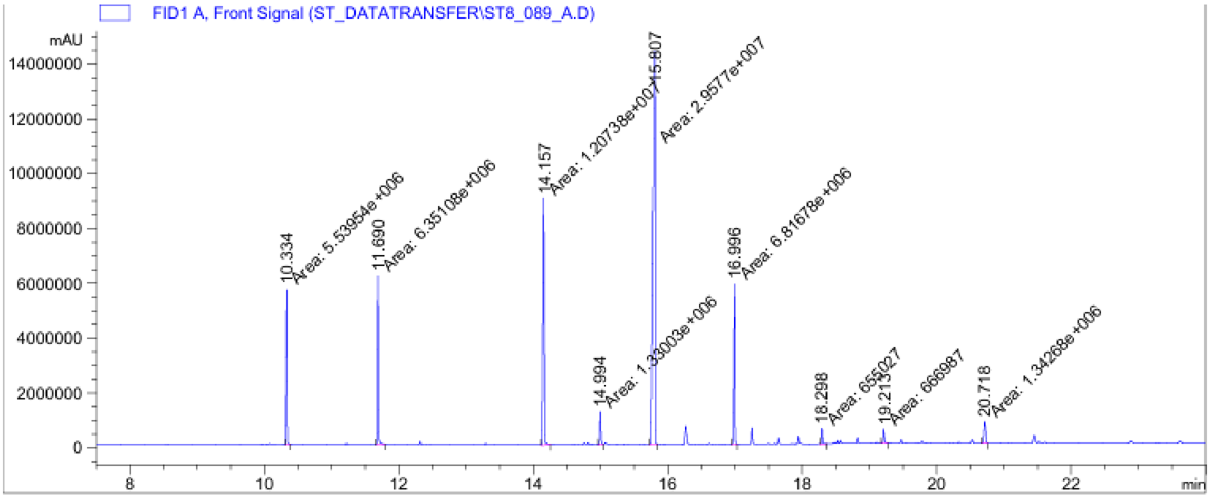

Retention Time (min)	Sample	Peak Area	%Yield
10.334	Dodecane	5539540	N/A
11.690	Biphenyl	6351080	12.8
14.157	Anthracene	12073800	18.3
14.994	Methylanthracene	1330030	1.9
15.807	Bromoanthracene	29577000	42.7
16.996	Phenylanthracene	6816780	7.2
18.298	Bromophenylanthracene	655027	0.7
19.213	Diphenylanthracene	666987	0.5
20.716	Bianthracene	1342680	2.0

Dodecane added = 7.0 mg

Run 2

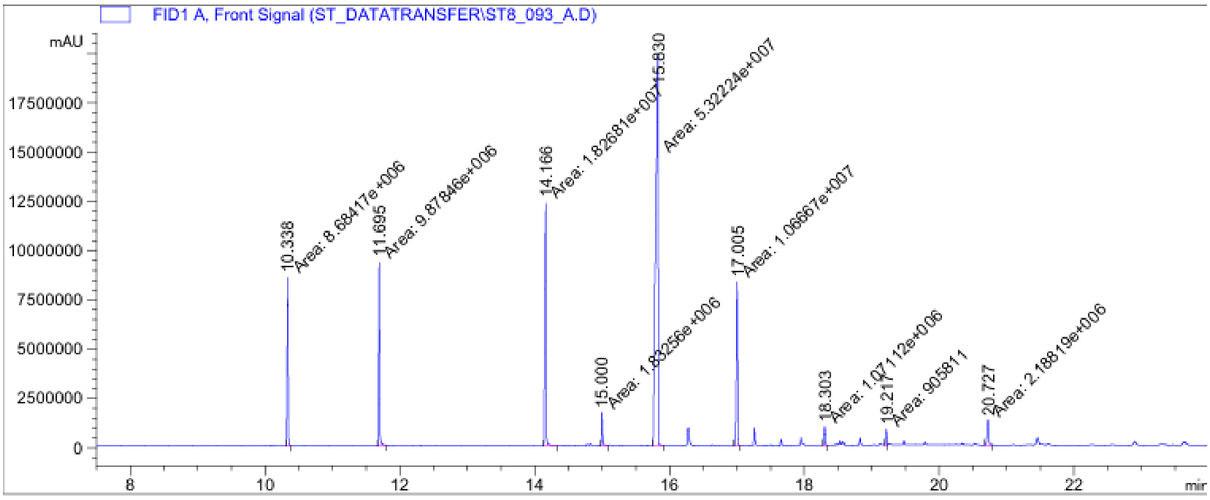

Retention Time (min)	Sample	Peak Area	%Yield
10.338	Dodecane	8684170	N/A
11.695	Biphenyl	9878460	12.1
14.166	Anthracene	18268100	16.9
15.000	Methylanthracene	1832560	1.6
15.830	Bromoanthracene	53222400	46.9
17.005	Phenylanthracene	10666700	6.9
18.303	Bromophenylanthracene	1071120	0.7
19.217	Diphenylanthracene	905811	0.4
20.727	Bianthracene	2188190	2.0

Dodecane added = 6.7 mg

Run 3

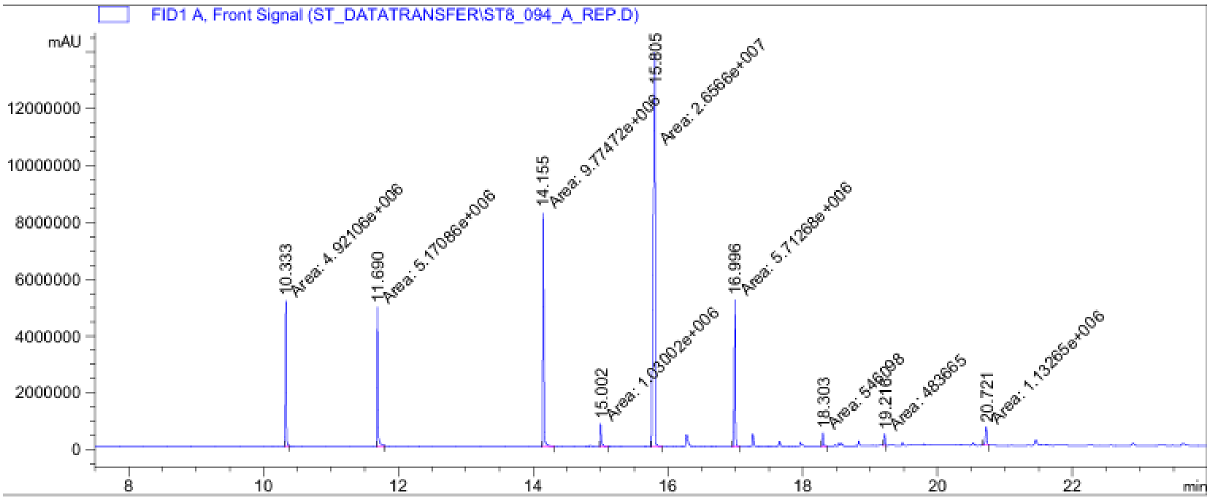

Retention Time (min)	Sample	Peak Area	%Yield
10.333	Dodecane	4921060	N/A
11.690	Biphenyl	5170860	12.4
14.155	Anthracene	9774720	17.7
15.002	Methylanthracene	1030020	1.7
15.805	Bromoanthracene	26566000	45.6
16.996	Phenylanthracene	5712680	7.2
18.303	Bromophenylanthracene	546098	0.7
19.216	Diphenylanthracene	483665	0.5
20.271	Bianthracene	1132650	2.0

Dodecane added = 7.4 mg

GC-MS data for reaction of 40-d_1 (Table 2, entry 2)

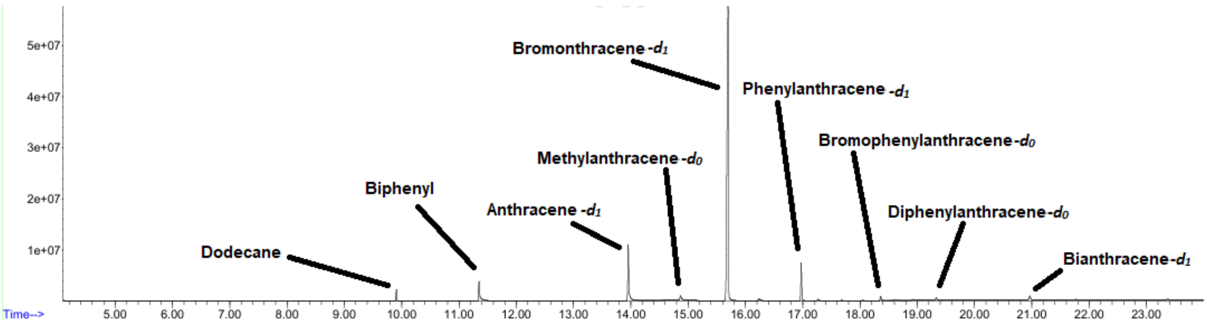

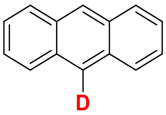

(Calcd.) *m/z*: 179.08 (100.0%), 180.09 (15.2%), 181.09 (1.1%)

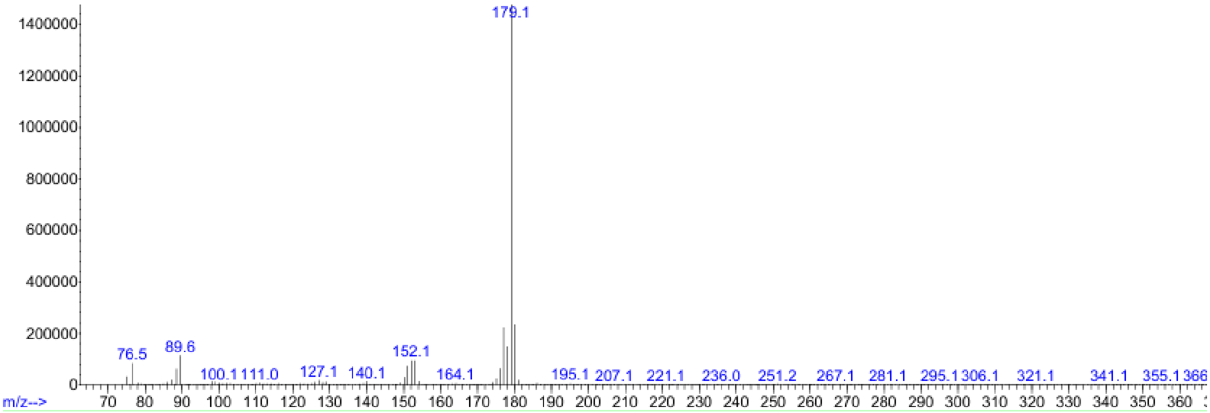

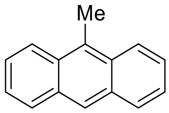

(Calcd.) *m/z*: 192.09 (100.0%), 193.10 (16.4%), 194.10 (1.3%)

Shows a mixture of 9-methylanthracene-d_0 and a small quantity of 9-methylanthracene-d_1, resulting respectively from either *para*- or *ipso*-attack of a methyl radical on the Br site of bromoanthracene-d_1.

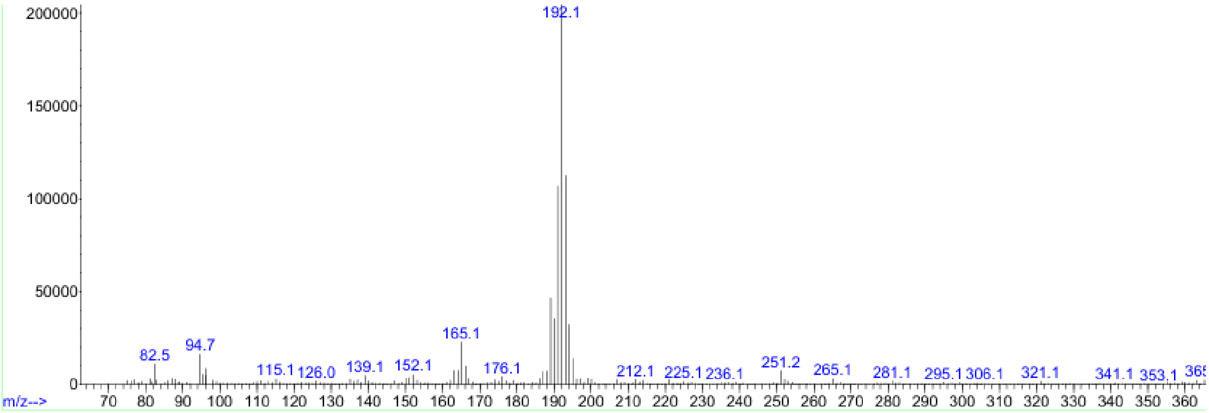

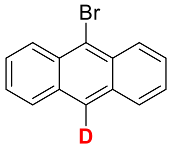

(Calcd.) *m/z*: 257.00 (100.0%), 258.99 (97.3%), 258.00 (15.2%), 260.00 (14.8%)

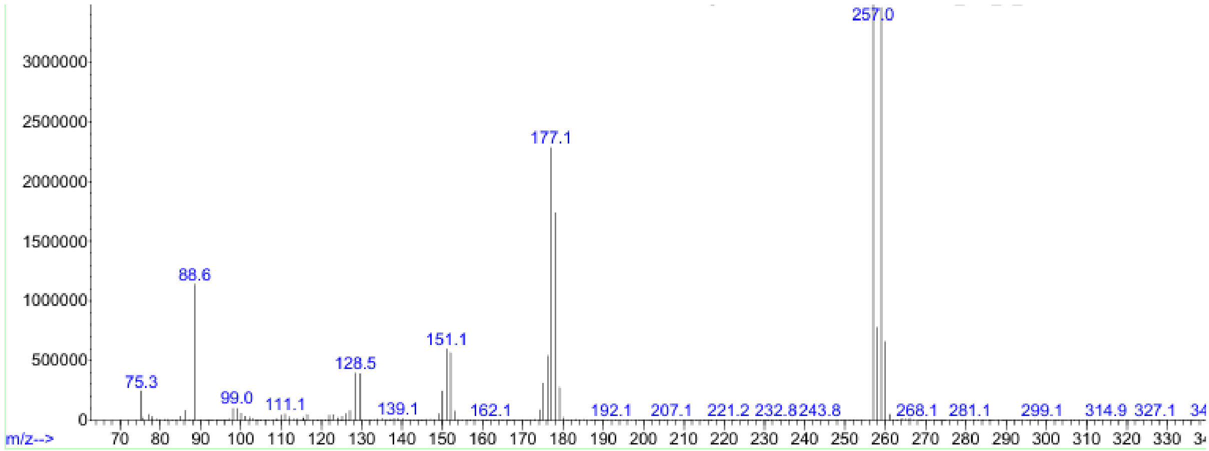

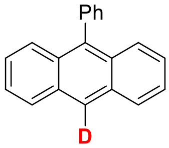

(Calcd.) *m/z*: 255.12 (100.0%), 256.12 (21.8%), 257.12 (2.2%)

Shows a mixture of 9-phenylanthracene-d_1 and 9-phenylanthracene-d_0, resulting respectively from

either (i) attack of an anthracenyl-d_1 radical on benzene (d_1) or *ipso*-attack of a phenyl radical on bromoanthracene-d_1 (d_1) or (ii) *para*-attack of a phenyl radical on bromoanthracene-d_1 (d_0).

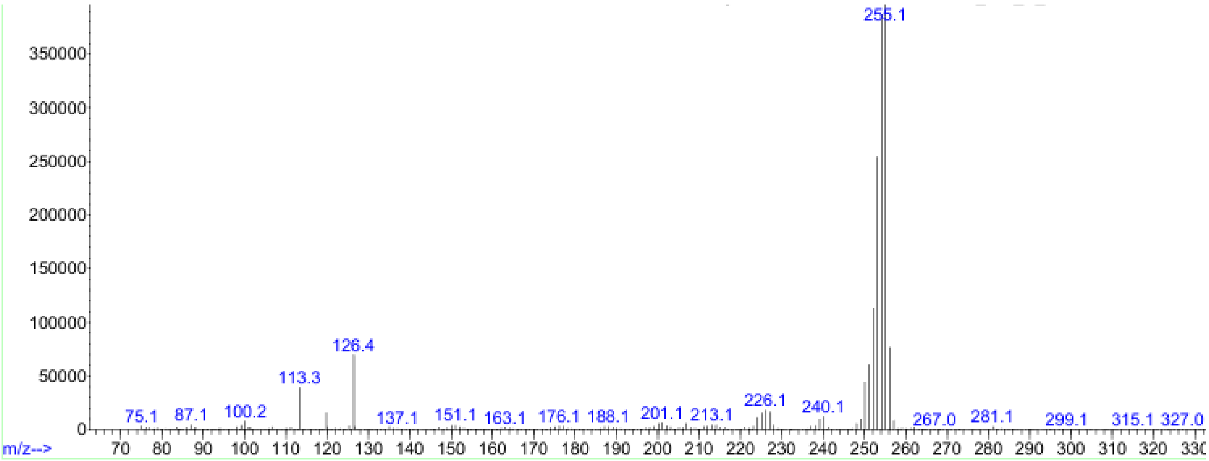

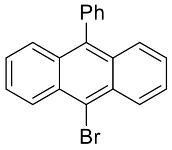

(Calcd.) *m/z*: 332.02 (100.0%), 334.02 (97.3%), 333.02 (21.6%), 335.02 (21.2%)

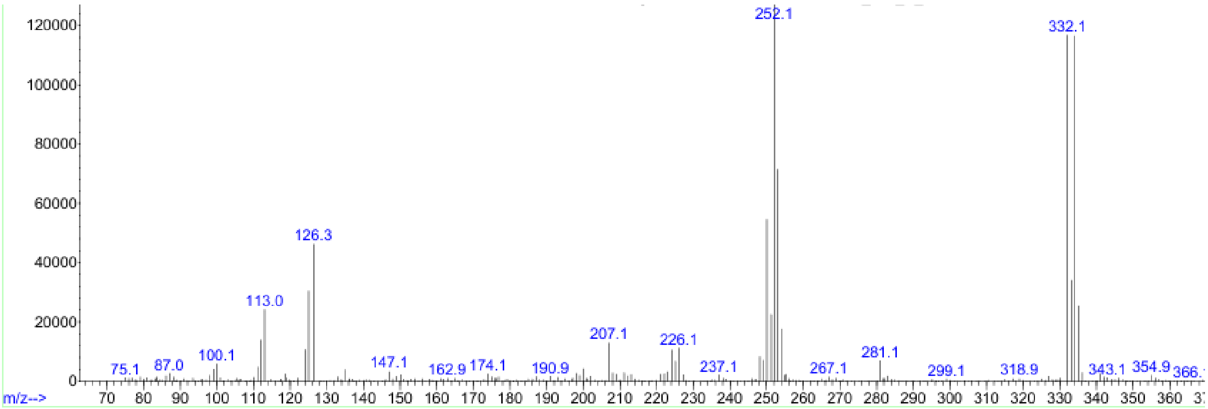

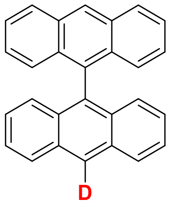

(Calcd.) *m/z*: 355.15 (100.0%), 356.15 (30.5%), 357.15 (4.4%)

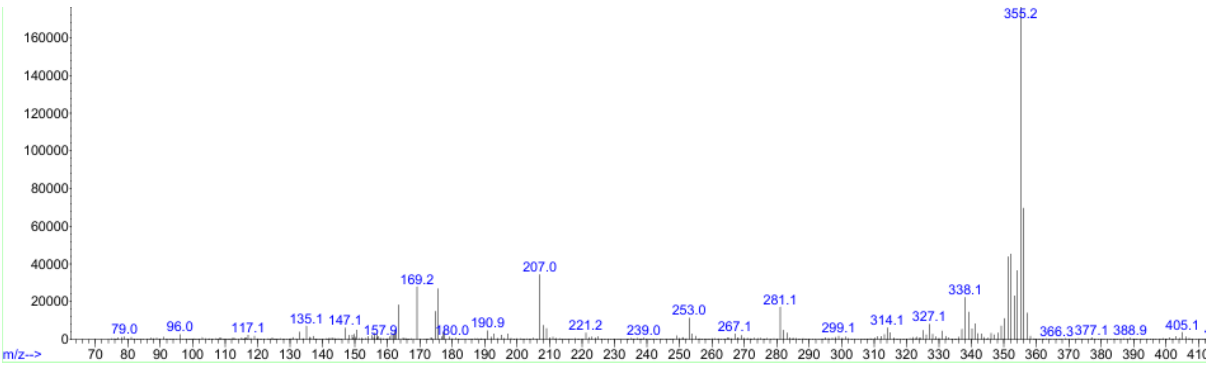

GCFID data for reaction of 40-d_1 including table quantitating components that had been separately calibrated.

Run 1

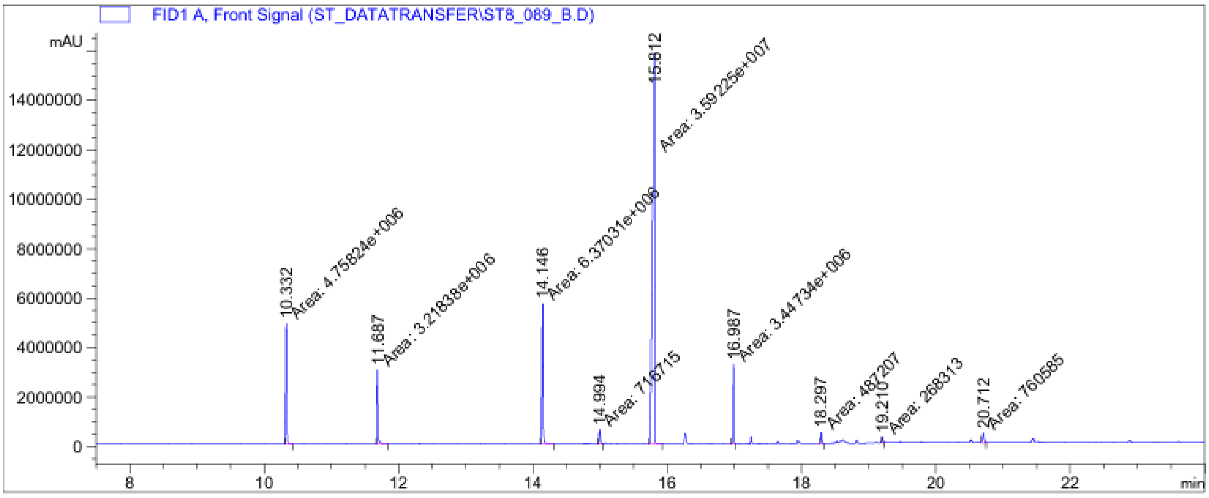

Retention Time (min)	Sample	Peak Area	%Yield
10.332	Dodecane	4758240	N/A
11.687	Biphenyl	3218380	7.6
14.146	Anthracene	6370310	11.4
14.994	Methylanthracene	716715	1.2
15.812	Bromoanthracene	35922500	61.2
16.987	Phenylanthracene	3447340	4.3
18.297	Bromophenylanthracene	487207	0.6
19.210	Diphenylanthracene	268313	0.3
20.712	Bianthracene	760585	1.4

Dodecane added = 7.1 mg

Run 2

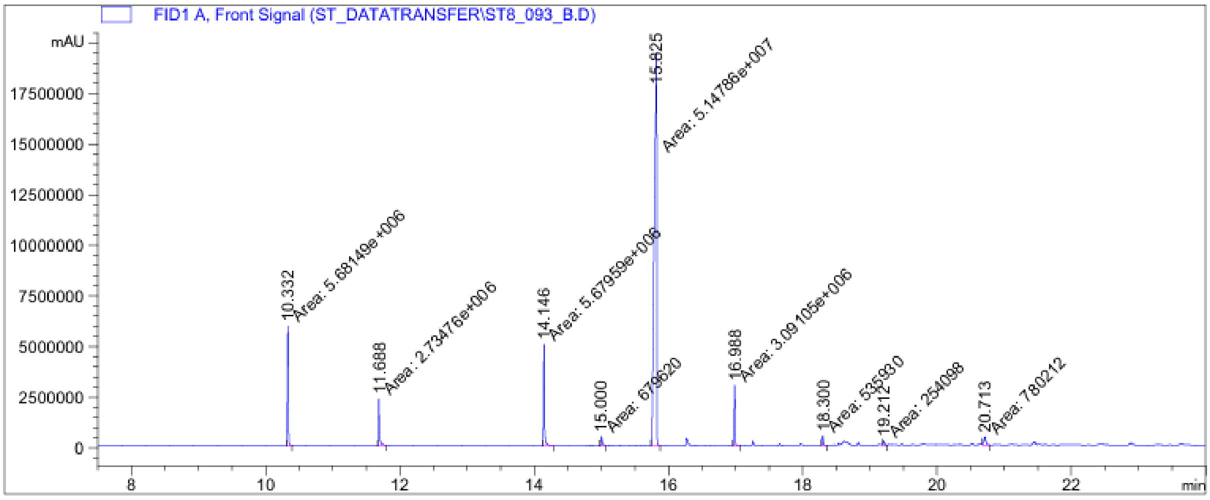

Retention Time (min)	Sample	Peak Area	%Yield
10.332	Dodecane	5681490	N/A
11.688	Biphenyl	2734760	4.9
14.146	Anthracene	5679590	7.7
15.000	Methylanthracene	679620	0.9
15.825	Bromoanthracene	51478600	16.2
16.988	Phenylanthracene	3091050	2.9
18.300	Bromophenylanthracene	535930	0.5
19.212	Diphenylanthracene	254098	0.2
20.713	Bianthracene	780212	1.0

Dodecane added = 6.4 mg

Run 3

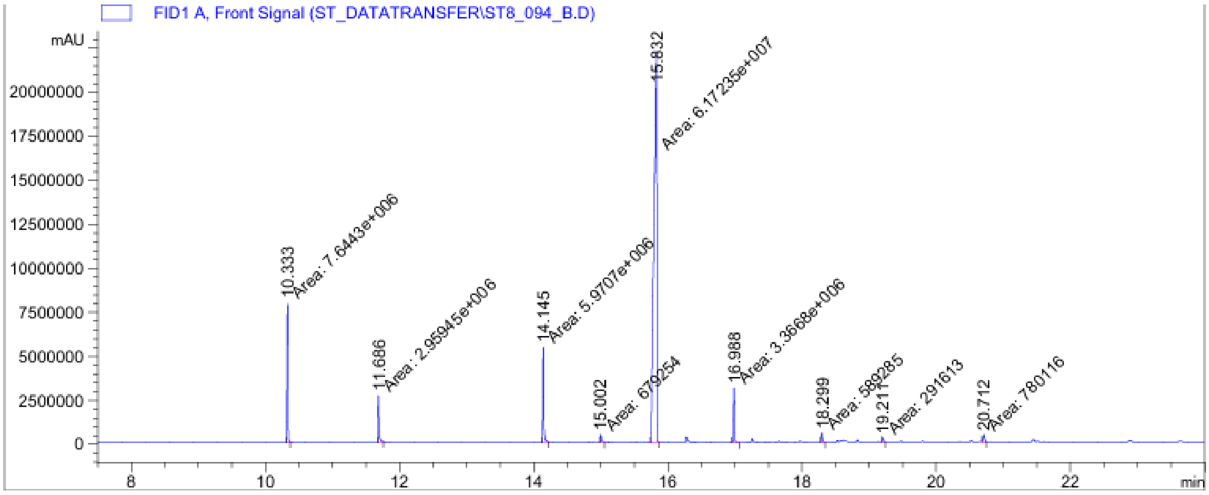

Retention Time (min)	Sample	Peak Area	%Yield
10.333	Dodecane	7644300	N/A
11.686	Biphenyl	2959450	4.6
14.145	Anthracene	5970700	6.9

15.002	Methylanthracene	679254	0.7
15.832	Bromoanthracene	61723500	68.3
16.988	Phenylanthracene	3366800	2.7
18.299	Bromophenylanthracene	589285	0.5
19.211	Diphenylanthracene	291613	0.2
20.712	Bianthracene	780116	1.0

Dodecane added = 7.4 mg

GC-MS data for reaction of 40-d_8 (Table 2, entry 3)

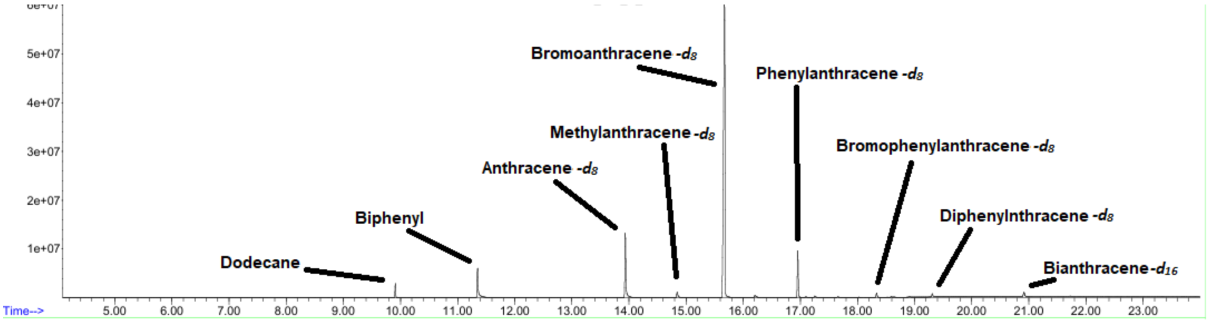

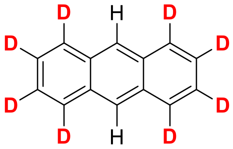

(Calcd.) *m/z*: 186.13 (100.0%), 187.13 (15.2%), 188.14 (1.1%)

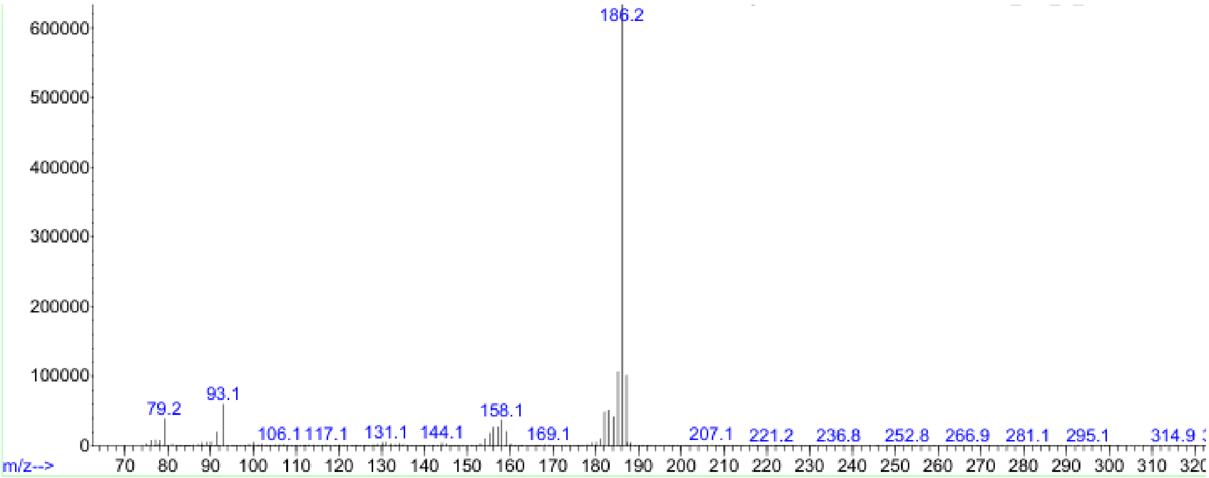

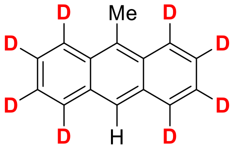

(Calcd.) *m/z*: 200.14 (100.0%), 201.15 (16.3%), 202.15 (1.2%)

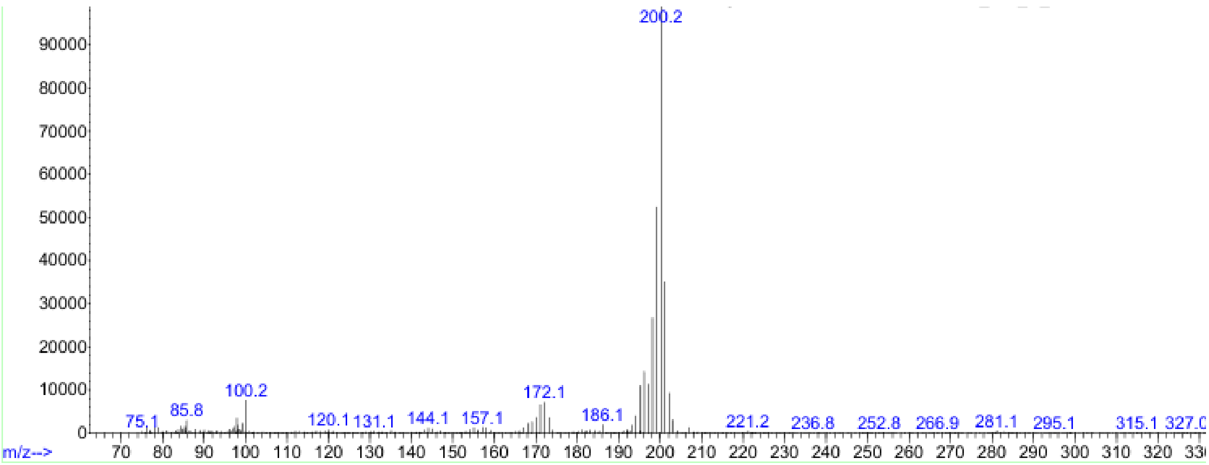

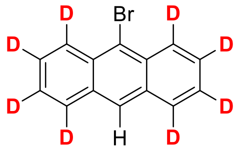

(Calcd.) *m/z*: 264.04 (100.0%), 266.04 (97.3%), 265.04 (15.1%), 267.04 (14.7%)

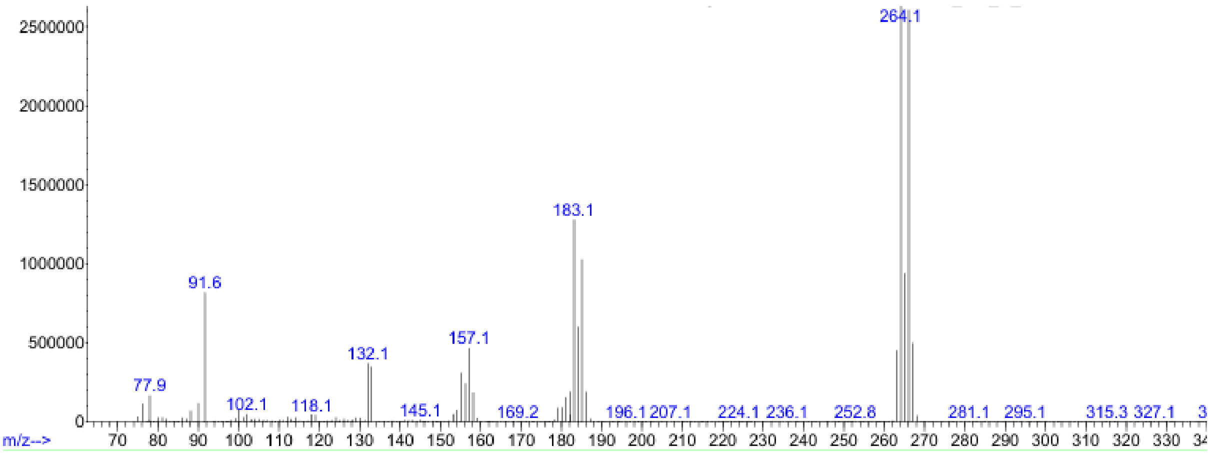

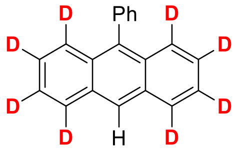

(Calcd.) *m/z*: 262.16 (100.0%), 263.16 (21.6%), 264.17 (2.2%)

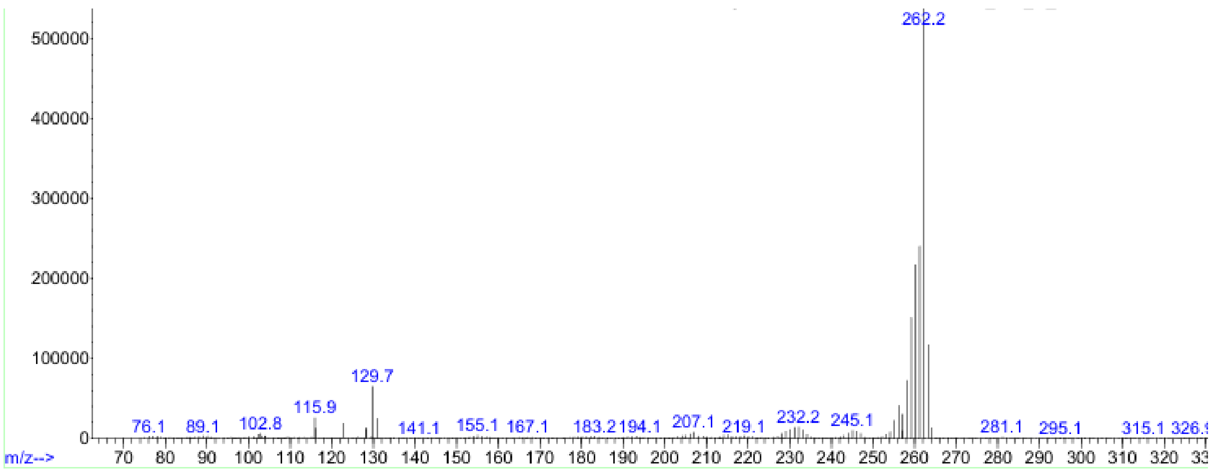

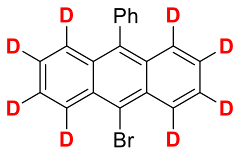

(Calcd.) *m/z*: 340.07 (100.0%), 342.07 (97.3%), 341.07 (21.6%), 343.07 (21.1%)

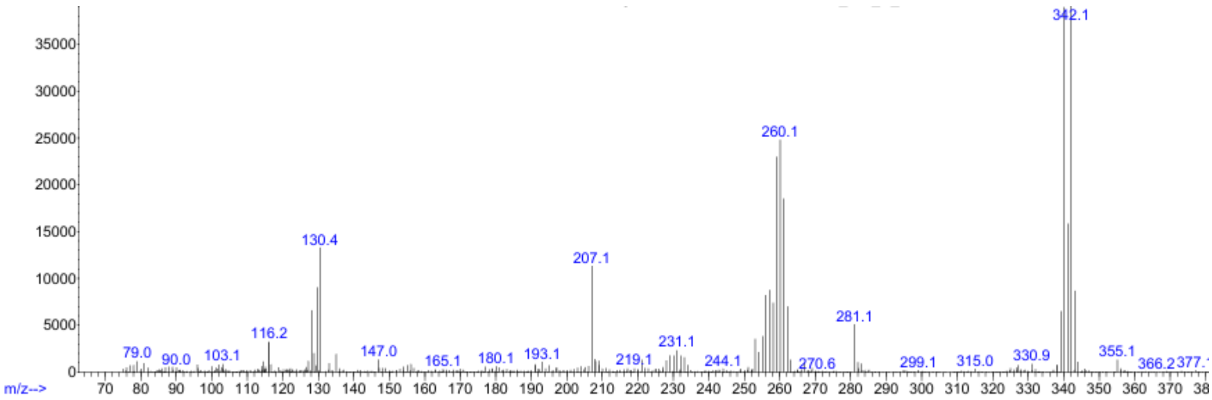

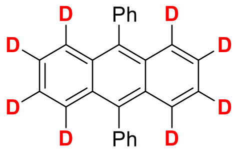

(Calcd.) *m/z*: 338.19 (100.0%), 339.19 (28.1%), 340.20 (3.8%)

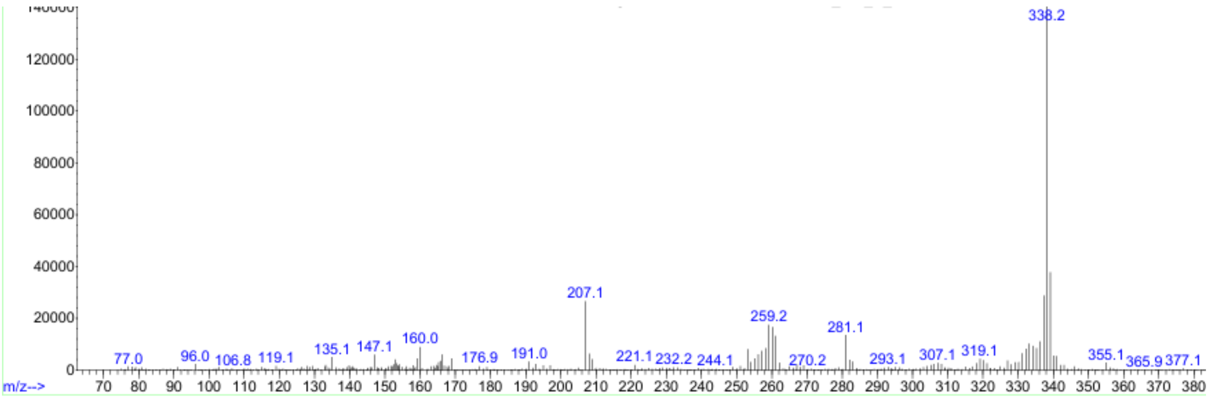

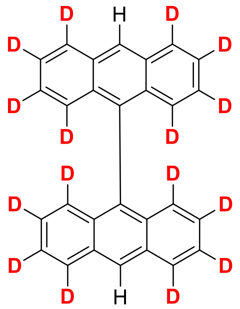

(Calcd.) *m/z*: 370.24 (100.0%), 371.24 (30.3%), 372.25 (4.4%)

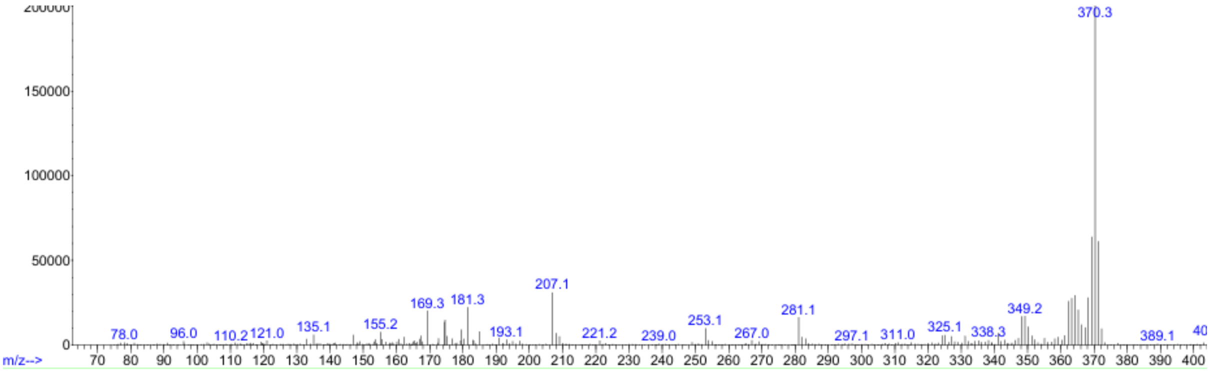

GCFID data for reaction of 40-d_8 including table quantitating components that had been separately calibrated.

Run 1

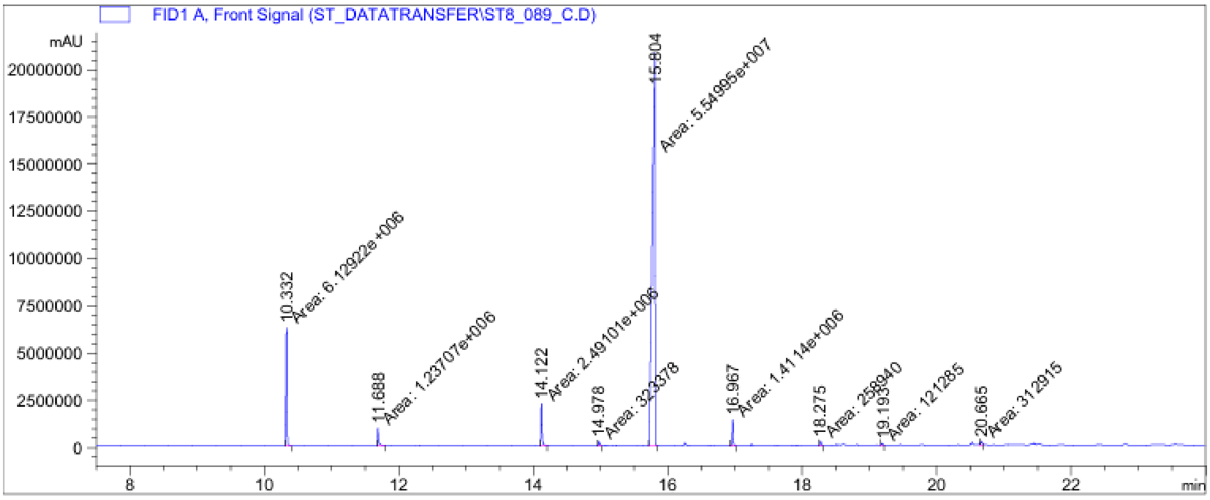

Retention Time (min)	Sample	Peak Area	%Yield
10.332	Dodecane	6129220	N/A
11.688	Biphenyl	1237070	2.3
14.122	Anthracene	2491010	3.5
14.978	Methylanthracene	323378	0.4
15.804	Bromoanthracene	55499500	73.4
16.967	Phenylanthracene	1411400	1.4
18.275	Bromophenylanthracene	258940	0.2
19.193	Diphenylanthracene	121285	0.09
20.665	Bianthracene	312915	0.4

Dodecane added = 7.1 mg

Run 2

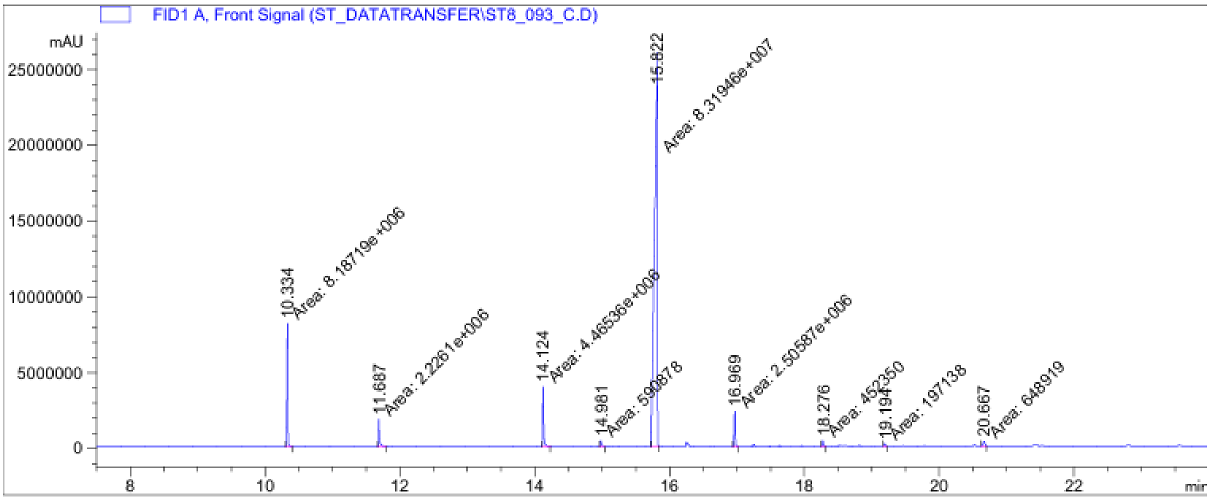

Retention Time (min)	Sample	Peak Area	%Yield
10.334	Dodecane	8187190	N/A
11.687	Biphenyl	2226100	2.9
14.124	Anthracene	4465360	4.3
14.981	Methylanthracene	590878	0.5
15.822	Bromoanthracene	83194600	76.6
16.969	Phenylanthracene	2505870	1.7
18.276	Bromophenylanthracene	452350	0.3
19.194	Diphenylanthracene	197138	0.1
20.667	Bianthracene	648919	0.6

Dodecane added = 6.6 mg

Run 3

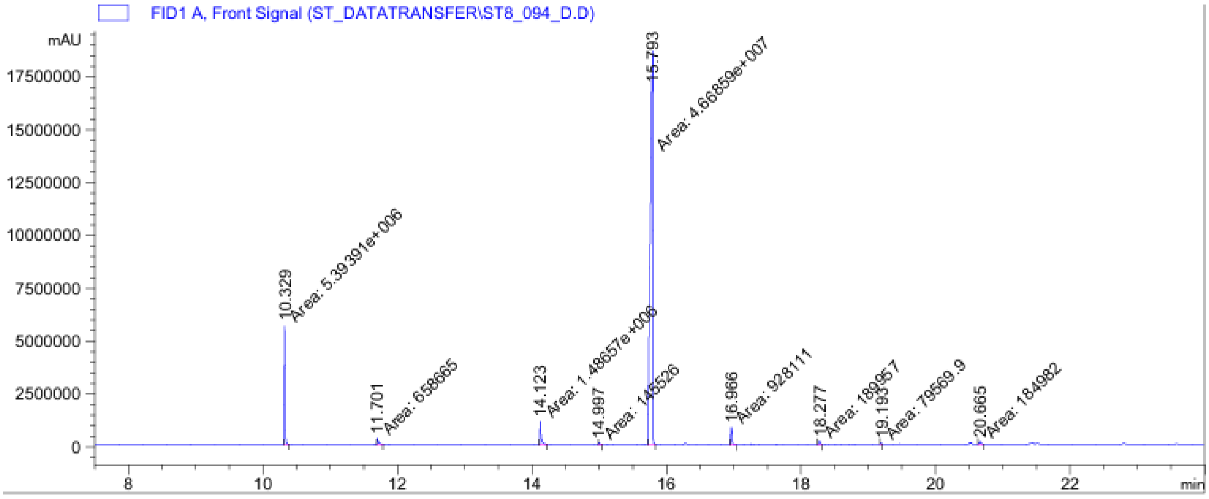

Retention Time (min)	Sample	Peak Area	%Yield
10.329	Dodecane	5393910	N/A
11.701	Biphenyl	658665	1.5
14.123	Anthracene	1486570	2.6
14.997	Methylanthracene	145526	0.2
15.793	Bromoanthracene	46685900	77.1
16.966	Phenylanthracene	928111	1.1
18.277	Bromophenylanthracene	189957	0.2
19.193	Diphenylanthracene	79570	0.07
20.665	Bianthracene	184982	0.4

Dodecane added = 7.8 mg

GC-MS data for reaction of 40-d_9 (Table 2, entry 4)

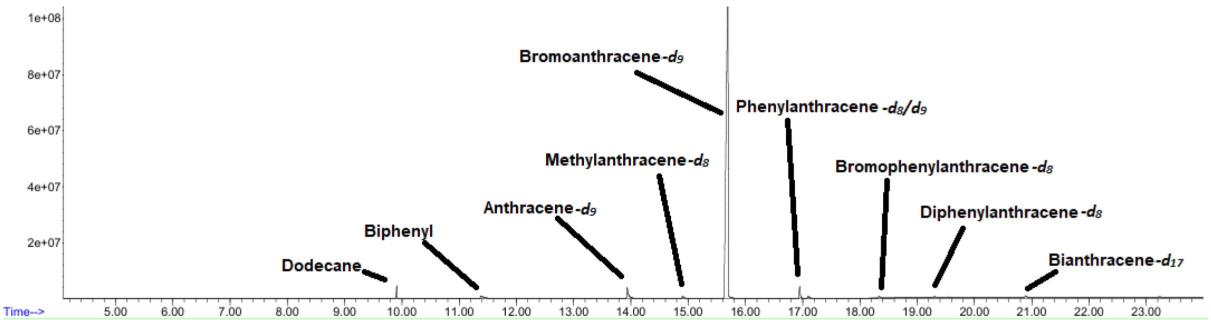

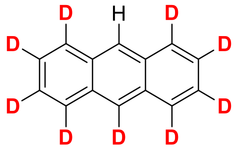

(Calcd.) *m/z*: 187.13 (100.0%), 188.14 (15.2%), 189.14 (1.1%)

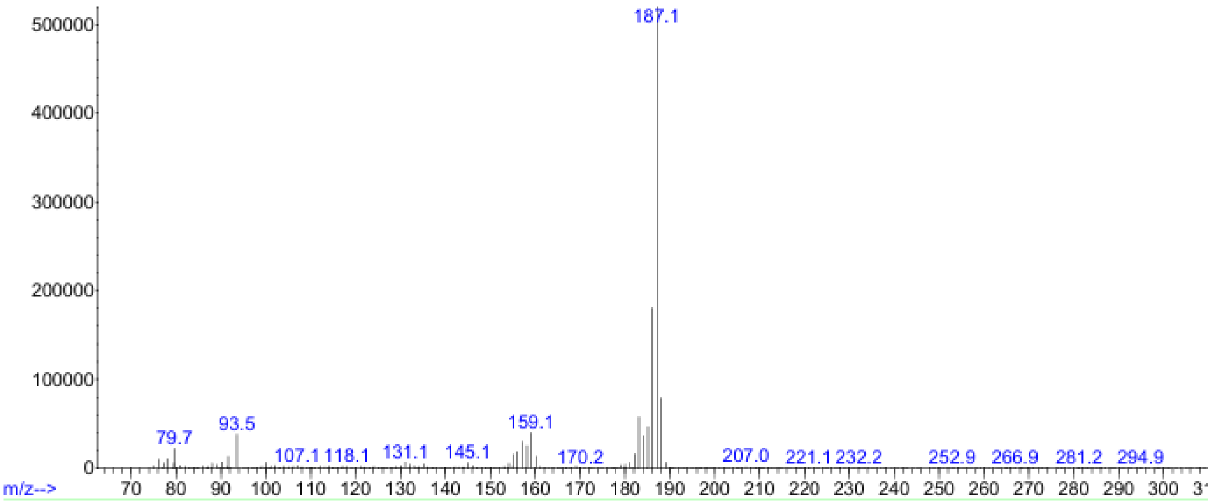

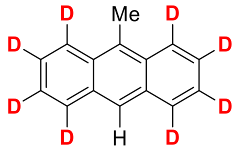

(Calcd.) *m/z*: 200.14 (100.0%), 201.15 (16.3%), 202.15 (1.2%)

Shows a mixture of 9-methylanthracene-d_8 and a small amount of 9-methylanthracene-d_9, resulting from either *para*- or *ipso*-attack of a methyl radical on bromoanthracene-d_9

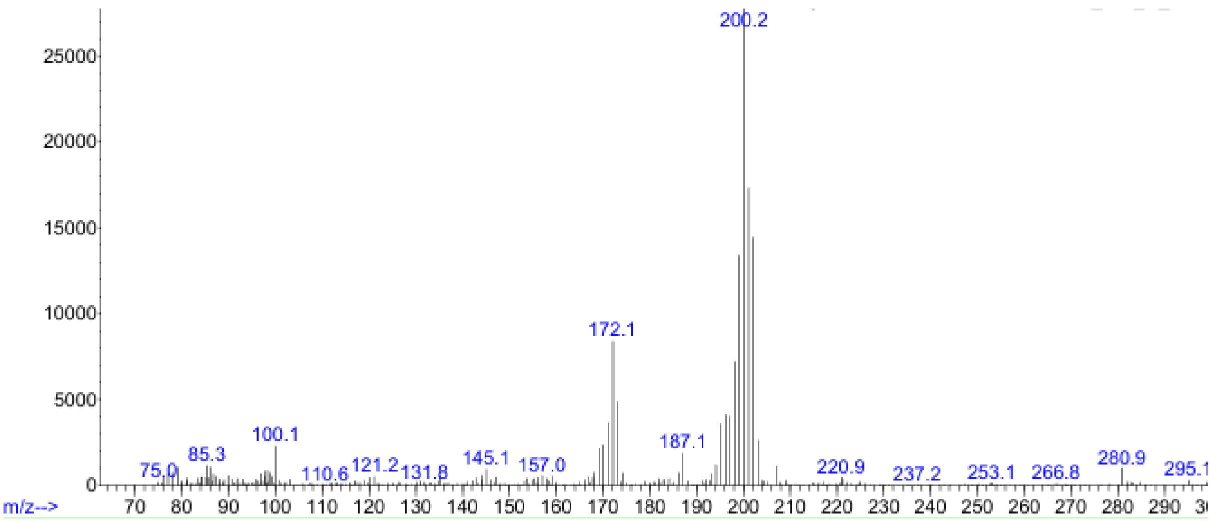

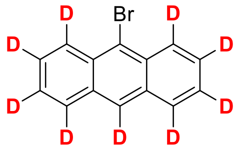

(Calcd.) *m/z*: 265.05 (100.0%), 267.04 (97.3%), 266.05 (15.1%), 268.05 (14.7%)

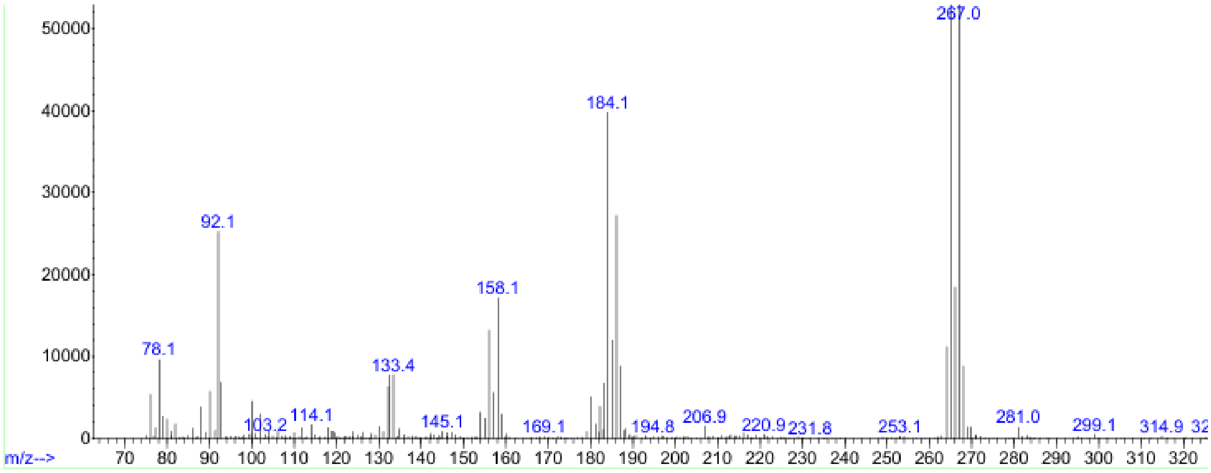

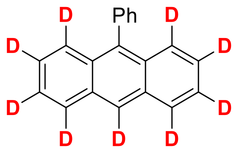

(Calcd.) *m/z*: 263.17 (100.0%), 264.17 (21.7%), 265.17 (2.2%)

Shows a mixture of 9-phenylanthracene-d_8 and 9-phenylanthracene-d_9, resulting from (i) attack of an anthracenyl-d_9 radical on benzene (d_9) or ipso attack of a phenyl radical on bromoanthracene-d_9, or (ii) *para* attack of a phenyl radical on bromoanthracene-d_9 (d_8)

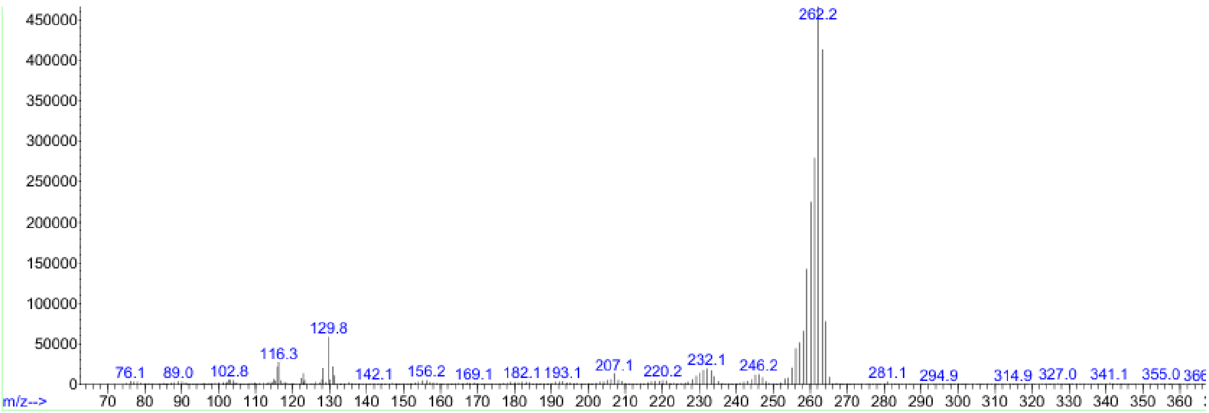

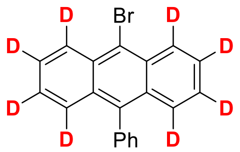

(Calcd.) *m/z*: 340.07 (100.0%), 342.07 (97.3%), 341.07 (21.6%), 343.07 (21.1%)

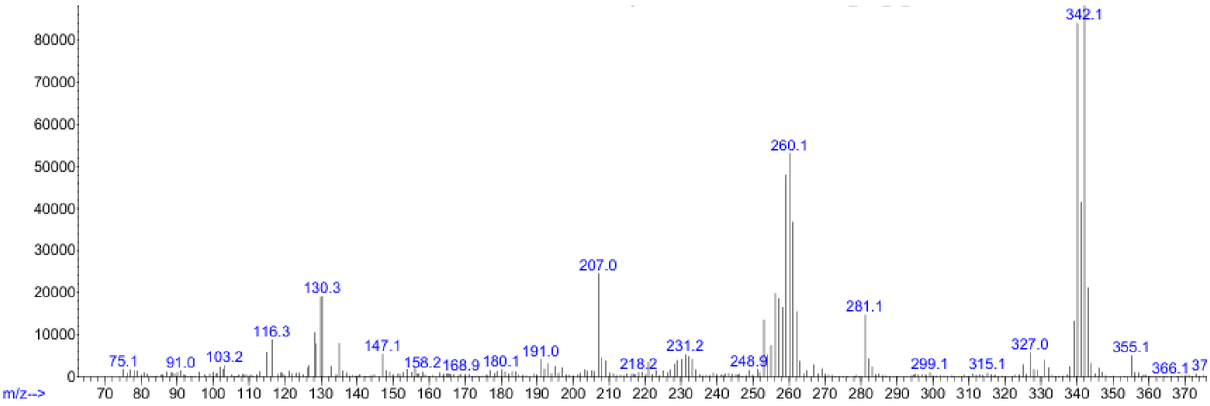

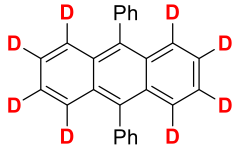

(Calcd.) *m/z*: 338.19 (100.0%), 339.19 (28.1%), 340.20 (3.8%)

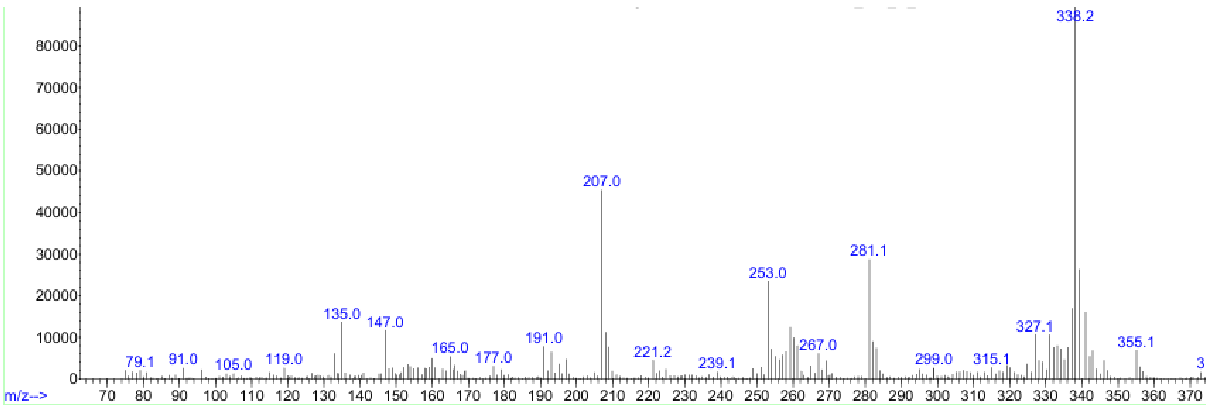

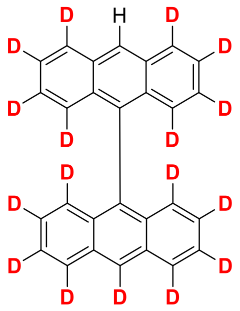

(Calcd.) *m/z*: 371.25 (100.0%), 372.25 (30.3%), 373.25 (4.4%)

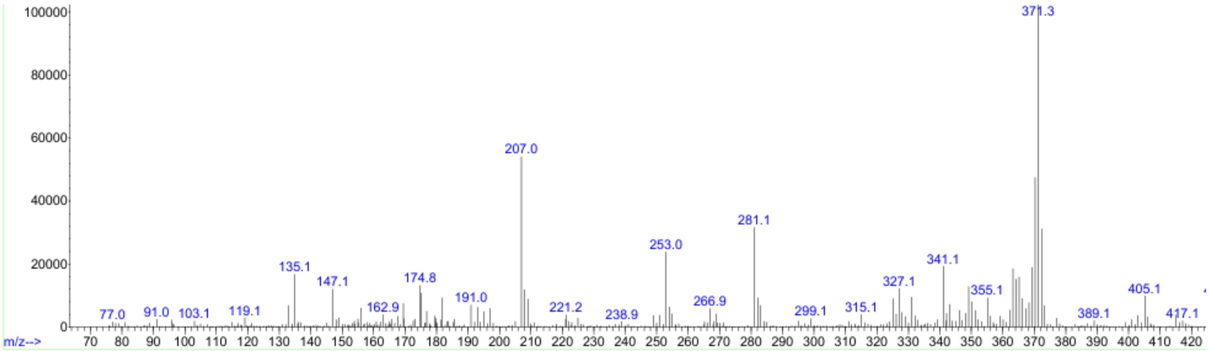

GCFID data for reaction of 40-d_9 including table quantitating components that had been separately calibrated.

Run 1

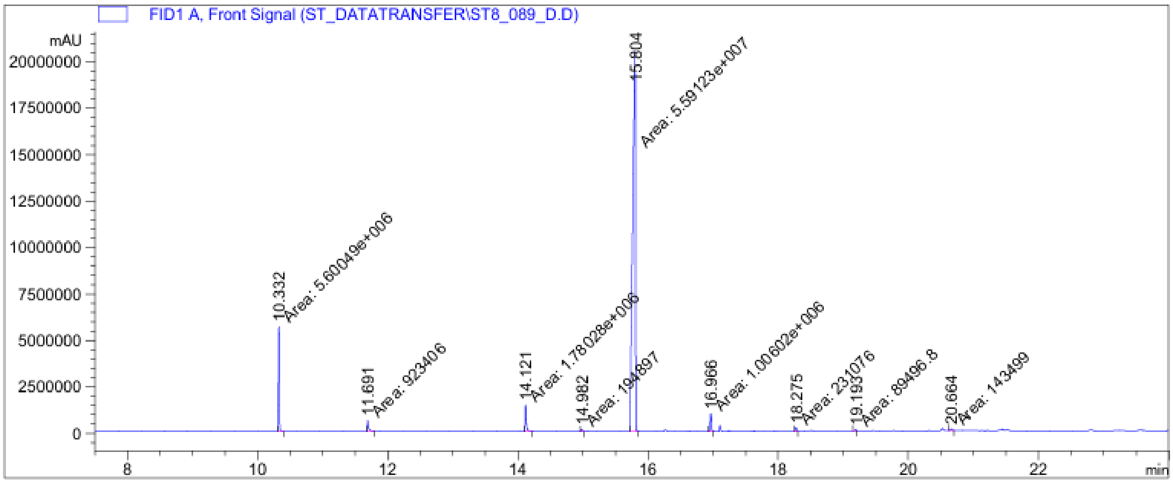

Retention Time (min)	Sample	Peak Area	%Yield
10.332	Dodecane	5600490	N/A
11.691	Biphenyl	923406	1.8
14.121	Anthracene	1780280	2.7
14.982	Methylanthracene	194897	0.3
15.804	Bromoanthracene	55912300	79.8
16.966	Phenylanthracene	1006020	1.1
18.275	Bromophenylanthracene	231076	0.2
19.193	Diphenylanthracene	89496	0.07
20.664	Bianthracene	143499	0.2

Dodecane added = 7.0 mg

Run 2

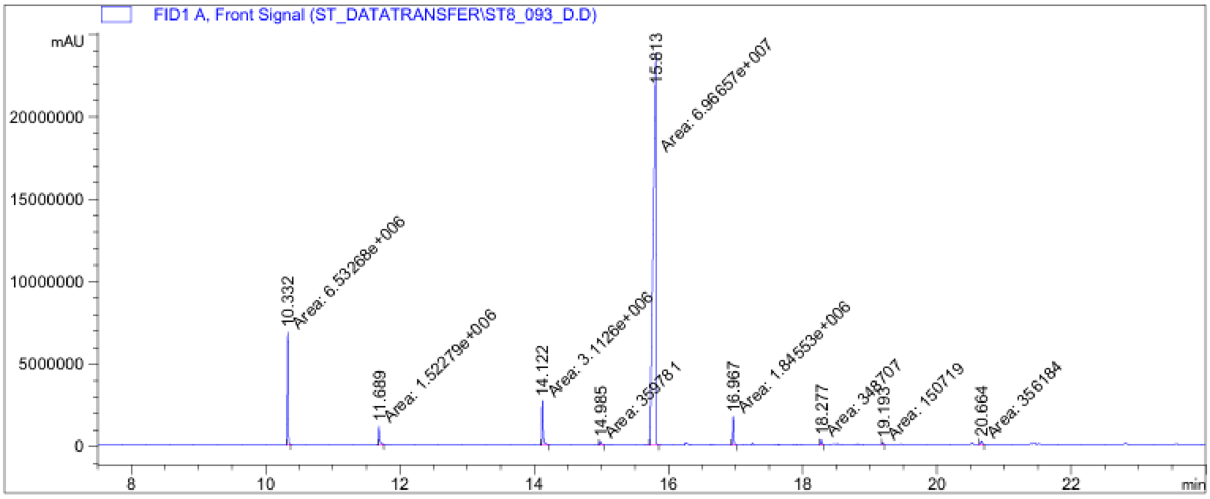

Retention Time (min)	Sample	Peak Area	%Yield
10.332	Dodecane	6532680	N/A
11.689	Biphenyl	1522790	2.4
14.122	Anthracene	3112600	3.7
14.985	Methylanthracene	359781	0.4
15.813	Bromoanthracene	69665700	79.2
16.967	Phenylanthracene	1845530	1.5
18.277	Bromophenylanthracene	348707	0.3
19.193	Diphenylanthracene	150719	0.1
20.664	Bianthracene	356184	0.4

Dodecane added = 6.5 mg

Run 3

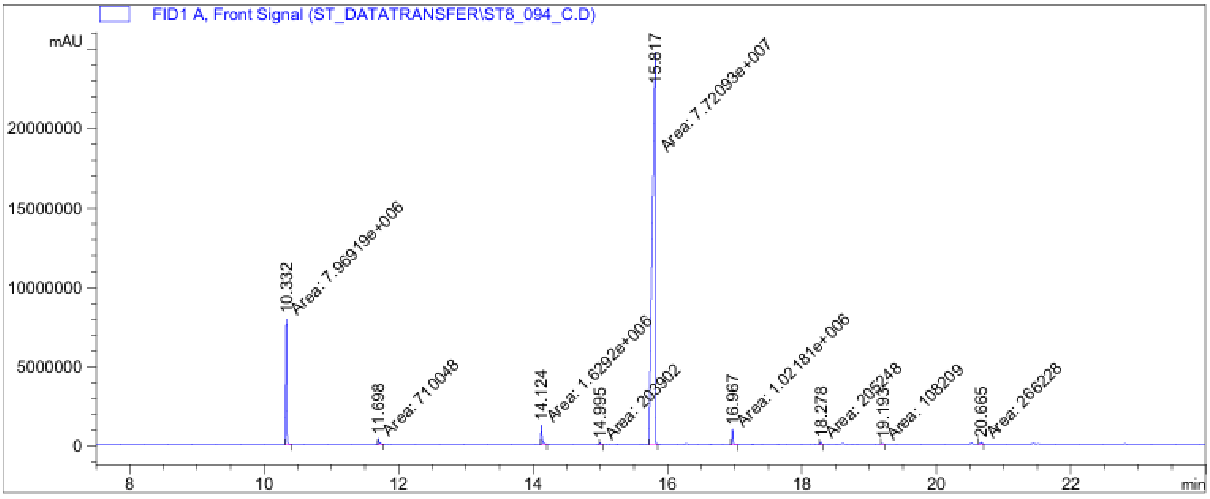

Retention Time (min)	Sample	Peak Area	%Yield
10.332	Dodecane	7969190	N/A
11.698	Biphenyl	710048	1.1
14.124	Anthracene	1629200	1.9

14.995	Methylanthracene	203902	0.2
15.617	Bromoanthracene	77209300	84.1
16.967	Phenylanthracene	1021810	0.8
18.278	Bromophenylanthracene	205248	0.2
19.193	Diphenylanthracene	108209	0.07
20.665	Bianthracene	266228	0.3

Dodecane added = 7.6 mg

Reactions of 9-bromoanthracene 40 with KO*t*Bu and TEMPO in C_6H_6

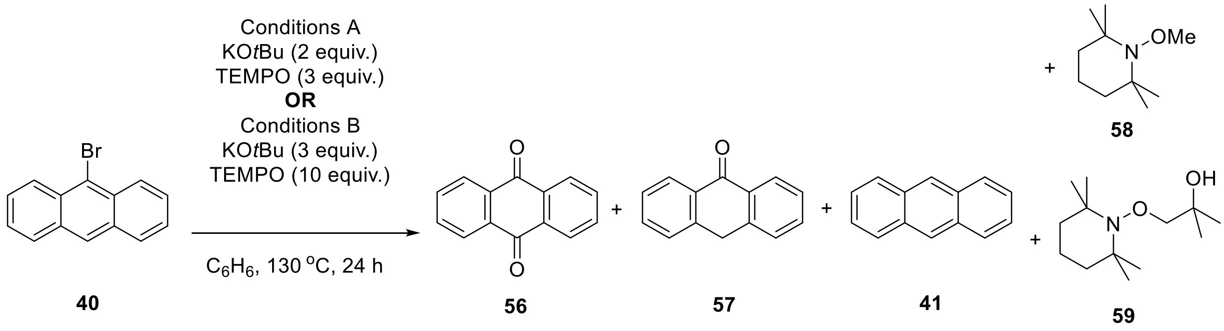

This reaction was carried out twice with differing amounts of KO*t*Bu and TEMPO (Conditions A and Conditions B). To an oven-dried microwave vial, primed with a stirrer bar, in a glovebox was added 9-bromoanthracene **40** (90 mg, 0.35 mmol, 1 equiv.), KO*t*Bu (Conditions A = 79 mg, 0.7 mmol, 2 equiv. OR Conditions B = 118 mg, 1.05 mmol, 3 equiv.), TEMPO (Conditions A = 164 mg, 1.05 mmol, 3 equiv. OR Conditions B = 547 mg, 3.5 mmol, 10 equiv.) and benzene (3.5 mL) with the vial subsequently sealed and stirred at 130 °C in a pre-heated oil bath for 45 mins. Once complete, the crude mixture was quickly cooled to room temperature, D_2O (0.2 mL) and an accurately weighed mass of *n*-dodecane in EtOAc (5 mL) was added. An aliquot of the crude mixture was then analysed by both GCMS and GC-FID (Method 2). Quantification of products could not be carried out due to the overlap of an unknown peak with *n*-dodecane in GC-FID spectrum.

GC-MS data for Conditions A (lower trace is a vertical expansion)

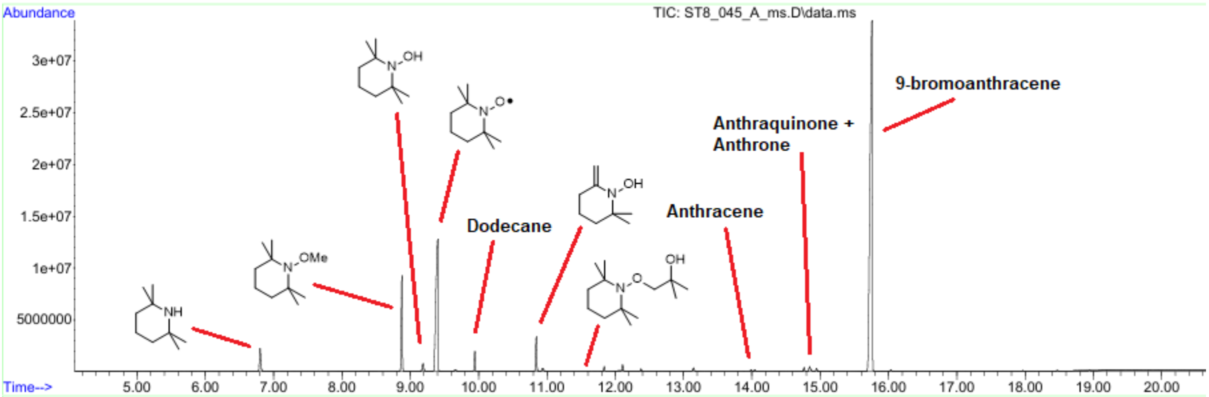

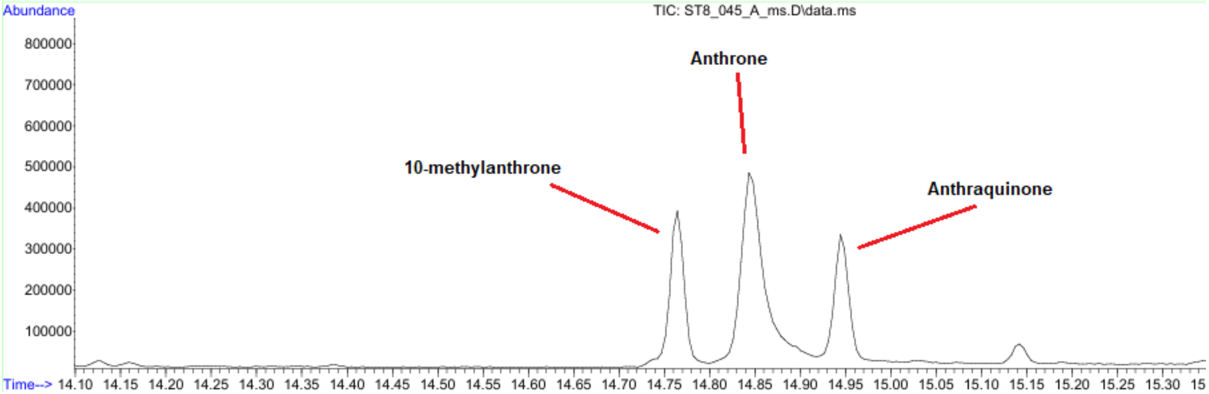

The GC-MS of the crude reaction mixture from this reaction shows consumption of the substrate 9-bromoanthracene **40** has been almost entirely supressed, although small amounts of anthracene **41** are observed. An interesting cluster of peaks corresponding to anthraquinone **56** and anthrone **57** (retention times confirmed by comparison to authentic samples), along with 10-methylanthrone (or an isomer thereof). Along with residual TEMPO, TEMPO-H and 2,2,6,6-tetramethylpiperidine, a peak corresponding to TEMPO-Me **58** is also observed. While this could arise through trapping of methyl radicals by TEMPO, it has also been shown to arise through thermal decomposition of TEMPO producing methyl radicals along with an alkene as a byproduct,[60] for which a peak with the corresponding mass was also observed. Additionally, a trace peak was observed with *m/z* 229 was observed, corresponding to TEMPO–*tert*-butanol adduct **59**, providing evidence for HAT from the methyl groups of butoxide.

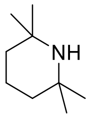

(Calc.) *m/z*: 141.1517 (100.0%), 142.1551 (9.7%)

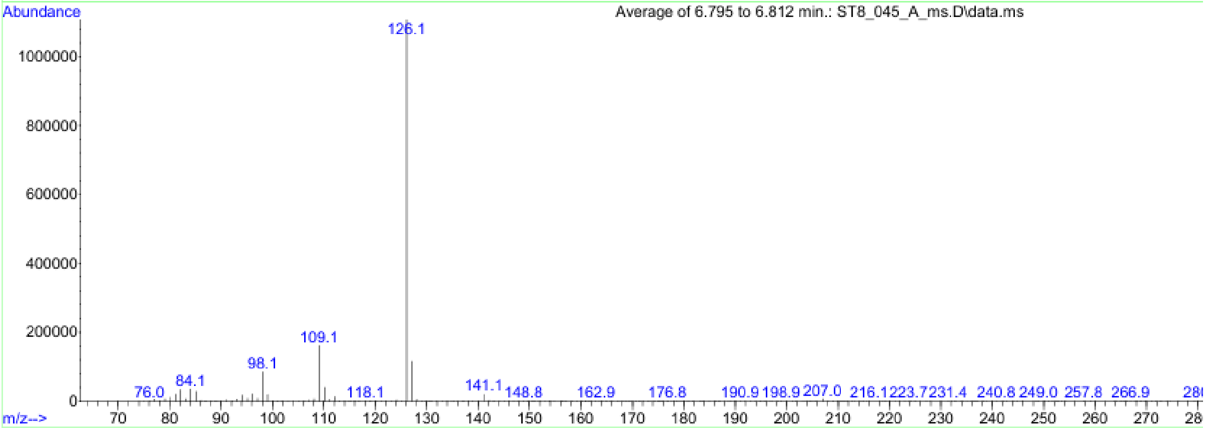

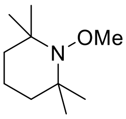

(Calc.) *m/z*: 171.1623 (100.0%), 172.1657 (10.8%)

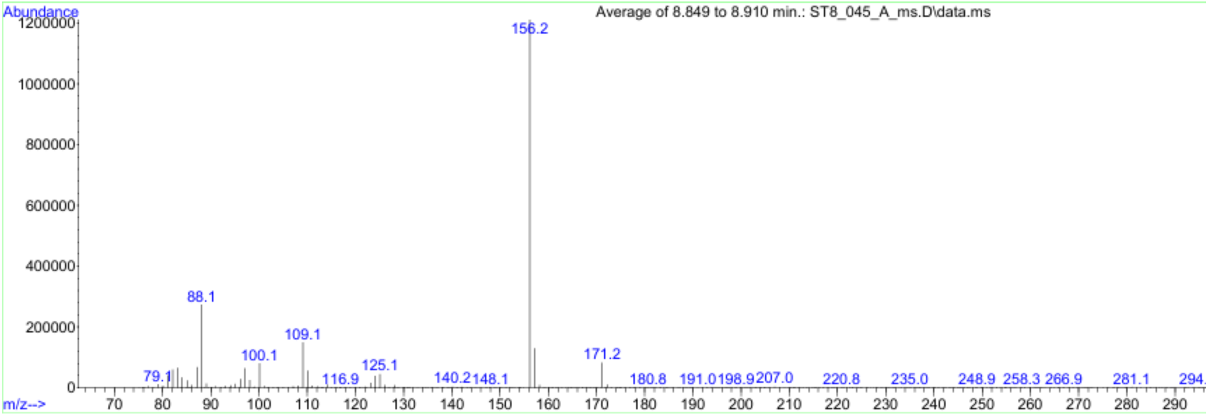

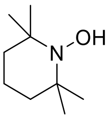

(Calc.) *m/z*: 157.1467 (100.0%), 158.1500 (9.7%)

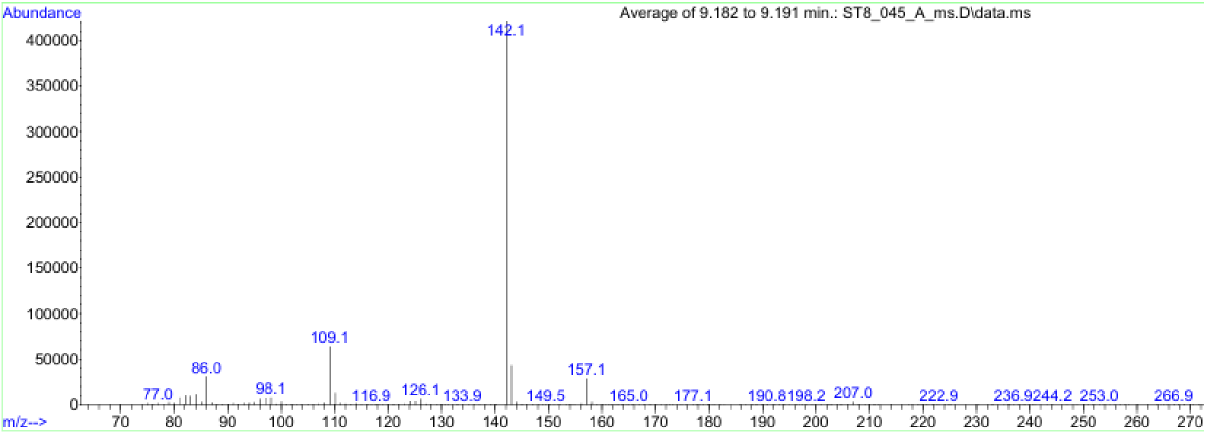

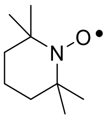

(Calc.) *m/z*: 156.1388 (100.0%), 157.1422 (9.7%)

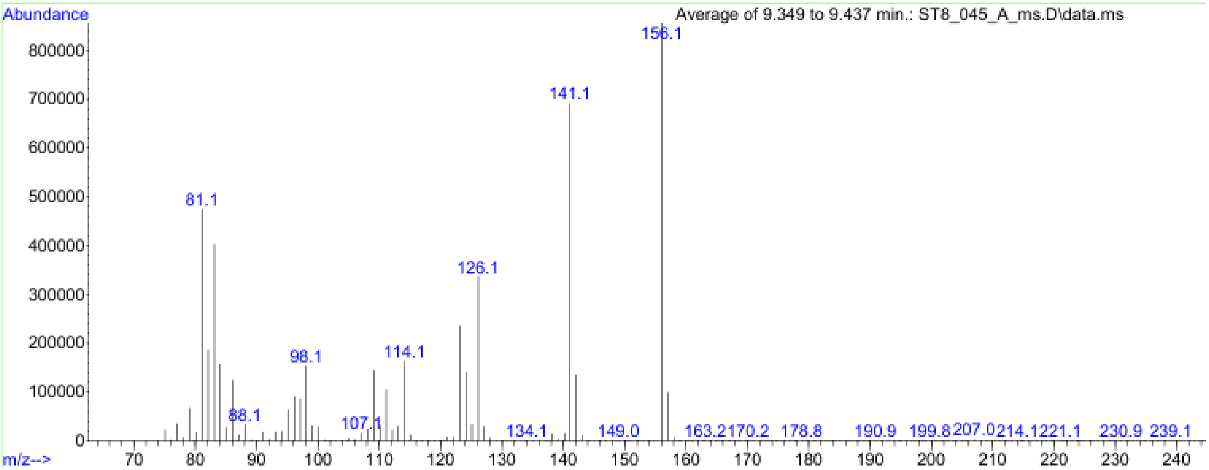

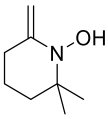

(Calc.) *m/z*: 141.1154 (100.0%), 142.1187 (8.7%)

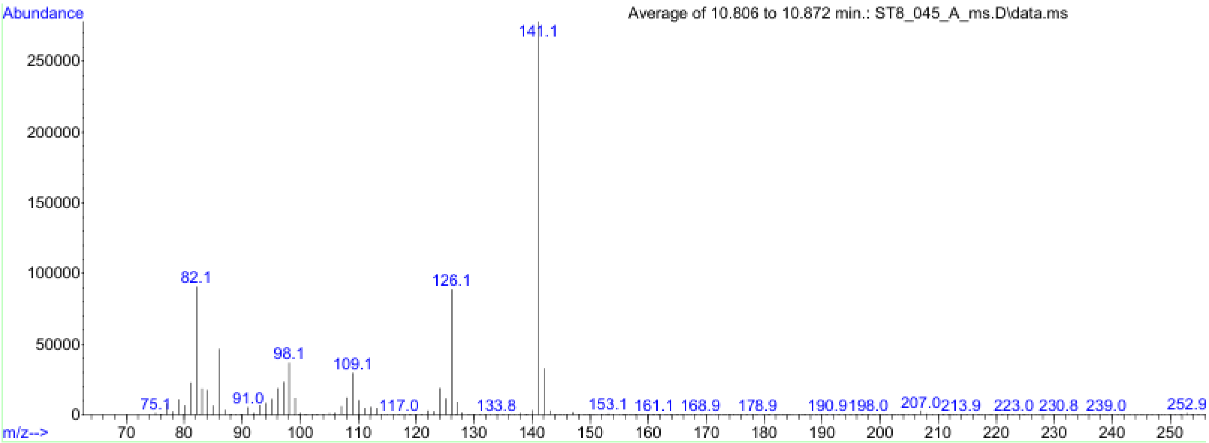

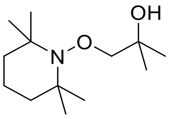

(Calc.) *m/z*: 229.2042 (100.0%), 230.2075 (14.1%)

Compound **59** - **HRMS** (ESI+) [*m/z*] calcd. for $C_{13}H_{28}NO_2$ (M+H$^+$) 230.2114, found 230.2111.

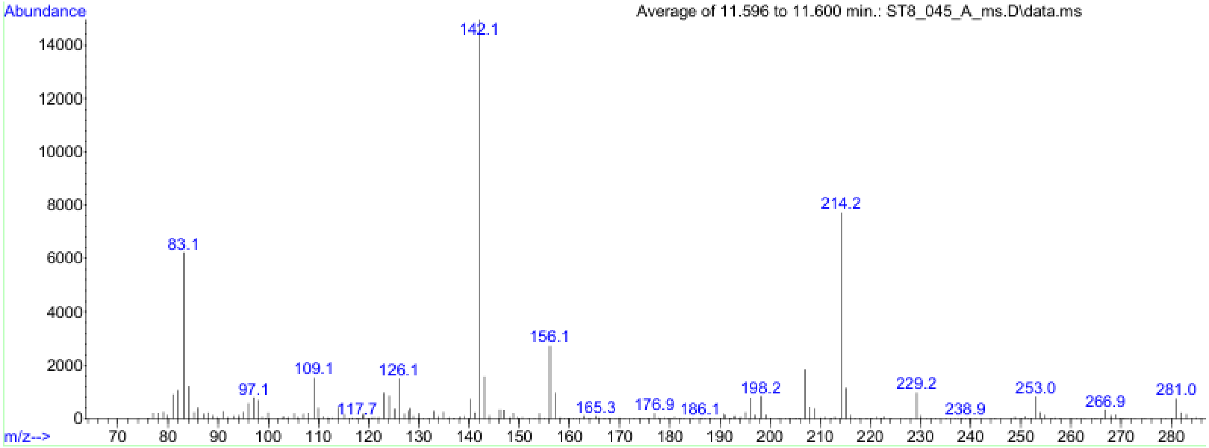

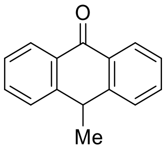

(Calc.) *m/z*: 208.0888 (100.0%), 209.0922 (16.2%), 210.0955 (1.2%)

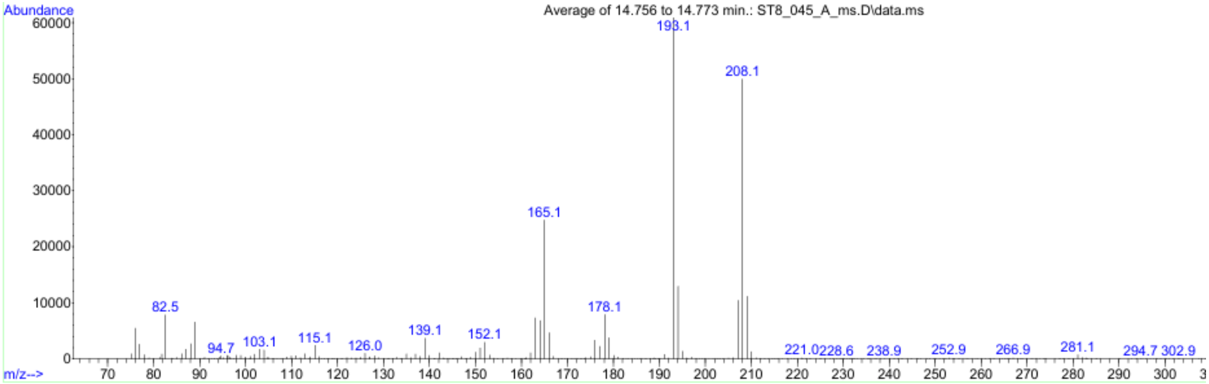

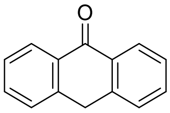

(Calc.) *m/z*: 194.0732 (100.0%), 195.0765 (15.1%), 196.0799 (1.1%)

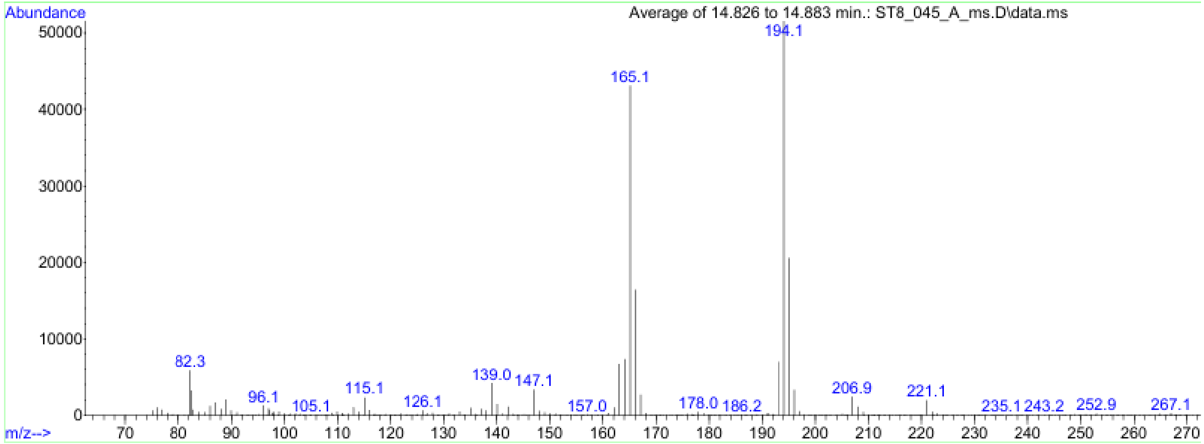

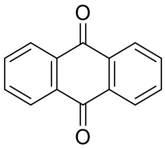

(Calc.) *m/z*: 208.0524 (100.0%), 209.0558 (15.1%), 210.0591 (1.1%)

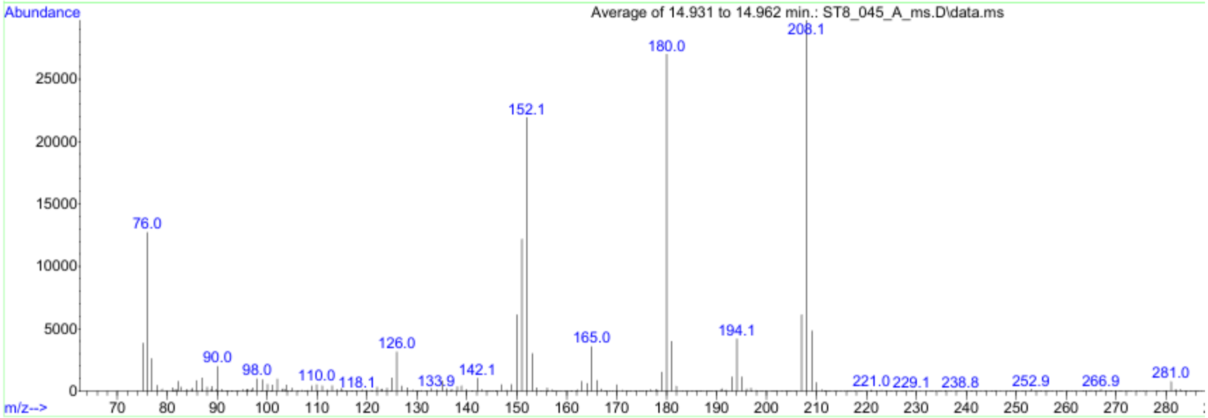

GC-MS data for Conditions B

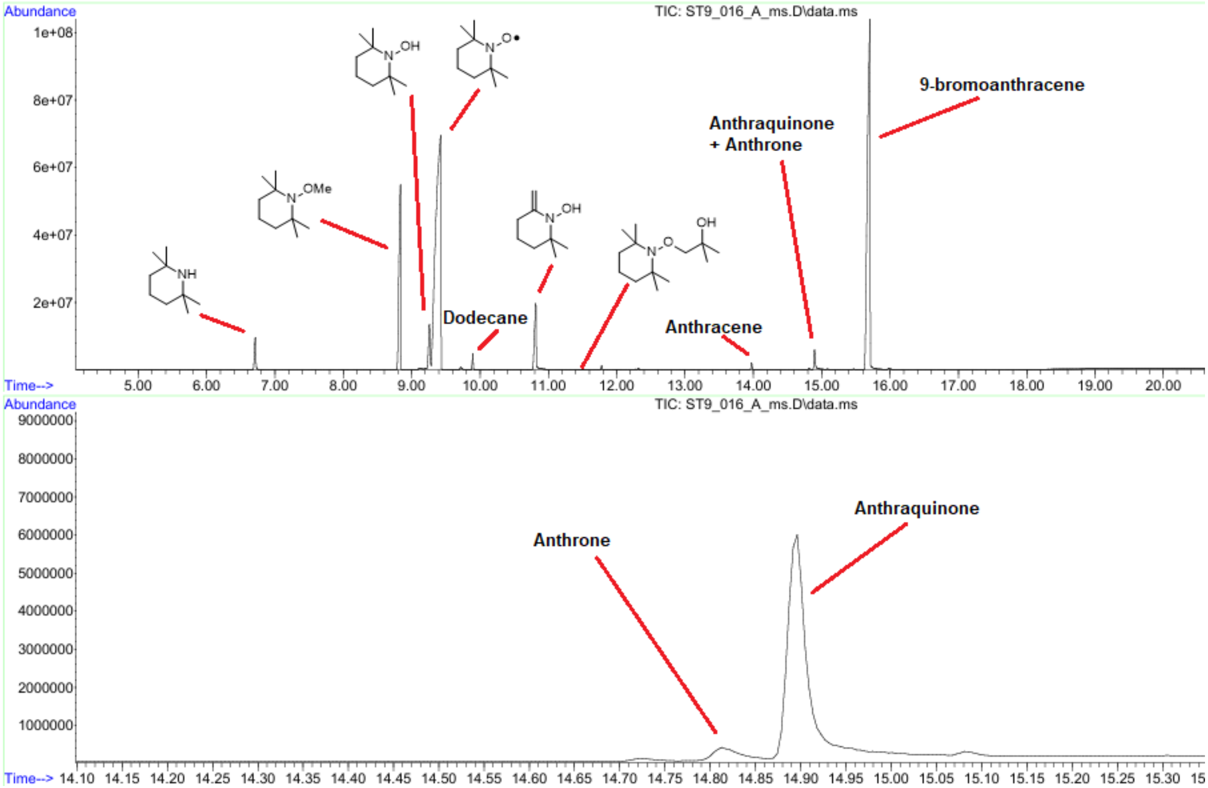

The GC-MS of the crude mixture from this reaction shows a similar reaction profile, with 9-bromoanthracene remaining mostly unreacted and traces of anthracene formed. We also see traces of TEMPO-butoxy adduct **59**. However, compared to the reaction under Conditions A, the ratio of anthraquinone **56** : anthrone **57** is much higher here, likely due to a higher concentration of TEMPO leading to more efficient trapping of *para*-benzyne.

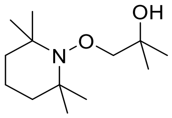

(Calc.) *m/z*: 229.2042 (100.0%), 230.2075 (14.1%)

Compound **59** - **HRMS** (ESI+) [*m/z*] calcd. for $C_{13}H_{28}NO_2$ (M+H+) 230.21146, found 230.2111.

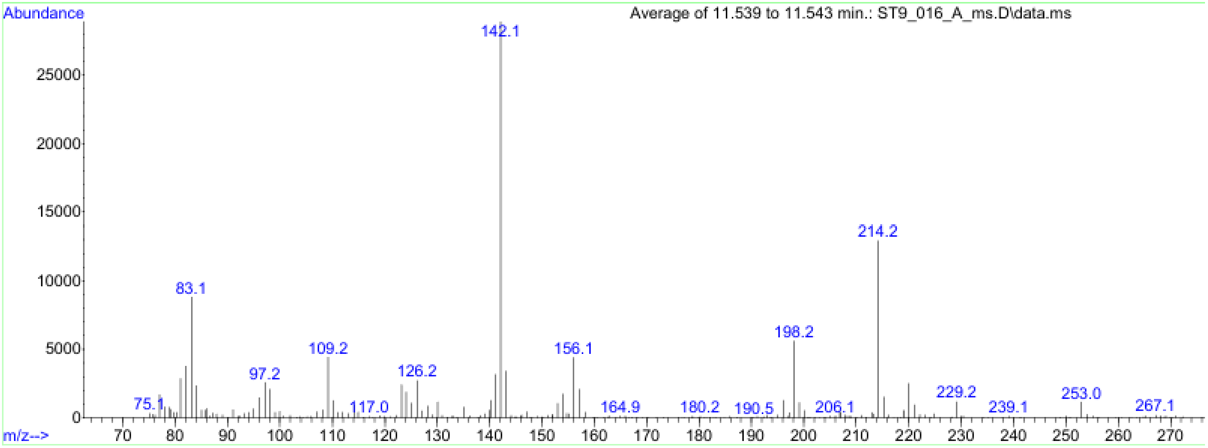

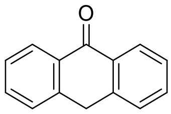

(Calc.) *m/z*: 194.0732 (100.0%), 195.0765 (15.1%), 196.0799 (1.1%)

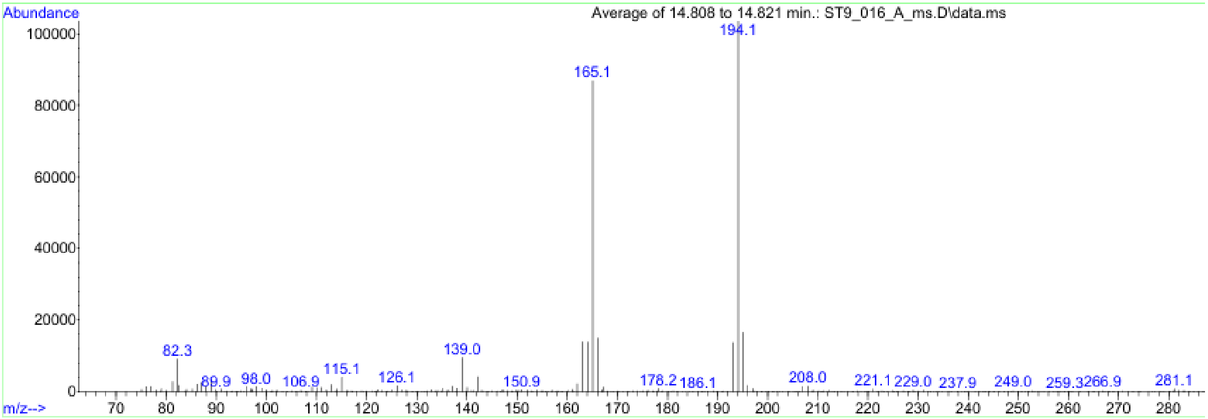

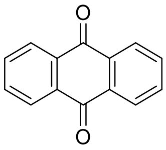

(Calc.) *m/z*: 208.0524 (100.0%), 209.0558 (15.1%), 210.0591 (1.1%)

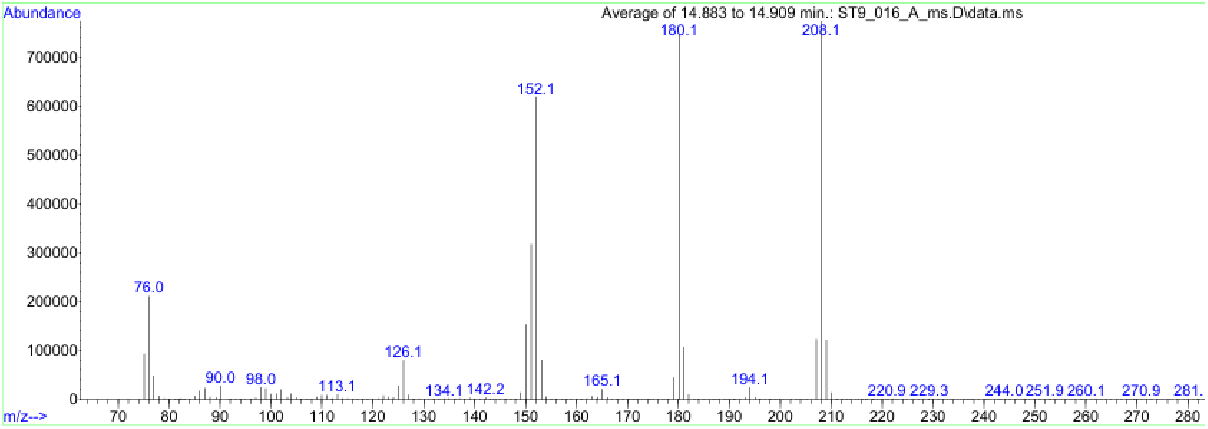

Methylanthracene isomer determination

To determine which isomer of methylanthracene was forming in our reactions, ^{1}H NMR of the crude reaction below in $CDCl_3$, was taken. This was then compared with a commercial sample of 9-methylanthracene and references in +the literature.[61] From the spectra below, it can be seen that the methyl peak in the pure sample of 9-methylanthracene matches the singlet in the crude spectrum from the reaction (9-methylanthracene 3.12 ppm; 1-methylanthracene, 2.85 ppm; 2-

methylanthracene, 2.53 ppm). This, along with the evidence from the literature, suggests that 9-methylanthracene is the main isomer formed in our reactions.

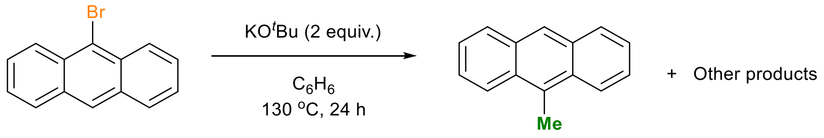

40 **45**, 3.1%

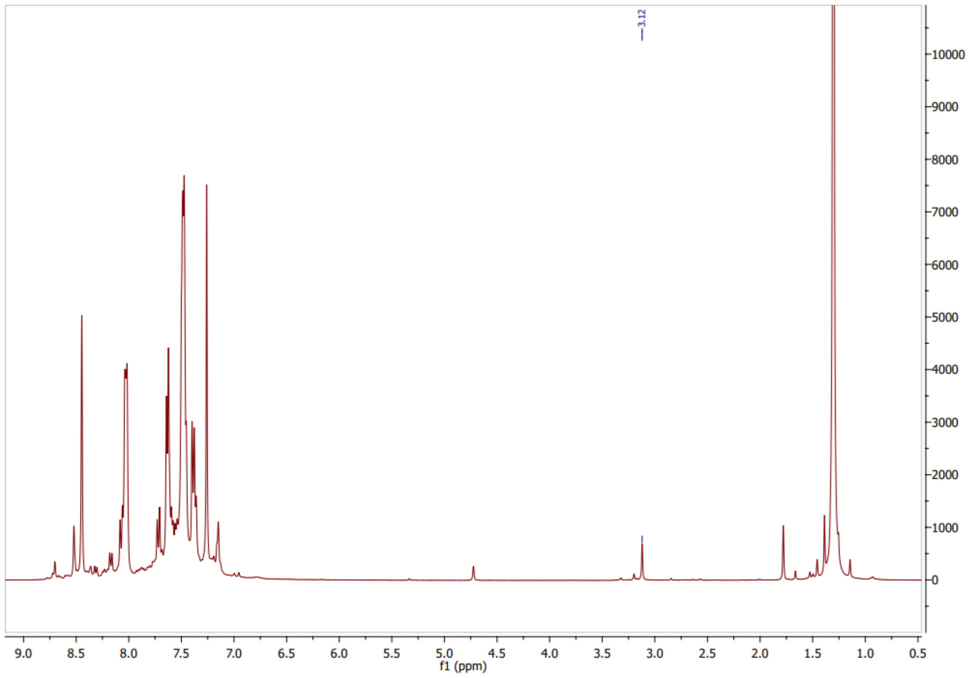

Commercial sample of 9-methylanthracene below – methyl signal at 3.11 ppm (*cf.* 3.12 ppm above)

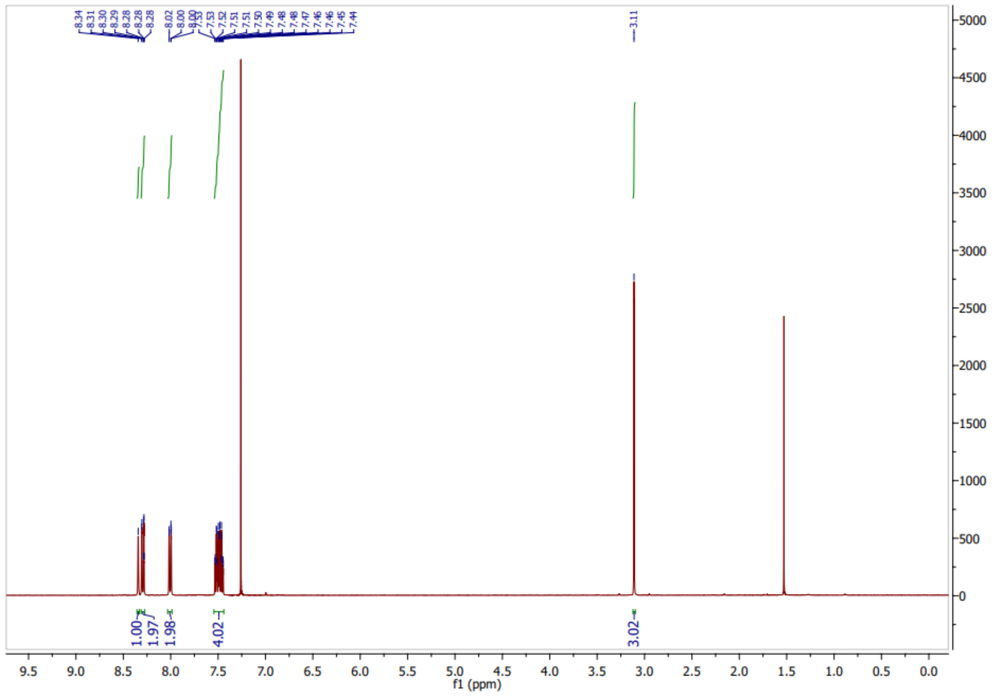

Reactions of Dibromoarene substrates

Side-by-side-by-side reactions of 9,10-dibromoanthracene isotopologues

To an oven-dried microwave vial, primed with a stirrer bar, in a glovebox was added a 9,10-dibromoanthracene **68** isotopologue (0.35 mmol, 1 equiv.) or mixture of two isotopologues (0.175 mmol, 0.5 eq. of each), KO*t*Bu (79 mg, 0.7 mmol, 2 equiv.) and benzene (3.5 mL) with the vial subsequently sealed and stirred at 130 °C in a pre-heated oil bath for 15 mins. Once complete, the crude mixture was quickly cooled to room temperature, D_2O (0.2 mL) and an accurately weighed mass of *n*-dodecane in toluene (10 mL) was added. An aliquot of the crude mixture was then analysed by both GCMS and GC-FID (Method 2).

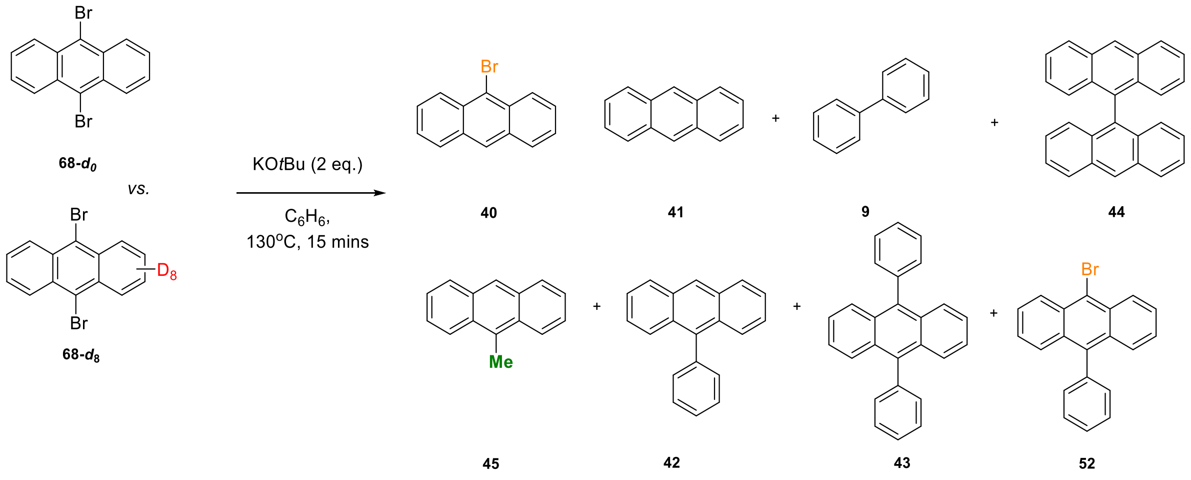

Or isotopologues, depending on substrate used

Substrate	Dodecane (mg)	% Yield[a]								
		**68**	**40**	**9**	**41**	**42**	**43**	**44**	**45**	**52**
**68-*d*₀**	7.5	41.0	22.1	19.1	0.7	2.4	0.4	Trace	0.4	3.7
**68-*d*₈**	7.3	85.1	4.1	2.8	0.0	0.3	0.0	0.0	0.0	4.1

[a] *Yields determined by GC-FID calibrated with authentic samples. Isotopologue yields calculated using calibrations of non-labelled compounds.*

GCFID data for reaction of 68-d_0 including table quantitating components that had been separately calibrated.

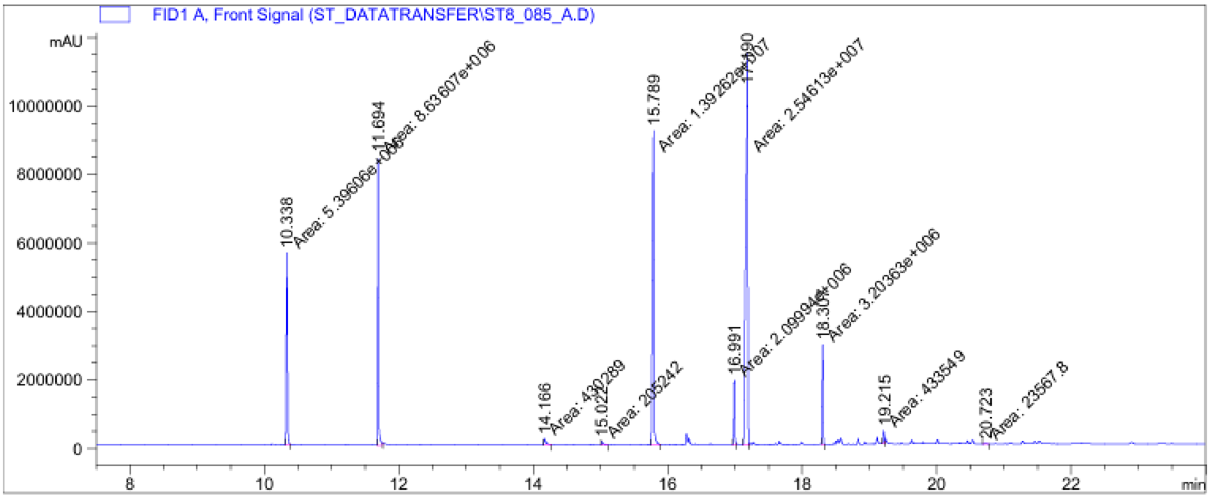

Retention Time (min)	Sample	Peak Area	%Yield
10.338	Dodecane	5396060	N/A
11.694	Biphenyl	8636070	19.1
14.166	Anthracene	430289	0.7
15.022	Methylanthracene	205242	0.4
15.789	Bromoanthracene	13926200	22.1
16.991	Phenylanthracene	2099940	2.4
17.109	Dibromoanthracene	25461300	41.0
18.302	Bromophenylanthracene	3203630	3.7
19.215	Diphenylanthracene	433549	0.4

Dodecane added = 7.5 mg

GCFID data for reaction of 68-d_8

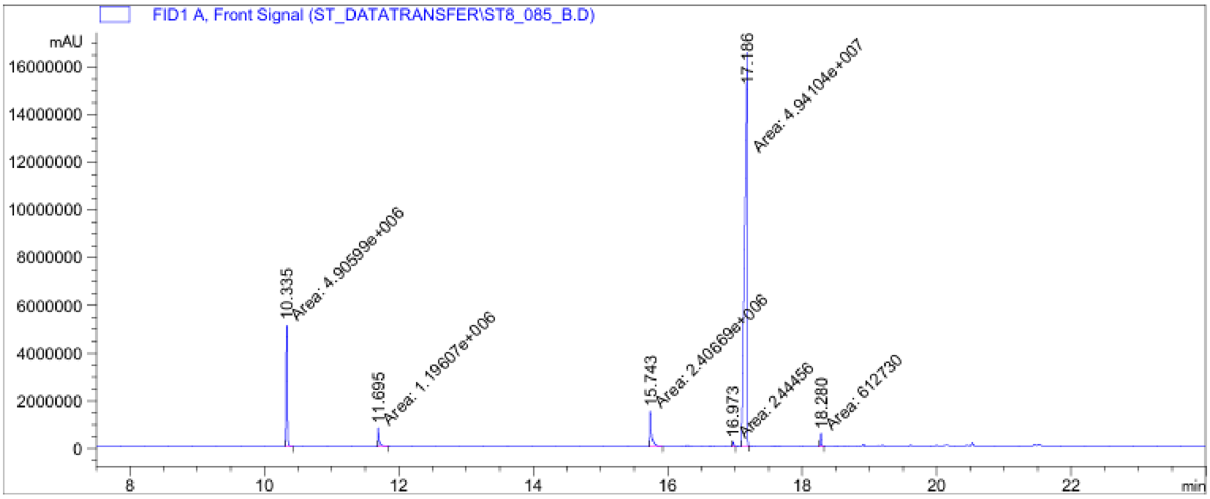

Retention Time (min)	Sample	Peak Area	%Yield
10.335	Dodecane	4905990	N/A
11.695	Biphenyl	1196070	2.8
15.743	Bromoanthracene	2406690	4.1
16.973	Phenylanthracene	244456	0.3
17.186	Dibromoanthracene	49410400	85.1
18.280	Bromophenylanthracene	612730	0.8

Dodecane added = 7.3 mg

Reaction of (((2,3-dibromo-1,4-phenylene)bis(oxy))bis(methylene))dibenzene

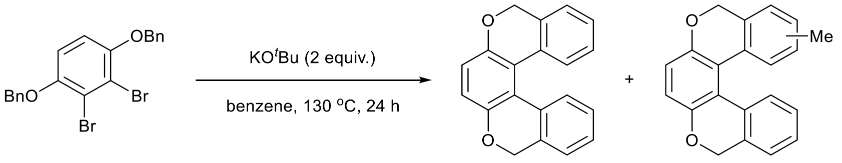

69 70 S6

To an oven-dried pressure tube equipped with a stirrer bar was added (((2,3-dibromo-1,4-phenylene)bis(oxy))bis(methylene))dibenzene **69** (224 mg, 0.5 mmol, 1 equiv.); the tube was flushed with argon and sealed. The tube was then transferred into a glovebox where KO*t*Bu (112 mg, 1 mmol, 2 equiv.) and benzene (5 mL) were added, and the tube sealed. The vial was removed from the glovebox and refluxed in a pre-heated oil bath at 130°C for 24 h. After reflux, the pressure tube was cooled before removing the lid and the reaction mixture quenched with H_2O (30 mL) and extracted with Et_2O (3 x 20 mL). The combined organic phases were dried over Na_2SO_4 and concentrated *in vacuo*. The crude product was purified by chromatography (50% toluene in hexanes → 100% toluene) affording starting material **69** (28.1 mg, 13%) as an off-white solid, and 1,6-dihydrobenzo[*c*]isochromeno[4,3-*f*]chromene **70** (13 mg, 9%) as a white solid. **Mp** 195-196 °C; **^{1}H NMR** (400 MHz, DCM-d_2) δ 7.52 (d, *J* = 7.8 Hz, 2H), 7.29 – 7.24 (m, 4H), 7.16 – 7.09 (m, 2H), 6.92 (s, 2H), 5.03 (m, 4H); **^{13}C NMR** (101 MHz, $CDCl_3$) 151.9, 132.2, 130.4, 127.4, 127.4 125.0, 120.4, 118.2, 69.2; **IR** ν_{max} (neat)/cm^{-1} 1487, 1427, 1195, 1062, 995, 767, 748; **HRMS** (ESI+) [*m/z*] calcd. for $C_{20}H_{14}O_2$ (M^+) 286.0988, found 286.0983. The structure of the doubly cyclised **70** was expected to be the '*cis'* isomer shown based on initial observations in the ^{1}H NMR spectrum. A singlet is expected for the 4 benzylic methylene (Ar-C<u>H</u>$_2$-O) protons with a chemical shift of ~5 ppm. Instead, a broad peak roughly representing two broad singlets was observed. This was evidence of non-equivalence and is proposed to arise from the helical conformation in **70**. Peak broadening in helicenes is precedented.[62] The structure of **70** was then determined by 1D nOe spectroscopy (page S125).

Methylated product **S6** was detected by GCMS as a trace component (RT 18.628 min).

S6 HRMS (EI+) [*m/z*] calcd. for $C_{21}H_{16}O_2$ (M^+) 300.1150, found 300.1143.

Reaction of (((2,3-dibromo-1,4-phenylene)bis(oxy))bis(methylene))dibenzene at 160 °C

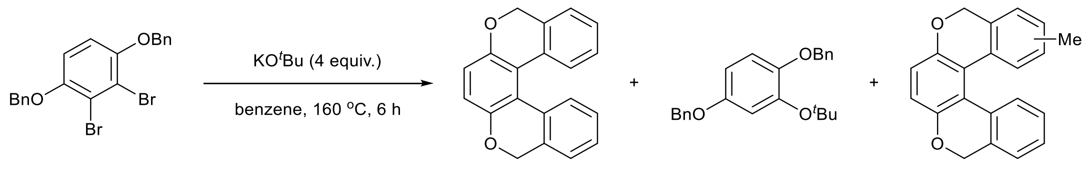

69 **70** **S7** **S6**

(((2,3-Dibromo-1,4-phenylene)bis(oxy))bis(methylene))dibenzene **69** (224 mg, 0.5 mmol, 1 equiv.), KO*t*Bu (224 mg, 2 mmol, 4 equiv.) and benzene (5 mL) were refluxed in an oven-dried pressure tube at 160°C for 6 h. The crude product was purified by chromatography (100% petroleum ether→5% EtOAc in petroleum ether) affording 1,6-dihydrobenzo[*c*]isochromeno[4,3-*f*]chromene **70** (19.2 mg, 13%) as a white solid, and (((2-(*tert*-butoxy)-1,4-phenylene)bis(oxy))bis(methylene))dibenzene **S7** (40 mg, 21%) as an off-white solid. Analytical data for **70** are in agreement with the corresponding data above.

S7 Mp 82-84 °C; **^{1}H NMR** (400 MHz, $CDCl_3$) δ 7.47 – 7.28 (m, 10H), 6.85 (d, *J* = 8.8 Hz, 1H), 6.70 (d, *J* = 3.2 Hz, 1H), 6.62 (dd, *J* = 9.0, 2.9 Hz, 1H), 5.02 (s, 2H), 4.99 (s, 2H), 1.35 (s, 9H) ppm; **^{13}C NMR** (101 MHz, $CDCl_3$) δ 153.4, 147.8, 146.5, 137.8, 137.2, 128.6, 128.4, 127.9, 127.6, 127.5, 127.4, 116.7, 113.5, 109.5, 80.2, 72.2, 70.6, 28.8 ppm; **IR** v_{max} (neat)/cm^{-1} 2978, 1500, 1263, 1153, 1026, 732; **HRMS** (ESI+) [*m/z*] calcd. for $C_{24}H_{26}O_3Na$ $(M+Na)^+$ 385.1774, found 385.1773.

Reaction of (((2,3-dibromo-1,4-phenylene)bis(oxy))bis(methylene))dibenzene

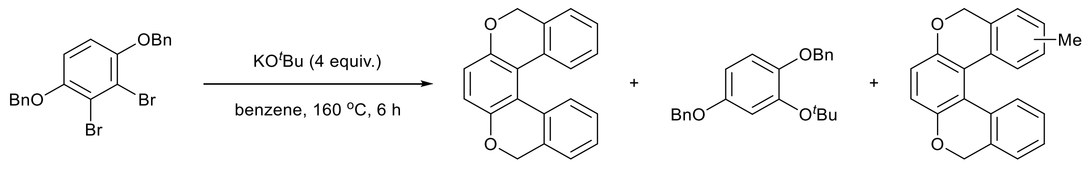

69 **70** **S7** **S6**

This was a duplicate experiment of the above. (((2,3-Dibromo-1,4-phenylene)bis(oxy))-bis(methylene))dibenzene **69** (112 mg, 0.25 mmol, 1 equiv.), KO*t*Bu (112 mg, 1 mmol, 4 equiv.) and benzene (5 mL) were refluxed in an oven-dried pressure tube at 160°C for 6 h. The crude product was purified by chromatography (100% petroleum ether→5% EtOAc in petroleum ether) affording 1,6-dihydrobenzo[*c*]isochromeno[4,3-*f*]chromene **70** (13 mg, 20%) as a white solid, and (((2-(*tert*-butoxy)-1,4-phenylene)bis(oxy))bis(methylene))dibenzene **S7** (22 mg, 26%) as an off-white solid. Analytical data for **70** and **S7** are consistent with the corresponding data above.

Reaction of (((2,3-dibromo-1,4-phenylene)bis(oxy))bis(methylene))dibenzene

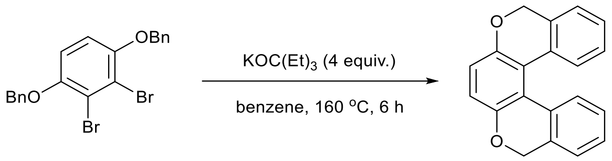

69 70

The same procedure was followed as for when KO*t*Bu was used. (((2,3-Dibromo-1,4-phenylene)bis(oxy))bis(methylene))-dibenzene **69** (105 mg, 0.23 mmol, 1 equiv.), $KOC(Et)_3$ (145 mg, 0.94 mmol, 4 equiv.) and benzene (2.3 mL) were refluxed in an oven-dried pressure tube at 160 °C for 6 h. The crude product was purified by chromatography (50% toluene in hexanes→100% toluene) affording 1,6-dihydrobenzo[*c*]isochromeno[4,3-*f*]chromene **70** (21.2 mg, 32%) as a white solid. Analytical data for **70** are in agreement with the corresponding data above.

Control Thermal Reaction of (((2,3-dibromo-1,4-phenylene)bis(oxy))bis(methylene))dibenzene to test for spontaneous thermal reaction.

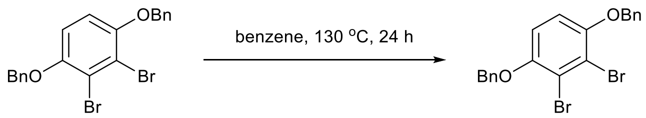

69 69

(((2,3-Dibromo-1,4-phenylene)bis(oxy))bis(methylene))dibenzene **69** (224 mg, 0.5 mmol, 1 equiv.) and benzene (5 mL) were refluxed in an oven-dried pressure tube at 160°C for 6 h. After reflux, the pressure tube was cooled before removing the lid and the reaction mixture quenched with H_2O (30 mL) and extracted with Et_2O (3 x 30 mL). The combined organic phases were dried over Na_2SO_4 and concentrated *in vacuo*. The crude product was analysed by GC-MS, affording unchanged starting material **69**. Analytical data for **69** are in agreement with the corresponding data above.

Reaction of 1,4-bis(benzyloxy)-2,3-dibromonaphthalene 71

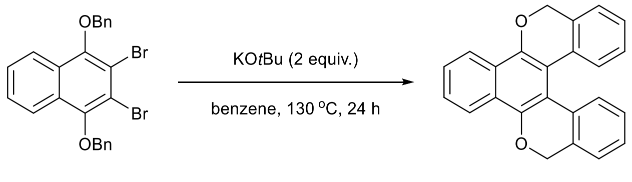

71 72

1,4-Bis(benzyloxy)-2,3-dibromonaphthalene **71** (140 mg, 0.23 mmol, 1 equiv.), KO*t*Bu (63 mg, 0.56 mmol, 2 equiv.) and benzene (2.38mL) were refluxed in an oven-dried pressure tube at 130°C for 24 h. After reflux, the pressure tube was cooled before removing the lid and the reaction mixture quenched with H_2O (15 mL) and extracted with Et_2O (3 x 30 mL). The combined organic phases were dried over Na_2SO_4 and concentrated *in vacuo*. The crude product was purified by chromatography (100% hexanes→2% EtOAc in hexanes) affording 6,10b,10c,15-tetrahydrodibenzo[*c*,*h*]isochromeno-[4,3-*f*]chromene **72** (10.7 mg, 11%) as a yellow crystalline solid. **Mp** 234-236 °C; **^{1}H NMR** (400 MHz, $(CD_3)_2CO$) δ 8.25 – 8.19 (m, 2H), 7.60 – 7.55 (m, 2H), 7.47 – 7.42 (dd, *J* = 7.5, 0.8 Hz, 2H), 7.41 – 7.37 (d, *J* = 8.0 Hz, 2H), 7.33 – 7.28 (td, *J* = 7.4, 1.2 Hz, 2H), 7.21 – 7.16 (td, *J* = 7.8, 1.2 Hz, 2H), 5.54 – 5.42 (d, *J* = 12.5 Hz, 2H), 5.22 – 5.11 (d, *J* = 12.3 Hz, 2H) ppm. **^{13}C NMR** (101 MHz, $(CD_3)_2CO$) δ 148.7, 132.0, 131.5, 128.3, 128.1, 127.9, 127.7, 126.7, 125.9, 123.0, 115.2, 70.0 ppm; **IR** ν_{max} (neat/cm^{-1}) 1479, 1392, 1333, 1076, 754; **HRMS** (APCI+) [*m/z*] calcd. for $C_{24}H_{17}O_2$ $(M+H)^+$ 337.1223, found 337.1212.

Substrate synthesis

Synthesis of iodobenzene-*d*$_5$

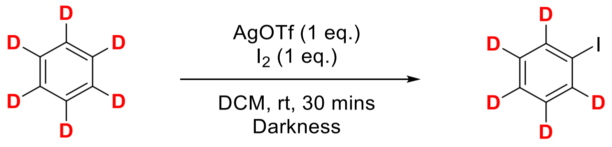

4-*d*$_6$ **1-*d*$_5$**

This reaction was carried out according to a literature procedure.[63] An oven-dried 25 mL Schlenk flask under argon was charged with 99.5%D C_6D_6 **4-*d*$_6$** (443 µL, 5 mmol, 1 equiv.), AgOTf (1.28 g, 5 mmol, 1 equiv.) and dry DCM (15 mL). The mixture was stirred in the dark for 10 mins, then I_2 (1.27 g, 5 mmol, 1 equiv.) was added. The reaction was stirred in the dark for 30 mins at room temperature. After this time, the reaction mixture was passed through a celite plug and washed with further DCM. The organics were then washed with sat. Na_2SO_3, then brine, then dried over $MgSO_4$ and concentrated under reduced pressure. The crude was then purified by column chromatography (100% pentane) yielding **1-*d*$_5$** as a colourless liquid (824 mg, 3.94 mmol, 79 %). **^{2}H NMR** (61 MHz, $CHCl_3$) δ 7.29, 6.91, 6.70. **^{13}C NMR** (101 MHz, $CHCl_3$) δ 137.9 – 136.3 (m), 130.5 – 128.9 (m), 127.6 – 126.3 (m), 94.1. **GC-MS (EI)**: 209.0 ([M$^+$]). The data are in agreement with literature data.[64]

Synthesis of KO*t*Bu-*d₉*

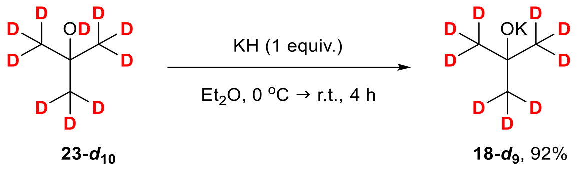

In an oven-dried three necked flask, primed with a stirrer bar, and back filled with argon was added KH (400 mg, 10 mmol, 1 equiv.) and Et_2O (10 mL) and the solution stirred at 0 °C. To this solution was added *tert*-butanol-d_{10} **23-d_{10}** (0.94 mL, 10 mmol, 1 equiv.) and the solution was stirred for 4 h. Once complete, the Et_2O was removed using the house vacuum until dryness. This yielded potassium 2-(methyl-d_3)propan-2-olate-1,1,1,3,3,3-d_6 **18-d_9** (KO*t*Bu-d_9) as a white powder (1.119 mg, 9.2 mmol, 92%). **^{13}C NMR** (101 MHz, THF-D_8) δ 66.2, δ 34.6 (sept, *J* = 18.7 Hz) [Confirmed by comparing with ^{13}C NMR spectrum of HO*t*Bu-d_{10}: **^{13}C NMR** (101 MHz, THF-D_8) δ 57.4, 30.5 (sept, *J* = 18.9 Hz)].

Synthesis of $KOCEt_3$ [65]

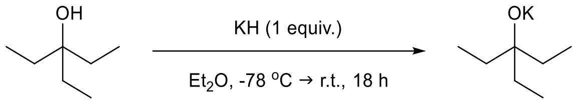

To an oven-dried three-necked flask, equipped with a stirrer bar and vacuum tap, under an atmosphere of argon, was added washed potassium hydride (400 mg, 10 mmol, 1 equiv.) and dry diethyl ether (20 mL) and the solution was stirred at -78 °C for 30 min. To this mixture was slowly added triethylcarbinol (1.37 mL, 10 mmol, 1 equiv.) and the mixture stirred at -78 °C for 2 h, then allowed to warm to room temperature overnight. The majority of the solvent was removed on the house vacuum line, with the rest of the solvent removed on the high vacuum line and the crude material allowed to dry for a further 5 h. The flask was then put under an atmosphere of argon and transferred to a glovebox. This yielded potassium 3-ethylpentan-3-olate as a pale-yellow solid (1.442 g, 9.3 mmol, 93%). **^{1}H NMR** (400 MHz, C_6D_6) δ 1.22 (q, *J* = 7.5 Hz, 6H), 0.83 (t, *J* = 7.5 Hz, 9H) ppm. **^{13}C NMR** (101 MHz, C_6D_6) δ 72.7, 33.8, 8.9 ppm. Data for the product were consistent with the literature.[65]

Synthesis of 9-bromoanthracene-*d₉* ST8- 91

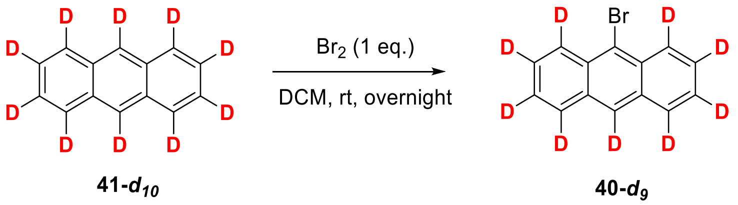

To a 50 mL round-bottom flask was added anthracene-d_{10} **41-d_{10}** (377 mg, 2 mmol, 1 eq.), dry DCM (50 mL) and a stirbar. The mixture was stirred at room temperature until the substrate dissolved, then bromine (102 μL, 2 mmol, 1 eq.) was added dropwise, giving an orange solution with was left to stir

at room temperature overnight. Analysis of the crude showed a mixture of mono-brominated and di-brominated anthracene along with unreacted starting material. The reaction mixture was diluted with DCM (75 mL) and washed with water (50 mL), then the organics were dried over $MgSO_4$ and concentrated under reduced pressure. The crude was purified by column chromatography (100% hexane) yielding a yellow solid which was subsequently recrystallized from DCM/hexane in a freezer, yielding bright yellow needle-like crystals of 9-bromoanthracene-d_9 **40-d_9** (317 mg, 1.19 mmol, 60 %). **^{2}H NMR** (61 MHz, $CHCl_3$) δ 8.58, 8.52, 8.07, 7.66, 7.57. **^{13}C NMR** (101 MHz, $CDCl_3$) δ 132.2, 130.7, 128.6 – 127.9 (m), 127.7 – 127.0 (m), 127.3 – 126.8 (m), 127.2 – 126.5 (m), 125.6 – 124.9 (m), 122.4. **GC-MS (EI)**: 265.0, 267.0 (1:1 [M^+]).

Synthesis of 9,10-dibromoanthracene 68-d_8

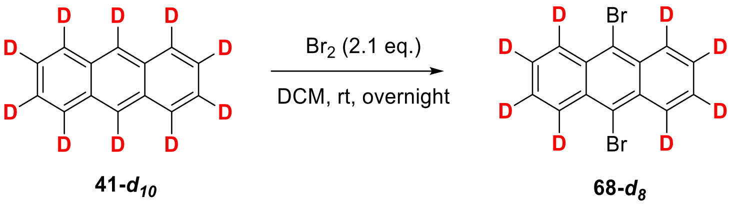

A 50 mL round-bottom flask was charged with anthracene-D_{10} **41-d_{10}** (504 mg, 2.68 mmol, 1 equiv.), dry DCM (25 mL) and a stirbar, then stirred at room temperature until all the substrate dissolved. Bromine (228 µL, 5.62 mmol, 2.1 equiv.) was then added dropwise, initially the solution turned yellow and by the end of the addition was a dark orange-red with precipitates. The solution was allowed to stir at room temperature overnight, then dissolved in DCM (150 mL). The organics were washed with water (100 mL) and then brine (100 mL), then dried over $MgSO_4$. The organics were then concentrated to approximately 50% volume and left to stand, producing a crop of bright yellow needle-like crystals of 9,10-dibromoanthracene-d_8. Further concentration of the mother liquor produced a second crop of crystals (total = 774 mg, 2.13 mmol, 80 %). **^{2}H NMR** (61 MHz, $CHCl_3$) δ 8.64, 7.69. **^{13}C NMR** (101 MHz, $CHCl_3$) δ 131.0, 128.2 – 127.4 (m), 127.3 – 126.5 (m), 123.4. **GC-MS (EI)**: 344.0, 342.0, 346.0 (2:1:1 [M^+]).

Synthesis of 9-bromoanthracene-d_1

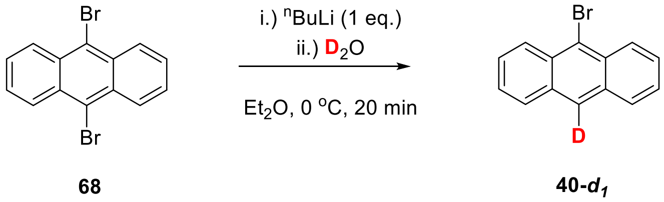

A oven-dried 25 mL flask was charged with 9,10-dibromoanthracene (672 mg, 2 mmol, 1 equiv.) and a stirbar, then evacuated and backfilled with argon three times. Dry Et_2O (15 mL) was added and then the mixture stirred at 0°C in an ice bath. A 2.1 M solution of ^{n}BuLi (896 µL, 2 mmol, 1 eq.) was then added dropwise, yielding an orange solution which was allowed to stir for 30 mins before D_2O (0.6 mL) was added. After warming to room temperature, the reaction mixture was diluted with DCM (50 mL)

and washed with water (30 mL), then the organics dried over $MgSO_4$ and concentrated under reduced pressure. The crude was then purified by column chromatography (100% hexane). The combined fractions were the concentrated and subsequently recrystallized by dissolving in warm hexane then leaving in the freezer overnight, yielding bright yellow needle-like crystals of 9-bromoanthracene-d_1 **40-D₁** (331 mg, 1.29 mmol, 64 %). **^{1}H NMR** (400 MHz, $CDCl_3$) δ 8.53 (d, J = 8.9 Hz, 2H), 8.01 (d, J = 8.5 Hz, 2H), 7.63 – 7.58 (m, 2H), 7.53 – 7.48 (m, 2H). **^{2}H NMR** (61 MHz, $CHCl_3$) δ 8.49. **^{13}C NMR** (101 MHz, $CDCl_3$) δ 132.2, 130.7, 128.7, 127.8, 127.3, 127.2 – 126.6 (m), 125.8, 122.5. **GC-MS (EI)**: 257.1, 259.0 (1:1 [M^+]).

Synthesis of 9-bromoanthracene-d_8

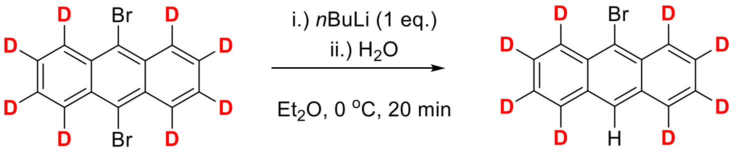

68-*d*₈ **40-*d*₈**

A oven-dried 25 mL flask was charged with 9,10-dibromoanthracene-d_8 **68-*d*₈**(516 mg, 1.5 mmol, 1 equiv.) and a stirbar, then evacuated and backfilled with argon three times. Dry Et_2O (11.5 mL) was added and then the mixture stirred at 0°C in an ice bath. A 2.1 M solution of ^{n}BuLi (710 µL, 1.5 mmol, 1 eq.) was then added dropwise, yielding an orange solution which was allowed to stir for 20 mins before H_2O (0.6 mL) was added. After warming to room temperature, the reaction mixture was diluted with DCM (50 mL) and washed with water (30 mL), then the organics dried over $MgSO_4$ and concentrated under reduced pressure. The crude was then purified by column chromatography (100% hexane). The combined fractions were the concentrated and subsequently recrystallized by dissolving in warm hexane then leaving in the freezer overnight, yielding bright yellow needle-like crystals of 9-bromoanthracene-d_8 (294 mg, 1.11 mol, 74 %). **^{1}H NMR** (400 MHz, $CDCl_3$) δ 8.46 (s, 1H). **^{2}H NMR** (61 MHz, $CHCl_3$) δ 8.58, 8.07, 7.67, 7.57. **^{13}C NMR** (101 MHz, $CHCl_3$) δ 132.0, 130.5, 128.4 – 127.8 (m), 127.4 – 126.9, 126.9, 127.0 – 126.3 (m), 125.4 – 124.8 (m), 122.2. **GC-MS (EI)**: 264.0, 266.0 (1:1 [M^+]).

(((2,3-dibromo-1,4-phenylene)bis(oxy))bis(methylene))dibenzene

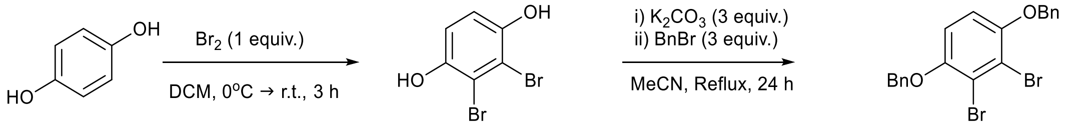

S13 **S14** **69**

+ 2,5-dibromo isomer **S15** and monobromohydroquinone **S16**

+ dibenzyl ether of 2,5-dibromo isomer **S17** + dibenzyl ether of monobromohydroquinone **S18**

The reaction was carried out according to a literature procedure on a 91 mmol scale.[66] To a stirred solution of hydroquinone **S13** (10 g, 91 mmol, 1 equiv.) in DCM (80 mL) at 0 °C was added bromine (4.6 mL, 91 mmol, 1 equiv.) dropwise within 15 min and the mixture stirred for 3 h at r.t. The reaction was quenched with 10% $Na_2S_2O_3$ (50 mL) and the aqueous layer extracted with EtOAc (3 x 50 mL).

Combined organic layers were washed with brine (50 mL), dried over Na_2SO_4, and concentrated *in vacuo*. The crude product was purified by chromatography (100% hexanes→5% EtOAc in hexanes) affording 2,3-dibromohydroquinone **S14** alongside the expected inseparable products (monobromohydroquinone **S15** and 2,5-dibromohydroquinone **S16**) (total, 5 g). Chromatography to remove unreacted hydroquinone **S13** was essential in order to achieve purified product in the second synthetic step. Monobromohydroquinone **S15** was isolated by careful chromatography for characterisation. [Later repeats of the syntheses isolated the mixture of brominated isomers for subsequent synthesis].

Bromohydroquinone **S15** (720 mg, 4%) was isolated as a beige solid. **Mp** 102-104 °C; **^{1}H NMR** (400 MHz, $CDCl_3$) δ 6.98 (d, *J* = 3.0 Hz, 1H), 6.89 (d, *J* = 8.7 Hz, 1H), 6.72 (dd, *J* = 8.2, 2.4 Hz, 1H) ppm. **^{13}C NMR** (101 MHz, $CDCl_3$) δ 149.7, 146.7, 118.7, 116.5, 116.5, 110.0 ppm. **GC-MS** [*m/z* (%)] (9.67 min) 188 (M^+, 21), 190 (21), 160 (7), 162 (7), 132 (9), 106 (46), 104 (46), 79 (94), 53 (100). **ATR IR ν_{max}** (neat)/cm^{-1}: 3500-3000 (broad OH), 1597, 1517, 1446, 1361, 1228, 1197. The NMR data are consistent with the literature.[67]

The benzylation reaction was carried out on a 26.5 mmol scale. Under a positive pressure of argon, benzyl bromide (9.5 mL, 79.5 mmol, 3 equiv.) was added to a stirred solution of 2,3-dibromohydroquinone **S14** and the other inseparable brominated isomers and monobromohydroquinone **S15** [5 g, 26.5 mmol (based on lowest MW product bromohydroquinone, 1 equiv.)] and K_2CO_3 (11 g, 79.5 mmol, 3 equiv.) in acetonitrile (80 mL) and the mixture refluxed for 24 h. The crude mixture was filtered and concentrated *in vacuo*. The crude product was purified first by hot filtration in ethanol. Only the 2,5-dibrominated isomer, (((2,5-dibromo-1,4-phenylene)bis(oxy))-bis(methylene))dibenzene **S16** is insoluble in hot ethanol. The crude product was further purified by chromatography (100% hexanes→5% EtOAc in hexanes) to yield (((2,3-dibromo-1,4-phenylene)bis(oxy))bis(methylene))dibenzene **69** (1.16 g, 3%, over two steps) as a crystalline white solid and (((2-bromo-1,4-phenylene)bis(oxy))bis(methylene))dibenzene **S17** (0.99 g, 3%) as a beige solid.

69 Mp 128-131 °C; **^{1}H NMR** (400 MHz, $CDCl_3$) δ 7.48–7.43 (m, 4H), 7.41–7.36 (m, 4H), 7.35–7.29 (m, 2H), 6.84 (s, 2H), 5.09 (s, 4H) ppm; **^{13}C NMR** (101 MHz, $CDCl_3$) δ 151.1, 136.6, 128.7, 128.2, 127.3, 117.8, 113.3, 72.1 ppm**; IR** ν_{max} (neat)/cm^{-1} 3039, 1492, 1452, 1274, 1224, 1209, 1043, 1012, 842, 732, 694. **HRMS** (APCI+) [*m/z*]: calcd. for $C_{20}H_{16}{}^{79}Br_2O_2$ (M^+): 445.9512, found: 445.9512. The molecular structure was also confirmed by X-Ray crystallography. **(see page S135)**.

S17 Mp 52-54 °C; **^{1}H NMR** (400 MHz, $CDCl_3$) δ 7.49–7.28 (m, 10H), 7.23 (d, *J* = 2.7 Hz, 1H), 6.89–6.81 (m, 2H), 5.09 (s, 2H), 5.00 (s, 2H) ppm; **^{13}C NMR** (101 MHz, $CDCl_3$) δ 153.6, 149.7, 136.9, 136.8, 128.7, 128.6, 128.2, 128.0, 127.6, 127.3, 120.2, 115.4, 114.7, 113.2, 71.9, 70.9 ppm; **GC-MS** [*m/z* (%)] (18.09 min) 370 (6), 368 (M+, 6), 91 (100), 65 (18); **ATR IR** ν_{max} (neat)/cm^{-1}, 3062, 3035, 2858, 1604, 1492, 1452, 1379, 1273, 1222, 1207, 1041, 1010, 732. ^{1}H NMR data are in agreement with the literature.[68]

2,3-Dibromonaphthalene-1,4-diol

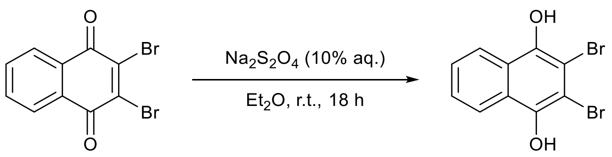

S18 **S19**

This product was synthesised according to an adapted literature procedure.[69] An aqueous solution of $Na_2S_2O_4$ (10% w/v, 100 mL) was added to a stirring solution of 2,3-dibromonaphthalene-1,4-dione **S18** (2000 mg, 6.3 mmol, 1 equiv.) dissolved in Et_2O (100 mL) and the biphasic mixture stirred vigorously at RT overnight. After reaction, the mixture was extracted with EtOAc (3 x 50 mL), dried over Na_2SO_4, and concentrated *in vacuo* to afford the title compound **S19** as an off-white solid. The product was quickly used in the following benzylation because of its sensitivity to oxidation. **^{1}H NMR** (400 MHz, $CDCl_3$) δ 8.21 – 8.15 (m, 2H), 7.59 – 7.53 (m, 2H), 5.70 (s, 2H). **^{13}C NMR** (101 MHz, $CDCl_3$) δ 143.5, 127.3, 123.9, 122.6, 104.8.

1,4-Bis(benzyloxy)-2,3-dibromonaphthalene 71

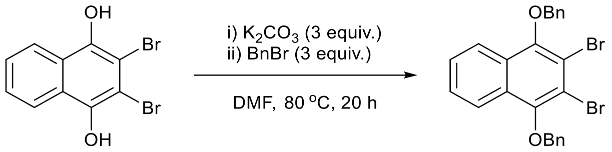

S19 **71**

Under a positive pressure of argon, 2,3-dibromonaphthalene-1,4-diol **S19** (1.1 g, 3.5 mmol, 1 equiv.) and K_2CO_3 (1.45 g, 10.5 mmol, 3 equiv.) were stirred in DMF (30 mL) at rt for 10 min. To the flask was added benzyl bromide (1.2 mL, 10.5 mmol, 3 equiv.) and the contents heated to 80 °C for 20 h. After reaction, the crude mixture was partitioned between EtOAc (50 mL) and H_2O (50 mL). The separated organics were washed with H_2O (2 x 50 mL). The combined aqueous phase was washed with EtOAc (50 mL). The combined organics were washed with brine (30 mL), dried over Na_2SO_4, and concentrated *in vacuo*. The crude mixture was purified by chromatography (100% hexanes → 5% EtOAc in hexanes) affording 1,4-bis(benzyloxy)-2,3-dibromonaphthalene **71** as an impure solid. Further purification by recrystallisation from hot ethanol and hexane afforded **71** (140.4 mg, 8%) as a white solid. **Mp** 125-126 °C; **^{1}H NMR** (400 MHz, $CDCl_3$) δ 8.14 – 8.08 (m, 2H), 7.66 – 7.61 (m, 4H), 7.57 – 7.52 (m, 2H), 7.50 – 7.37 (m, 6H), 5.13 (s, 4H) ppm. **^{13}C NMR** (101 MHz, $CDCl_3$) δ 150.2, 136.7, 128.8, 128.7, 128.6, 128.4, 127.6, 123.0, 116.9, 75.9 ppm; **IR** ν_{max} (neat/cm^{-1}), 1555, 1435, 1342, 1074, 961, 912, 738, 692. **HRMS** (ESI+) [*m/z*] calcd. for $C_{24}H_{22}O_2N_1Br_2$ $(M+NH_4)^+$ 514.0012, found 513.9998.

Synthesis of 1,3-diphenylbenzene

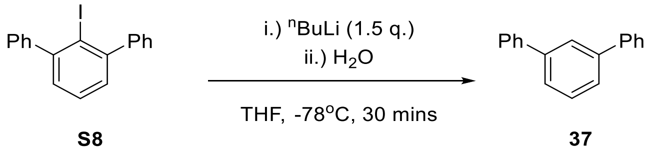

S8 **37**

An oven-dried 3-neck round bottom flask was charged with 2,6-diphenyliodobenzene **S8** (178 mg, 0.5 mmol 1 eq.) and evacuated/backfilled with argon 3 times. The solid was then dissolved in dry THF (10 mL) and cooled to -78°C in a dry ice/IPA bath. With stirring, a 2.5 M solution of ^{n}BuLi (0.3 mL, 0.75 mmol, 1.5 eq.) was added dropwise, giving a bright yellow solution. After stirring for 30 mins, water (0.5 mL) was added, giving a colourless mixture with white insolubles. The reaction was warmed to room temperature, washed with water (10 mL) and extracted into EtOAc (2 x 10 mL). Recrystallization of the crude from hot ethanol yielded 1,3-diphenylbenzene **37** as white needle-like crystals (94.9 mg, 0.41 mmol, 82 %). **^{1}H NMR** (400 MHz, $CDCl_3$) δ 7.84 (t, *J* = 1.6 Hz, 1H), 7.70 – 7.66 (m, 4H), 7.63 – 7.59 (m, 2H), 7.57 – 7.52 (m, 1H), 7.52 – 7.46 (m, 4H), 7.43 – 7.37 (m, 2H). **^{13}C NMR** (101 MHz, $CDCl_3$) δ 142.0, 141.4, 129.3, 129.0, 127.6, 127.4, 126.3, 126.3. **GC-MS (EI)**: 230.1 ([M^+]). The data are in agreement with the literature.[70]

Synthesis of 1,2-diphenylbenzene

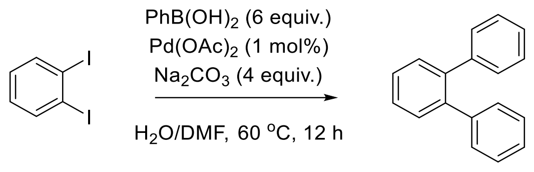

S23 **31**

Carried out according to a literature procedure.[14] A 25 mL microwave vial was charged with 1,2-diiodobenzene **S23** (131 µL, 1 mmol, 1 equiv.), $PhB(OH)_2$ (731 mg, 6 mmol, 6 equiv.), Na_2CO_3 (424 mg, 4 mmol, 4 equiv.), $Pd(OAc)_2$ (2.2 mg, 0.01 mmol, 0.01 equiv.), H_2O (3.5 mL) and DMF (3 mL) under air. A stirbar was added, then the vial was capped and stirred at 60 °C for 12 h. The reaction was allowed to cool to room temperature, and diluted with EtOAc (50 mL), then washed with H_2O (2 x 30 mL) and sat. LiCl (30 mL). The organics were then dried over $MgSO_4$ and concentrated under reduced pressure. The crude was purified by column chromatography (100% hexane) yielding 1,2-diphenylbenzene **31** as a white crystalline solid (184 mg, 0.80 mmol, 80 %). **^{1}H NMR** (400 MHz, $CDCl_3$) δ 7.46 – 7.41 (m, 4H), 7.25 – 7.13 (m, 10H). **^{13}C NMR** (101 MHz, $CDCl_3$) δ 141.6, 140.6, 130.6, 129.9, 127.9, 127.5, 126.5. **GC-MS (EI)**: 230.1 ([M^+]). The data are in agreement with the literature.[70]

NMR spectra

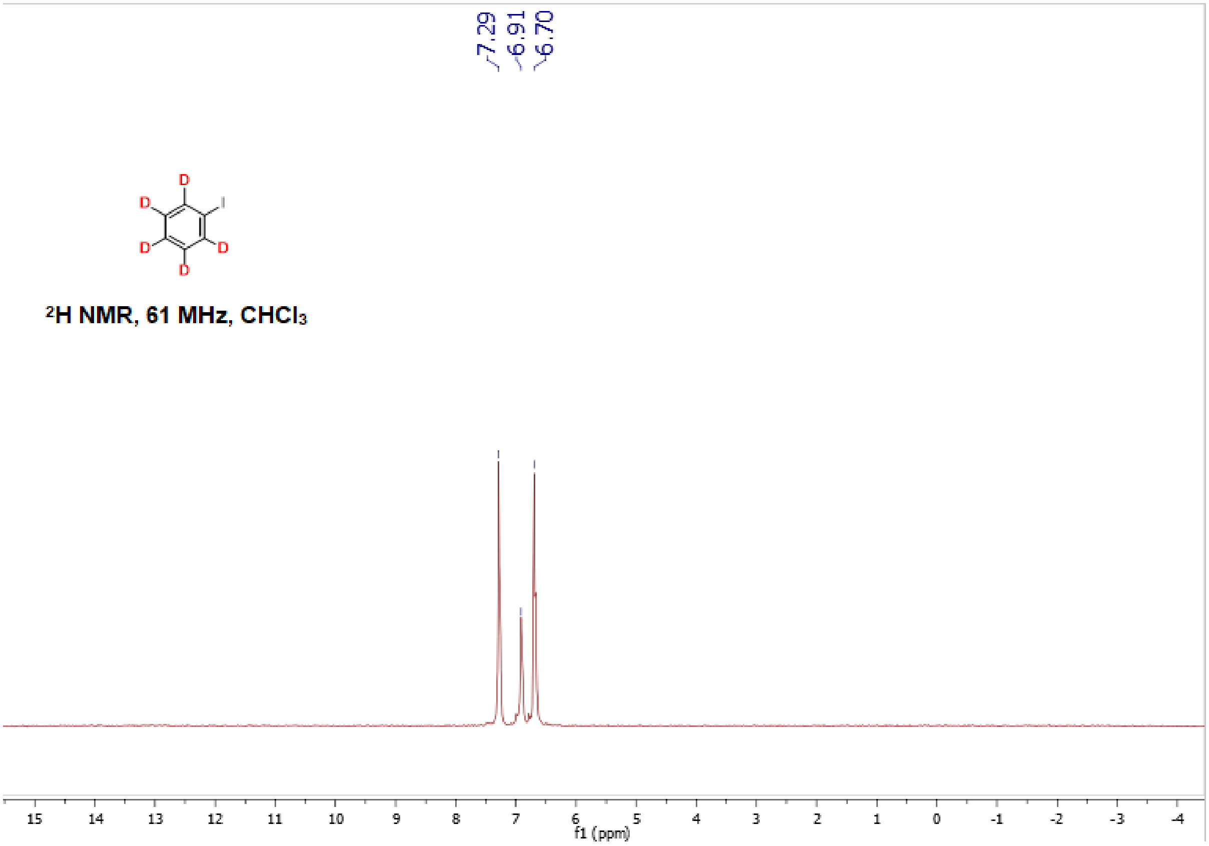

7.29
6.91
6.70
D
D
I
D
D
D
²H NMR, 61 MHz, CHCl3
15 14 13 12 11 10 9 8 7 6 5 4 3 2 1 0 -1 -2 -3 -4
f1 (ppm)

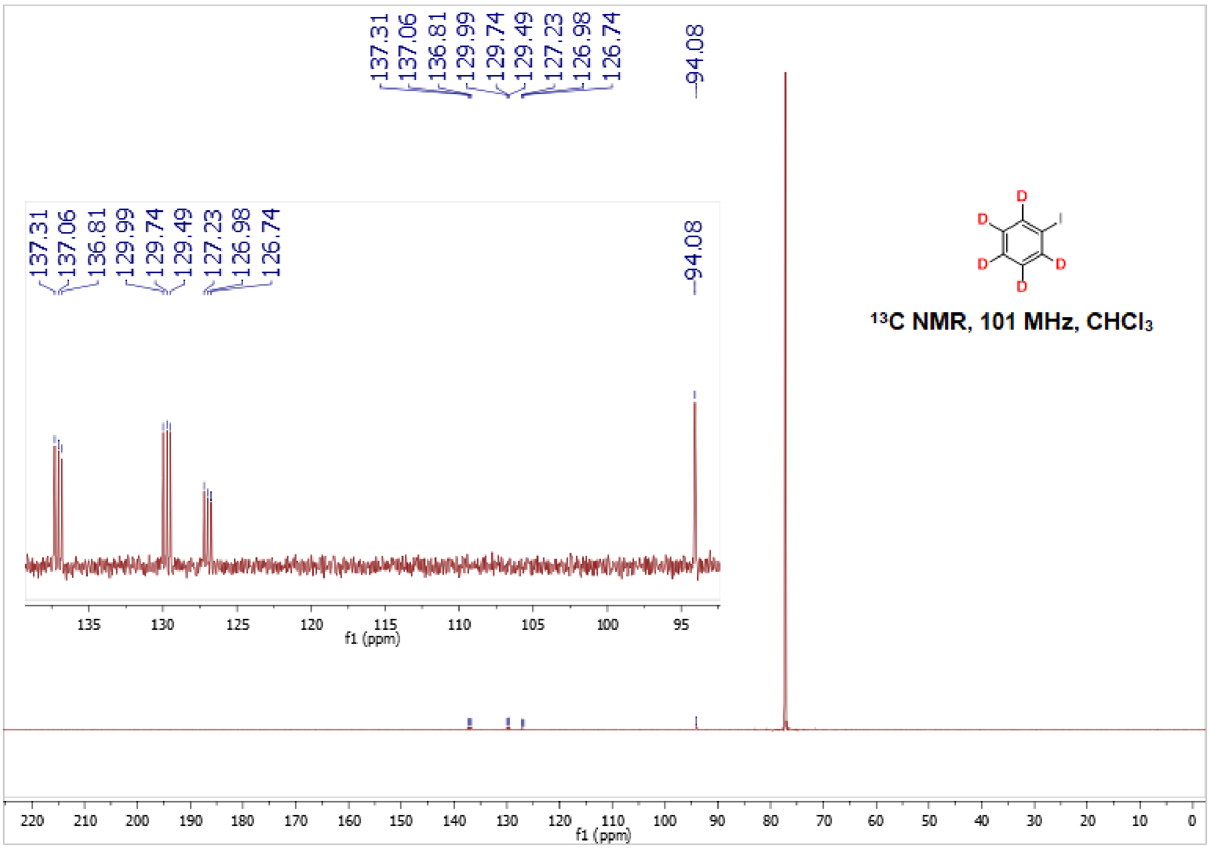

137.31
137.06
136.81
129.99
129.74
129.49
127.23
126.98
126.74
94.08
135 130 125 120 115 110 105 100 95
f1 (ppm)
D
D
I
D
D
D
13C NMR, 101 MHz, CHCl3
220 210 200 190 180 170 160 150 140 130 120 110 100 90 80 70 60 50 40 30 20 10 0
f1 (ppm)

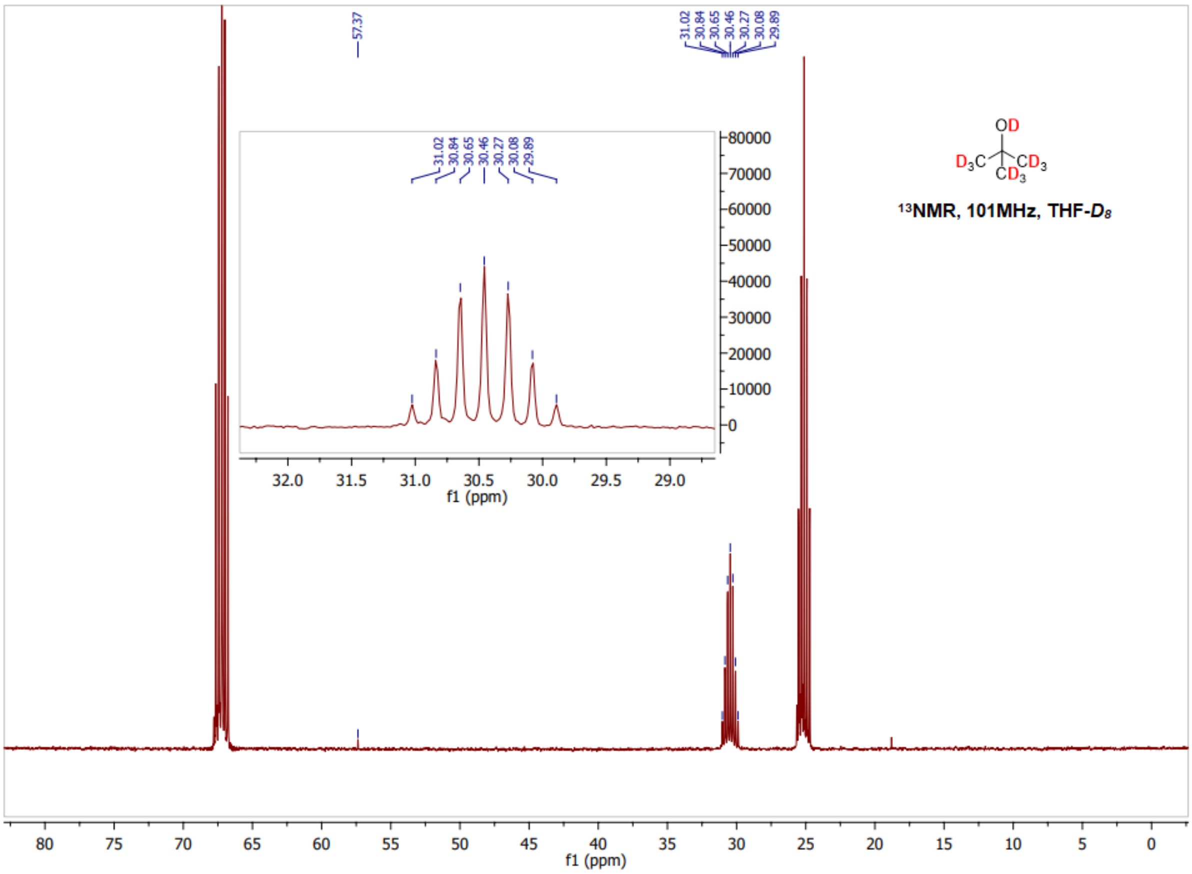
OD
D_3C CD_3 CD_3
13NMR, 101MHz, THF-D_8
57.37
31.02 30.84 30.65 30.46 30.27 30.08 29.89
f1 (ppm)

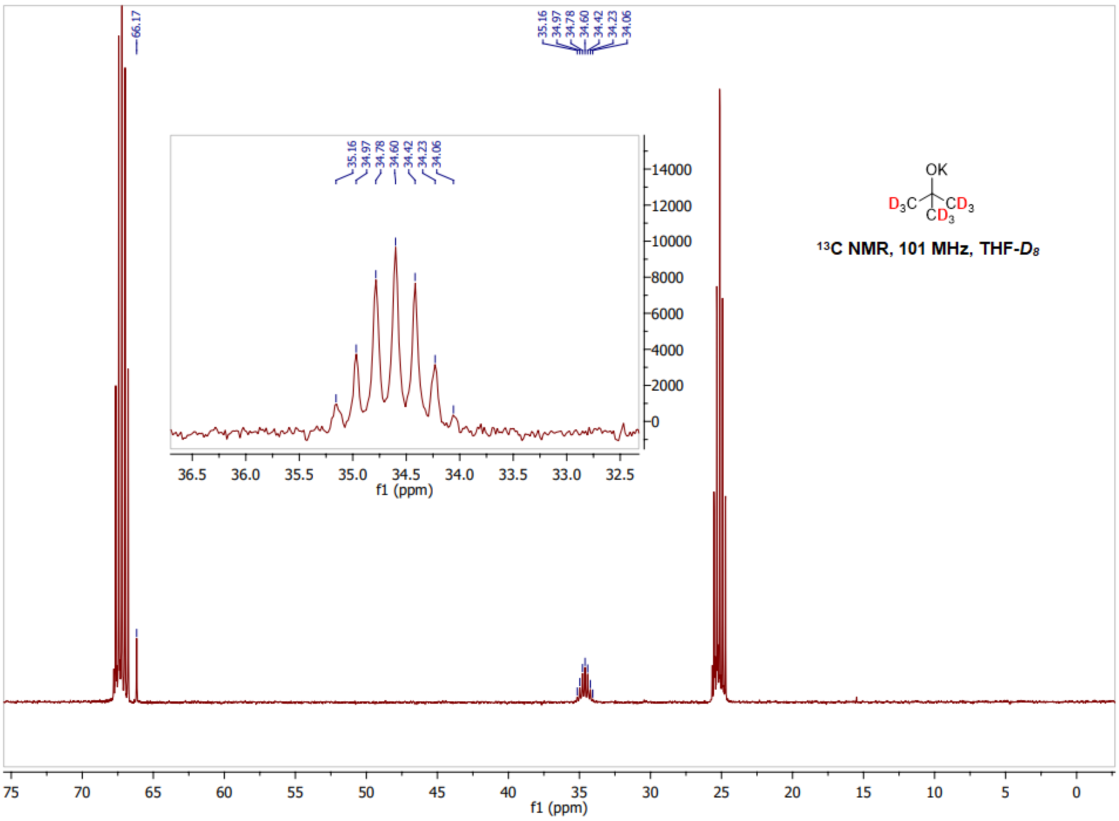
OK
D_3C CD_3 CD_3
13C NMR, 101 MHz, THF-D_8
66.17
35.16 34.97 34.78 34.60 34.42 34.23 34.06
f1 (ppm)

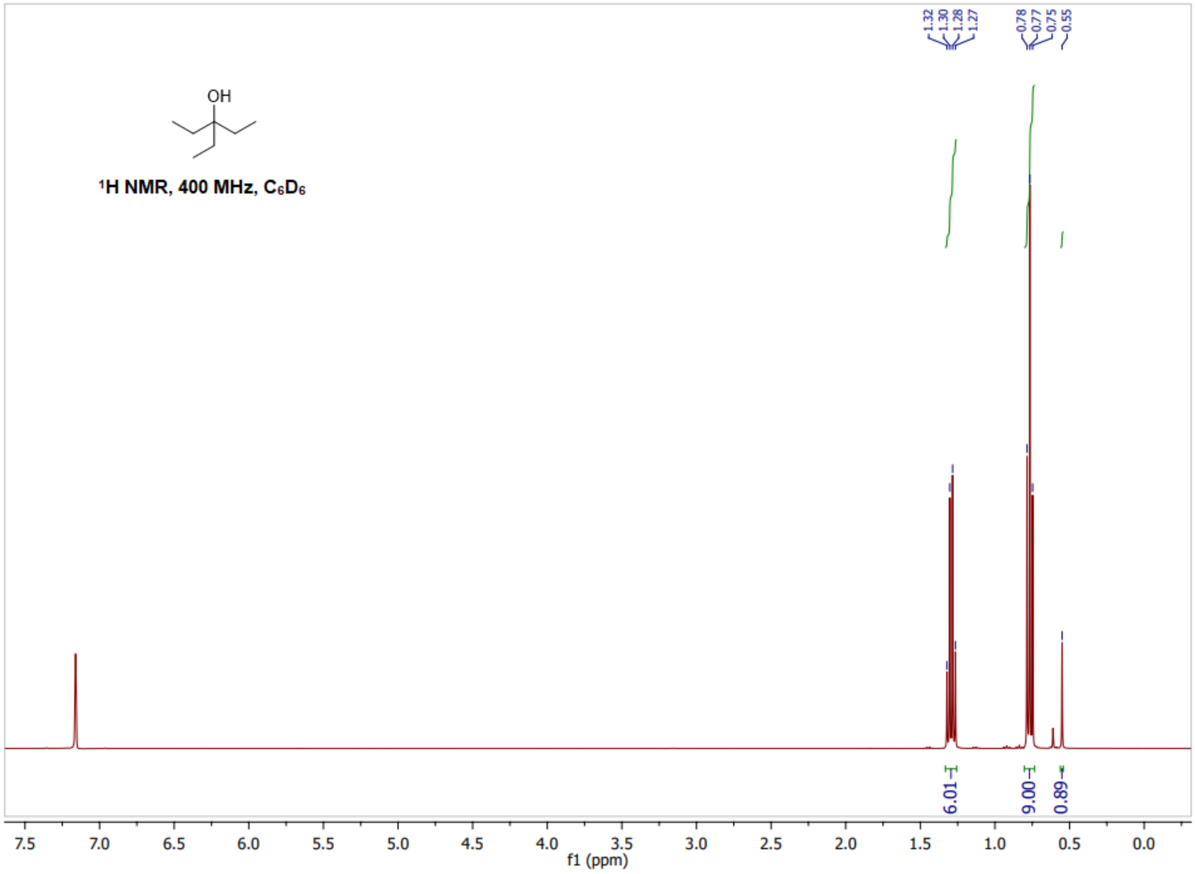
OH
1H NMR, 400 MHz, C6D6
1.32
1.30
1.28
1.27
0.78
0.77
0.75
0.55
6.01
9.00
0.89
7.5
7.0
6.5
6.0
5.5
5.0
4.5
4.0
3.5
3.0
2.5
2.0
1.5
1.0
0.5
0.0
f1 (ppm)

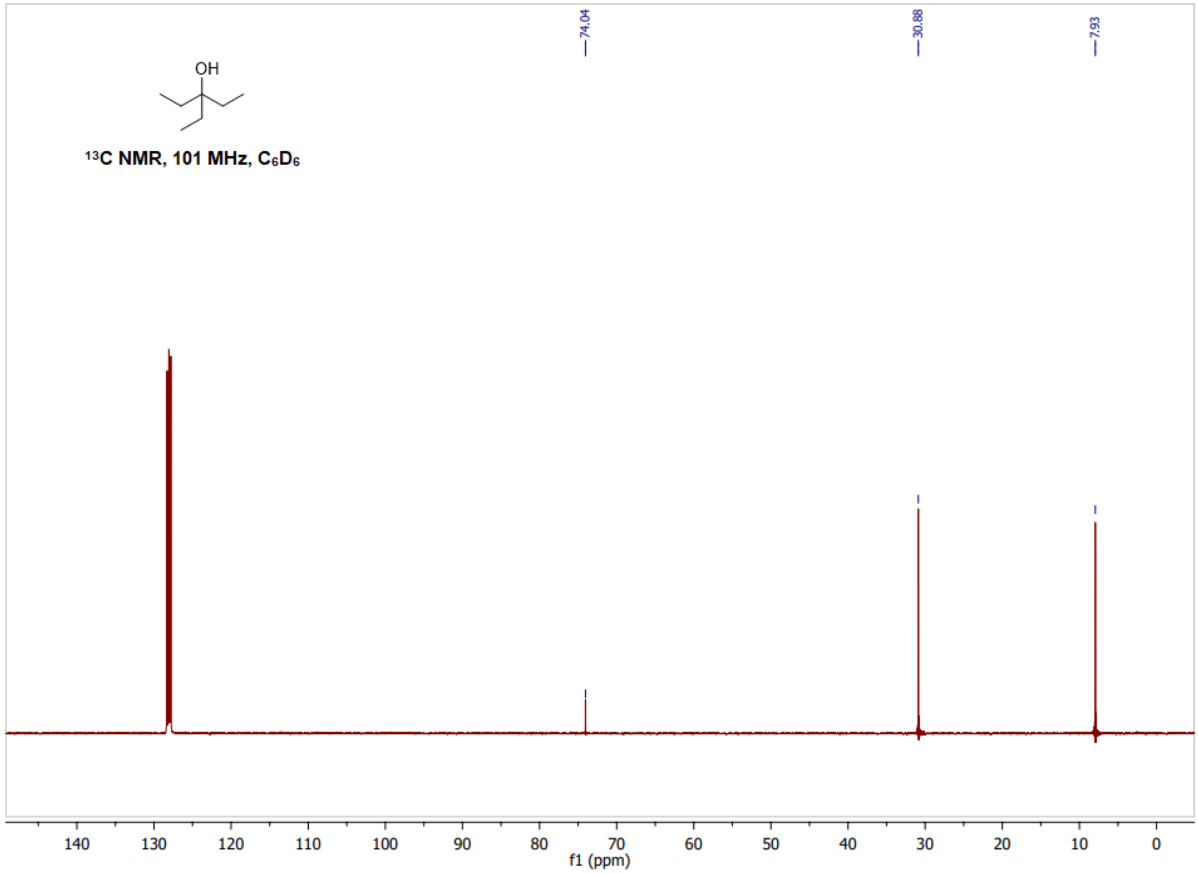
OH
13C NMR, 101 MHz, C6D6
74.04
30.88
7.93
140
130
120
110
100
90
80
70
60
50
40
30
20
10
0
f1 (ppm)

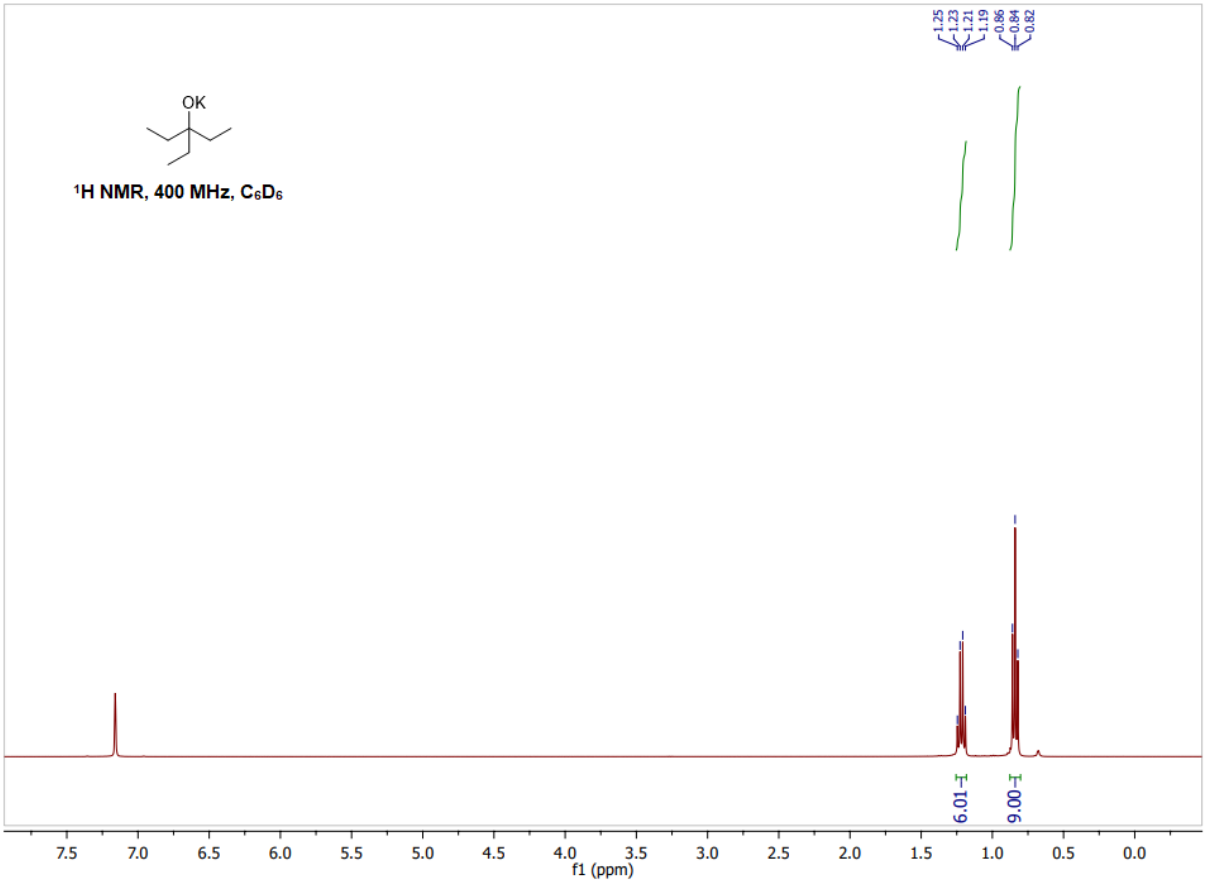
1.25
1.23
1.21
1.19
0.86
0.84
0.82
OK
¹H NMR, 400 MHz, C6D6
6.01
9.00
7.5 7.0 6.5 6.0 5.5 5.0 4.5 4.0 3.5 3.0 2.5 2.0 1.5 1.0 0.5 0.0
f1 (ppm)

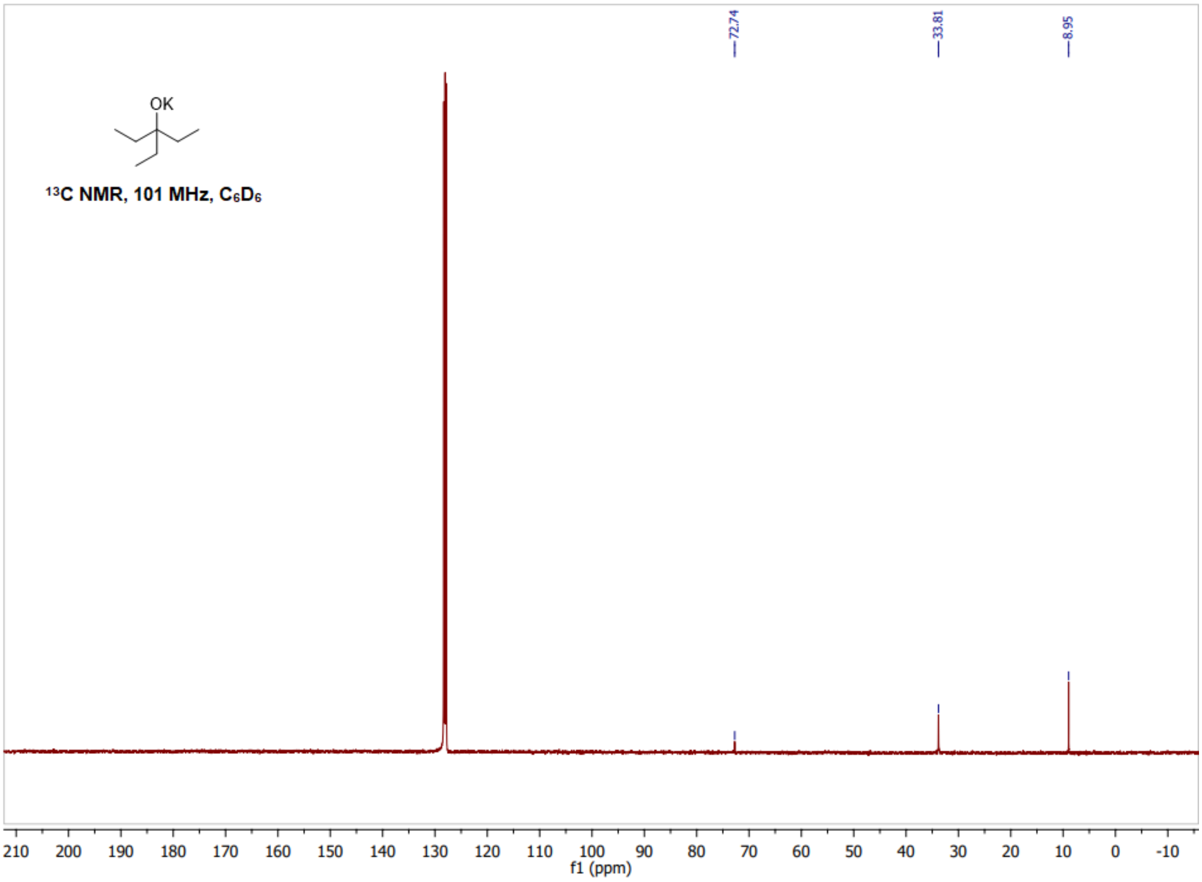
72.74
33.81
8.95
OK
¹³C NMR, 101 MHz, C6D6
210 200 190 180 170 160 150 140 130 120 110 100 90 80 70 60 50 40 30 20 10 0 -10
f1 (ppm)

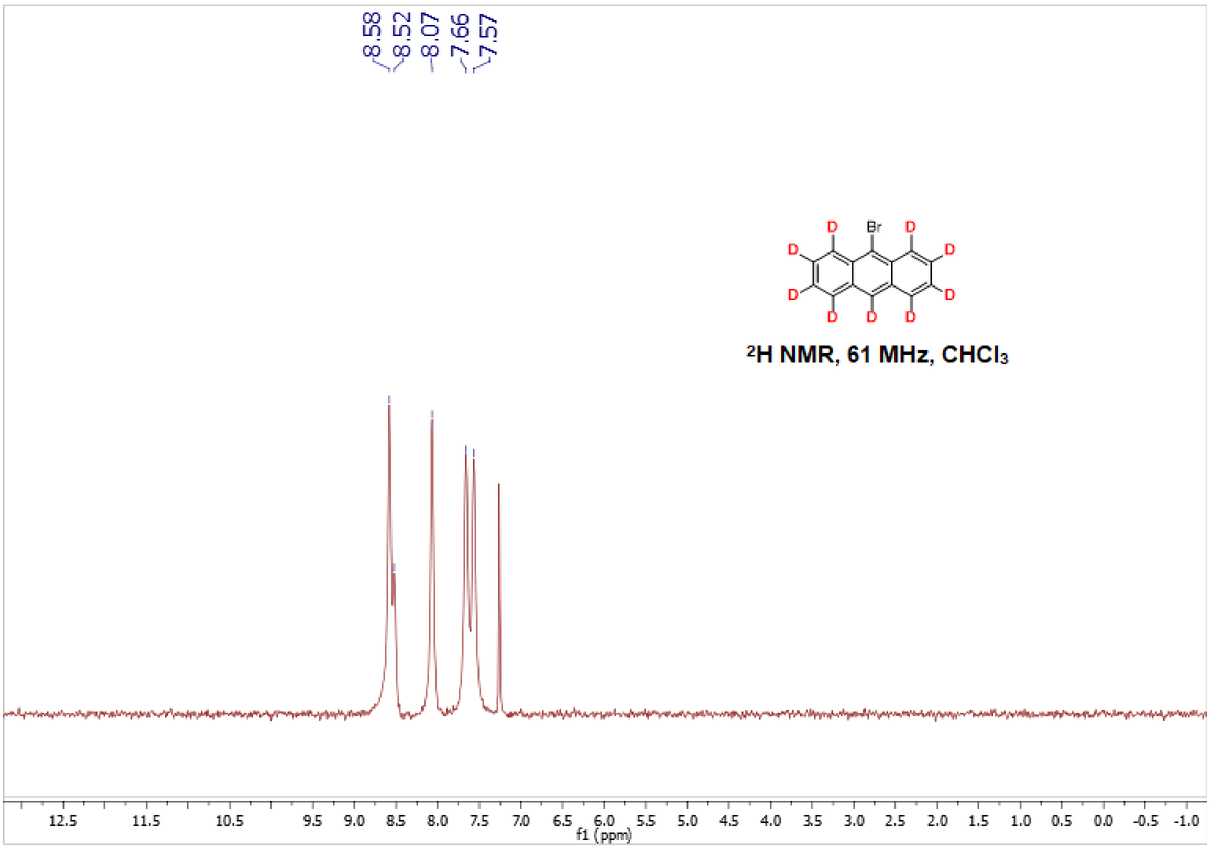
8.58
8.52
8.07
7.66
7.57
^{2}H NMR, 61 MHz, $CHCl_3$
f1 (ppm)

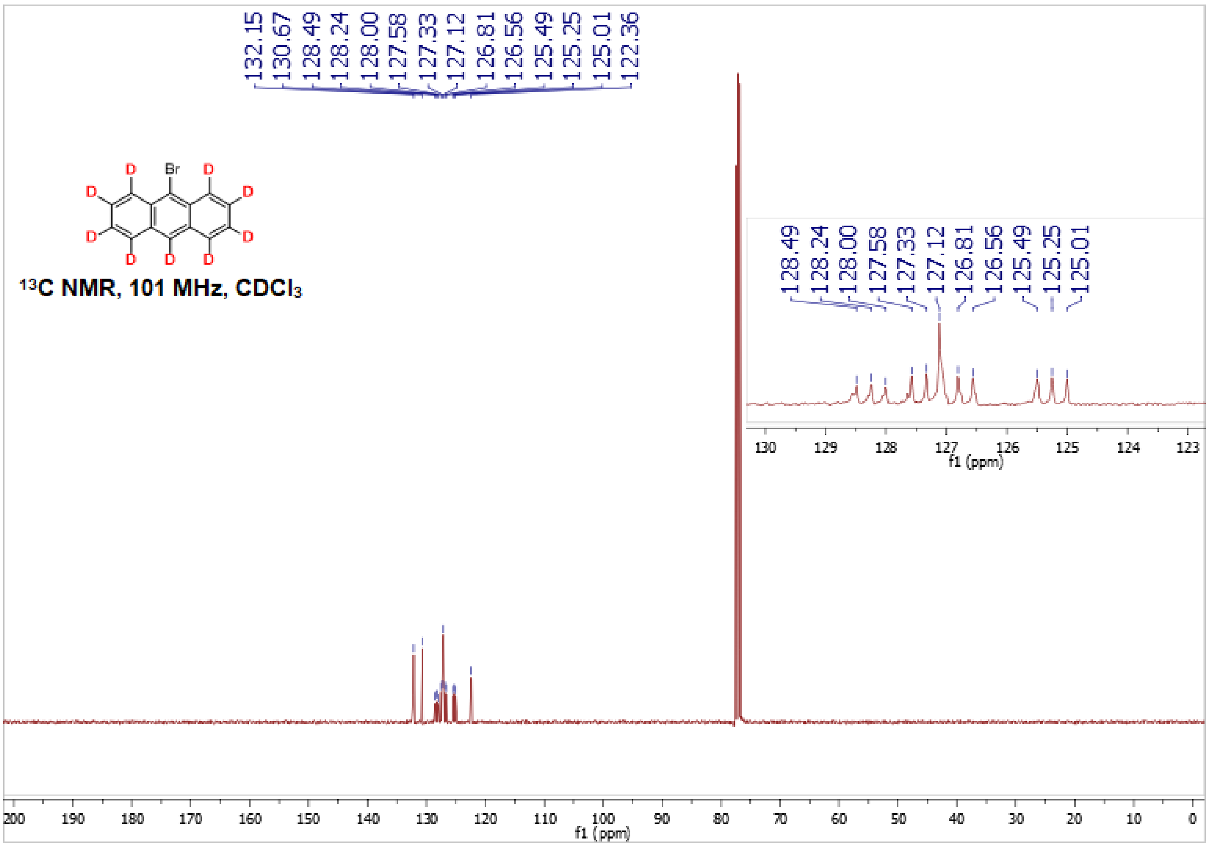
132.15
130.67
128.49
128.24
128.00
127.58
127.33
127.12
126.81
126.56
125.49
125.25
125.01
122.36
^{13}C NMR, 101 MHz, $CDCl_3$
f1 (ppm)

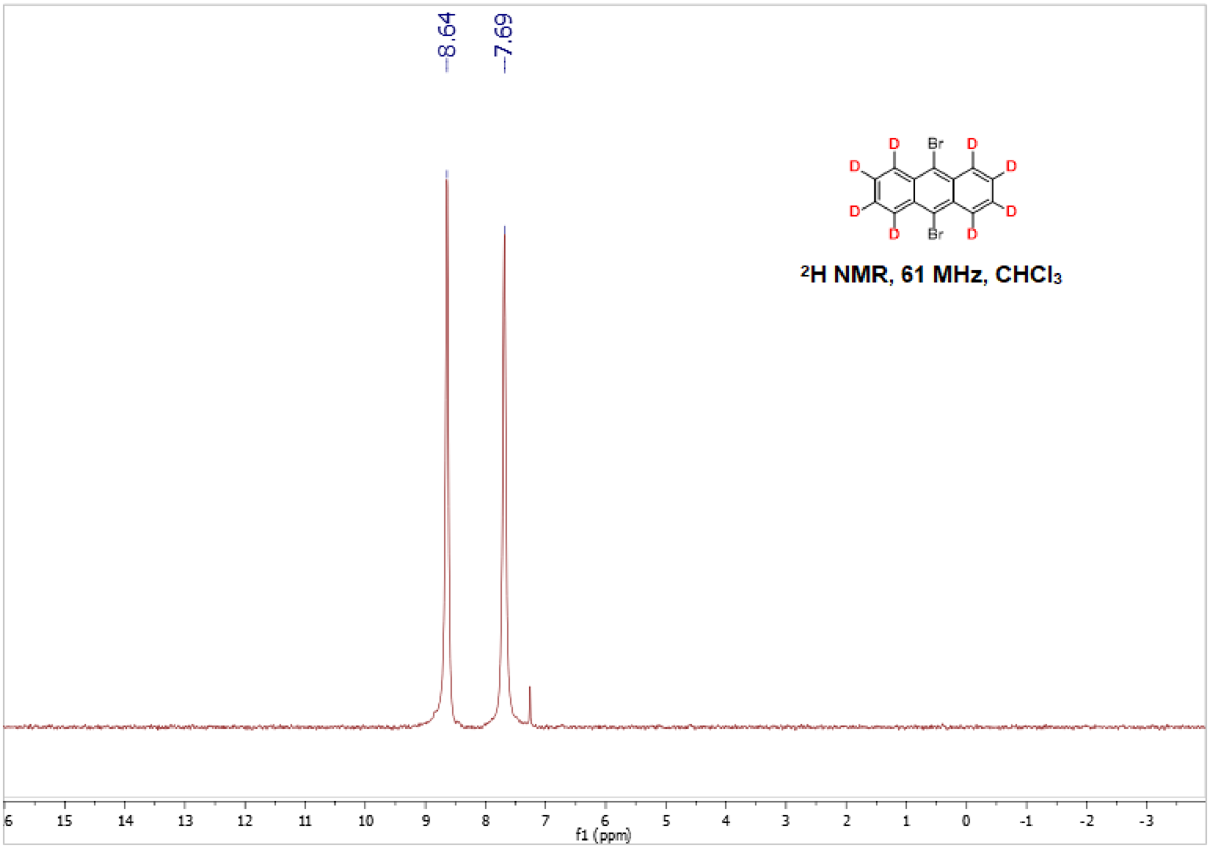
-8.64
-7.69
D
Br
D
D
D
D
D
D
Br
D
²H NMR, 61 MHz, CHCl₃
f1 (ppm)

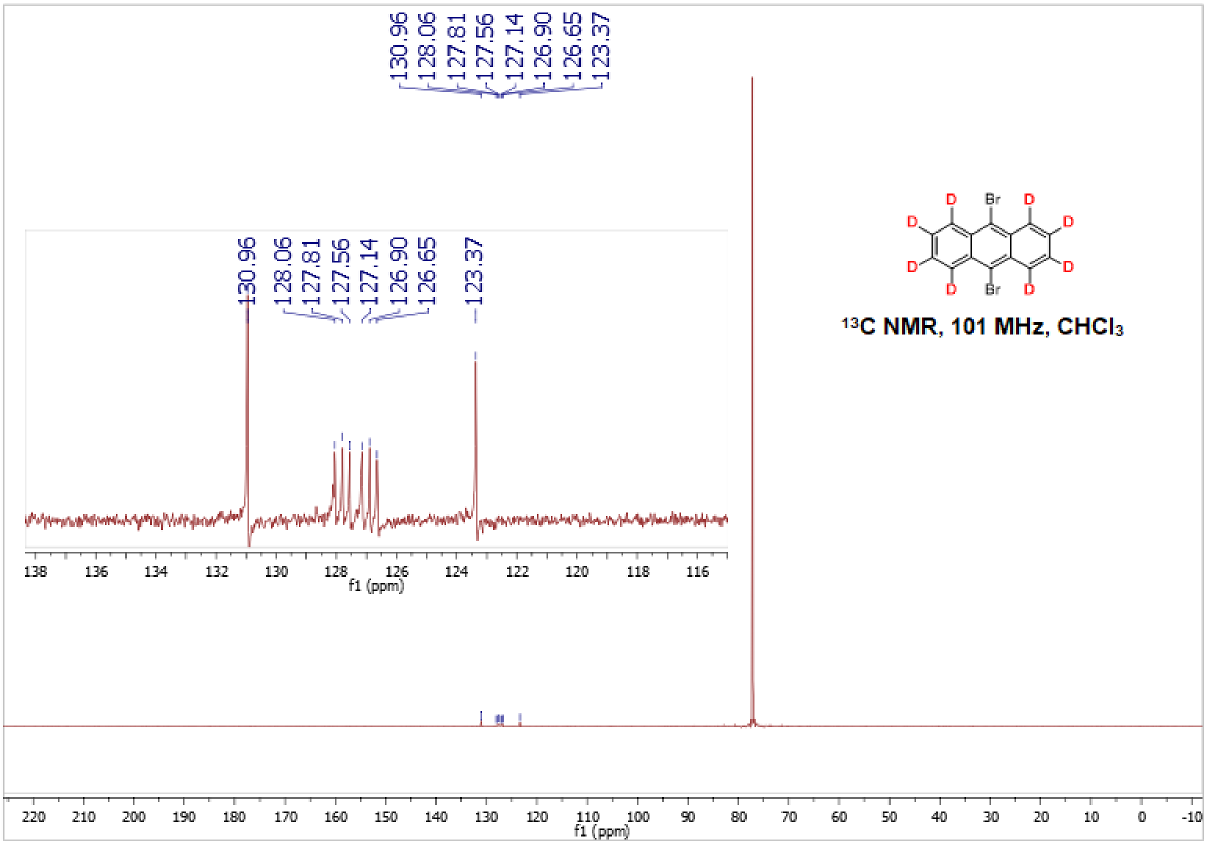
130.96
128.06
127.81
127.56
127.14
126.90
126.65
123.37
D
Br
D
D
D
D
D
D
Br
D
¹³C NMR, 101 MHz, CHCl₃
f1 (ppm)

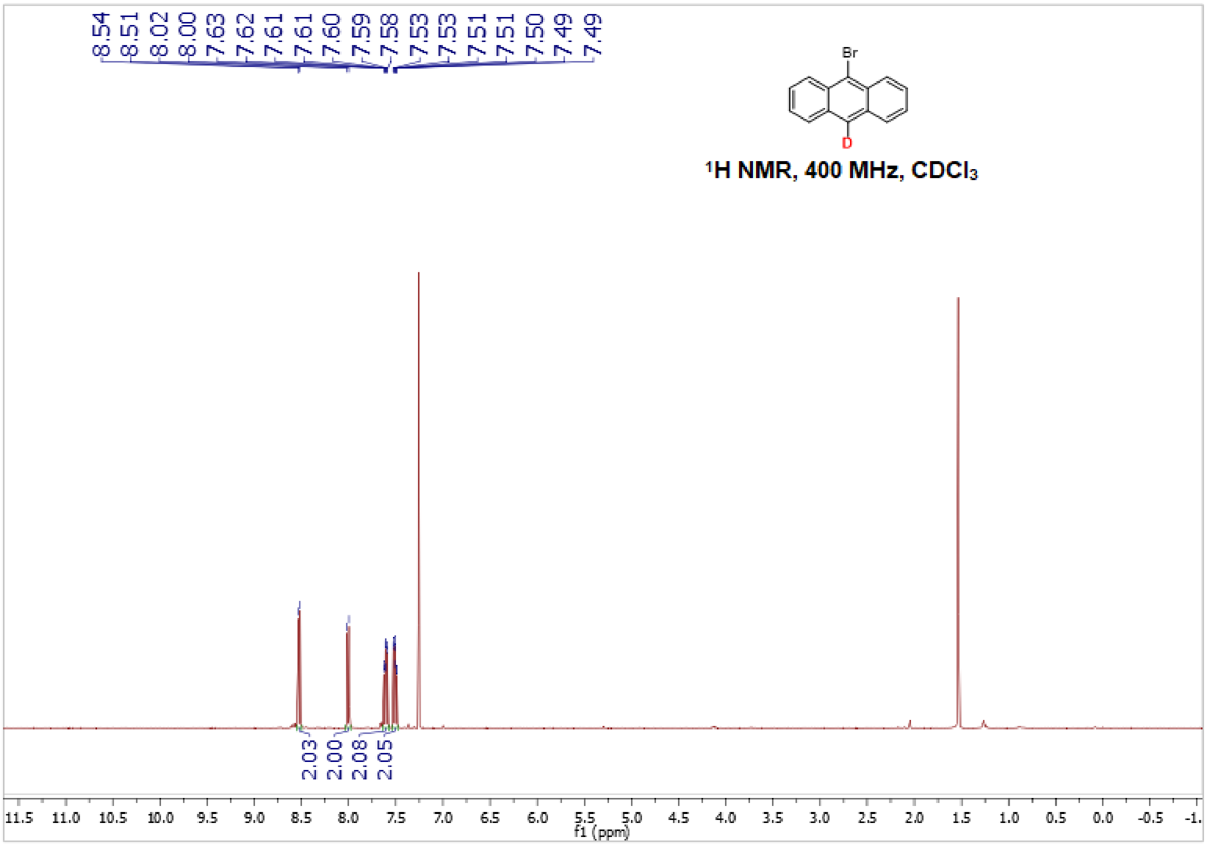
8.54
8.51
8.02
8.00
7.63
7.62
7.61
7.61
7.60
7.59
7.58
7.53
7.53
7.51
7.51
7.50
7.49
7.49
Br
D
¹H NMR, 400 MHz, CDCl₃
2.03
2.00
2.08
2.05
f1 (ppm)

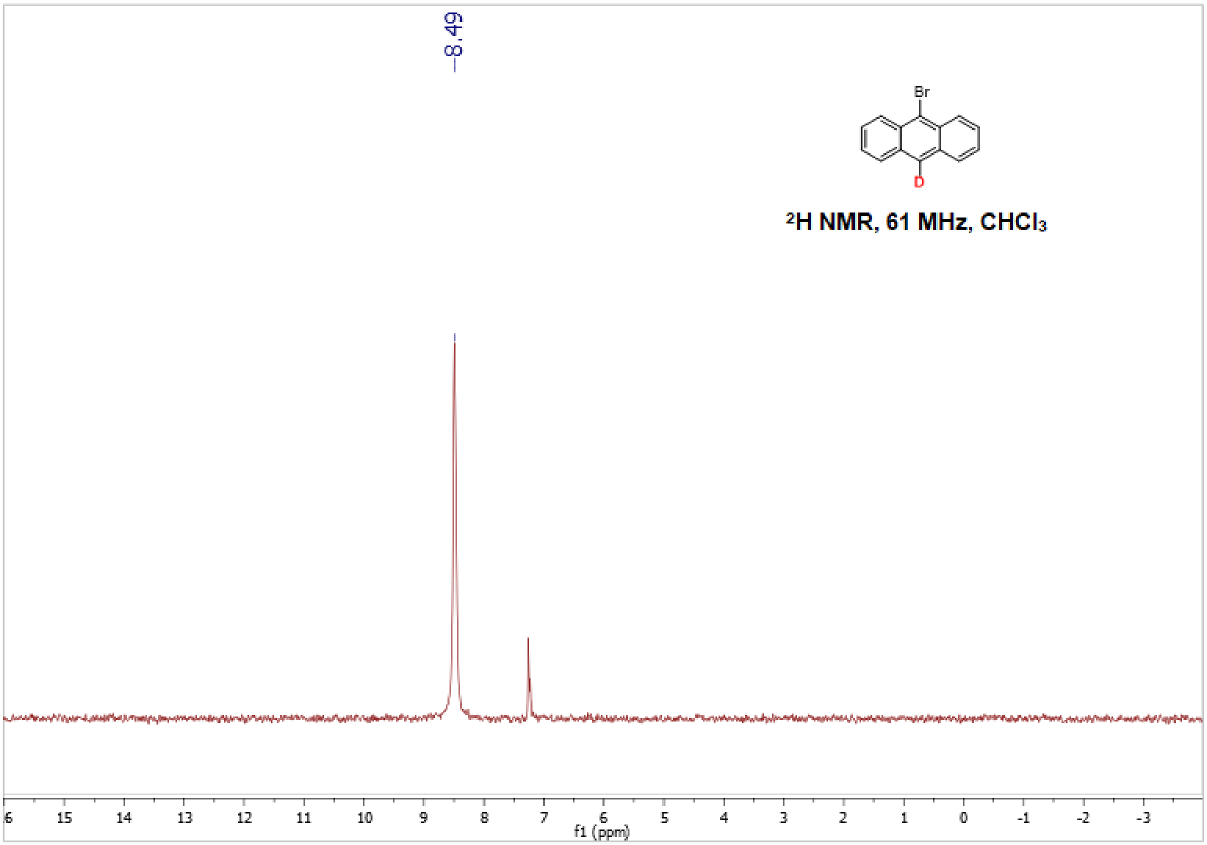
8.49
Br
D
²H NMR, 61 MHz, CHCl₃
f1 (ppm)

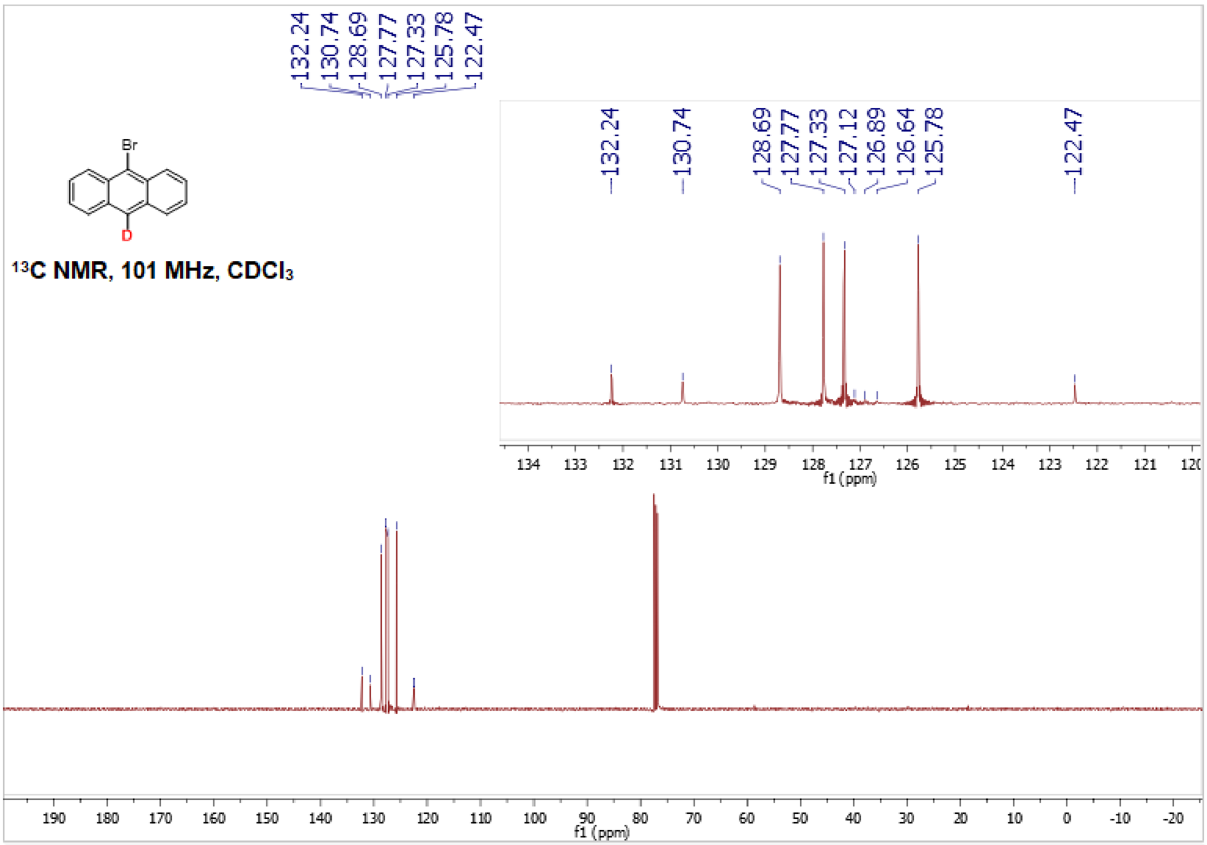
13C NMR, 101 MHz, CDCl3
Br
D
132.24
130.74
128.69
127.77
127.33
125.78
122.47
132.24
130.74
128.69
127.77
127.33
127.12
126.89
126.64
125.78
122.47
f1 (ppm)
f1 (ppm)

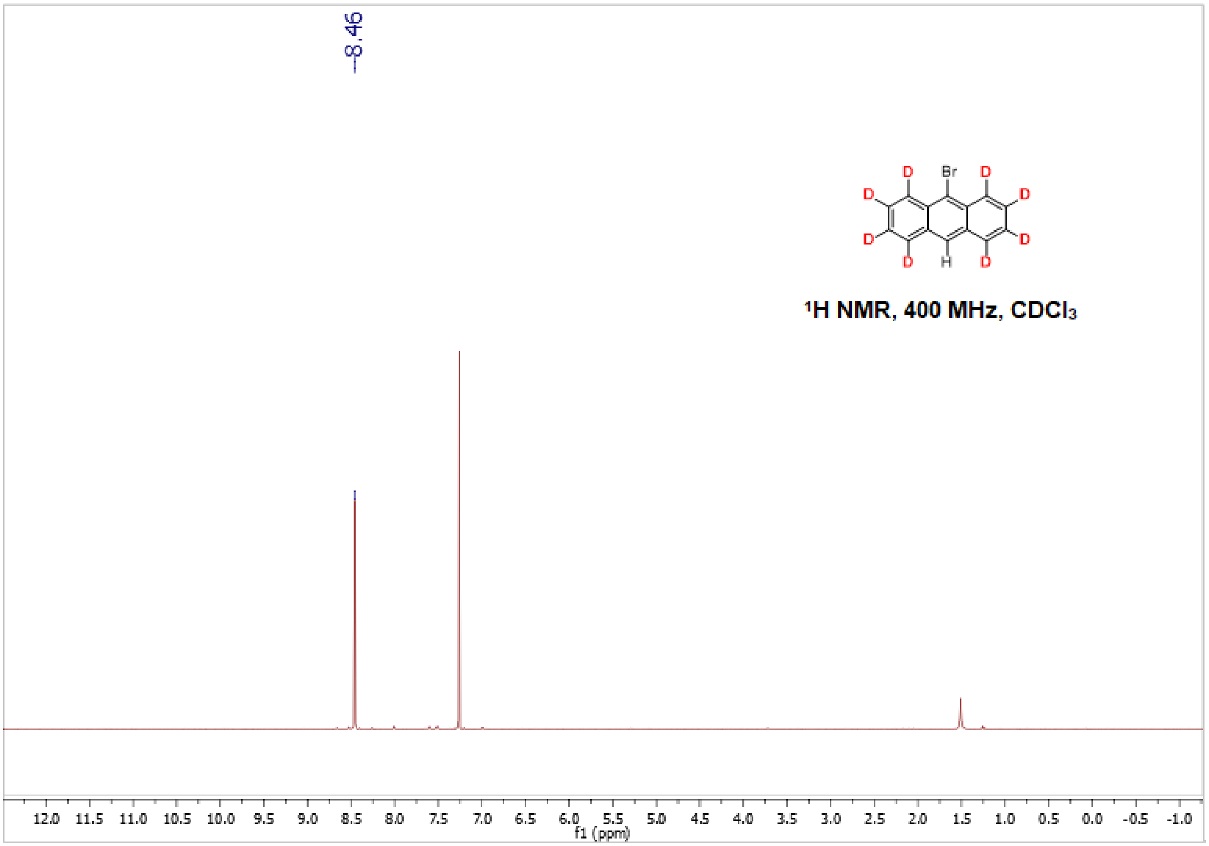
8.46
1H NMR, 400 MHz, CDCl3
Br
D
H
f1 (ppm)

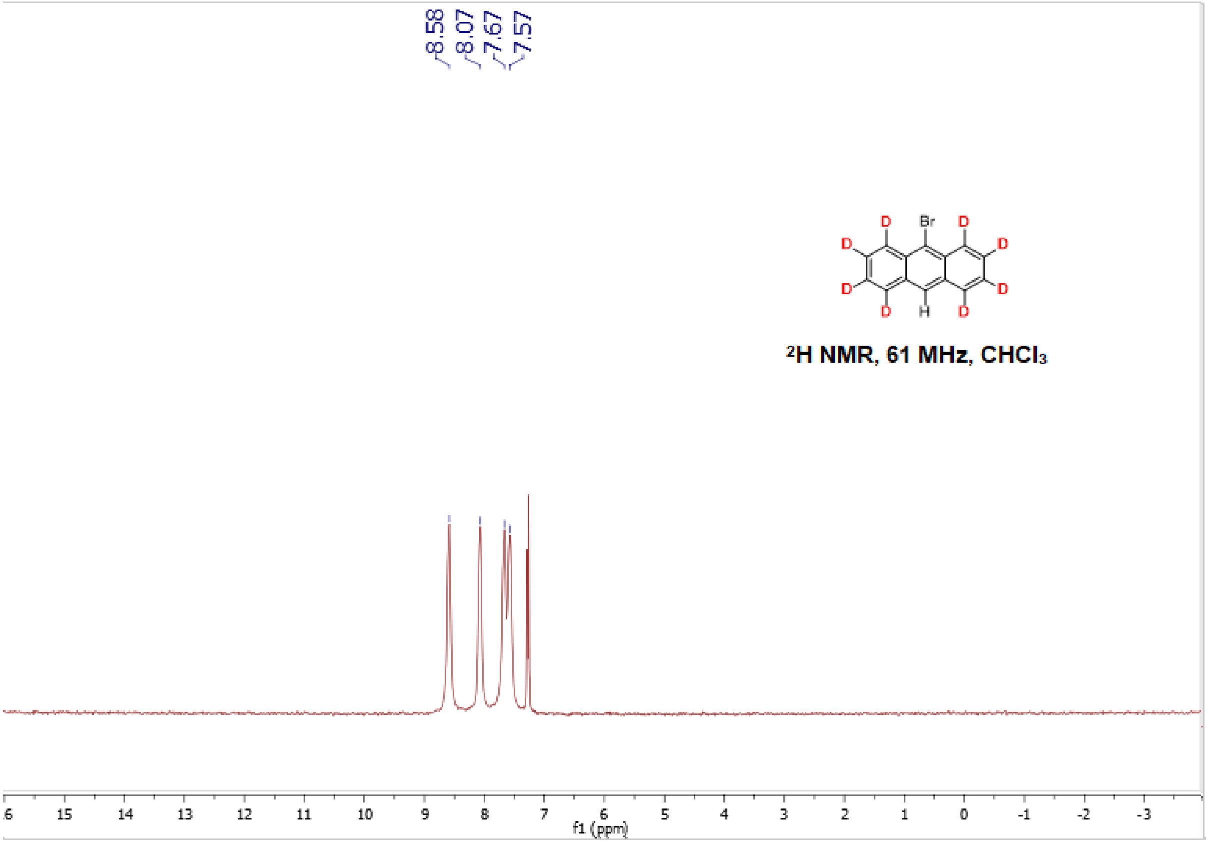
8.58
8.07
7.67
7.57
Br
D
H
2H NMR, 61 MHz, CHCl3
f1 (ppm)

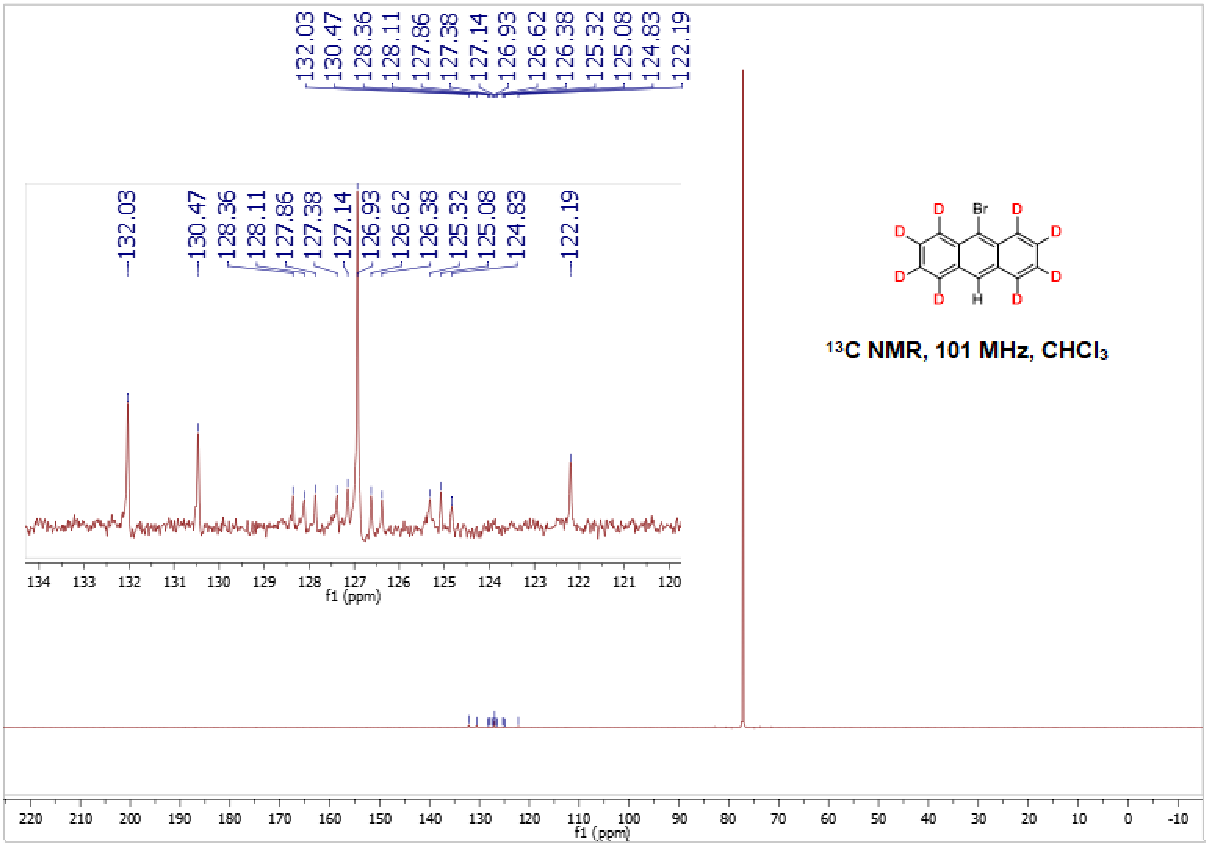
132.03
130.47
128.36
128.11
127.86
127.38
127.14
126.93
126.62
126.38
125.32
125.08
124.83
122.19
Br
D
H
13C NMR, 101 MHz, CHCl3
f1 (ppm)

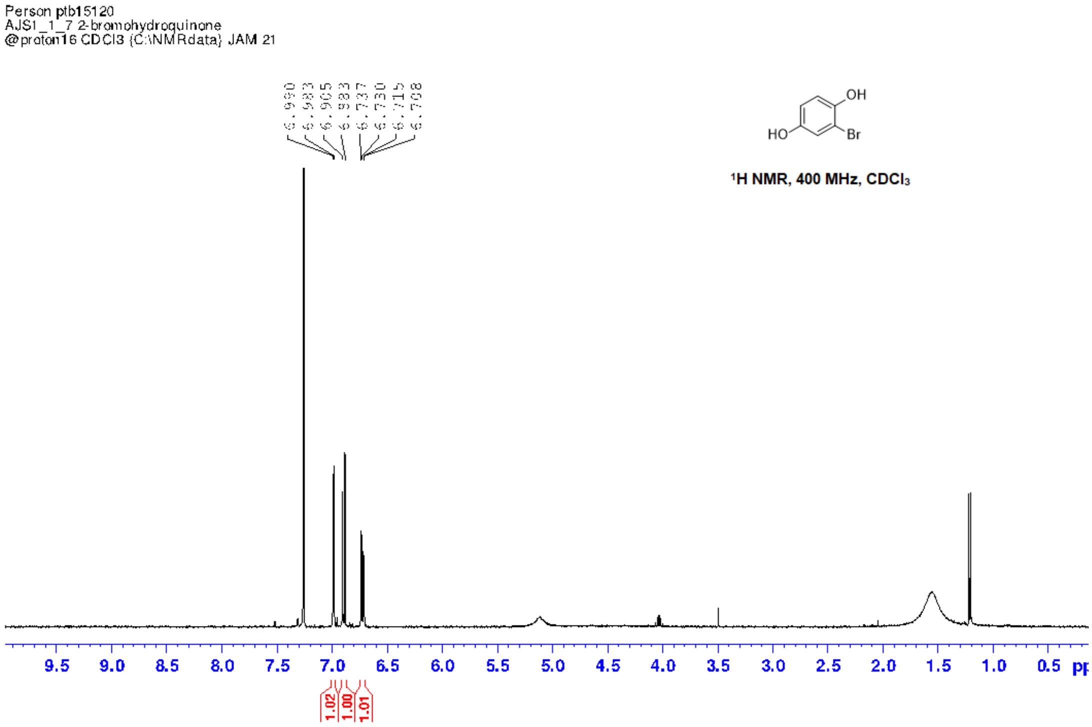

Person ptb15120
AJS1_1_7 2-bromohydroquinone
@proton16 CDCl3 {C:\NMRdata} JAM 21
6.990
6.983
6.965
6.883
6.737
6.730
6.715
6.708
OH
HO
Br
1H NMR, 400 MHz, CDCl3
9.5
9.0
8.5
8.0
7.5
7.0
6.5
6.0
5.5
5.0
4.5
4.0
3.5
3.0
2.5
2.0
1.5
1.0
0.5
1.02
1.00
1.01

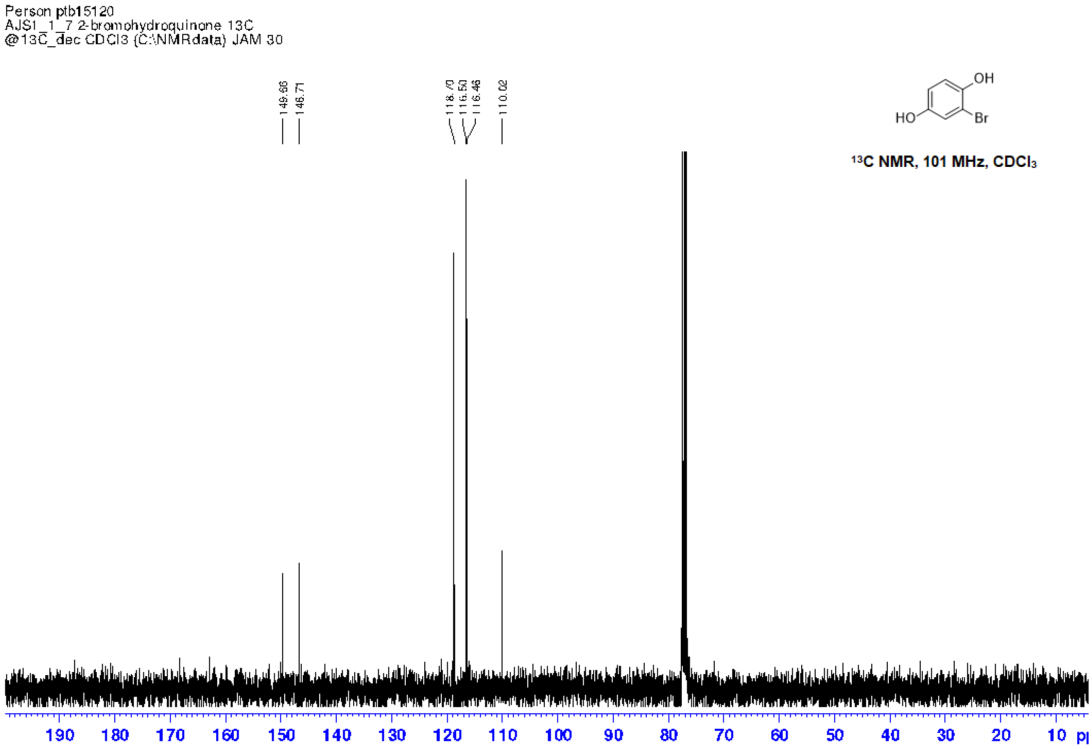

Person ptb15120
AJS1_1_7 2-bromohydroquinone 13C
@13C_dec CDCl3 {C:\NMRdata} JAM 30
149.66
146.71
118.70
116.50
116.46
110.02
OH
HO
Br
13C NMR, 101 MHz, CDCl3
190
180
170
160
150
140
130
120
110
100
90
80
70
60
50
40
30
20
10

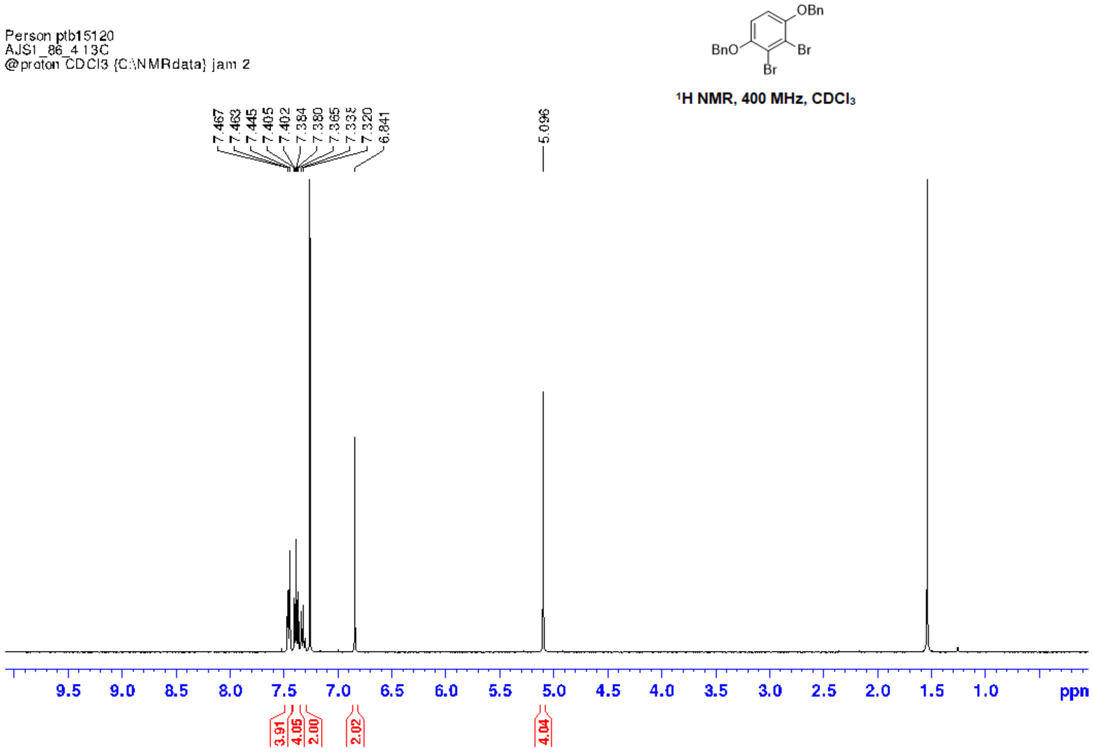
Person ptb15120
AJS1_86_4 13C
@proton CDCl3 {C:\NMRdata} jam 2
OBn
BnO
Br
Br
¹H NMR, 400 MHz, CDCl₃
7.467
7.463
7.445
7.405
7.402
7.384
7.380
7.365
7.338
7.320
6.841
5.096
9.5
9.0
8.5
8.0
7.5
7.0
6.5
6.0
5.5
5.0
4.5
4.0
3.5
3.0
2.5
2.0
1.5
1.0
ppm
3.91
4.05
2.00
2.02
4.04

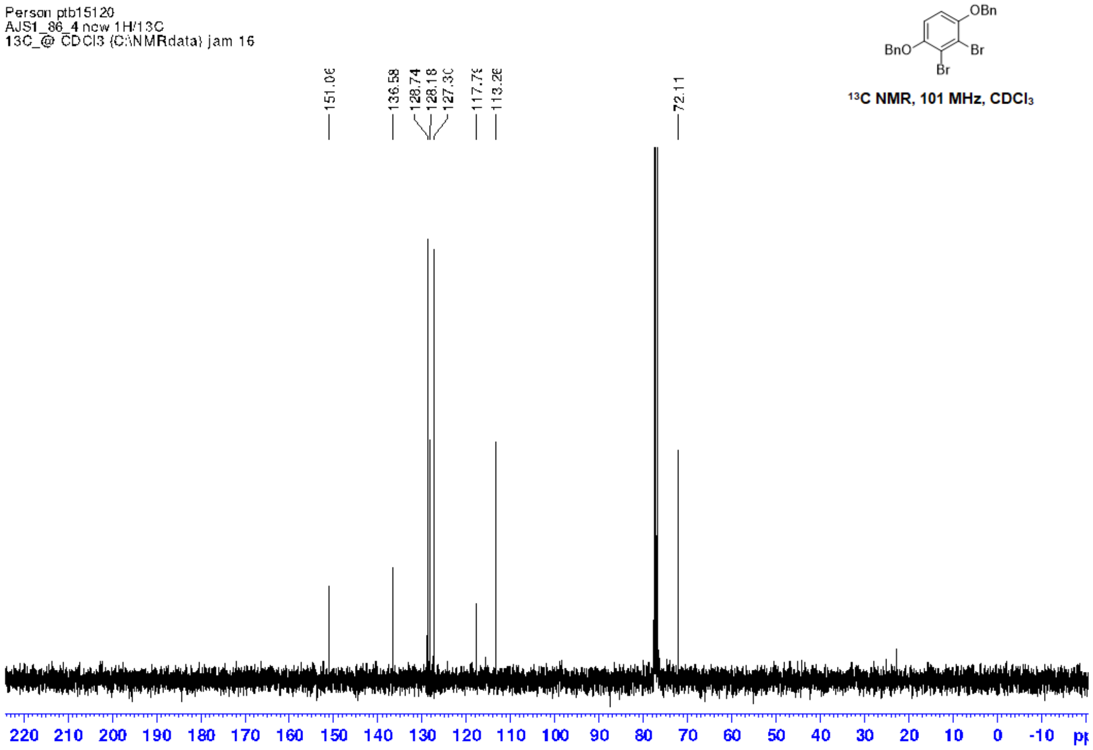
Person ptb15120
AJS1_86_4 now 1H/13C
13C_@ CDCl3 {C:\NMRdata} jam 16
OBn
BnO
Br
Br
¹³C NMR, 101 MHz, CDCl₃
151.06
136.58
128.74
128.18
127.30
117.79
113.26
72.11
220
210
200
190
180
170
160
150
140
130
120
110
100
90
80
70
60
50
40
30
20
10
0
-10
pp

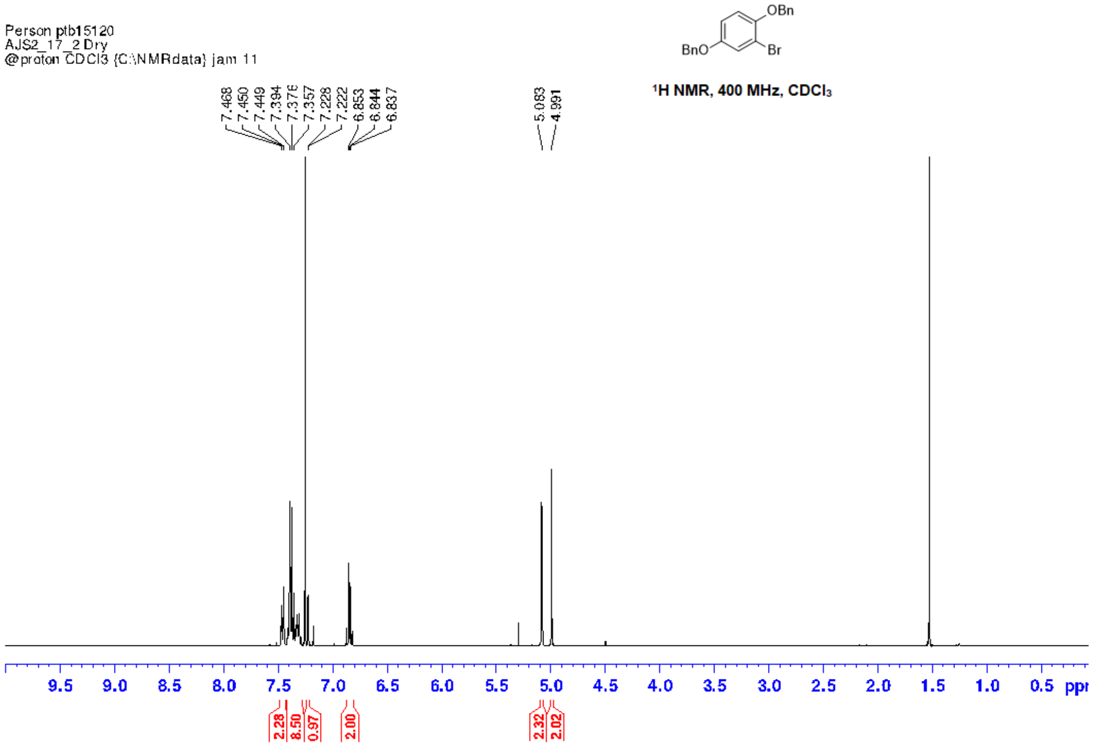
Person ptb15120
AJS2_17_2 Dry
@proton CDCl3 {C:\NMRdata} jam 11
OBn
BnO
Br
¹H NMR, 400 MHz, CDCl₃
7.468
7.450
7.449
7.394
7.376
7.357
7.228
7.222
6.853
6.844
6.837
5.083
4.991
2.28
8.50
0.97
2.00
2.32
2.02
9.5 9.0 8.5 8.0 7.5 7.0 6.5 6.0 5.5 5.0 4.5 4.0 3.5 3.0 2.5 2.0 1.5 1.0 0.5 ppm

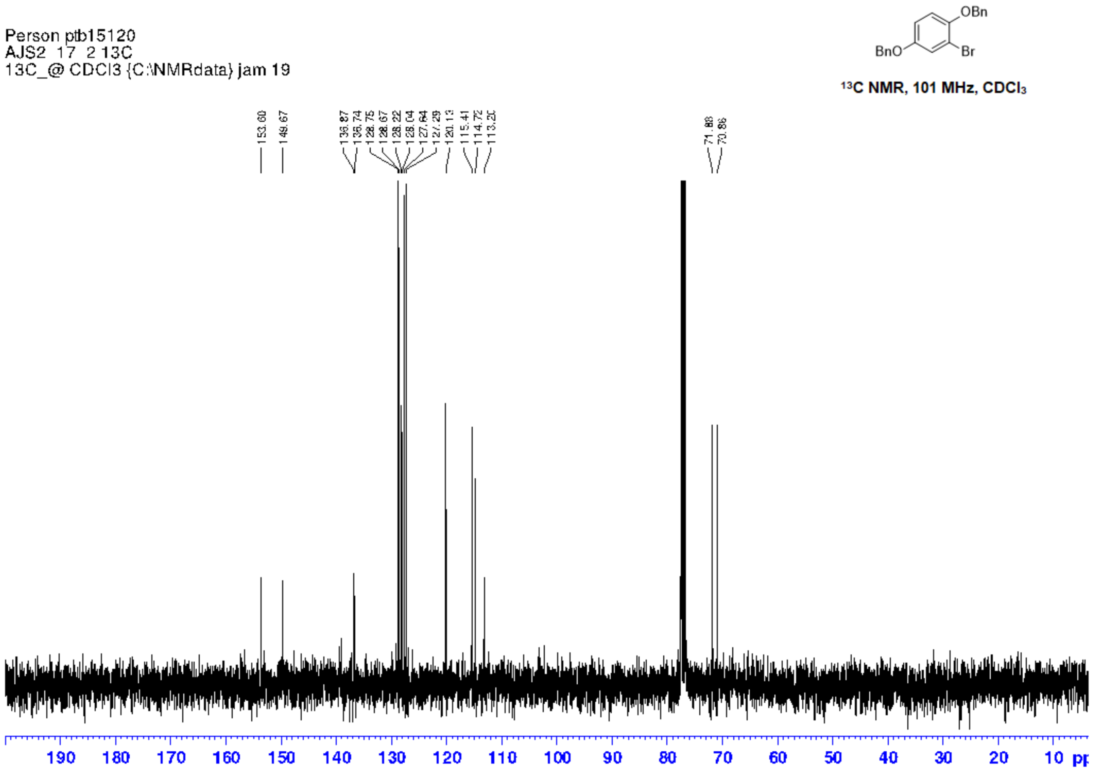
Person ptb15120
AJS2 17 2 13C
13C_@ CDCl3 {C:\NMRdata} jam 19
OBn
BnO
Br
¹³C NMR, 101 MHz, CDCl₃
153.60
149.67
136.87
136.74
128.75
128.67
128.22
128.04
127.64
127.29
120.13
115.41
114.72
113.35
71.88
70.86
190 180 170 160 150 140 130 120 110 100 90 80 70 60 50 40 30 20 10 ppm

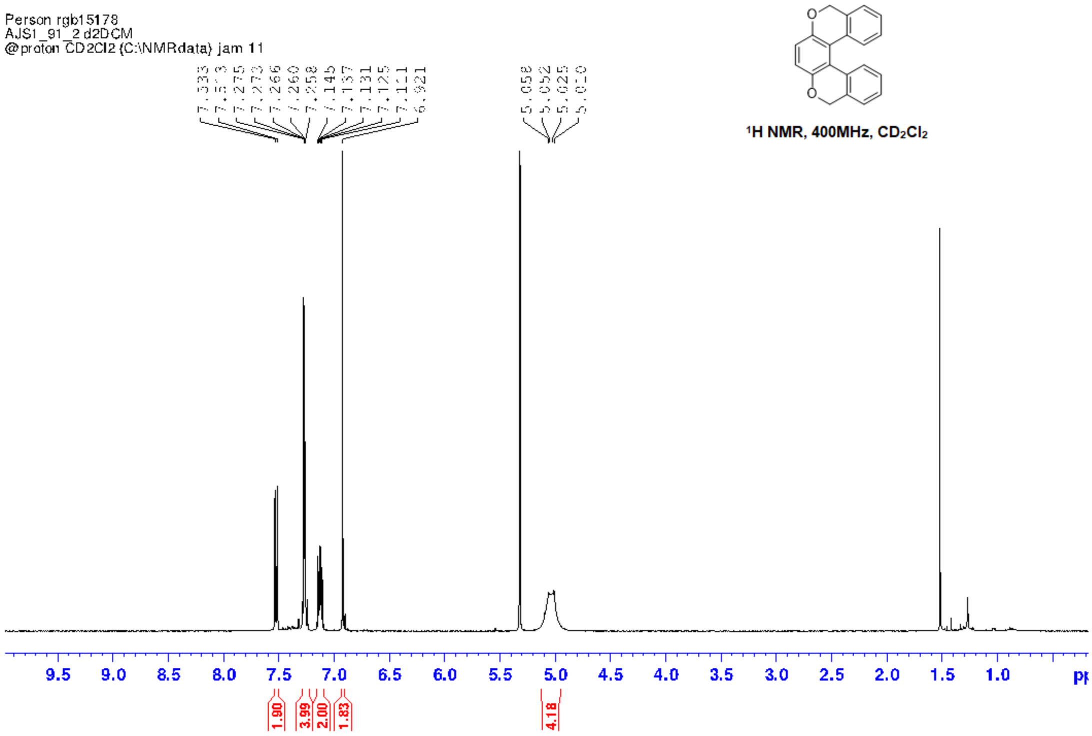
Person rgb15178
AJS1_91_2 d2DCM
@proton CD2Cl2 {C:\NMRdata} jam 11
7.533
7.275
7.273
7.266
7.260
7.258
7.145
7.137
7.131
7.125
6.921
5.058
5.052
5.025
1H NMR, 400MHz, CD2Cl2
O
O
9.5 9.0 8.5 8.0 7.5 7.0 6.5 6.0 5.5 5.0 4.5 4.0 3.5 3.0 2.5 2.0 1.5 1.0
1.90 3.99 2.00 1.83 4.18

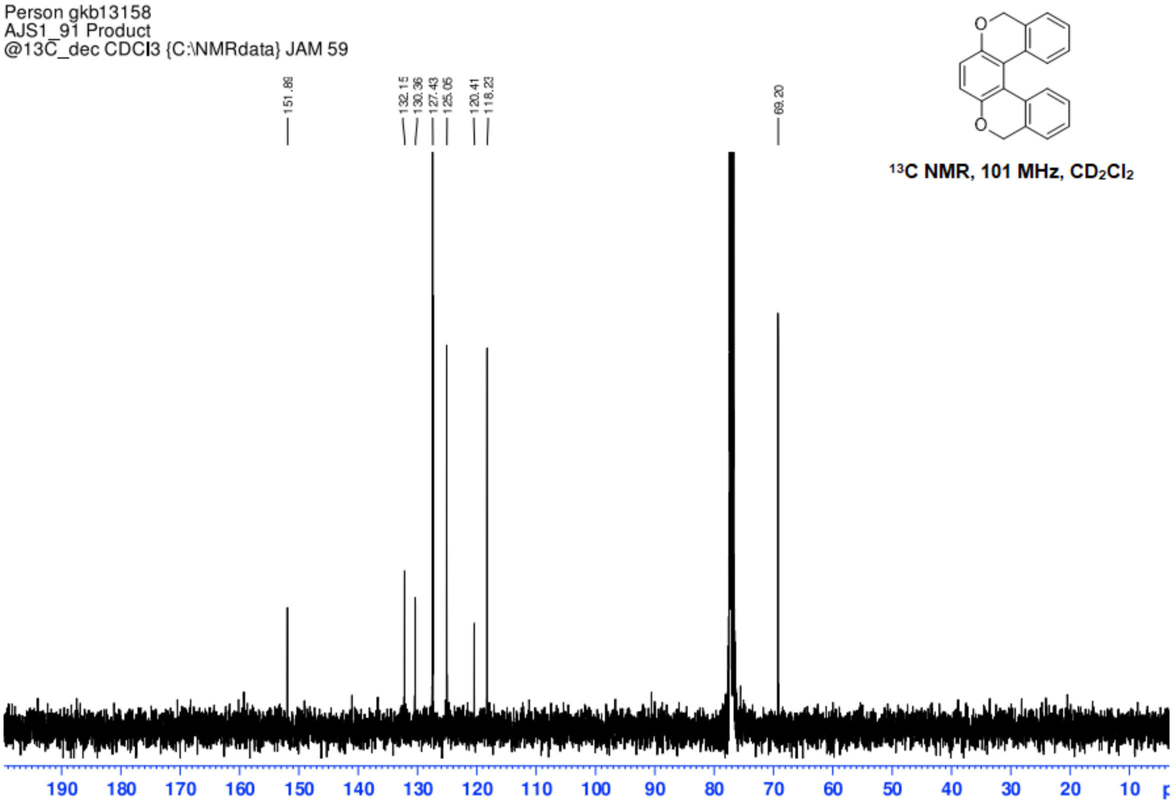
Person gkb13158
AJS1_91 Product
@13C_dec CDCl3 {C:\NMRdata} JAM 59
151.89
132.15
130.36
127.43
125.05
120.41
118.23
69.20
O
O
13C NMR, 101 MHz, CD2Cl2
190 180 170 160 150 140 130 120 110 100 90 80 70 60 50 40 30 20 10

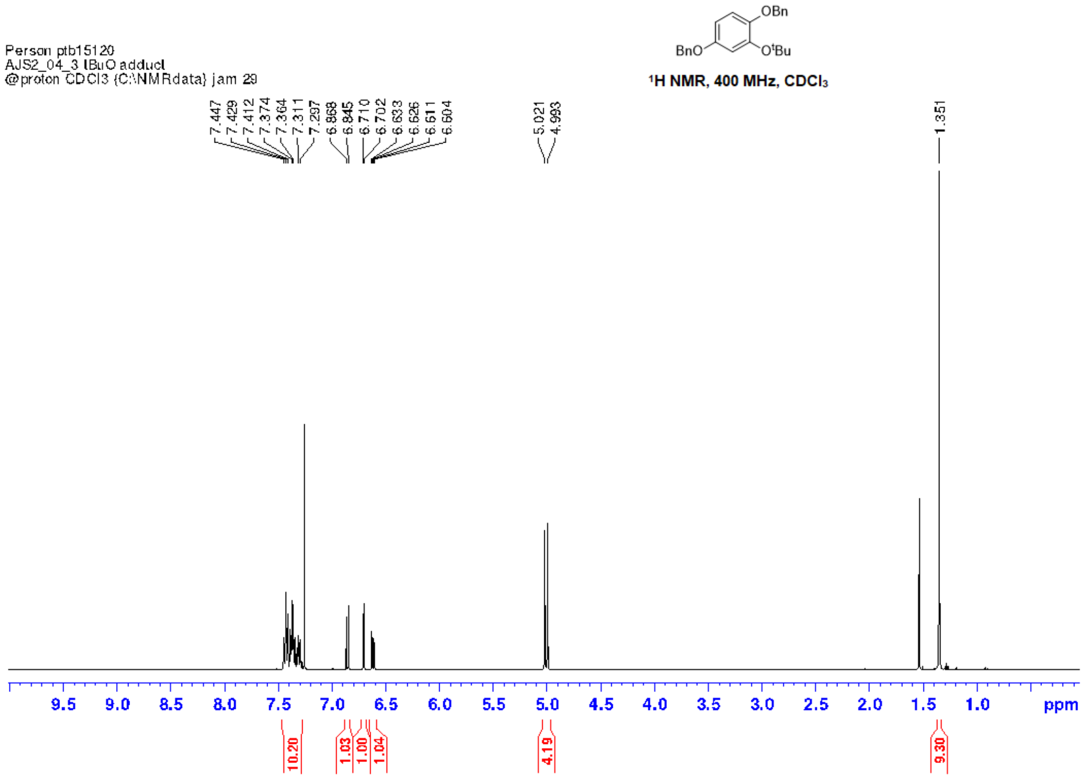
Person ptb15120
AJS2_04_3 tBuO adduct
@proton CDCl3 {C:\NMRdata} jam 29
OBn
BnO
O^{t}Bu
^{1}H NMR, 400 MHz, CDCl$_3$
7.447 7.429 7.412 7.374 7.364 7.311 7.297 6.868 6.845 6.710 6.702 6.633 6.626 6.611 6.604
5.021 4.993
1.351
9.5 9.0 8.5 8.0 7.5 7.0 6.5 6.0 5.5 5.0 4.5 4.0 3.5 3.0 2.5 2.0 1.5 1.0 ppm
10.20 1.03 1.00 1.04 4.19 9.30

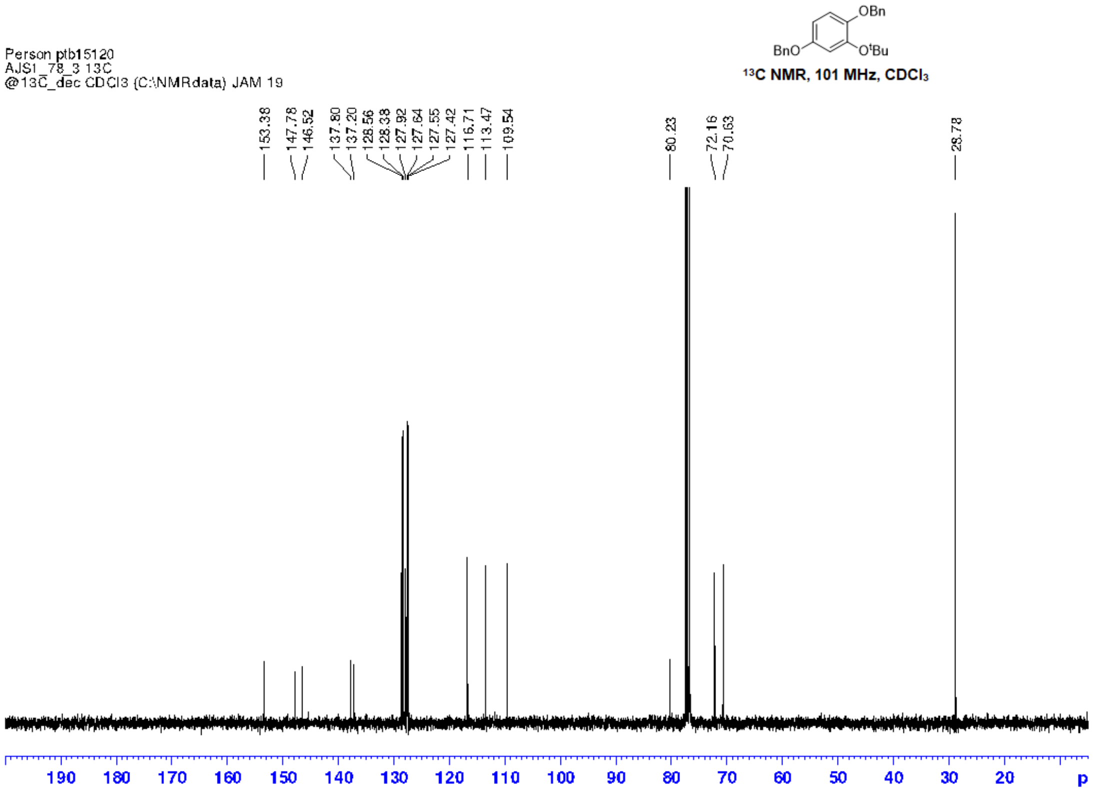
Person ptb15120
AJS1_78_3 13C
@13C_dec CDCl3 {C:\NMRdata} JAM 19
OBn
BnO
O^{t}Bu
^{13}C NMR, 101 MHz, CDCl$_3$
153.38 147.78 146.52 137.80 137.20 128.56 128.33 127.92 127.64 127.55 127.42 116.71 113.47 109.54
80.23 72.16 70.63
28.78
190 180 170 160 150 140 130 120 110 100 90 80 70 60 50 40 30 20 p

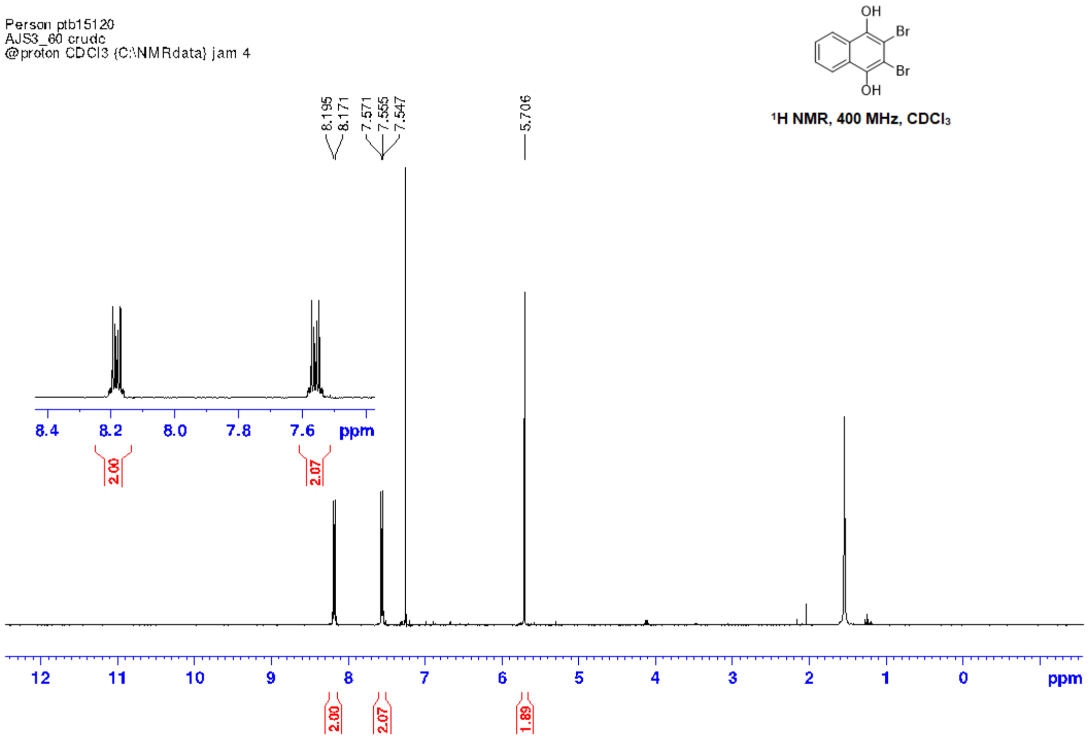
Person ptb15120
AJS3_60 crude
@proton CDCl3 {C:\NMRdata} jam 4
OH
Br
Br
OH
1H NMR, 400 MHz, CDCl3
8.195
8.171
7.571
7.555
7.547
5.706
2.00
2.07
1.89
ppm

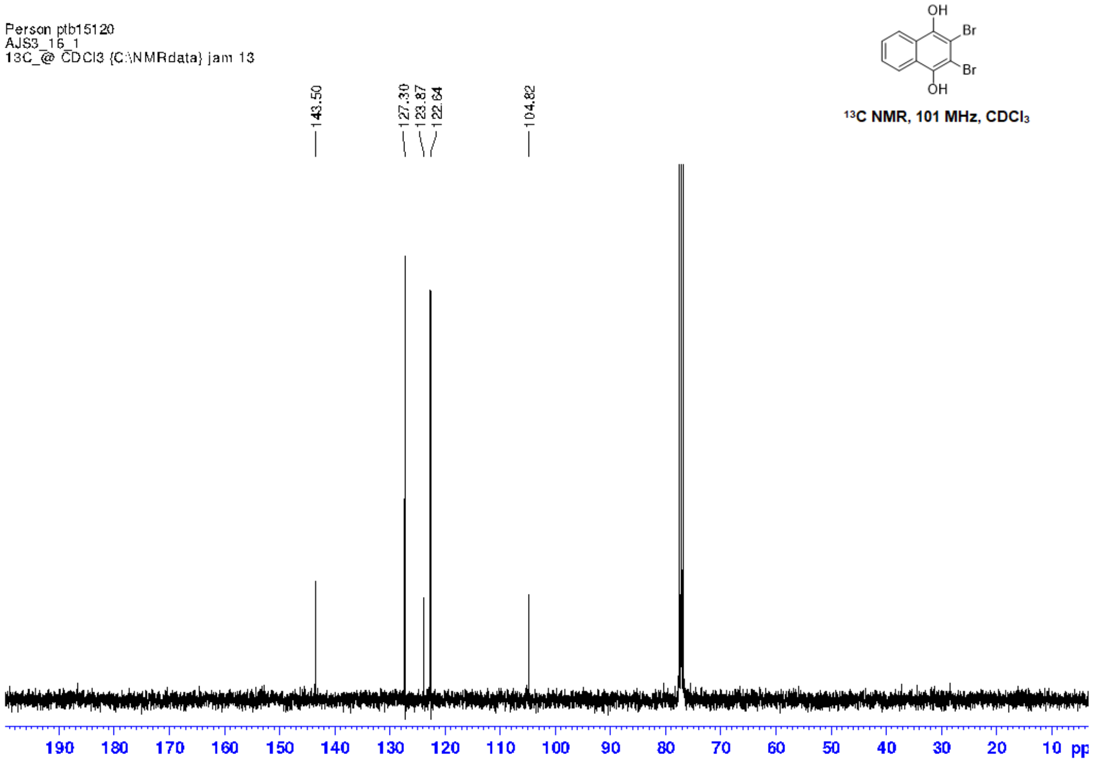
Person ptb15120
AJS3_16_1
13C_@ CDCl3 {C:\NMRdata} jam 13
OH
Br
Br
OH
13C NMR, 101 MHz, CDCl3
143.50
127.30
123.87
122.64
104.82

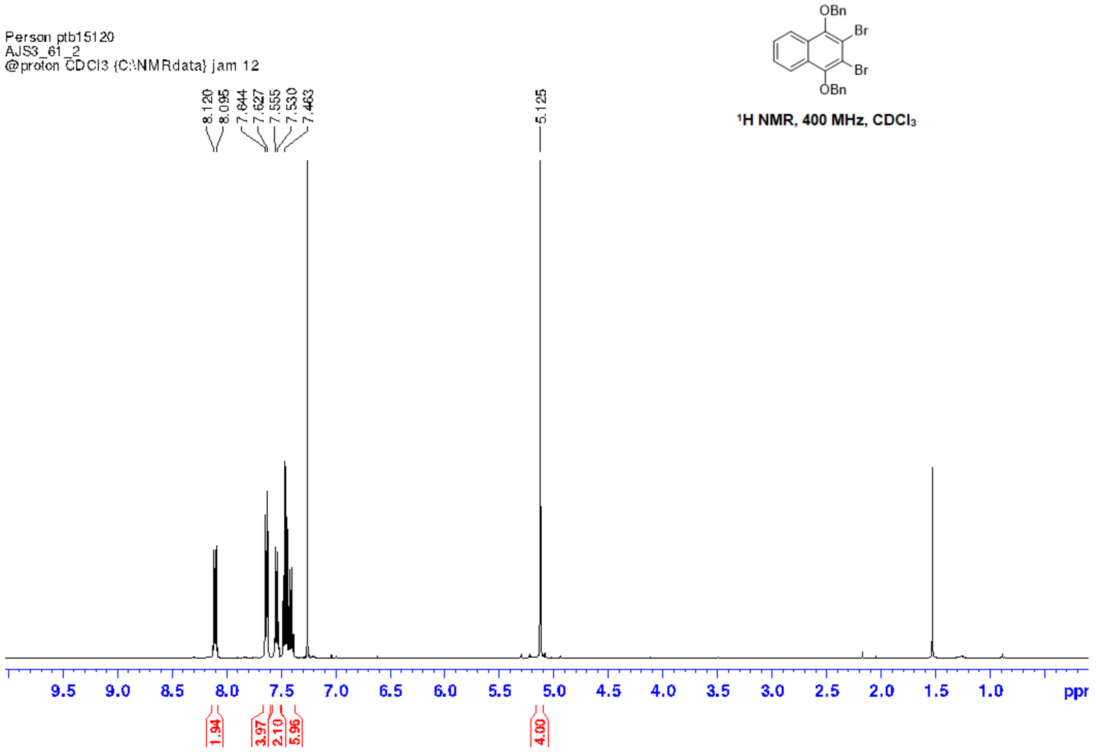
Person ptb15120
AJS3_61_2
@proton CDCl3 {C:\NMRdata} jam 12
8.120
8.095
7.644
7.627
7.555
7.530
7.463
5.125
OBn
Br
Br
OBn
1H NMR, 400 MHz, CDCl3
9.5
9.0
8.5
8.0
7.5
7.0
6.5
6.0
5.5
5.0
4.5
4.0
3.5
3.0
2.5
2.0
1.5
1.0
ppm
1.94
3.97
2.10
5.96
4.00

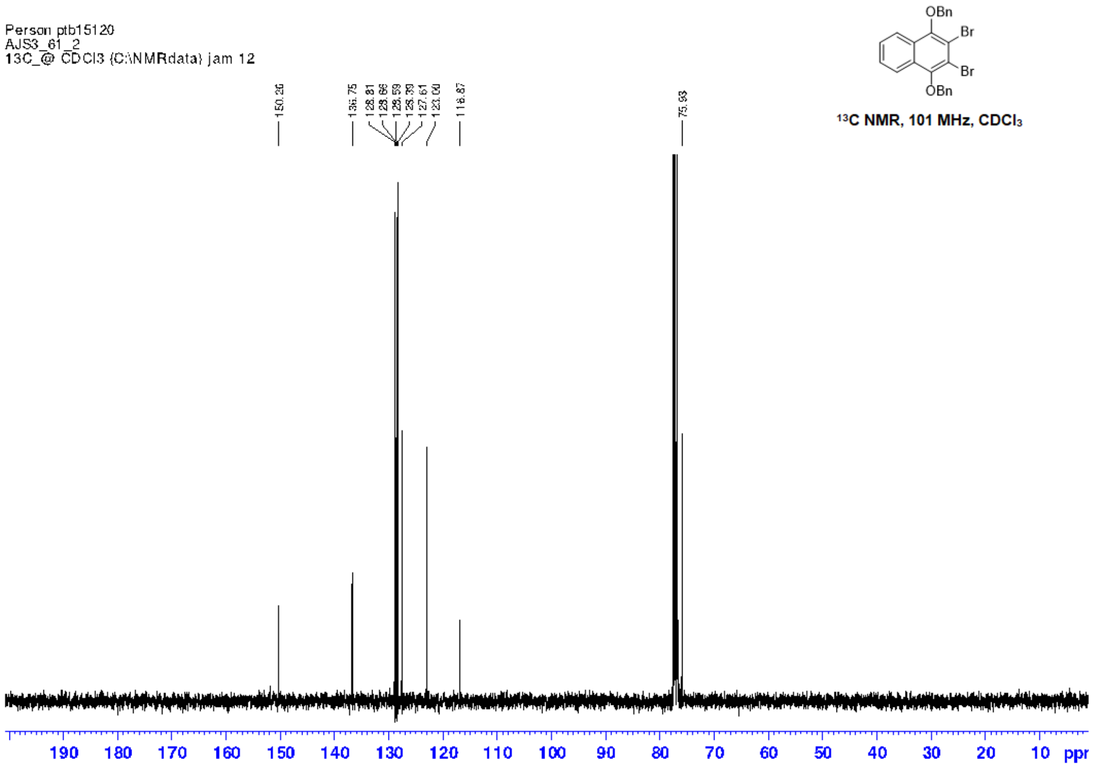
Person ptb15120
AJS3_61_2
13C_@ CDCl3 {C:\NMRdata} jam 12
150.26
136.75
128.81
128.66
128.59
128.39
127.61
123.00
116.87
75.93
OBn
Br
Br
OBn
13C NMR, 101 MHz, CDCl3
190
180
170
160
150
140
130
120
110
100
90
80
70
60
50
40
30
20
10
ppm

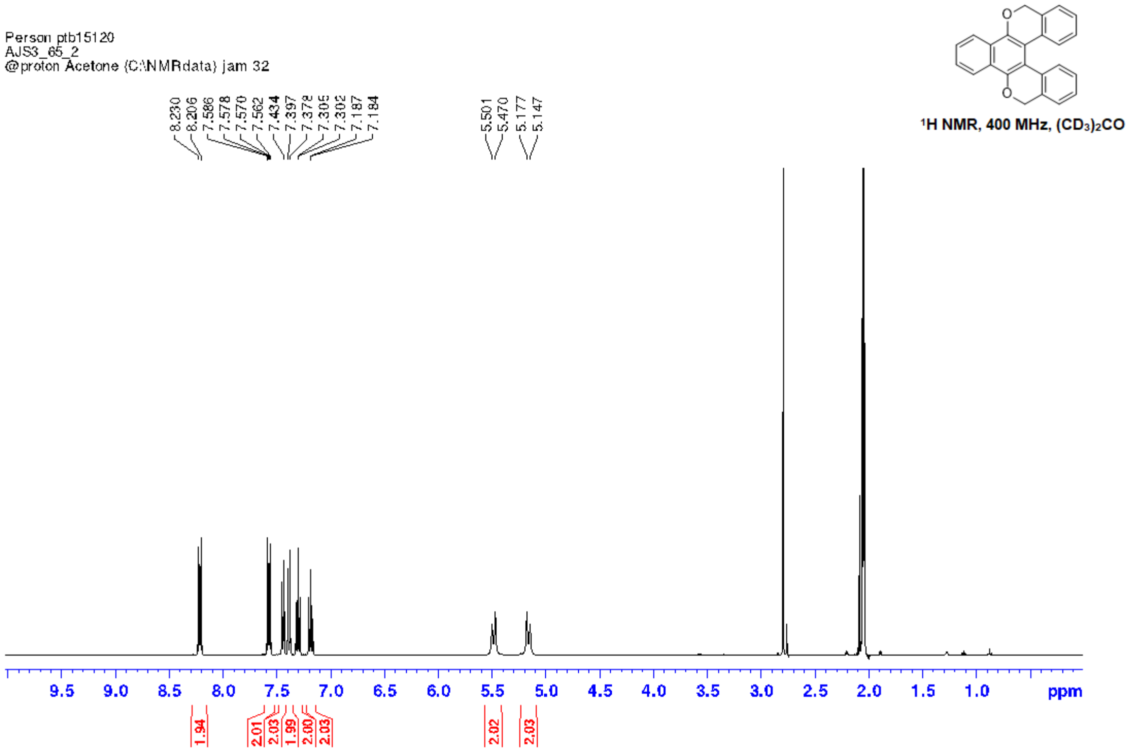
Person ptb15120
AJS3_65_2
@proton Acetone {C:\NMRdata} jam 32
8.230
8.206
7.586
7.578
7.570
7.562
7.434
7.397
7.378
7.309
7.302
7.187
7.184
5.501
5.470
5.177
5.147
O
O
¹H NMR, 400 MHz, (CD₃)₂CO
9.5 9.0 8.5 8.0 7.5 7.0 6.5 6.0 5.5 5.0 4.5 4.0 3.5 3.0 2.5 2.0 1.5 1.0 ppm
1.94
2.01
2.03
1.99
2.00
2.03
2.02
2.03

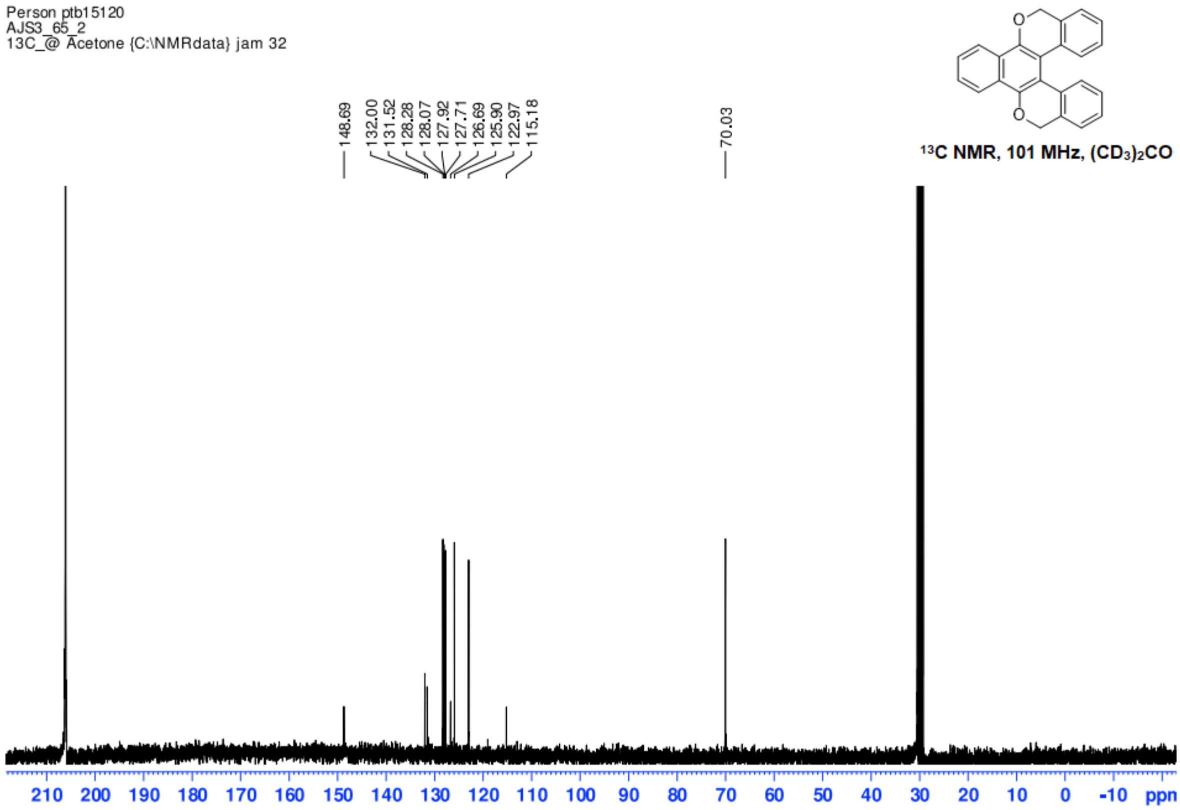
Person ptb15120
AJS3_65_2
13C_@ Acetone {C:\NMRdata} jam 32
148.69
132.00
131.52
128.28
128.07
127.92
127.71
126.69
125.90
122.97
115.18
70.03
O
O
¹³C NMR, 101 MHz, (CD₃)₂CO
210 200 190 180 170 160 150 140 130 120 110 100 90 80 70 60 50 40 30 20 10 0 -10 ppm

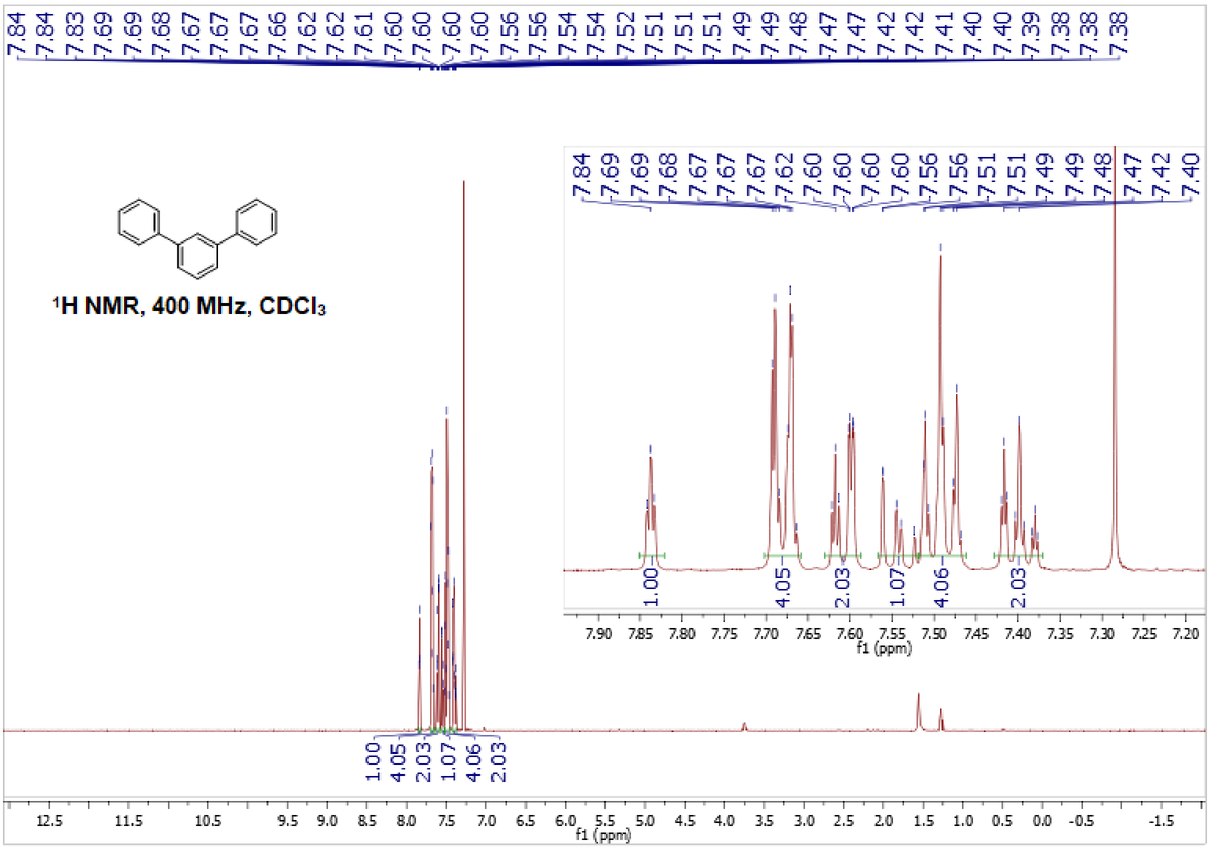
¹H NMR, 400 MHz, CDCl₃
7.84 7.84 7.83 7.69 7.69 7.68 7.67 7.67 7.67 7.66 7.62 7.62 7.61 7.60 7.60 7.60 7.60 7.56 7.56 7.54 7.54 7.52 7.51 7.51 7.51 7.49 7.49 7.48 7.47 7.47 7.42 7.42 7.41 7.40 7.40 7.39 7.38 7.38 7.38 7.38
1.00 4.05 2.03 1.07 4.06 2.03
f1 (ppm)

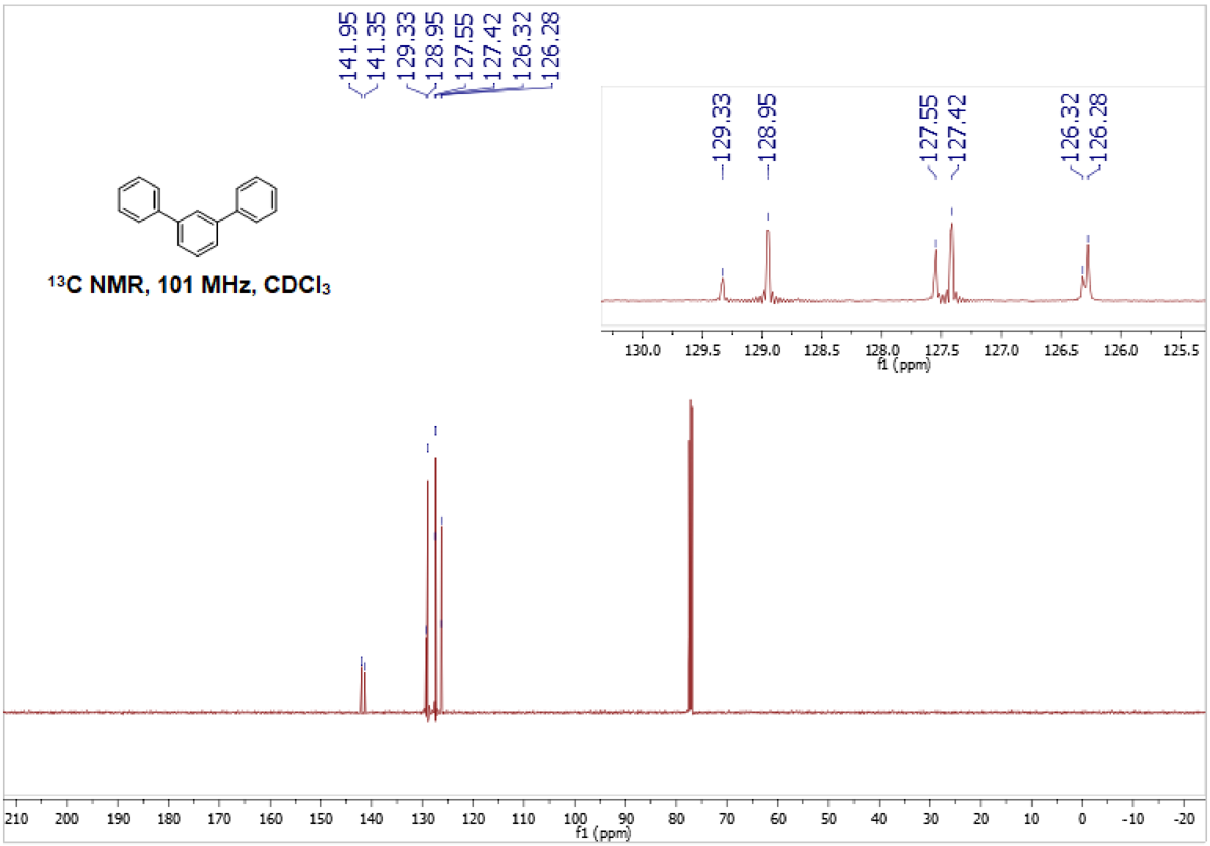
¹³C NMR, 101 MHz, CDCl₃
141.95 141.35 129.33 128.95 127.55 127.42 126.32 126.28
f1 (ppm)

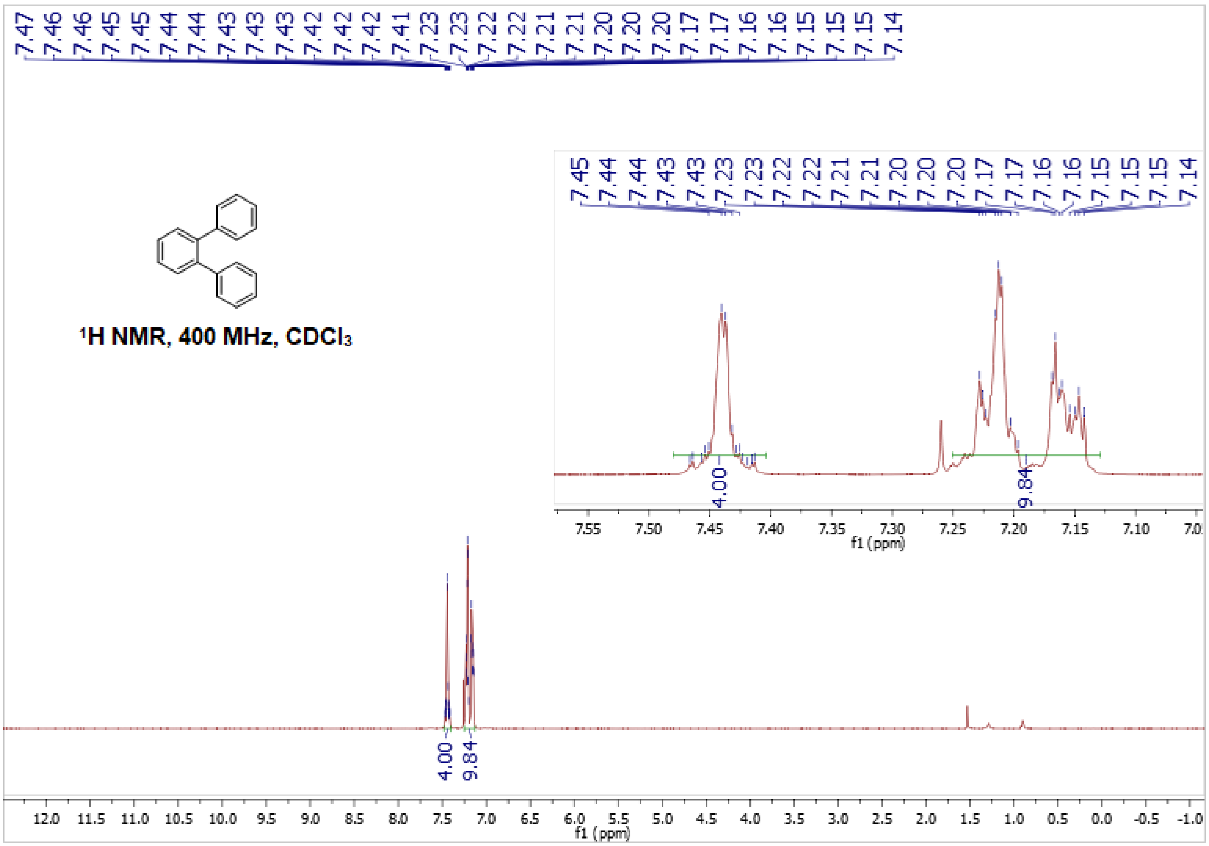
1H NMR, 400 MHz, CDCl3

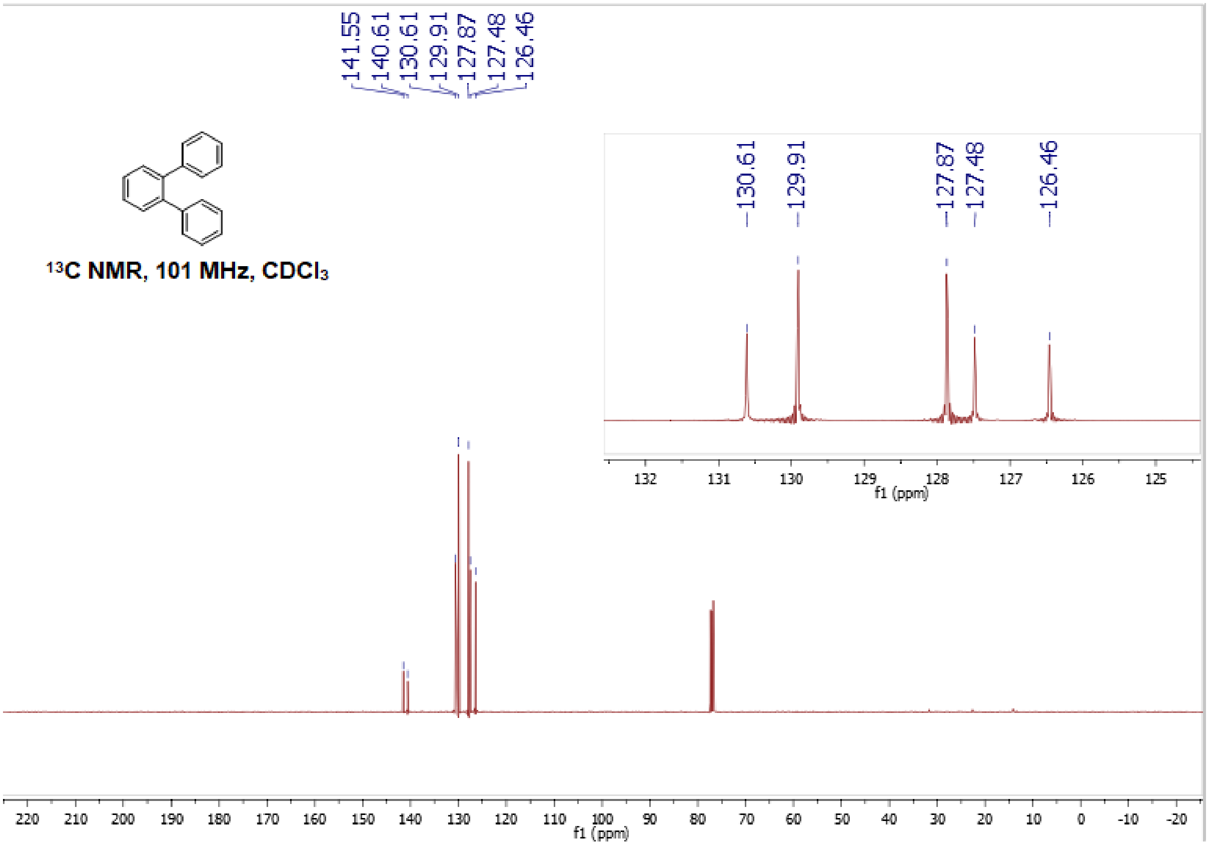
13C NMR, 101 MHz, CDCl3

nOe study

1D selective nOe NMR for compound 70: The structure of compound **70** was determined by 1D nOe spectroscopy. A significant nOe between the red **H** and the blue **H** is only possible in one structure, the *trans* isomer of **70**, shown in Figure S1. The "*cis*" isomer would not provide an nOe as the red and blue protons are well separated in space.

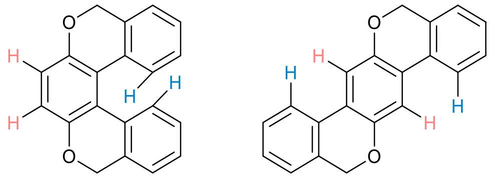

Figure S1 . Distinguishing between two possible isomers of **70** by nOe spectroscopy

Below, in Figure S2, the original spectrum of **70a** is shown in green. The signals, from left to right, correspond to 2, 4, 2, 2 protons. Selective irradiation of the upfield singlet (6.9 ppm, 2H), the signal corresponding to the protons of the central aromatic ring (Figure S1), gave no observable nOe (Figure S2, blue spectrum). It was expected that the *trans* compound, if present, would provide an nOe with the downfield doublet (7.59 ppm J = 8Hz, 2H), the signal for the blue protons.

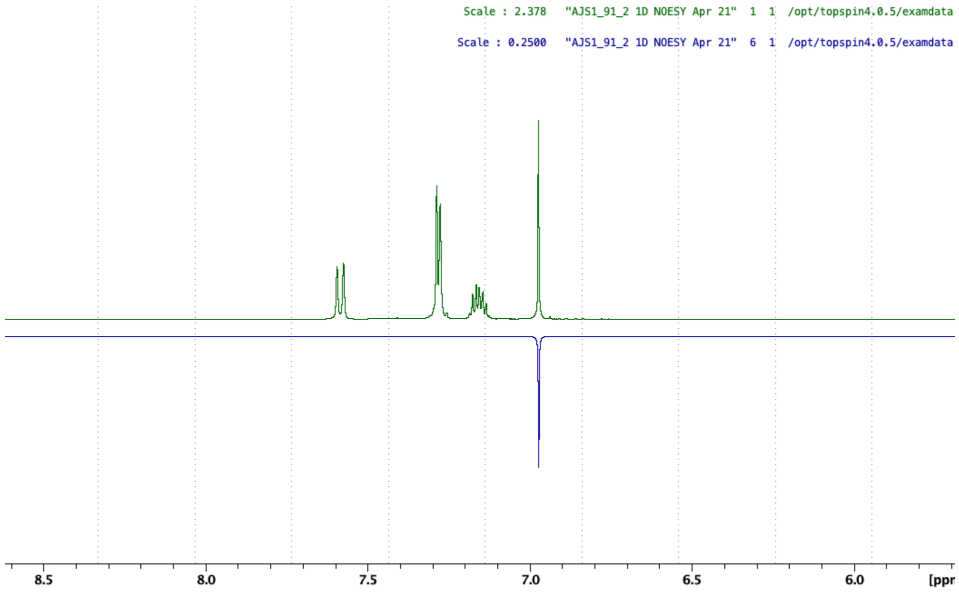

Figure S2. Selective irradiation of the indicated singlet of **70a** in a 1D nOe experiment. Blue nOe spectrum: irradiation at 6.97 ppm, mixing time 1 s, relaxation time 4 s

This is tangible evidence for the *cis* structure. To substantiate the absence (or presence) of the nOe, the experiment was performed in reverse. That is, irradiating the doublet representing the blue protons and observing the central ring singlet representing the red protons. Shown in Figure S3,

selective irradiation of this signal saw no observable nOe with the signal in question but gave an nOe of ~3% with the multiplet in the aromatic region. This 2H multiplet represents the protons *ortho* to the irradiated blue protons, likely to be much more impacted by the nOe. Close magnification shows a very small nOe (0.4%) with the protons *meta*- and *para*- to the irradiated signal. Additionally, there is a barely observable nOe (0.04%) between the irradiated signal and the upfield singlet due to the red protons. These atoms are ~6 Å apart and at the very upper limit of nOe. Any proposal of a *trans* isomer can be disregarded.

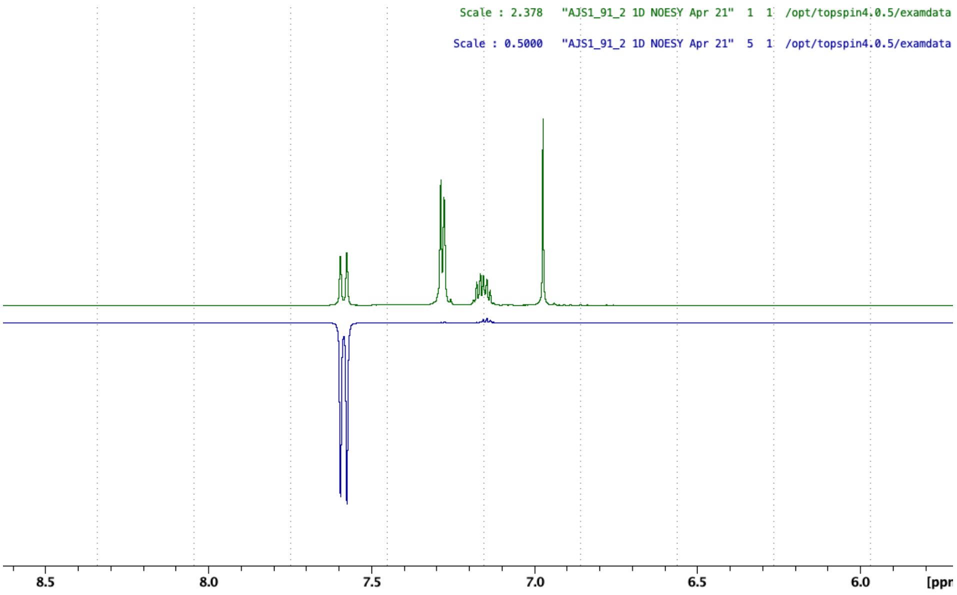

Figure S3. Selective irradiation of the downfield doublet in **70a**-*cis* nOe spectrum; irradiation at 7.58 ppm, mixing time 1 s, relaxation time 4 s.

GC-FID Calibration curves

To determine product yields by GC-FID analysis, calibration curves were constructed for each compound using authentic samples and an *n*-dodecane internal standard (IS). For each calibration curve, samples were made up with different molar ratios of each compound (X) and *n*-dodecane (IS). The samples were then analysed by GC-FID using the GC320 to get peak areas of X and IS. The calibration curves were constructed by plotting the molar ratio [mmol(X)/mmol(IS)] against the area ratio [area(X)/area(IS)]. The slope of the linear plot provides the Response Factor (RF) of the compound *vs.* dodecane. The yield of each compound can then be calculated from crude reaction mixtures, if a known mass of *n*-dodecane is added to the whole crude, using the following formula:

$$mmol(X) = \frac{area(X) * mmol(IS)}{area(IS) * RF(X)}$$

Due to a change in the instrument's detector during the course of the study, the response factors of each compound changed, and thus new calibration curves had to be constructed. The calibration curves for each method (Method 1 and Method 2) are provided below. In Method 2, calibrations for additional compounds were carried out due to an expansion of the study.

Method 1 *n*-dodecane retention time ≈ 10.7 min

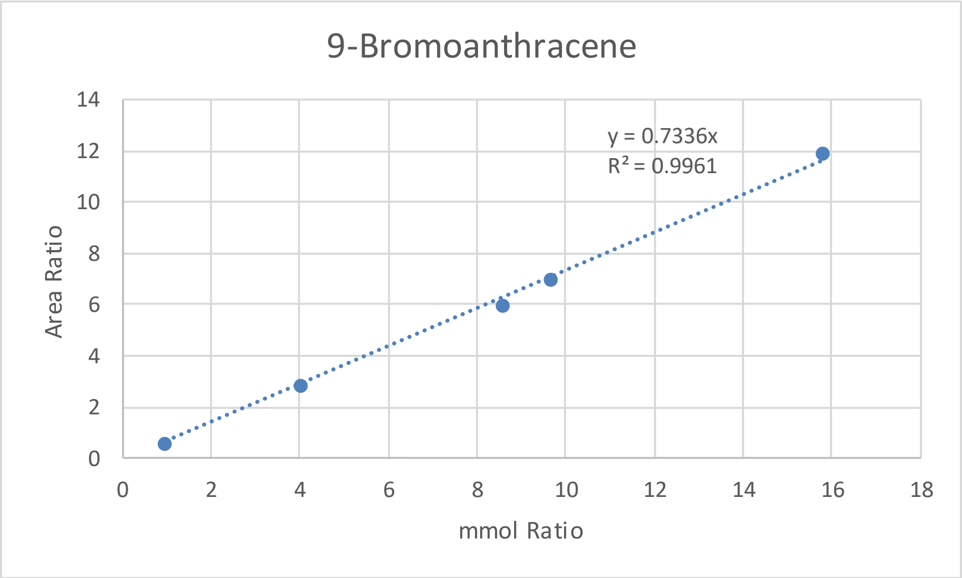

Retention time ≈ 16.2 min

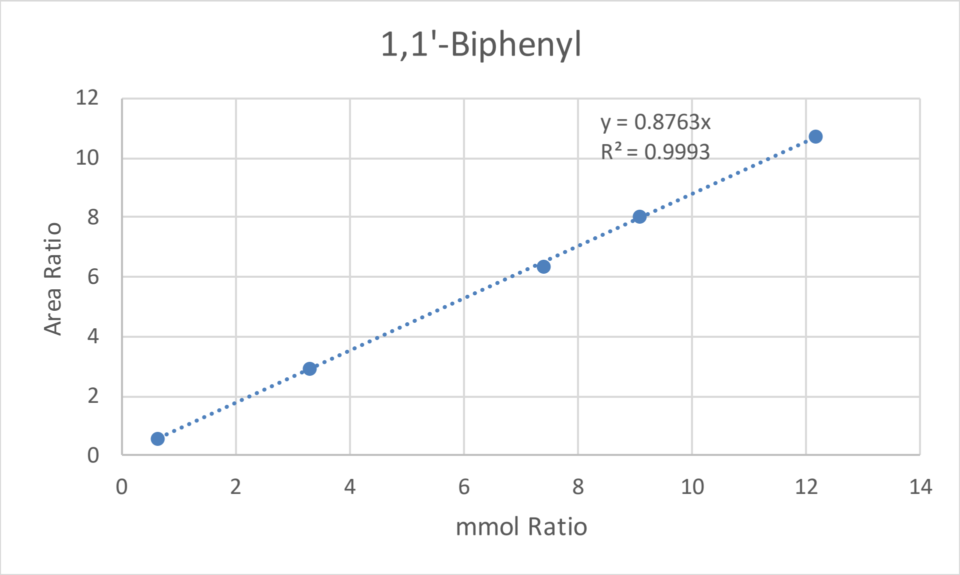

Retention time ≈ 12.1 min

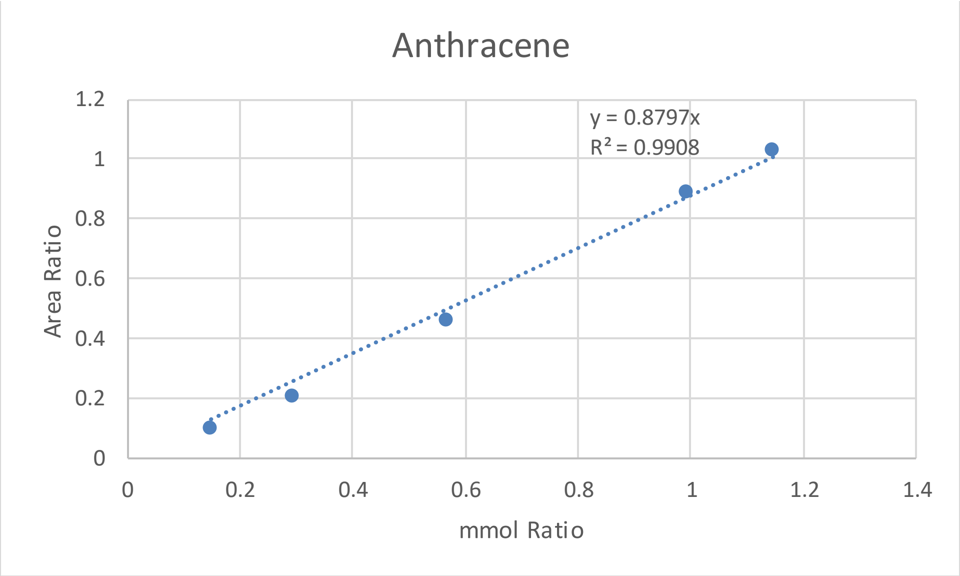

Retention time ≈ 14.5 min

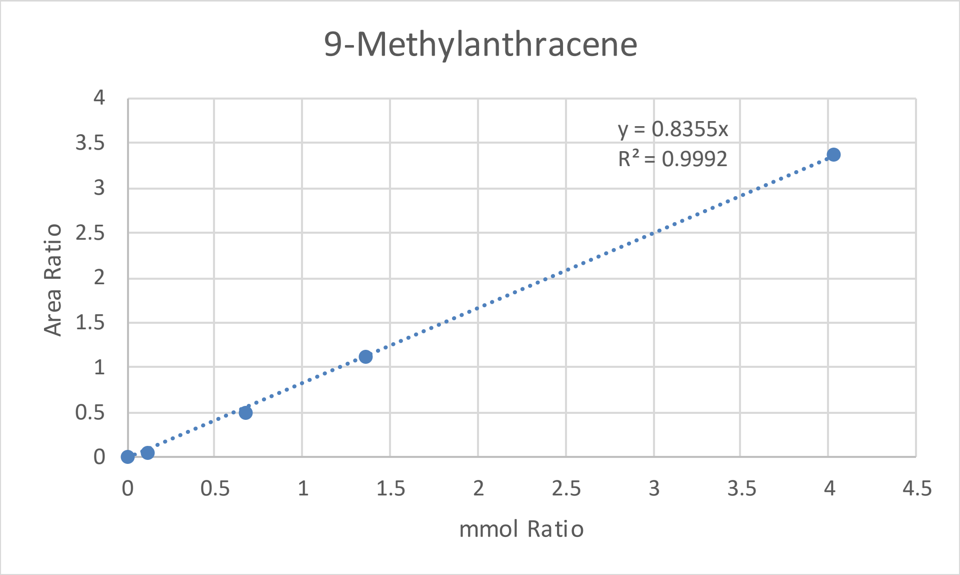

Retention time ≈ 15.4 min

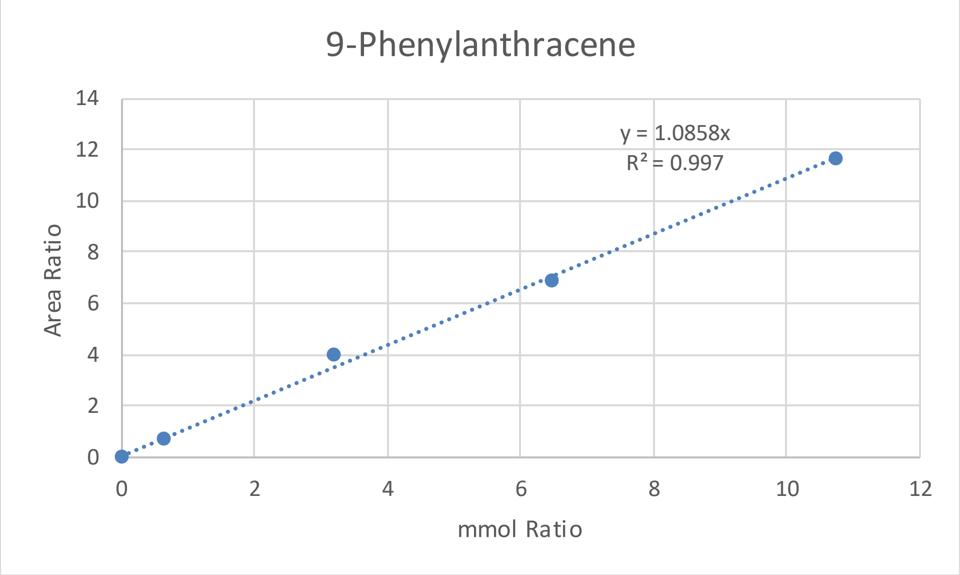

Retention time ≈ 17.4 min

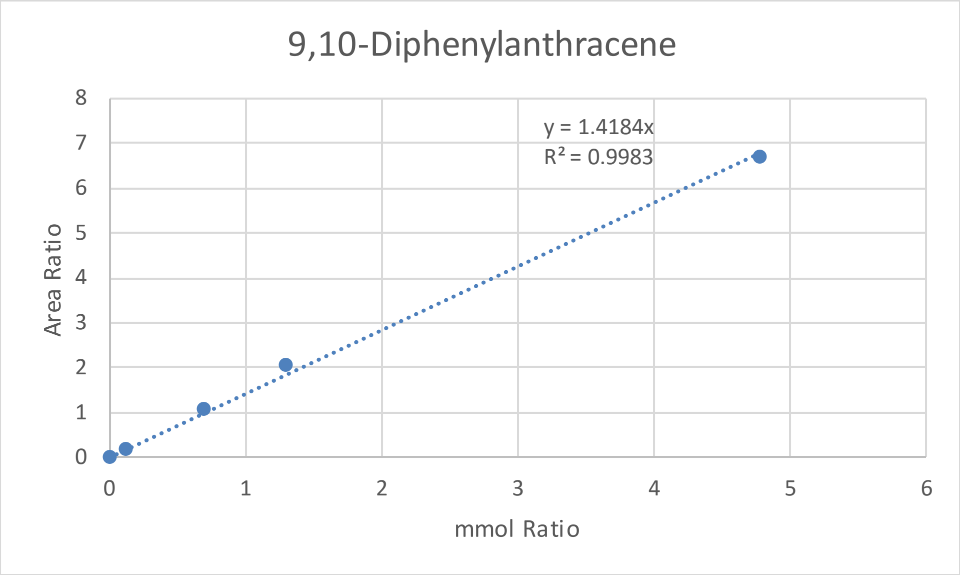

Retention time ≈ 19.7 min

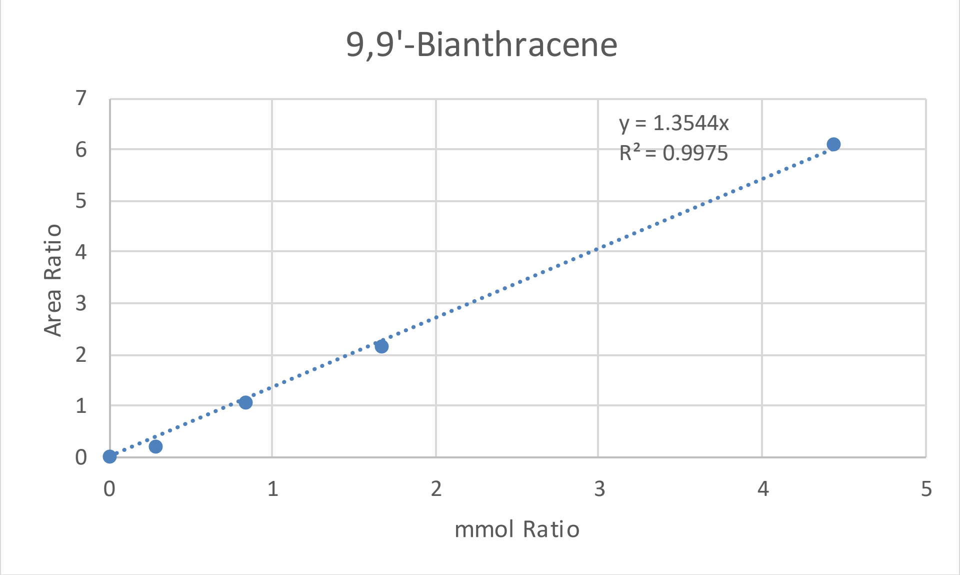

Retention time ≈ 21.3 min

Method 2 *n*-dodecane retention time ≈ 10.3 min

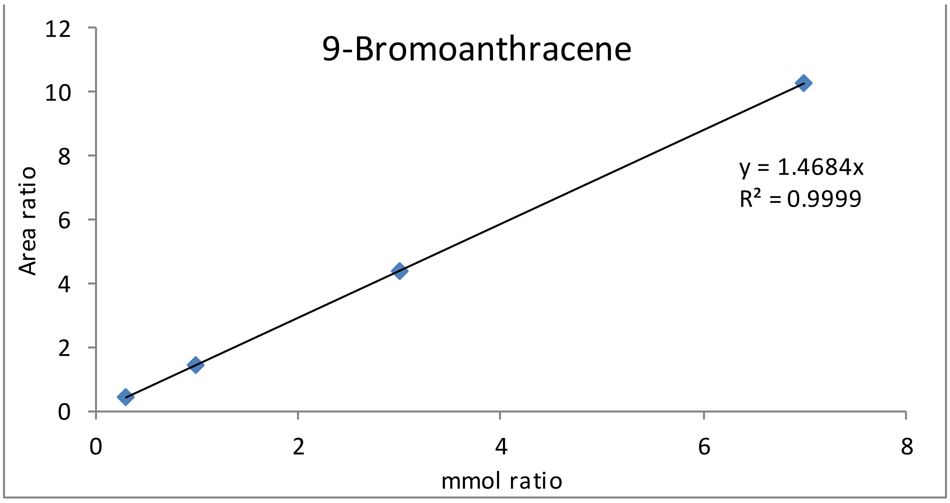

Retention time ≈ 15.8 min

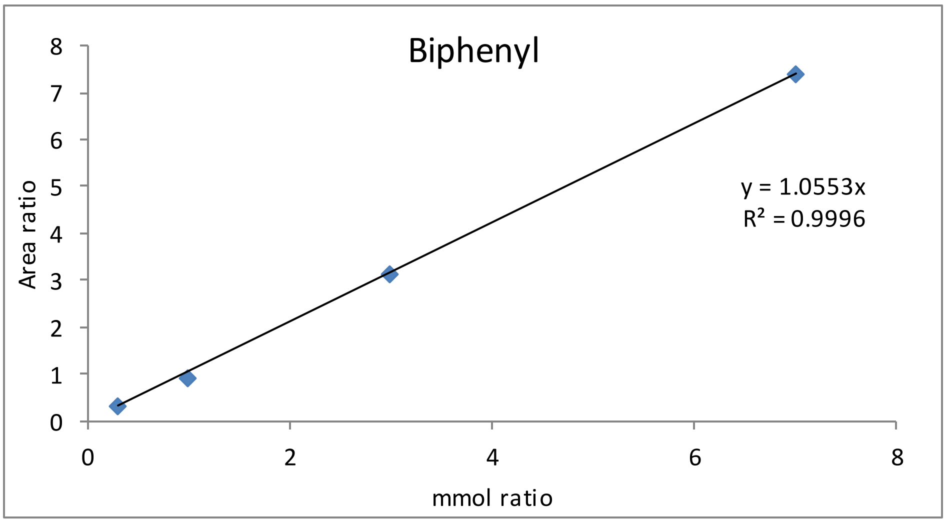

Retention time ≈ 11.7 min

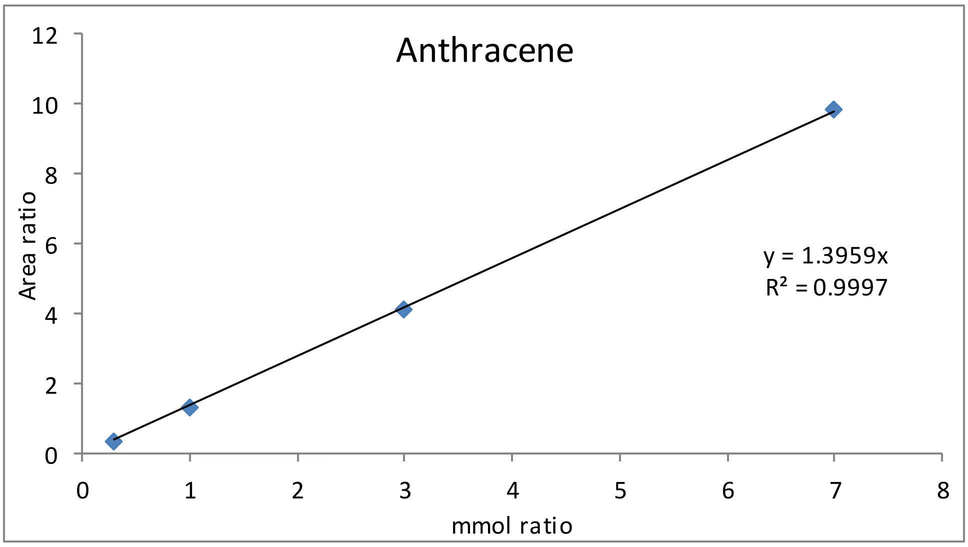

Retention time ≈ 14.2 min

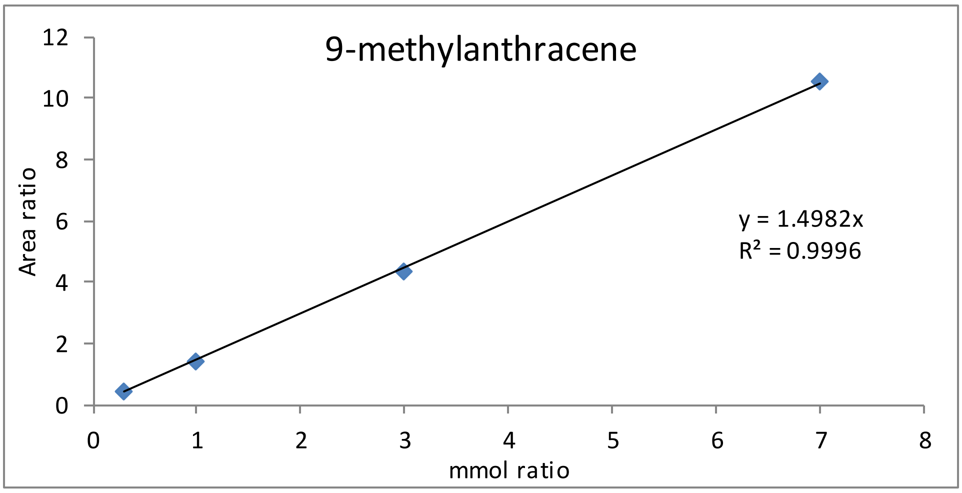

Retention time ≈ 15.0 min

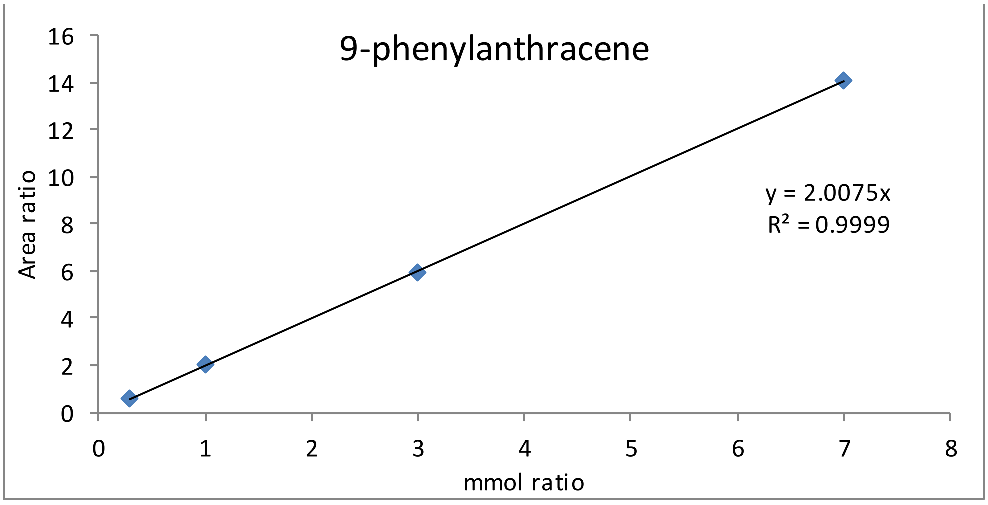

Retention time ≈ 17.0 min

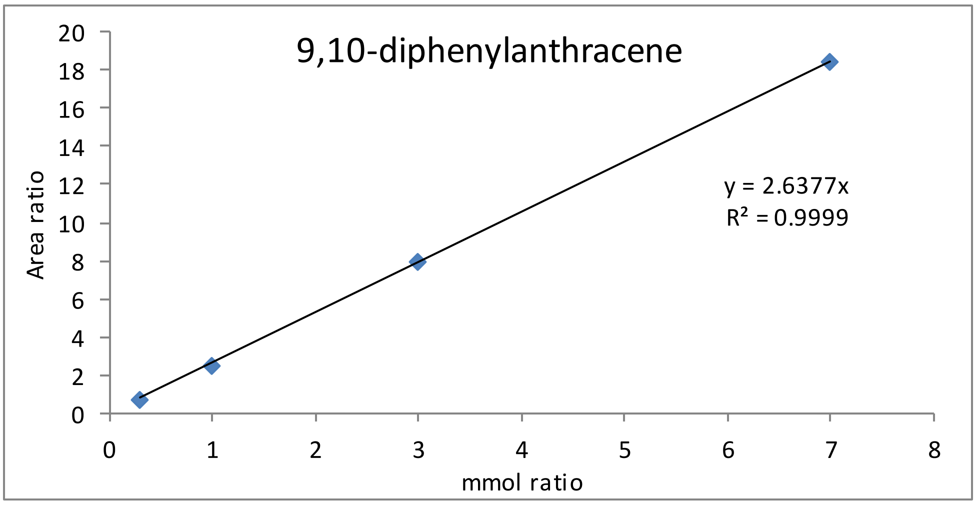

Retention time ≈ 19.2 min

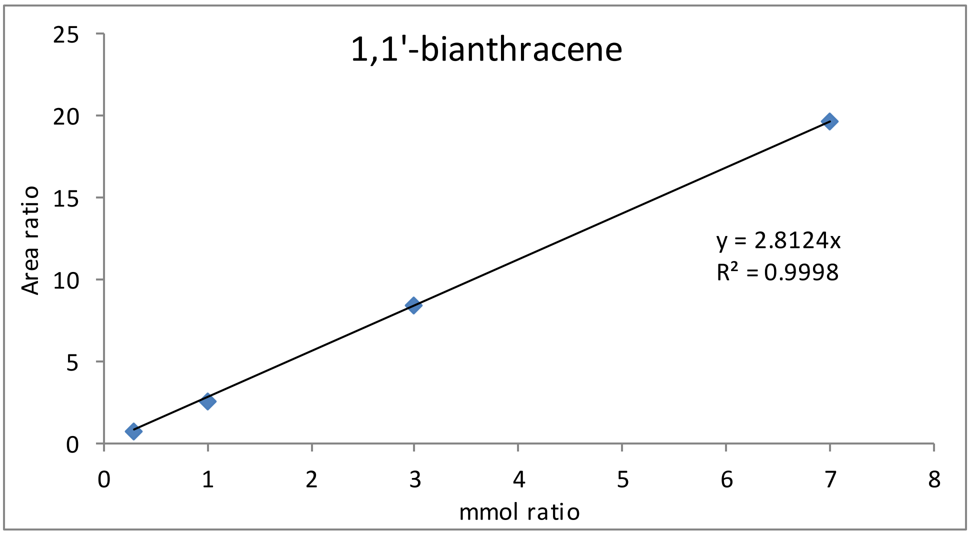

Retention time ≈ 20.7 min

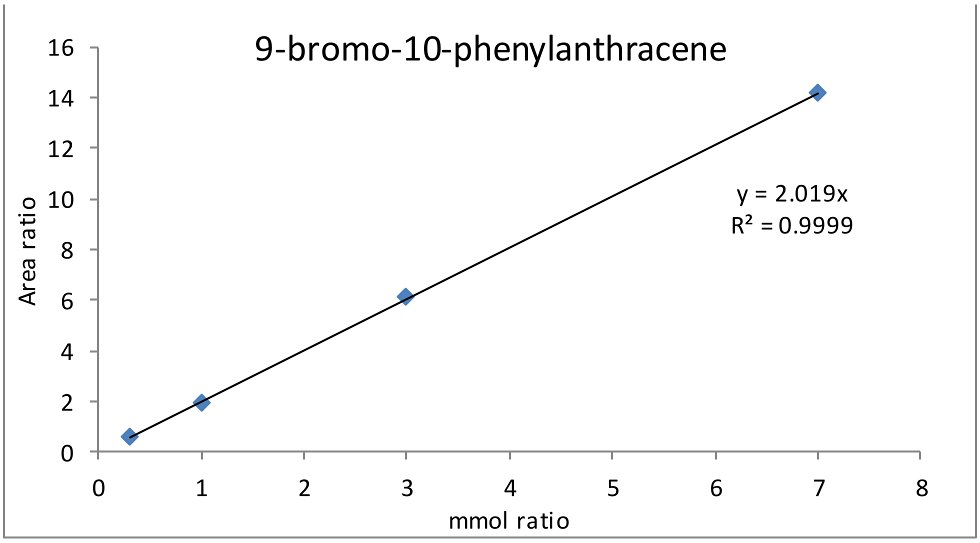

Retention time ≈ 18.3 min

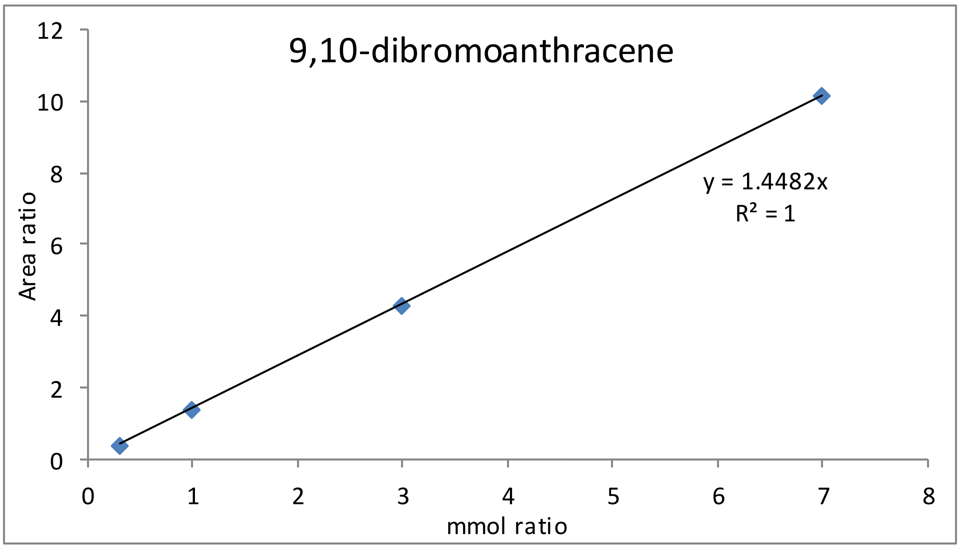

Retention time ≈ 17.2 min

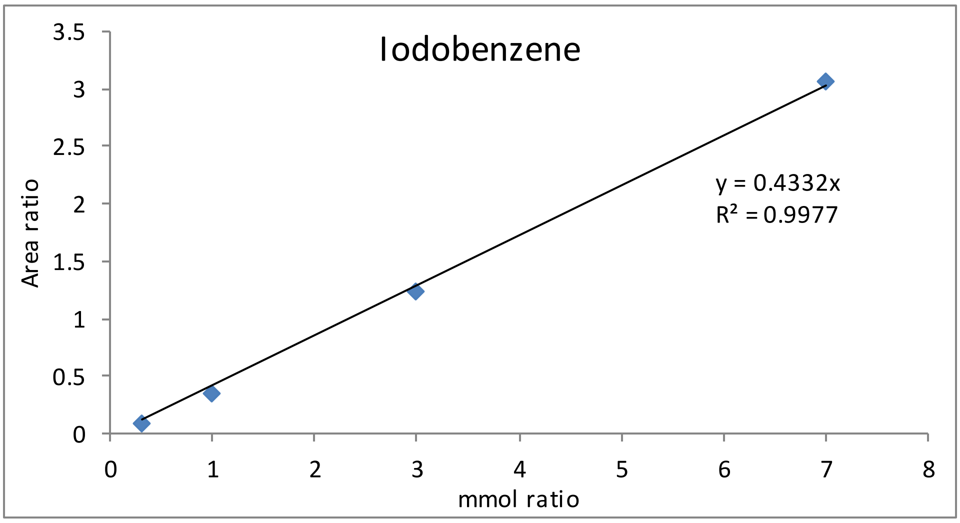

Retention time ≈ 8.9 min

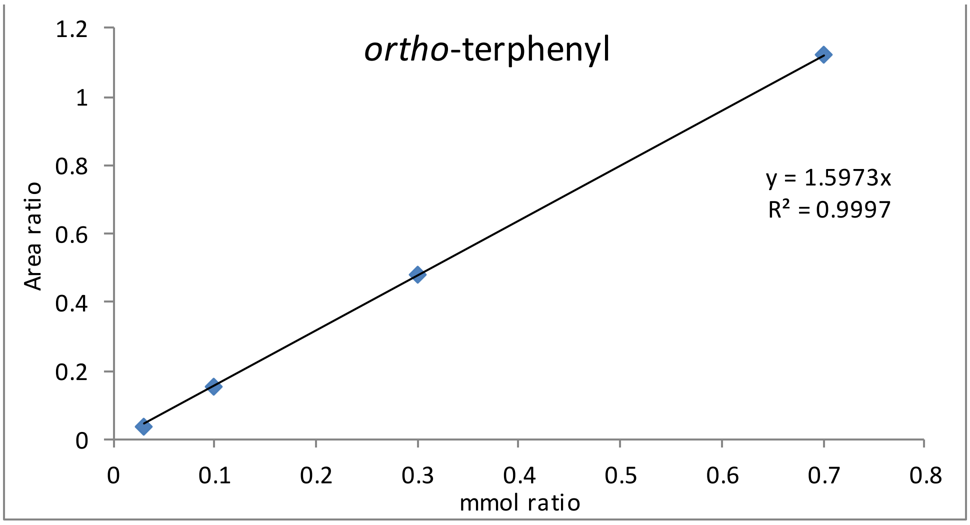

Retention time ≈ 14.6 min

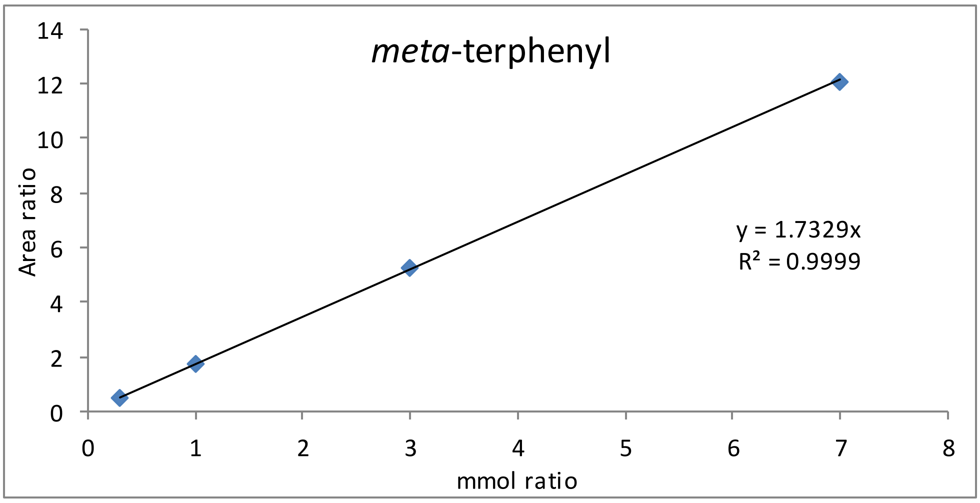

Retention time ≈ 15.8 min

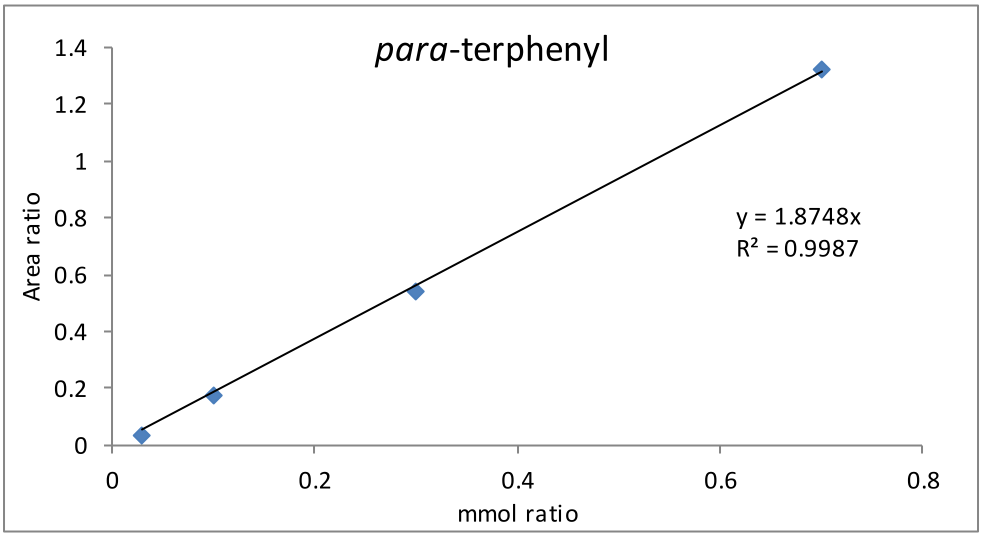

Retention time ≈ 16.0 min

X-ray crystallography data for 69

Crystallographic measurements were made with monochromatic Cu radiation (λ = 1.54184 Å) using a Rigaku Synergy-i diffractometer. Raw data processing utilised the program CrysalisPro.[71] The structure was solved using direct methods and was refined against F^2 to convergence using all unique reflections and the program Shelxl,[72] as implemented within WinGX.[73]

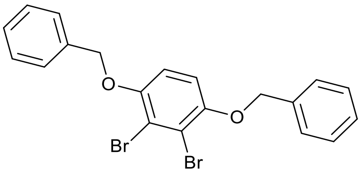

69

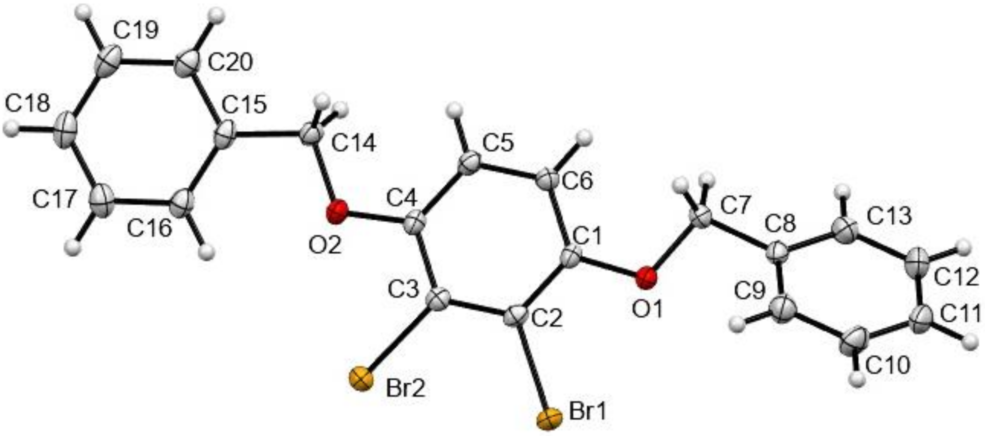

Table S1. Crystal data and structure refinement for compound **69** (Database ref number is CCDC 2211274)

Empirical formula	$C_{20}H_{16}Br_2O_2$	
Formula weight	448.15	
Temperature	100(2) K	
Wavelength	1.54184 Å	
Crystal system	Monoclinic	
Space group	P 21/c	
Unit cell dimensions	a = 17.8283(2) Å	α= 90°.
	b = 13.1051(2) Å	β= 96.5254(11)°.
	c = 7.43550(10) Å	γ = 90°.
Volume	1725.99(4) $Å^3$	
Z	4	
Density (calculated)	1.725 Mg/m^3	
Absorption coefficient	6.029 mm^{-1}	
F(000)	888	
Crystal size	0.33 x 0.06 x 0.06 mm3	
Theta range for data collection	2.494 to 71.418°.	
Index ranges	-21<=h<=21, -16<=k<=15, -6<=l<=9	
Reflections collected	14382	
Independent reflections	3340 [R(int) = 0.0336]	
Completeness to theta	= 70.000°	100.0 %
Absorption correction	Gaussian	
Max. and min. transmission	1.000 and 0.244	
Refinement method	Full-matrix least-squares on F2	
Data / restraints / parameters	3340 / 0 / 217	
Goodness-of-fit on F2	1.023	
Final R indices [I>2sigma(I)]	R1 = 0.0282, wR2 = 0.0743	
R indices (all data)	R1 = 0.0306, wR2 = 0.0756	
Extinction coefficient	n/a	
Largest diff. peak and hole	0.509 and -0.552 e.Å-3	

Table S2. Atomic coordinates (x 10^4) and equivalent isotropic displacement parameters (Å2x 103) for compound **69**. U(eq) is defined as one third of the trace of the orthogonalized Uij tensor.

	x	y	z	U(eq)
Br(1)	7729(1)	5830(1)	2311(1)	21(1)
Br(2)	5862(1)	5706(1)	1099(1)	22(1)
O(1)	8089(1)	6337(1)	6146(2)	20(1)
O(2)	5006(1)	6265(1)	4091(2)	22(1)
C(1)	7321(1)	6319(2)	5774(3)	17(1)
C(2)	7036(1)	6097(2)	3994(3)	17(1)
C(3)	6262(1)	6067(2)	3480(3)	18(1)
C(4)	5757(1)	6290(2)	4731(3)	18(1)
C(5)	6043(1)	6510(2)	6498(3)	18(1)
C(6)	6818(1)	6518(2)	7024(3)	18(1)
C(7)	8387(1)	6380(2)	8030(3)	20(1)
C(8)	9230(1)	6338(2)	8146(3)	20(1)
C(9)	9591(2)	5531(2)	7370(4)	27(1)
C(10)	10368(2)	5488(2)	7504(4)	31(1)
C(11)	10798(2)	6245(3)	8407(4)	34(1)

C(12)	10450(2)	7043(3)	9194(4)	36(1)
C(13)	9664(2)	7093(2)	9061(4)	28(1)
C(14)	4482(1)	6446(2)	5372(3)	20(1)
C(15)	3695(1)	6357(2)	4418(3)	20(1)
C(16)	3549(1)	6147(2)	2578(3)	23(1)
C(17)	2804(2)	6096(2)	1762(4)	27(1)
C(18)	2208(1)	6258(2)	2761(4)	27(1)
C(19)	2352(2)	6455(2)	4599(4)	28(1)
C(20)	3089(1)	6503(2)	5429(4)	24(1)

Table S3. Bond lengths [Å] and angles [°] for compound **69**.

Br(1)-C(2)	1.888(2)
Br(2)-C(3)	1.892(2)
O(1)-C(1)	1.367(3)
O(1)-C(7)	1.441(3)
O(2)-C(4)	1.370(3)
O(2)-C(14)	1.427(3)
C(1)-C(6)	1.387(3)
C(1)-C(2)	1.394(3)
C(2)-C(3)	1.389(3)
C(3)-C(4)	1.397(3)
C(4)-C(5)	1.384(3)
C(5)-C(6)	1.393(3)
C(7)-C(8)	1.497(3)
C(8)-C(13)	1.386(4)
C(8)-C(9)	1.397(4)
C(9)-C(10)	1.378(4)
C(10)-C(11)	1.381(4)
C(11)-C(12)	1.380(4)
C(12)-C(13)	1.395(4)
C(14)-C(15)	1.503(3)
C(15)-C(16)	1.391(4)
C(15)-C(20)	1.397(3)
C(16)-C(17)	1.396(4)
C(17)-C(18)	1.381(4)
C(18)-C(19)	1.386(4)
C(19)-C(20)	1.389(4)
C(1)-O(1)-C(7)	116.54(17)
C(4)-O(2)-C(14)	116.91(18)
O(1)-C(1)-C(6)	124.8(2)
O(1)-C(1)-C(2	116.4(2)
C(6)-C(1)-C(2)	118.8(2)
C(3)-C(2)-C(1)	120.7(2)
C(3)-C(2)-Br(1)	121.09(17)
C(1)-C(2)-Br(1)	118.23(17)
C(2)-C(3)-C(4)	120.4(2)
C(2)-C(3)-Br(2)	121.42(17)
C(4)-C(3)-Br(2)	118.18(18)

O(2)-C(4)-C(5)	125.0(2)
O(2)-C(4)-C(3)	116.3(2)
C(5)-C(4)-C(3)	118.7(2)
C(4)-C(5)-C(6)	120.9(2)
C(1)-C(6)-C(5)	120.4(2)
O(1)-C(7)-C(8)	108.11(18)
C(13)-C(8)-C(9)	119.0(2)
C(13)-C(8)-C(7)	120.2(2)
C(9)-C(8)-C(7)	120.7(2)
C(10)-C(9)-C(8)	120.5(3)
C(9)-C(10)-C(11)	120.3(3)
C(12)-C(11)-C(10)	119.9(3)
C(11)-C(12)-C(13)	120.1(3)
C(8)-C(13)-C(12)	120.1(3)
O(2)-C(14)-C(15)	108.58(19)
C(16)-C(15)-C(20)	119.1(2)
C(16)-C(15)-C(14)	122.7(2)
C(20)-C(15)-C(14)	118.2(2)
C(15)-C(16)-C(17)	120.0(2)
C(18)-C(17)-C(16)	120.7(3)
C(17)-C(18)-C(19)	119.5(2)
C(18)-C(19)-C(20)	120.4(2)
C(19)-C(20)-C(15)	120.4(2)

Table S4. Anisotropic displacement parameters (Å2x 103) for **69**. The anisotropic displacement factor exponent takes the form: $-2\pi^2[h^2a^{*2}U^{11} + ... + 2 h k a^* b^* U^{12}]$

	U11	U22	U33	U23	U13	U12
Br(1)	17(1)	29(1)	19(1)	-3(1)	6(1)	0(1)
Br(2)	19(1)	28(1)	17(1)	-2(1)	1(1)	-2(1)
O(1)	14(1)	30(1)	16(1)	0(1)	2(1)	-1(1)
O(2)	13(1)	32(1)	20(1)	-2(1)	2(1)	1(1)
C(1)	12(1)	19(1)	20(1)	2(1)	2(1)	-1(1)
C(2)	17(1)	14(1)	20(1)	0(1)	5(1)	1(1)
C(3)	18(1)	17(1)	18(1)	1(1)	2(1)	0(1)
C(4)	13(1)	18(1)	21(1)	2(1)	2(1)	-1(1)
C(5)	16(1)	18(1)	22(1)	-2(1)	5(1)	1(1)
C(6)	16(1)	19(1)	19(1)	-3(1)	3(1)	0(1)
C(7)	17(1)	26(1)	16(1)	-1(1)	0(1)	0(1)
C(8)	15(1)	26(1)	17(1)	3(1)	1(1)	-1(1)
C(9)	23(1)	28(1)	31(1)	-5(1)	3(1)	-1(1)
C(10)	21(1)	41(2)	34(1)	-1(1)	7(1)	9(1)
C(11)	15(1)	62(2)	26(1)	0(1)	1(1)	0(1)
C(12)	21(1)	53(2)	33(1)	-10(1)	1(1)	-11(1)
C(13)	24(1)	33(1)	27(1)	-7(1)	4(1)	-4(1)
C(14)	15(1)	22(1)	23(1)	-1(1)	4(1)	1(1)
C(15)	15(1)	15(1)	30(1)	1(1)	1(1)	1(1)
C(16)	19(1)	20(1)	29(1)	-1(1)	2(1)	-2(1)
C(17)	24(1)	24(1)	32(1)	-3(1)	-3(1)	-1(1)
C(18)	18(1)	22(1)	41(2)	1(1)	-3(1)	0(1)

C(19)	18(1)	26(1)	41(2)	2(1)	7(1)	2(1)
C(20)	21(1)	23(1)	29(1)	0(1)	5(1)	3(1)

Table S5. Hydrogen coordinates (x 104) and isotropic displacement parameters (Å2x 103) for compound **69**

	x	y	z	U(eq)
H(5)	5705	6659	7364	22
H(6)	7004	6659	8247	22
H(7A)	8195	5797	8691	24
H(7B)	8226	7020	8582	24
H(9)	9299	5008	6745	33
H(10)	10608	4935	6972	38
H(11)	11333	6216	8487	41
H(12)	10746	7560	9827	43
H(13)	9426	7645	9598	34
H(14A)	4561	7136	5901	24
H(14B)	4558	5940	6365	24
H(16)	3955	6038	1876	27
H(17)	2707	5949	506	33
H(18)	1703	6234	2193	33
H(19)	1943	6558	5296	33
H(20)	3183	6635	6691	29

DFT Calculations

Methodology

All DFT calculations were carried out using the Gaussian16 software package (*74*). Geometry optimisation was carried out at the M06-2X/6-311++G(d,p) (75) level of theory with solvation (benzene) applied using the implicit CPCM (76-80) model. For structures that contain iodine, Stuttgart/Dresden ECPs (81,82) were used (with the SDD keyword). The nature of each stationary point was confirmed using frequency calculations: minima have zero imaginary frequencies and transition states have exactly one. IRC calculations were used to confirm that each transition state linked the anticipated minima. Energies were refined with single point calculations at the M06-2X-D3/Def2-QZVPD/CPCM(benzene) level of theory (83-85). Free energies are obtained from the sum of: the electronic energy with the quadruple-ζ basis set, the correction to free energy obtained with the triple-ζ basis set, and 1.89 kcal/mol (to account for a 1 mol/L standard state).(86)
Open shell singlet energies were corrected for triplet contamination using Yamaguchi's approach, (87-91) i.e.

$E_{corrected,\ oss} = (2 \times E_{oss}) - (E_{triplet} \times \langle S^2 \rangle_{oss})) / (2 - \langle S^2 \rangle_{oss})$

Data Availability

All data are available via the ioChem-BD (92) repository hosted at the Barcelona Supercomputing Centre."

Coordinates from DFT Calculations

XYZ coordinates for all structures are provided as a separate supporting information file. Alternatively, these can be obtained from the ioChem-BD repository.

Energies from DFT Calculations

The energies from DFT calculations are recorded in Table S6.

Energetics of SET from [KO*t*Bu]$_4$ to aryl halides

The electron transfer process directly from the tetramer to iodobenzene or bromoanthracene can be ruled out entirely based on ΔG° alone.

Note that the radical anion of iodobenzene spontaneously ejects iodide to give phenyl radical and iodide.

$$\text{PhI} + [\text{KO}t\text{Bu}]_4 \longrightarrow \text{Ph}^{\bullet} + \text{I}^{\ominus} + [\text{KO}t\text{Bu}]_4^{\oplus\bullet} \qquad \Delta G = +\,94.6 \text{ kcal/mol}$$

$$\text{9-BrAn} + [\text{KO}t\text{Bu}]_4 \longrightarrow \text{9-BrAn}^{\ominus\bullet} + [\text{KO}t\text{Bu}]_4^{\oplus\bullet} \qquad \Delta G = +95.2 \text{ kcal/mol}$$

9-BrAn = 9-Bromoanthracene

Comparison of the reactivity of *p*-Benzyne *versus* phenyl radical in reactions with benzene *and* iodobenzene

The *para*-benzyne was modelled here for simplicity. *p*-Benzyne and phenyl radical are both capable of reacting with benzene and iodobenzene and the barriers are all rather similar.

The reactions of each radical species with benzene have a barrier of *ca.* 20 kcal/mol, while the reactions with iodobenzene have a barrier of *ca.* 23 kcal/mol. However, the *ipso*-substitution of iodobenzene is substantially more thermodynamically favourable in each case (ΔG = *ca.* –40 kcal/mol). Under the reaction conditions the addition of the radical to benzene is reversible, as the barrier to the C–C bond cleavage is *ca.* 25 kcal/mol.

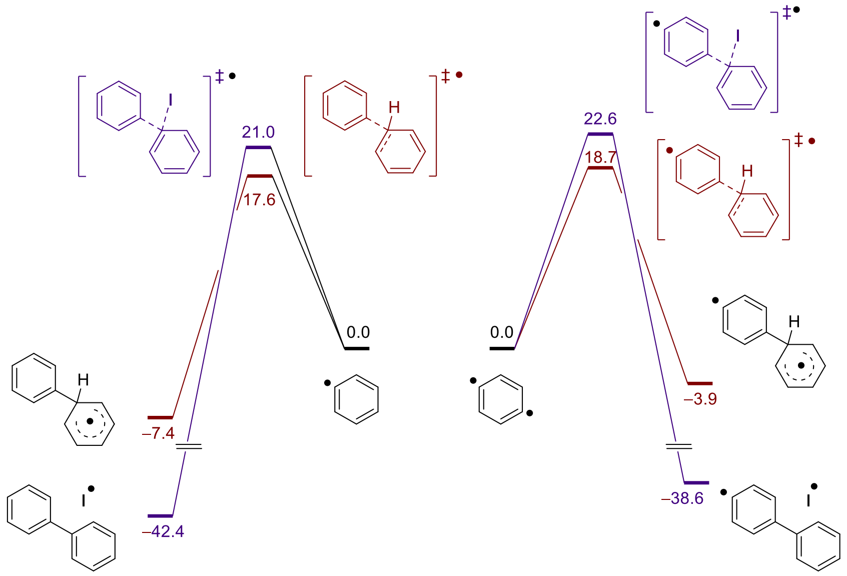

Role of KO*t*Bu as a hydrogen atom source and as a methyl radical source

(a) H-atom abstraction from KO*t*Bu by anthracenyl radical

Anthracenyl radical can abstract a hydrogen from potassium *tert*-butoxide tetramer readily ($\Delta G^{\ddagger}$ = 18.0 kcal/mol) and exergonically (ΔG = –11.8 kcal/mol). The radical thus formed can release methyl radical ($\Delta G^{\ddagger}$ = 21.3 kcal/mol, ΔG = –7.2 kcal/mol).

The methyl radical might then either:

- Abstract a hydrogen from the methyl of the potassium enolate, with quite a low barrier ($\Delta G^{\ddagger}$ = 14.5 kcal/mol, ΔG = –12.2 kcal/mol)
- Abstract a hydrogen from anthracene but this has a relatively high barrier and is endergonic ($\Delta G^{\ddagger}$ = 29.8 kcal/mol, ΔG = 8.3 kcal/mol)

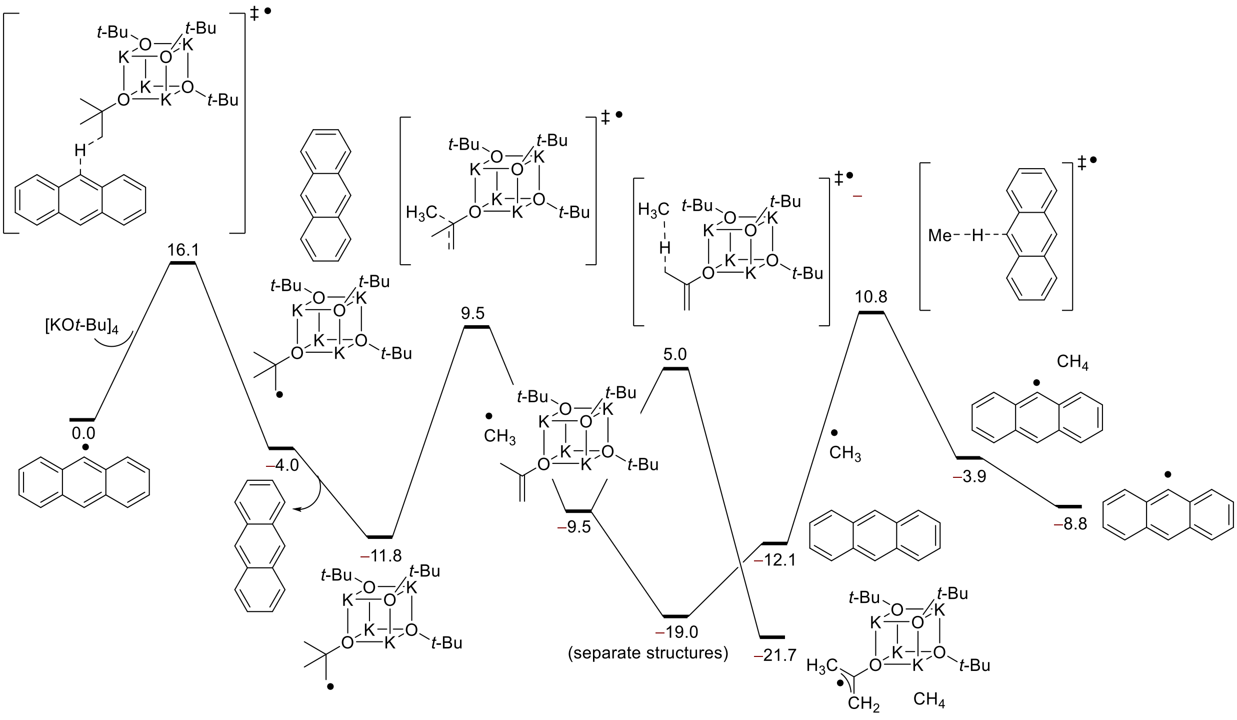

(b) H-atom abstraction from KO*t*Bu by phenyl radical

A similar situation emerges with phenyl radical/benzene.

- The initial C-H abstraction is slightly more challenging, by 1 kcal/mol, and is less exergonic, by 0.9 kcal/mol.
- Methyl radical more readily abstracts a hydrogen atom from benzene than from anthracene (by 2.1 kcal/mol).

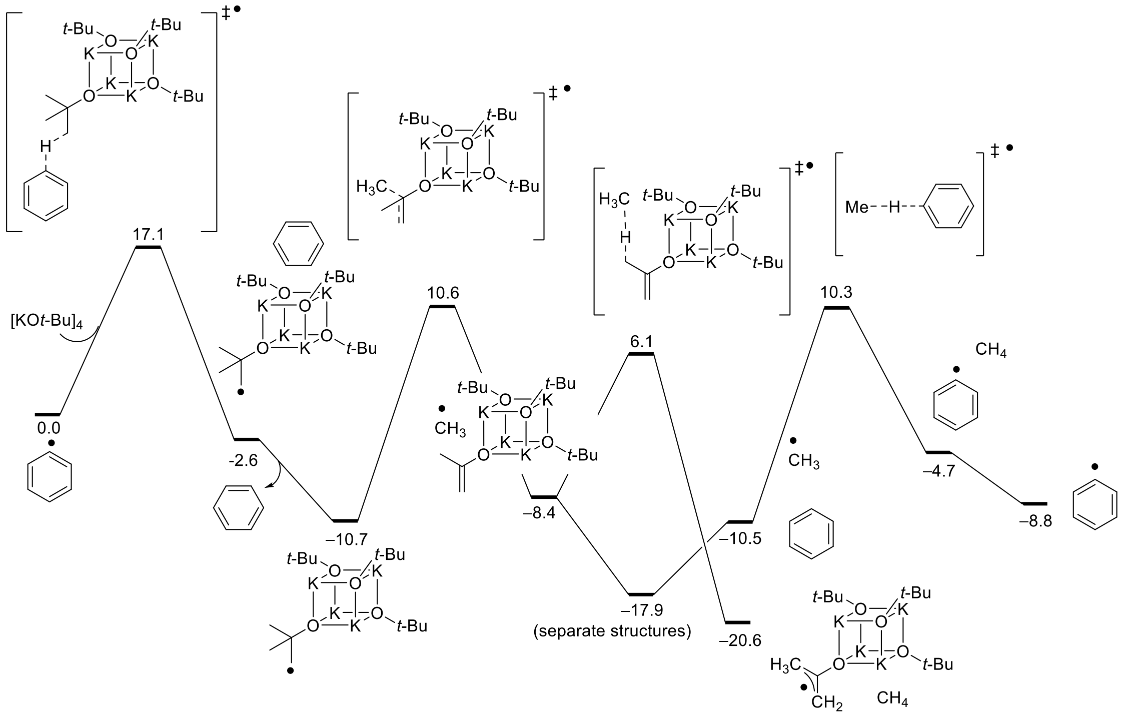

Benzene and Anthracene as substrate for C-C bond formation *vs* C-H abstraction by methyl radical.

Methyl radical can either abstract a C-H from an arene or attack the arene to form a (partially) dearomatized species. The table below records the barriers ($\Delta G^{\ddagger}$) and free energy changes (ΔG) for each of these processes for benzene and for anthracene. In the case of anthracene, the 1-, 2-, and 9-positions are considered (which are of course equivalent to the 4-, 3-, and 10-positions by symmetry. All energies below are free energies in kcal/mol with respect to methyl radical plus the arene.

For benzene, the two processes are quite close in energy (i.e. close to the limits of accuracy of DFT); radical attack is less endergonic.
For anthracene, there is a clear-cut preference for radical attack over C-H abstraction ($\Delta\Delta G^{\ddagger}$ = 10.0 kcal/mol), which is exergonic.

**Arene**	**Position**	**C-H Abstraction**		**Radical Attack**	
		**$\Delta G^{\ddagger}$**	**ΔG**	**$\Delta G^{\ddagger}$**	**ΔG**
Benzene	n/a	**26.3**	11.3	24.4	3.7
Anthracene	1	27.0	16.6	20.5	-8.7
	2	26.6	14.0	21.9	-4.7
	9	27.9	13.2	17.9	-16.2

Phenyl radical addition *vs.* C-H abstraction with benzene, *vs.* C-H abstraction from [KO*t*Bu]$_4$

- Phenyl radical addition to benzene has a barrier of 17.6 kcal/mol, as noted on page S141.
- Phenyl radical H abstraction from benzene – an identity reaction – has a barrier of 20.0 kcal/mol.
- Phenyl radical H abstraction from [KO*t*Bu]$_4$ has a barrier of 17.1 kcal/mol, as noted on page S143

The abstraction of H *vs* D from benzene shows a primary KIE with $\Delta\Delta G^{\ddagger}$ = 1.1 kcal/mol.

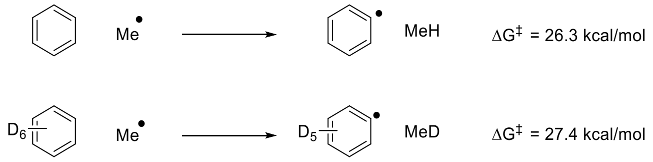

The abstraction of H *vs* D from [KO*t*Bu]$_4$ shows a primary KIE with $\Delta\Delta G^{\ddagger}$ = 0.8 kcal/mol.

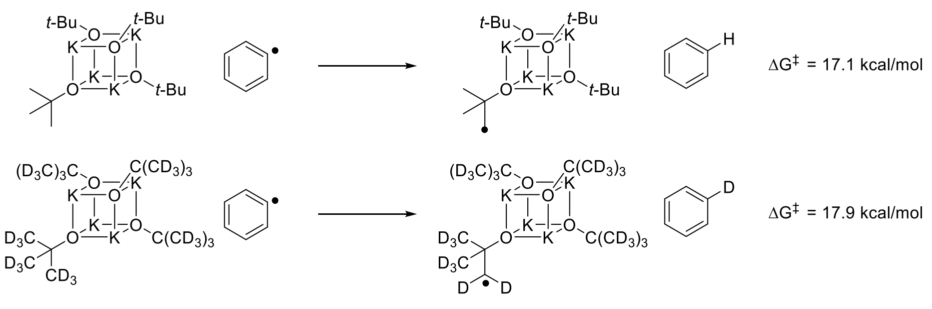

Table S6. Details of each structure characterised using DFT calculations										
Structure	M06-2X/6-311++G(d,p)/CPCM(benzene)					M06-2X-D3/Def2-QZVPD/CPCM(benzene)				
			Hartrees				Hartrees		kcal/mol	
	ν (cm^{-1})	<S**2>	E	Gcorr	G	<S**2>	E	G	G	G'
o-benzyne		0.0000	-230.868745	0.036236	-230.832509	0.0000	-230.915594	-230.879358	-144879.0	-144877.1
benzene		0.0000	-232.200301	0.061492	-232.138809	0.0000	-232.247427	-232.185935	-145698.9	-145697.0
iodobenzene		0.0000	-242.966926	0.044701	-242.922225	0.0000	-529.279750	-529.235049	-332100.0	-332098.1
p-benzyne		0.9966	-230.830188	0.035420	-230.794768	0.9969	-230.876132			
						2.0109	-230.872332			
							-230.879908	-230.844488	-144857.1	-144855.2
TS p-benzyne addition to benzene	420i	1.0231	-463.023455	0.120730	-462.902725	1.0244	-463.115517	-462.994787	-290533.6	

						2.0336	-463.112809	-463.112809	-290607.7	
							-463.118361	-462.997631	-290535.4	-290533.5
C6H4-C6H6 diradical		1.0448	-463.066609	0.124490	-462.942119	1.0448	-463.158064	-463.033574	-290558.0	
						2.0442	-463.158037	-463.158037	-290636.1	
							-463.158094	-463.033604	-290558.0	-290556.1
TS p-benzyne addition to iodobenzene	510i	1.0312	-473.787578	0.105415	-473.682163	1.0332	-760.143545	-760.038130	-476931.1	
						2.0368	-760.141282	-760.141282	-476995.9	
							-760.145963	-760.040548	-476932.6	-476930.8
C6H4-Ph radical + I radical		1.0136	-473.883001	0.108453	-473.774548	1.0190	-760.246547	-760.138094	-476993.9	
						2.0203	-760.246605	-760.246605	-477061.9	

							-760.246487	-760.138034	-476993.8	-476991.9
phenyl radical		0.7620	-231.514556	0.047625	-231.466931	0.7620	-231.561096	-231.513471	-145276.9	-145275.0
TS phenyl radical addition to benzene	427i	0.7806	-463.708051	0.132441	-463.575610	0.7814	-463.800805	-463.668364	-290956.3	-290954.4
Ph-C6H6 radical		0.7823	-463.752733	0.136607	-463.616126	0.7823	-463.844846	-463.708239	-290981.3	-290979.4
TS phenyl radical addition to iodobenzene	513i	0.7828	-474.472740	0.117422	-474.355318	0.7842	-760.829411	-760.711989	-477354.0	-477352.1
Ph-Ph + I radical		0.7521	-474.569398	0.120395	-474.449003	0.7572	-760.933403	-760.813008	-477417.4	-477415.5
KOtBu tetramer		0.0000	-3332.133524	0.378633	-3331.754891	0.0000	-3332.355436	-3331.976803	-2090847.0	-2090845.1
9-anthracenyl radical		0.7749	-538.741915	0.129408	-538.612507	0.7748	-538.849543	-538.720135	-338052.0	-338050.1

anthracenyl radical C-<br>H abstraction from<br>KOtBu	141<br>2i	0.768<br>7	-<br>3870.8672<br>90	0.5288<br>35	-<br>3870.3384<br>55	0.769<br>5	-<br>3871.1970<br>72	-<br>3870.6682<br>37	-<br>2428881<br>.0	-<br>2428879<br>.1
anthracene +<br>(KOtBu3)(KOC(Me)2C<br>H2•)		0.756<br>1	-<br>3870.9029<br>76	0.5323<br>94	-<br>3870.3705<br>82	0.756<br>6	-<br>3871.2327<br>68	-<br>3870.7003<br>74	-<br>2428901<br>.2	-<br>2428899<br>.3
(KOtBu3)(KOC(Me)2C<br>H2•)		0.756<br>0	-<br>3331.4644<br>30	0.3639<br>93	-<br>3331.1004<br>37	0.756<br>2	-<br>3331.6854<br>45	-<br>3331.3214<br>52	-<br>2090435<br>.8	-<br>2090433<br>.9
anthracene		0.000<br>0	-<br>539.42903<br>5	0.1429<br>94	-<br>539.28604<br>1	0.000<br>0	-<br>539.53731<br>4	-<br>539.39432<br>0	-<br>338475.<br>0	-<br>338473.<br>2
(KOtBu3)(KOC(Me)2C<br>H2•) methyl radical<br>ejection	609i	0.776<br>8	-<br>3331.4291<br>42	0.3639<br>60	-<br>3331.0651<br>82	0.777<br>3	-<br>3331.6514<br>08	-<br>3331.2874<br>48	-<br>2090414<br>.4	-<br>2090412<br>.5
(KOtBu3)(KOC(Me)=C<br>H2) + Me•		0.754<br>9	-<br>3331.4569<br>56	0.3622<br>80	-<br>3331.0946<br>76	0.755<br>6	-<br>3331.6799<br>76	-<br>3331.3176<br>96	-<br>2090433<br>.4	-<br>2090431<br>.5
(KOtBu3)(KOC(Me)=C<br>H2) CH abstraction by<br>Me•	157<br>3i	0.761<br>1	-<br>3331.4312<br>90	0.3592<br>00	-<br>3331.0720<br>90	0.761<br>3	-<br>3331.6538<br>88	-<br>3331.2946<br>88	-<br>2090419<br>.0	-<br>2090417<br>.1
[(KOtBu3)(KOC(CH2)2<br>)]• + methane		0.776<br>6	-<br>3331.4736<br>29	0.3597<br>66	-<br>3331.1138<br>63	0.777<br>1	-<br>3331.6969<br>37	-<br>3331.3371<br>71	-<br>2090445<br>.6	-<br>2090443<br>.7
(KOtBu3)(KOC(Me)=C<br>H2)		0.000<br>0	-<br>3291.6282<br>62	0.3382<br>22	-<br>3291.2900<br>40	0.000<br>0	-<br>3291.8423<br>06	-<br>3291.5040<br>84	-<br>2065450<br>.0	-<br>2065448<br>.1

Me•		0.7546	-39.821314	0.001146	-39.820168	0.7547	-39.829922	-39.828776	-24992.9	-24991.0
Me• + anthracene		0.7547	-579.255817	0.160203	-579.095614	0.7550	-579.372269	-579.212066	-363461.1	-363459.2
Me• CH abstraction from anthracene	1473i	0.7675	-579.221821	0.163152	-579.058669	0.7680	-579.338721	-579.175569	-363438.2	-363436.3
methane + 9-anthracenyl radical		0.7761	-579.241862	0.160047	-579.081815	0.7759	-579.359155	-579.199108	-363452.9	-363451.0
methane		0.0000	-40.496954	0.016752	-40.480202	0.0000	-40.506495	-40.489743	-25407.7	-25405.8
phenyl radical C-H abstraction from KOtBu	1405i	0.7622	-3563.636437	0.444657	-3563.191780	0.7626	-3563.904620	-3563.459963	-2236104.9	-2236103.0
benzene + (KOtBu3)(KOC(Me)2CH2•)		0.7560	-3563.670545	0.447531	-3563.223014	0.7564	-3563.938930	-3563.491399	-2236124.6	-2236122.7
Me• + benzene		0.7547	-272.024312	0.077122	-271.947190	0.7548	-272.080043	-272.002921	-170684.4	-170682.5
Me• CH abstraction from benzene	1456i	0.7615	-271.993086	0.078887	-271.914199	0.7617	-272.048694	-271.969807	-170663.6	-170661.7

methane + phenyl radical		0.7621	-272.012972	0.075467	-271.937505	0.7621	-272.069121	-271.993654	-170678.6	-170676.7
attack benzene TS	620i	0.7792	-272.006407	0.088491	-271.917916	0.7800	-272.061381	-271.972890	-170665.6	-170663.7
attack benzene products		0.7817	-272.043671	0.092616	-271.951055	0.7817	-272.098451	-272.005835	-170686.2	-170684.3
attack 1-anthracene TS	623i	0.7983	-579.240993	0.169724	-579.071269	0.7996	-579.357097	-579.187373	-363445.6	-363443.7
attack 1-anthracene products		0.7853	-579.293530	0.175189	-579.118341	0.7856	-579.409184	-579.233995	-363474.8	-363472.9
attack 2-anthracene TS	631i	0.7960	-579.238665	0.169752	-579.068913	0.7975	-579.354956	-579.185204	-363444.2	-363442.3
attack 2-anthracene products		0.7862	-579.286328	0.174801	-579.111527	0.7868	-579.402448	-579.227647	-363470.8	-363468.9
attack 9-anthracene TS	586i	0.8104	-579.244714	0.169111	-579.075603	0.8121	-579.360696	-579.191585	-363448.2	-363446.3

attack 9-anthracene products		0.7788	-579.307015	0.176356	-579.130659	0.7795	-579.422311	-579.245955	-363482.3	-363480.4
abstract 1-anthracene TS	1458i	0.7644	-579.222149	0.161893	-579.060256	0.7647	-579.339015	-579.177122	-363439.1	-363437.2
abstract 1-anthracene products		0.7691	-579.242582	0.166121	-579.076461	0.7689	-579.359828	-579.193707	-363449.5	-363447.6
abstract 2-anthracene TS	1455i	0.7630	-579.221847	0.160947	-579.060900	0.7634	-579.338672	-579.177725	-363439.5	-363437.6
abstract 2-anthracene products		0.7661	-579.242567	0.162000	-579.080567	0.7660	-579.359759	-579.197759	-363452.1	-363450.2
KOtBu tetramer radical cation		0.7589	-3331.914984	0.385133	-3331.529851	0.7616	-3332.136151	-3331.751018	-2090705.3	-2090703.4
iodobenzene radical anion		0.7646	-243.0351272	0.032359	-243.002768	0.7649	-529.3424635	-529.310105	-332147.1	-332145.2
bromoanthracene		0	-3112.99921	0.128192	-3112.871018	0	-3113.191467	-3113.063275	-1953476.7	-1953474.8

bromoanthracene radical anion		0.7661	-3113.068627	0.122587	-3112.946040	0.7668	-3113.259902	-3113.137315	-1953523.2	-1953521.3
Ph rad + PhH	1443i	0.763	-463.6953269	0.124073	-463.571254	0.7634	-463.7886072	-463.664534	-290953.9	-290952.0

References.

(60) Ciriano, M. V.; Korth, H. G.; van Scheppingen, W. B.; Mulder, P. Thermal Stability of 2,2,6,6-Tetramethylpiperidine-1-oxyl (TEMPO) and Related *N*-Alkoxyamines, *J. Am. Chem. Soc.* **1999**, *121*, 6375–6381. https://doi.org/10.1021/ja9837102

(61) Kuninobu, Y.; Tatsuzaki, T.; Matsuki, T.; Takai, K. Indium-Catalyzed Construction of Polycyclic Aromatic Hydrocarbon Skeletons via Dehydration, *J. Org. Chem.* **2011**, *76*, 7005–7009. https://doi.org/10.1021/jo200861s

(62) Stará, I. G.; Starý, I.; Kollárovič, A.; Teplý, F.; Vyskočil, Š.; Šaman, D. Transition metal catalysed synthesis of tetrahydro derivatives of [5]-, [6]- and [7]-helicene, *Tetrahedron Lett.* 1999, **40**, 1993–1996. https://doi.org/10.1016/S0040-4039(99)00099-4

(63) Kaishap, P. P.; Duarah, G.; Sarma, B.; Chetia, D.; Gogoi, S. Ruthenium(II)-Catalyzed Synthesis of Spirobenzofuranones by a Decarbonylative Annulation Reaction, *Angew. Chem. Int. Ed.* **2018**, *57*, 456–460. https://doi.org/10.1002/anie.201710049 S.

(64) Cadge, J. A.; Gates, P. J.; Bower, J. F.; Russell, C. A. Migratory Insertion of CO into a Au–C Bond, *J. Am. Chem. Soc.* **144**, 19719–19725 (2022). https://doi.org/10.1021/jacs.2c10432

(65) Nocera, G.; Young, A.; Palumbo, F.; Emery, K. J.; Coulthard, G.; McGuire, T.; Tuttle, T.; J. A. Murphy, Electron Transfer Reactions: KO*t*Bu (but not NaO*t*Bu) Photoreduces Benzophenone under Activation by Visible Light, *J. Am. Chem. Soc.* **2018**, *140*, 9751–9757. https://doi.org/10.1021/jacs.8b06089

(66) Deng, Y.; Jiang, K.; Cai, M. J.; Qu, S. J.; Dai, Y. R.; Tan, C. H. The synthesis of dendroflorin**,** *J. Asian Nat. Prod. Res.* **2017**, *19*, 602–609. 10.1080/10286020.2017.1324953

(67) Baudet, K.; Guerra, S.; Piguet, C. Chemical Potential of the Solvent: A Crucial Player for Rationalizing Host–Guest Affinities, *Chem. Eur. J.* **2017**, *23*, 16787–16798. https://doi.org/10.1002/chem.201703184

(68) Benniston, A. C.; Copley, G.; Elliott, K. J.; Harrington, R. W.; Clegg, W. *Eur. J. Org. Chem.* **2008**, *16*, 2705–2713. http://dx.doi.org/10.1002/ejoc.200800191

(69) Yamakado, T.; Takahashi, S.; Watanabe, K.; Matsumoto, Y.; Osuka, A.; Saito, S. Conformational Planarization versus Singlet Fission: Distinct Excited-State Dynamics of Cyclooctatetraene-Fused Acene Dimers, *Angew. Chem. Int. Ed.* **2018**, *57*, 5438–5443. https://doi.org/10.1002/anie.201802185

(70) Leifang, L.; Yuhong, Z.; Bingwei, X. *J. Org. Chem.*, **2006**, *71*, 3994–3997. https://doi.org/10.1021/jo060122v

(71) CrysalisPRO. 2016. Rigaku Oxford Diffraction Ltd., Yarnton, England.
(72) Sheldrick, G. M. Crystal Structure Refinement with SHELXL, *Acta Cryst. C.* **2015**, *71*, 3-8. https://doi.org/10.1107/S2053229614024218
(73) Farrugia, L. J. WnGX and ORTEP for Windows: an update. *J Appl. Cryst*. **2012**, *45*, 849–854. https://doi.org/10.1107/S0021889812029111
(74). Gaussian 16, Revision C.01, M. J. Frisch, G. W. Trucks, H. B. Schlegel, G. E. Scuseria, M. A. Robb, J. R. Cheeseman, G. Scalmani, V. Barone, G. A. Petersson, H. Nakatsuji, X. Li, M. Caricato, A. V. Marenich, J. Bloino, B. G. Janesko, R. Gomperts, B. Mennucci, H. P. Hratchian, J. V. Ortiz, A. F. Izmaylov, J. L. Sonnenberg, D. Williams-Young, F. Ding, F. Lipparini, F. Egidi, J. Goings, B. Peng, A. Petrone, T. Henderson, D. Ranasinghe, V. G. Zakrzewski, J. Gao, N. Rega, G. Zheng, W. Liang, M. Hada, M. Ehara, K. Toyota, R. Fukuda, J. Hasegawa, M. Ishida, T. Nakajima, Y. Honda, O. Kitao, H. Nakai, T. Vreven, K. Throssell, J. A. Montgomery, Jr., J. E. Peralta, F. Ogliaro, M. J. Bearpark, J. J. Heyd, E. N. Brothers, K. N. Kudin, V. N. Staroverov, T. A. Keith, R. Kobayashi, J. Normand, K. Raghavachari, A. P. Rendell, J. C. Burant, S. S. Iyengar, J. Tomasi, M. Cossi, J. M. Millam, M. Klene, C. Adamo, R. Cammi, J. W. Ochterski, R. L. Martin, K. Morokuma, O. Farkas, J. B. Foresman, and D. J. Fox, Gaussian, Inc., Wallingford CT, 2016.
(75). Ojima, I. Modern Molecular Approaches to Drug Design and Discovery**,** *Acc. Chem. Res.* **2008**, *41*, 157–167 https://doi.org/10.1021/ar700111a
(76). Blaudeau, J.-P.; McGrath, M. P.; Curtiss, L. A.; Radom, L. Extension of Gaussian-2 (G2) theory to molecules containing third-row atoms K and Ca *J. Chem. Phys.* **1997**, *107*, 5016–5021. https://doi.org/10.1063/1.474865
(77). Curtiss, L. A.; McGrath, M. P.; Blaudeau, J.-P.; Davis, N. E.; Binning, R. C.; Radom, L. Extension of Gaussian-2 theory to molecules containing third-row atoms Ga-Kr *J. Chem. Phys.* **1995**, *103*, 6104–6113. https://doi.org/10.1063/1.470438
(78). Krishnan, R.; Binkley, J. S.; Seeger, R.; Pople, J. A. Self-consistent molecular orbital methods. XX. A basis set for correlated wave functions *J. Chem. Phys.* **1980**, *72*, 650–654. https://doi.org/10.1063/1.438955
(79). Barone, V.; Cossi, M. Quantum calculation of molecular energies and energy gradients in solution by a conductor solvent model, *J. Phys. Chem. A*, **1998**, *102*, 1995–2001. https://doi.org/10.1021/jp9716997
(80). Cossi, M.; Rega, N.; Scalmani, G.; Barone, V. "Energies, structures, and electronic properties of molecules in solution with the C-PCM solvation model," *J. Comp. Chem.*, **2023**, *24*, 669–681. https://doi.org/10.1002/jcc.10189

(81). Fuentealba, P.; Stoll, H.; v. Szentpály, L.; Schwerdtfeger, P.; Preuss, H. On the reliability of semi-empirical pseudopotentials – simulation of Hartree-Fock and Dirac-Fock results, *J. Phys. B*, 1983, **16**, L323-L328. https://doi.org/10.1088/0022-3700/16/11/001

(82). Bergner, A.; Dolg, M.; Kuechle, W.; Stoll, H.; Preuss, H. Ab-initio energy-adjusted pseudopotentials for elements of groups 13-17, *Mol. Phys.*, **1993**, *80*, 1431–1441. https://doi.org/10.1080/00268979300103121

(83). Grimme, S.; Antony, J.; Ehrlich, S.; Krieg, H. A consistent and accurate ab initio parameterization of density functional dispersion correction (DFT-D) for the 94 elements H-Pu, *J. Chem. Phys.* **2010**, *132*, 154104. https://doi.org/10.1063/1.3382344

(84). Rappoport, D.; Furche, F. Property-optimized Gaussian basis sets for molecular response calculations *J. Chem. Phys.* **2010**, *133*, 134105. https://doi.org/10.1063/1.3484283

(85). Weigend, F.; Furche, F.; Ahlrichs, R. Gaussian basis sets of quadruple zeta valence quality for atoms H-Kr. *J. Chem. Phys.* **2003**, *119*, 12753–12762. https://doi.org/10.1063/1.1627293

(86). Harvey, J. N.; Himo, F.; Maseras, F.; Perrin, L Scope and Challenge of Computational Methods for Studying Mechanism and Reactivity in Homogeneous Catalysis, *ACS Catal.* **2019**, 6803–6813. https://doi.org/10.1021/acscatal.9b01537

(87). Yamaguchi, K.; Jensen, F.; Dorigo, A.; Houk, K. N. A spin correction procedure for unrestricted Hartree-Fock and Møller-Plesset wavefunctions for singlet diradicals and polyradicals, *Chem. Phys. Lett.* **1988**, *149*, 537–542. https://doi.org/10.1016/0009-2614(88)80378-6

(88). Yamanaka, S.; Kawakami, T.; Nagao, H.; Yamaguchi, K. Effective exchange integrals for open-shell species by density functional methods, *Chem. Phys. Lett.* **1994**, *231*, 25–33. https://doi.org/10.1016/0009-2614(94)01221-0

(89). Lim, M. H.; Worthington, S. E.; Dulles, F. J.; Cramer, C. J. in *Chemical Applications of Density Functional Theory*, Vol. 629 (Eds. Laird, B. B.; Ross, R. B.; Ziegler, T.), American Chemical Society, Washington DC, (1996) p 402.

(90). Isobe, H.; Takano, Y.; Kitagawa, Y.; Kawakami, T.; Yamanaka, S.; Yamaguchi, K.; Houk, K. N. Extended Hartree-Fock (EHF) theory of chemical reactions VI: hybrid DFT and post-Hartree-Fock approaches for concerted and non-concerted transition structures of the Diels-Alder reaction, *Mol. Phys.* **2002**, *100*, 717–727. https://doi.org/10.1080/00268970110092375

(91). Yu, H.; Fu, Y.; Guo, Q.; Lin, Z. Studies on Reactions of Transition Metal Complexes with O_2, *Organometallics* **2009**, *28*, 4443–4451. https://doi.org/10.1021/om9002957

(92). Álvarez-Moreno, M.; de Graaf, C.; López, N.; Maseras, F.; Poblet, J. M.; Bo, C. Managing the Computational Chemistry Big Data Problem: The ioChem-BD Platform. *J. Chem. Inf. Model.* **2015**, *55*, 95–103.
